# Hypoxia promotes the generation of a versican-rich extracellular matrix by human coronary artery endothelial cells

**DOI:** 10.1016/j.jbc.2025.110459

**Published:** 2025-07-05

**Authors:** Sara M. Jørgensen, Song Huang, Lasse G. Lorentzen, Fallen K.Y. Teoh, Richard Karlsson, John R. Harkness, Rebecca L. Miller, Michael J. Davies, Christine Y. Chuang

**Affiliations:** 1 Panum Institute, Department of Biomedical Sciences, University of Copenhagen, Copenhagen, Denmark; 2 Department of Vascular Surgery, Heart Center, University Hospital Copenhagen - Rigshospitalet, Copenhagen, Denmark; 3 Copenhagen Center for Glycomics, Department of Cellular and Molecular Medicine, University of Copenhagen, Copenhagen, Denmark

**Keywords:** hypoxia, endothelial cell, extracellular matrix, versican, reactive oxygen species, proteomics

## Abstract

Normal endothelial cell (EC) function is essential for vascular wall homeostasis, whereas dysfunction increases the risk of cardiovascular disease. Low O_2_ tension (hypoxia) promotes EC dysfunction and the formation of atherosclerotic plaques. Increasing evidence suggests that hypoxia drives extracellular matrix (ECM) remodeling, an established contributing factor in atherosclerosis. However, the effects of hypoxia on ECs and associated ECM proteins are poorly understood. The aim of this study was to investigate whether the culture of human coronary artery ECs under 1% O_2_ (hypoxia) alters the ECM generated by these cells, and whether this affects HCAEC function. Exposure of HCAECs to 1% O_2_ resulted in a hypoxic response (HIF-1α stabilization), dysfunction (increased oxidant formation and decreased eNOS), and inflammatory activation (increased IL-6 and ICAM-1 expression). Proteomic analysis of HCAECs cultured under 1% and 20% O_2_ for 7 days revealed many hypoxia-induced changes to extracellular proteins, particularly increased versican, a key ECM proteoglycan. Increased versican expression and deposition were confirmed at the mRNA and protein level, along with its glycosaminoglycan (chondroitin sulfate) chains and particularly 6-O-sulfated species. This versican-rich ECM showed increased hyaluronan binding and decreased cell adhesiveness, but attached cells proliferated at a greater rate. The generation of a versican-rich ECM under 1% O_2_ provides a link between hypoxia and atherosclerosis, since versican is reported to accumulate in plaques, where it binds and retains low-density lipoproteins and is involved in inflammatory cell recruitment, thereby potentiating low-density lipoprotein modification and the accumulation of lipid-laden (foam) cells.

The endothelium is a semi-permeable monolayer of endothelial cells (ECs) that line the inside of arteries. Apart from mediating the transfer of O_2_ and nutrients between blood and tissues, the endothelium controls many aspects of vascular wall homeostasis including regulation of vascular tone, hemostasis, inflammation, and angiogenesis (the formation of new blood vessels). In healthy adults, the endothelium is largely quiescent; however, disturbances to EC homeostasis can induce EC dysfunction, an activated state that increases the risk of cardiovascular disease (1). This includes atherosclerosis, a chronic inflammatory disease characterized by the build-up of fibro-fatty plaque in the subendothelial space of arteries (2). Plaques can grow undetected for decades but may undergo sudden rupture or erosion resulting in thrombus or embolus formation, partial or complete occlusion of the artery, and consequent end-organ ischemia (*e.g.* myocardial infarction and ischemic stroke) with a high risk of morbidity or mortality (3).

EC dysfunction is characterized by decreased nitric oxide (NO^•^) bioavailability, proinflammatory and prothrombotic changes, and increased permeability (1). This combination of factors promotes atherosclerotic plaque formation by allowing the accumulation of low-density lipoprotein (LDL) and the recruitment of leukocytes (4). Pro-inflammatory activation of the endothelium involves the expression of leukocyte adhesion molecules (intercellular adhesion molecule-1, vascular adhesion molecule-1, platelet endothelial cell adhesion molecule-1, selectin-E and -P; ICAM-1, VCAM-1, PECAM-1, SELE, and SELP, respectively), cytokines (interleukin-1β, −6, and tumour necrosis factor-α; IL-1β, IL-6, and TNFα, respectively), and chemokines (monocyte chemoattractant protein-1 and interleukin-8; MCP-1 and IL-8, respectively) (2). Low O_2_ tension (hypoxia) is a cardiovascular risk factor for EC dysfunction. Short-term (24–72 h) exposure of endothelial cells to hypoxia (<1% O_2_) *in vitro* decreases endothelial nitric oxide synthase (eNOS) expression, increases inflammatory signaling and cytokine production, and enhances the formation of reactive oxidants (5). Thus, hypoxia may be a contributing factor in atherosclerosis, and this has been reported in human atherosclerotic plaques (6). Cells adapt to hypoxia through the activation of hypoxia-inducible factors (HIFs), a group of transcription factors that control the expression of genes involved in angiogenesis, energy metabolism, and cell survival. Active HIFs are heterodimers of one α- and one β-subunit. All nucleated cells constitutively express HIF-1α, but under normal O_2_ levels, this is targeted for proteasomal degradation. When O_2_ levels decrease, HIF-1α escapes proteasomal degradation and regulates downstream genes (7).

In healthy arteries, ECs produce and associate with a basement membrane of extracellular matrix (ECM) proteins that provide structural support and regulate cellular processes. This basement membrane, composed mainly of laminins, collagen type IV, nidogen, and heparan sulfate proteoglycans, is situated on top of a thin layer of interstitial ECM consisting of fibrillar collagens, chondroitin sulfate proteoglycans, hyaluronan, and elastic fibers (8). Together, ECs and the subendothelial ECM constitute the innermost (intimal) layer of arteries, with changes to EC function and ECM remodeling implicated in the initiation, progression, and complications of atherosclerosis (9, 10, 11). Thus, thickening of the subendothelial ECM and higher concentrations of proteoglycans and hyaluronan can enhance low-density lipoprotein retention and lipid accumulation (9, 12, 13). Deposition of ECM proteins in the subendothelial space also contribute to plaque growth and forms a fibrous cap over the plaque core (14, 15). This cap confers stability, but its degradation due to enhanced inflammation and activated proteases is associated with both the rupture and the erosion of plaques (16, 17).

Increasing evidence indicates that hypoxia drives ECM remodeling, as it can modulate the expression of many ECM proteins, including collagen types I and III (18), versican and perlecan (19, 20), and key ECM regulators such as collagen-modifying enzymes (21, 22, 23) and ECM proteases (24, 25, 26, 27). However, the effects of long-term hypoxia on arterial ECs and associated ECM are poorly understood. This study therefore investigated whether exposure of primary human coronary artery ECs (HCAECs) to hypoxia (1% O_2_) for 7 days alter ECM synthesis and composition, and cell behavior.

## Results

### Exposure to hypoxia results in HIF-1α stabilization, enhances oxidant production and alters expression of genes related to EC dysfunction and activation

**Figure 1:**
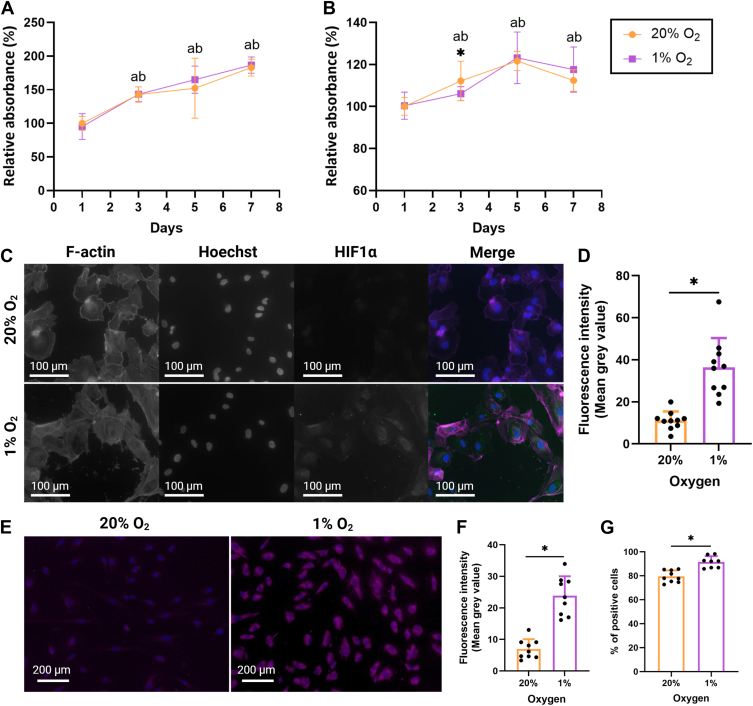
**Viability, proliferation, stabilization of HIF-1α and oxidant generation in HCAECs exposed to 1% O_2_ compared to 20%**. (*A*) cell viability (PrestoBlue) and (*B*) proliferation (BrdU incorporation and ELISA) was measured for HCAECs cultured under 20% or 1% O_2_ over the 24 h time periods: 0 to 1, 2 to 3, 4 to 5, and 6 to 7 days. Cells were cultured in 96-well plates at a density of 1 × 10^4^ cells/well. Data (from three independent experiments with six replicates in each) are presented as the mean ± SD and expressed relative to the 20% O_2_/day one condition. Data was analyzed for statistical difference by a two-way ANOVA with Tukey’s *post hoc* test for multiple comparisons, and significance was assumed at *p* < 0.05. Statistical significance in the comparison between 20% and 1% O_2_ at each time point is annotated by (∗), whereas for the comparison between time points, “a” and “b” are used for 20% and 1% O_2_, respectively. (*C*) HCAEC were cultured for 24 h under either 20% or 1% O_2_ in 8-well chamber slides with a density of 4 × 10^5^ cells/well. Cells were stained for HIF-1α (*green*), F-actin (*magenta*), and nuclei (*blue*). The intracellular localization of HIF-1α is visible in the merged image (representative images from four independent experiments). (*D*) quantification of HIF-1α immunostaining in *panel* (*A*). Data are presented as the mean *grey*-scale intensity ± SD. (*E*) oxidant production by HCAECs was detected with Deep Red ROX dye in HCAECs exposed to 20% or 1% O_2_ for 24 h in 8-well chamber slides with a density of 4 × 10^5^ cells/well (representative data from three independent experiments). (*F*) quantification of Deep Red ROX signal in *panel* (*C*). Data are presented as the mean *grey*-scale intensity ± SD from a total of nine images (images of three different areas of each independent experiment). (*G*) oxidant production, after 7 days of culture in T-175 flasks with 1 × 10^6^ cells/flask, was examined by flow cytometry with Deep *Red* ROX dye. Data (from three independent experiments with triplicate) are presented as the mean percentage of ROX-positive cells ± SD. (*D*), (*F*), and (*G*), data were analyzed for statistical difference by unpaired *t* tests, with significance (∗) assumed at *p* < 0.05.

HCAEC viability was not significantly affected by culture under 1% compared to 20% O_2_ (Fig. 1*A*) for 1 to 7 days. The cellular proliferation rate was determined by bromodeoxyuridine (BrdU) incorporation into newly synthesized DNA over 24-h periods (Day 0–1, Day 2–3, Day 4–5, and Day 6–7). Proliferation was unaffected by O_2_ levels, except for day 2 to 3, where HCAECs proliferated less at 1% compared to 20% O_2_ (Fig. 1*B*).

**Figure 2:**
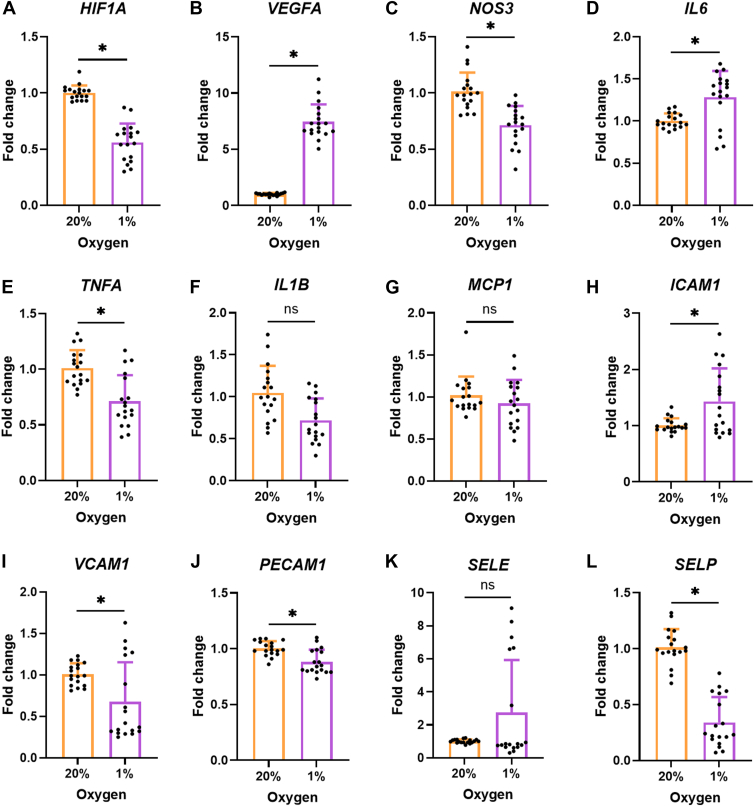
**Expression of genes related to hypoxia, endothelial activation and endothelial dysfunction in HCAECs exposed to long-term hypoxia**. HCAECs were cultured for 7 days in 6-well plates at a density of 5 × 10^4^ cells/well before RNA was extracted for determination of gene expression levels by RT-qPCR. The expression of the following genes was investigated: (*A*) hypoxia-inducible factor 1α (*HIF1A*), (*B*) vascular endothelial growth factor A (*VEGFA*), (*C*) endothelial nitric oxide synthase (*NOS3*), (*D*) interleukin 6 (*IL6*), (*E*) tumor necrosis factor (*TNF*), (*F*) interleukin 1β (*IL1B*), (*G*) monocyte chemoattractant protein 1 (*MCP1*), (*H*) intercellular adhesion molecule 1 (*ICAM1*), (*I*) vascular adhesion factor 1 (*VCAM1*), (*J*) platelet and endothelial cell adhesion molecule (*PECAM1*), (*K*) selectin E (*SELE*), and (*L*) selectin P (*SELP*). Two housekeeping genes, *ACTB* and *18S*, were used. Data (from three independent experiments with six replicates in each) are presented as means ± SD and expressed as a fold change relative to the 20% O_2_ condition. Statistical significance of differentially expressed genes was analyzed by an unpaired *t* test with *p* < 0.05 (∗) accepted as significant.

Cellular adaptation to hypoxia is orchestrated by HIF-1α stabilization (7). HCAECs cultured under 20% or 1% O_2_ for 24 h were immunostained with an anti-HIF-1α antibody, with this resulting in significantly greater immunoreactivity in the HCAECs exposed to 1% O_2_ (Fig. 1, *C* and *D*), consistent with HIF-1α stabilization. Hypoxia, oxidative stress, and inflammation are interlinked processes, and all three can induce EC dysfunction (5). The fluorescent dye, Deep Red ROX, was used to detect oxidant formation in HCAECs in response to 1% O_2_. After 24 h, hypoxic HCAECs showed significantly increased intracellular staining consistent with enhanced oxidant formation (Fig. 1, *E* and *F*). Prolonged hypoxia (7 days), also resulted in significantly increased intracellular oxidation, as assessed by flow cytometry (Fig. 1*G*). HIF-1α stabilization was attenuated at the 7-day time point with HIF-1α mRNA levels decreased by ∼50% when compared to HCAECs cultured under 20% O_2_ (Fig. 2*A*), consistent with previous data (28).

Transient stabilization and activation of HIF-1α modulates the expression of multiple hypoxia-responsive genes, including the angiogenesis-stimulating growth factor, vascular endothelial growth factor A (VEGFA), with the mRNA level of *VEGFA* increased 8-fold on exposure to hypoxia for 7 days (Fig. 2*B*). This condition also reduced the mRNA levels of eNOS (*NOS3*), consistent with EC dysfunction (Fig. 2*C*). At the same time, inflammatory markers of EC activation were significantly increased, including the cytokine interleukin-6 (*IL6*) and the adhesion molecule intercellular adhesion molecule 1 (*ICAM1*; Fig. 2, *D* and *H*). Selectin E (*SELE*) showed an upward trend but failed to reach significance (Fig. 2*K*). In contrast, tumor necrosis factor α (*TNFA*), interleukin-1β (*IL1B*), vascular adhesion molecule 1 (*VCAM1*), platelet and endothelial adhesion molecule 1 (*PECAM1*), and selectin P (*SELP*) were significantly decreased by 7 days of hypoxia (Fig. 2, *E*, *F*, *I*, *J*, and *L*). No change was detected for monocyte chemoattractant protein 1 (*MCP1*; Fig. 2*G*). These data show that HCAECs exposed to prolonged hypoxia exhibit hallmarks of endothelial dysfunction and activation.

### Proteomic analysis reveals multiple changes to extracellular and intracellular proteins in HCAECs exposed to 1% O_2_

**Figure 3:**
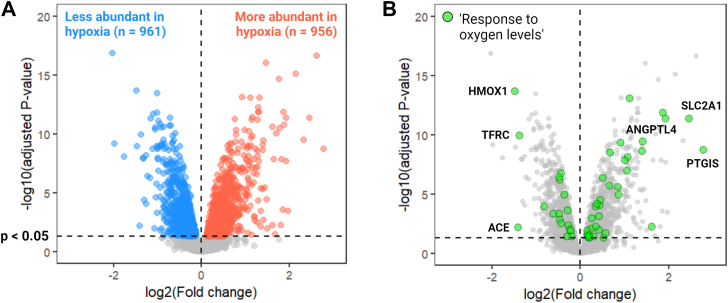
**Proteomic analysis of HCAECs cultured under 20% and 1% O_2_**. HCAECs were cultured for 7 days in 6-well plates at a density of 5 × 10^4^ cells/well under either 20% or 1% O_2,_ followed by cell lysis, protein capture, clean up, and tryptic digestion to peptides. Samples collected from three independent experiments with six replicates were subsequently analyzed by LC-MS/MS to detect and quantify peptides resulting from enzymatic digestion. (*A*) Volcano plot highlighting significantly differentially abundant proteins in HCAECs cultured at 1% compared to 20% O_2_. More abundant proteins are *red*, and less abundant proteins are *blue*. Data was analyzed with a robust linear model and FDR-tested using the Benjamini-Hochberg method (refer to Table S2 for the full list of differentially abundant proteins). (*B*) Volcano plot highlighting proteins from the data set in *panel* A that are annotated with the term *response to oxygen levels* as a biological process (BP) in the gene ontology (GO) database.

Proteomic (Liquid Chromatography-Mass Spectrometry, LC-MS) analysis was carried out to compare the proteomes of HCAECs cultured under 1% *versus* 20% O_2_. HCAECs were cultured for 7 days under 20% or 1% O_2_ after which the cells and ECM material were solubilized. Proteins were isolated, cleaned up and digested to peptides prior to LC-MS analysis. This revealed multiple changes to both extracellular and intracellular proteins. In total, 7811 proteins were detected (intensity-based absolute quantification, IBAQ, values are presented in Table S1). After filtering out single peptide identifications, 6410 proteins were quantified. Of these, 1917 (∼30%) were detected as differentially expressed (*p* < 0.05) between the 1% and 20% O_2_ conditions (Fig. 3*A* and Table S2).

Only ∼3% of the differentially abundant proteins have previously been associated with the gene ontology (GO) biological process (BP): *response to oxygen levels*. Among these were prostacyclin synthase (PTGIS), solute carrier family two member 1 (SLC2A1; also known as GLUT1), and angiopoietin-like four glycoprotein (ANGPTL4), which were more abundant under 1% O_2_, and heme oxygenase 1 (HMOX1), angiotensin-converting enzyme (ACE), and transferrin receptor protein 1 (TFRC; also known as CD71), which were less abundant (Fig. 3*B*). Gene set enrichment analysis (GSEA) resulted in *glycolysis* being the most upregulated biological process in HCAECs cultured under 1% compared to 20% O_2_, and several biological processes related to mitochondrial respiration and translation were downregulated. This is in accordance with previously described metabolic changes in hypoxic cells (29). The top 10 up- and downregulated biological processes are presented in Table 1 and the full list is presented in Table S3. Disease ontology analysis indicated that many of the protein changes in HCAECs cultured under 1% compared to 20% O_2_ are associated with cardiovascular disease, implying a role for hypoxia (Table 2 and the full list is presented in Table S4).

**Table 1:** Gene enrichment analysis (GSEA)

Upregulated biological processes				
Rank	ID	Description	NES	*p*-value
1	GO:0006096	Glycolytic process	2.45	1.28 × 10^−5^
2	GO:0046939	Nucleotide phosphorylation	2.21	1.68 × 10^−5^
3	GO:0061041	Regulation of wound healing	2.07	4.54 × 10^−4^
4	GO:0007517	Muscle organ development	2.03	8.43 × 10^-5^
5	GO:0061061	Muscle structure development	1.91	1.45 × 10^−5^
6	GO:0032409	Regulation of transporter activity	1.90	1.05 × 10^−3^
7	GO:0030198	Extracellular matrix organization	1.90	8.43 × 10^−4^
7	GO:0043062	Extracellular structure organization	1.90	8.43 × 10^−4^
7	GO:0045229	External encapsulating structure organization	1.90	8.43 × 10^−4^
10	GO:0098609	Cell-cell adhesion	1.87	2.73 × 10^−6^

Downregulated biological processes				
Rank	ID	Description	NES	*p*-value
1	GO:0033108	Mitochondrial respiratory chain complex assembly	−2.84	9.69 × 10^−9^
2	GO:0042254	Ribosome biogenesis	−2.82	9.69 × 10^−9^
3	GO:0006364	rRNA processing	−2.74	9.69 × 10^−9^
4	GO:0140053	Mitochondrial gene expression	−2.72	9.69 × 10^−9^
5	GO:0032543	Mitochondrial translation	−2.71	9.69 × 10^−9^
6	GO:0016072	rRNA metabolic process	−2.68	9.69 × 10^−9^
7	GO:0042773	ATP synthesis coupled electron transport	−2.58	2.00 × 10^−8^
8	GO:0042775	Mitochondrial ATP synthesis coupled electron transport	−2.58	2.00 × 10^−8^
9	GO:0042255	Ribosome assembly	−2.49	2.91 × 10^−7^
10	GO:1990542	Mitochondrial transmembrane transport	−2.11	3.09 × 10^−4^

The top 10 up- and downregulated biological processes in hypoxic HCAECs ranked by normalized enrichment score (NES). The full list of enriched terms can be found in Table S3.

**Table 2:** Disease ontology (DO) analysis

Rank	ID	Disease	NES	*p*-value
1	DOID:326	Ischemia	1.79	0.0039
2	DOID:178	Vascular disease	1.76	<0.0001
6	DOID:5844	Myocardial infarction	1.75	0.0071
7	DOID:1287	Cardiovascular system disease	1.75	<0.0001
8	DOID:0050828	Artery disease	1.74	0.0002
18	DOID:3393	Coronary artery disease	1.63	0.0089
19	DOID:10763	Hypertension	1.62	0.0071

Selected disease ontology terms related to cardiovascular disease and hypoxia, ranked by normalized enrichment score (NES). A full list of enriched disease terms can be found in Table S4.

**Figure 4:**
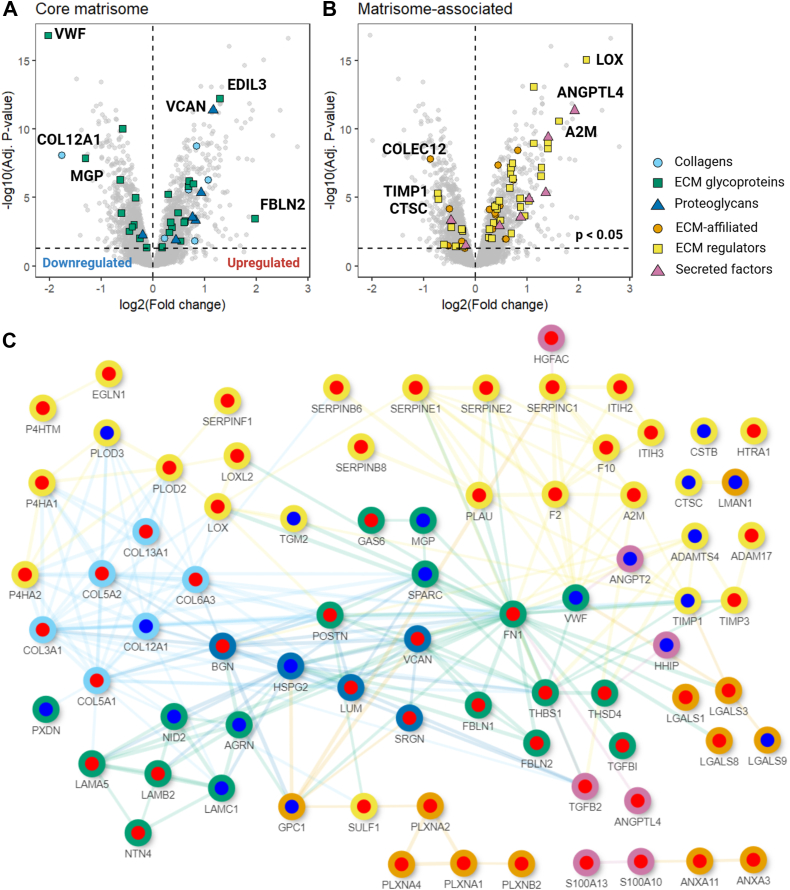
**Changes to matrisome proteins in HCAECs cultured under 1% compared to 20% O_2_**. (*A*) Volcano plot indicating down- and up-regulated core matrisome proteins, and (*B*) matrisome-associated proteins, extracted from HCAECs and associated ECM cultured under 1% O_2_ relative to the 20% condition. The division of the proteins into different classes is indicated by the color-coding shown in the *panel*. Refer to Table S8 for the full list of changes to the matrisome proteins. (*C*) network plot indicating associations between the down- (symbols with a *blue center*), and up- (symbols with a *red center*) regulated core matrisome, and matrisome-associated proteins. The outer circles of each symbol indicate the assigned division of the proteins using the same notation as used in *panels* (*A* and *B*).

*Extracellular matrix organization* was the seventh most upregulated biological process in hypoxic HCAECs (Table 1), and consequently, changes in ECM protein levels were assessed, with 175 matrisome proteins quantified (using the divisions defined in (30); Table S5; IBAQ values in Tables S6 and S7). Comparison of the data from HCAECs cultured under 1% and 20% O_2_ showed 97 significant (adjusted *p*-value < 0.05) differentially abundant matrisome proteins, including 38 core matrisome species (25 up- and 13 downregulated) and 59 matrisome-associated proteins (44 up- and 15 downregulated; Table S8, Fig. 4, *A* and *B*). The top 20 up- and downregulated core matrisome and matrisome-associated proteins are summarized in Tables 3 and 4, respectively. A network plot of associations between these proteins is presented in Figure 4*C*.

**Table 3:** List of the top 20 up- and down-regulated core matrisome proteins in HCAECs cultured under 1% O_2_ compared to 20% O_2_

Top 20 upregulated core matrisome proteins						Top 20 downregulated core matrisome proteins					
#	Protein (gene)	UniProt ID	Log2-fold change	Adj. *p*-value	Category	#	Protein (gene)	UniProt ID	Log2-fold change	Adj. *p*-value	Category
1	Fibulin 2 (FBLN2)	P98095	1.97	3.34 × 10^−4^	ECM glycoproteins	1	Von Willebrand Factor (VWF)	P04275	−2.03	1.41 × 10^−17^	ECM glycoproteins
2	EGF-like repeats and discoidin I-like domains 3 (EDIL3)	O43854	1.29	5.64 × 10^−13^	ECM glycoproteins	2	Collagen XII alpha 1 (COL12A1)	Q99715	−1.76	8.19 × 10^−9^	Collagens
3	Versican (VCAN)	P13611	1.16	3.99 × 10^−12^	Proteoglycans	3	Matrix Gla protein (MGP)	P08493	−1.31	1.30 × 10^−8^	ECM glycoproteins
4	Collagen type XIII alpha 1 (COL13A1)	Q5TAT6	1.07	5.52 × 10^−7^	Collagens	4	Von Willebrand Factor A domain containing 5A (VWA5A)	O00534	−0.64	4.89 × 10^−7^	ECM glycoproteins
5	Lumican (LUM)	P51884	0.93	4.14 × 10^−6^	Proteoglycans	5	Cysteine-rich with EGF-like domains 2 (CRELD2)	Q6UXH1	−0.61	1.33 × 10^−4^	ECM glycoproteins
6	Collagen type V alpha 2 (COL5A2)	P05997	0.85	1.84 × 10^−9^	Collagens	6	Multimerin 1 (MMRN1)	Q13201	−0.59	9.48 × 10^−11^	ECM glycoproteins
7	p53-responsive gene 4 (PRG4)	Q92954	0.82	4.72 × 10^−4^	Proteoglycans	7	Nidogen 2 (NID2)	Q14112	−0.46	2.84 × 10^−3^	ECM glycoproteins
8	Collagen type VI alpha 3 (COL6A3)	P12111	0.81	0.01	Collagens	8	Sushi-repeat containing protein, X-linked (SRPX)	P78539	−0.41	1.38 × 10^−3^	ECM glycoproteins
9	Netrin 4 (NTN4)	Q9HB63	0.78	9.71 × 10^−7^	ECM glycoproteins	9	Secreted protein acidic cysteine-rich, osteonectin (SPARC)	P09486	−0.37	9.84 × 10^−4^	ECM glycoproteins
10	Serglycin (SRGN)	P10124	0.76	2.74 × 10^−4^	Proteoglycans	10	Agrin (AGRN)	O00468	−0.34	1.03 × 10^−5^	ECM glycoproteins
11	Growth arrest-specific 6 (GAS6)	Q14393	0.70	6.27 × 10^-7^	ECM glycoproteins	11	Peroxidasin (PXDN)	Q92626	−0.26	9.74 × 10^−3^	ECM glycoproteins
12	Collagen type III alpha 1 (COL3A1)	P02461	0.69	2.66 × 10^−6^	Collagens	12	Heparan sulfate proteoglycan 2 (HSPG2)	P98160	−0.21	5.61 × 10^−3^	Proteoglycans
13	Fibulin 1 (FBLN1)	P23142	0.69	1.47 × 10^−6^	ECM glycoproteins	13	Laminin, gamma 1 (LAMC1)	P11047	−0.13	4.58 × 10^−2^	ECM glycoproteins
14	Hemicentin 1 (HMCN1)	Q96RW7	0.62	5.14 × 10^−4^	ECM glycoproteins	14					
15	Thrombospondin, type I, domain containing 4 (THSD4)	Q6ZMP0	0.60	6.56 × 10^−4^	ECM glycoproteins	15					
16	Tubulointerstitial nephritis antigen-like 1 (TINAGL1)	Q9GZM7	0.54	1.49 × 10^−2^	ECM glycoproteins	16					
17	Transforming growth factor, beta-induced (TGFBI)	Q15582	0.48	1.27 × 10^−4^	ECM glycoproteins	17					
18	Biglycan (BGN)	P21810	0.44	1.32 × 10^−2^	Proteoglycans	18					
19	ABI family, member 3 (NESH) binding protein (ABI3BP)	Q7Z7G0	0.37	1.49 × 10^−3^	ECM glycoproteins	19					
20	Fibronectin 1 (FN1)	P02751	0.34	6.21 × 10^−4^	ECM glycoproteins	20					

A full list of upregulated and downregulated core matrisome proteins can be found in Table S8.

**Table 4:** List of the top 20 up- and down-regulated matrisome-associated proteins in HCAECs cultured under 1% O_2_ compared to 20% O_2_

Top 20 upregulated matrisome-associated proteins						Top 20 downregulated core matrisome proteins					
#	Protein (gene)	UniProt ID	Log2-fold change	Adj. *p*-value	Category	#	Protein (gene)	UniProt ID	Log2-fold change	Adj. *p*-value	Category
1	Lysyl oxidase (LOX)	P28300	2.15	8.36 × 10^−7^	ECM regulators	1	Collectin sub-family member 12 (COLEC12)	Q5KU26	−0.86	1.50 × 10^−8^	ECM-affiliated
2	Angiopoietin-like 4 (ANGPTL4)	Q9BY76	1.93	4.26 × 10^−12^	Secreted factors	2	TIMP metallopeptidase inhibitor 1 (TIMP1)	P01033	−0.73	4.70 × 10^−6^	ECM regulators
3	Alpha-2-macroglobulin (A2M)	P01023	1.62	2.54 × 10–11	ECM regulators	3	Cathepsin C (CTSC)	P53634	−0.72	1.23 × 10^−5^	ECM regulators
4	Transforming growth factor beta 2 (TGFB2)	P61812	1.41	3.68 × 10^−10^	Secreted factors	4	ADAM with thrombospondin type 1 motif 4 (ADAMTS4)	O75173	−0.61	2.73 × 10^−2^	ECM regulators
5	Plasminogen activator urokinase (PLAU)	P00749	1.40	2.50 × 10^−9^	ECM regulators	5	Galectin 9 (LGALS9)	O00182	−0.52	3.47 × 10^−2^	ECM-affiliated proteins
6	Antithrombin (SERPINC1)	P01008	1.40	8.82 × 10^−10^	ECM regulators	6	Glypican 1 (GPC1)	P35052	−0.50	7.14 × 10^−5^	ECM-affiliated proteins
7	Growth differentiation factor 6 (GDF6)	Q6KF10	1.36	4.21 × 10^−6^	Secreted factors	7	Angiopoietin 2 (ANGPT2)	O15123	−0.47	4.72 × 10^−4^	Secreted factors
8	Coagulation factor X (F10)	P00742	1.28	6.07 × 10^−8^	ECM regulators	8	ADAM metallopeptidase domain 15 (ADAM15)	Q13444	−0.47	1.42 × 10^−3^	ECM regulators
9	Inter-alpha (globulin) inhibitor H3 (ITIH3)	Q06033	1.27	2.59 × 10^−7^	ECM regulators	9	Family with sequence similarity 20 member B (FAM20 B)	O75063	−0.39	3.60 × 10^−2^	ECM regulators
10	Inter-alpha (globulin) inhibitor H2 (ITIH2)	P19823	1.13	1.05 × 10^−9^	ECM regulators	10	Transglutaminase 2 (TGM2)	P21980	−0.27	1.99 × 10^−3^	ECM regulators
11	Procollagen-lysine, 2-oxoglutarate 5-dioxygenase 2 (PLOD2)	O00469	1.13	8.09 × 10^−14^	ECM regulators	11	Lectin, mannose-binding 1 (LMAN1)	P49257	−0.26	1.66 × 10^−2^	ECM-affiliated
12	HGF activator (HGFAC)	Q04756	1.04	1.26 × 10^−5^	Secreted factors	12	Procollagen-lysine, 2-oxoglutarate 5-dioxygenase 3 (PLOD3)	O60568	−0.25	2.65 × 10^−3^	ECM regulators
13	Serpin peptidase inhibitor, clade E, member 2 (SERPINE2)	P07093	1.04	1.67 × 10^−5^	ECM regulators	13	Cystatin B (CTSB)	P04080	−0.25	3.83 × 10^−2^	ECM regulators
14	Coagulation factor II, thrombin (F2)	P00734	0.95	2.11 × 10^−4^	ECM regulators	14	Annexin A4 (ANXA4)	P09525	−0.20	4.96 × 10^−2^	ECM-affiliated
15	S100 calcium binding protein A10 (S100A10)	P60903	0.88	2.85 × 10^−4^	Secreted factors	15	Hedgehog interacting protein (HHIP)	Q96QV1	−0.19	3.24 × 10^−2^	Secreted factors
16	TIMP metallopeptidase inhibitor 3 (TIMP3)	P35625	0.87	4.87 × 10^−5^	ECM regulators	16					
17	Lysyl oxidase-like 2 (LOXL2)	Q9Y4K0	0.87	1.11 × 10^−5^	ECM regulators	17					
18	Annexin A3 (ANXA3)	P12429	0.83	3.81 × 10^−9^	ECM- affiliated	18					
19	C-type lectin domain family 3, member B (CLEC3B)	P05452	0.74	5.27 × 10^−7^	ECM-affiliated	19					
20	Serpin peptidase inhibitor, clade F, member 1 (SERPINF1)	P36955	0.73	4.20 × 10^−7^	ECM regulators	20					

A full list of upregulated and downregulated matrisome-associated proteins can be found in Table S8.

The third most upregulated core matrisome protein, and the most upregulated proteoglycan, in the ECM generated by hypoxic HCAECs was versican (VCAN; Table 3 and Fig. 4*A*). VCAN has been described to be upregulated under hypoxic conditions in other cells (macrophages and vascular smooth muscle cells (19, 20)). VCAN is dramatically elevated in the subendothelial space in many vascular diseases, including pulmonary hypertension and atherosclerotic plaques, where it has been associated with the accumulation and retention of LDL (31). VCAN has previously been studied extensively in vascular smooth muscle cells in relation to atherosclerosis but not in ECs. We therefore investigated the synthesis and secretion of VCAN under 1% O_2_.

### Hypoxia increases the synthesis and deposition of versican by HCAECs

**Figure 5:**
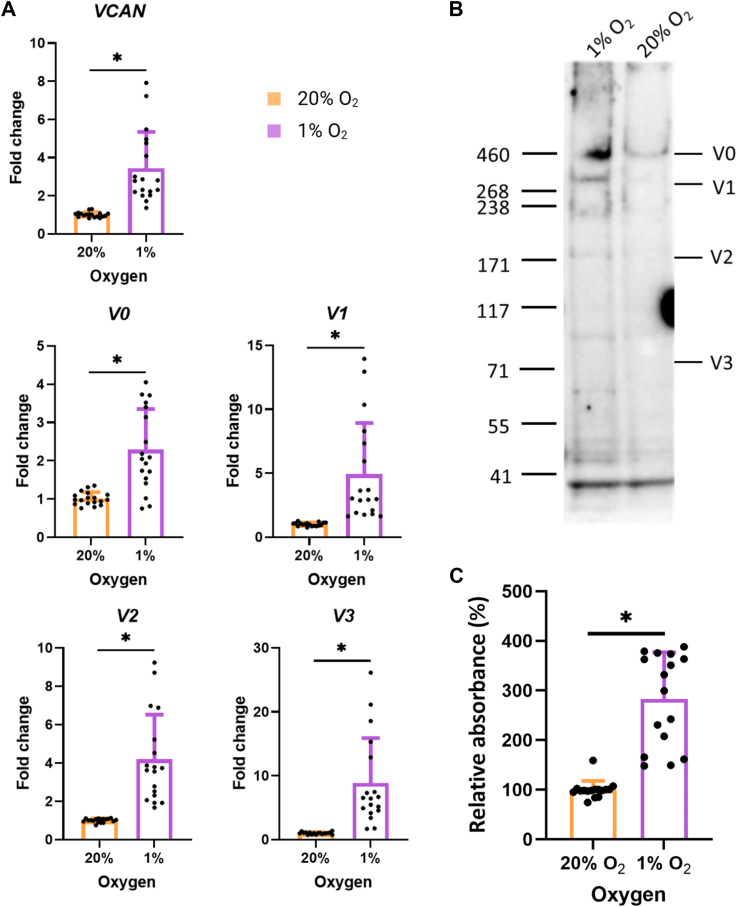
**Expression of versican (VCAN) by HCAECs cultured under 20% or 1% O_2_**. HCAECs were cultured for 7 days in 6-well plates at a density of 5 × 10^4^ cells/well before RNA was extracted for determination of gene expression levels by RT-qPCR. (*A*) detection of total *VCAN* mRNA levels and the levels of different VCAN isoforms (V0, V1, V2 and V3) by RT-qPCR in HCAEC after incubation under 20% or 1% O_2_ for 7 days. Two housekeeping genes, β-actin and 18S, were used. Data (from three independent experiments with six replicates in each) are presented as means ± SD and are expressed as fold change relative to the 20% O_2_ condition. Statistical differences were analyzed by an unpaired *t* test. In both *panelA* and *B*, *p* < 0.05 (∗) was accepted as significant. (*B*) HCAECs were cultured in T-175 flasks (density of 1 × 10^6^ cells/flask) for 7 days under 20% or 1% O_2_. VCAN (anti-G1-domain) was detected by immunoblotting in the total precipitated proteins from HCAECs and its ECM. Representative data from one of two independent experiments (Fig. S1). (*C*) HCAECs were cultured in 96-well plates (density of 1 × 10^4^ cells/well) for 7 days under 20% or 1% O_2_. The ECM was decellularized prior to detection of VCAN (anti-G1-domain) by ELISA. Data (from three independent experiments with 4–6 replicates in each) are presented as means ± SD and are expressed relative to the 20% O_2_ ECM. Statistical differences were analyzed by an unpaired *t* test. In both *panel* (*A* and *C*), *p* < 0.05 (∗) was accepted as significant.

To investigate the effect of 1% O_2_ on the synthesis of VCAN by HCAECs, RNA was extracted after 7 days of culture under 20% or 1% O_2_ for quantitative PCR. Total VCAN mRNA was increased by ∼4-fold in HCAECs cultured under 1% O_2_ compared to 20% (Figure 5*A*). Alternative splicing of VCAN can generate four different isoforms (V0, V1, V2, and V3) (32). The mRNAs of all isoforms were increased under 1% O_2_, with the most marked increase being for V3 (V0 ∼ 2-fold, V1 and V2 ∼ 4-fold, V3 ∼ 8-fold in Fig. 5*A*).

Immunoblotting experiments using an antibody against the VCAN G1 domain confirmed these data. The isoform distribution was determined based on their differing molecular masses arising from the variable expression of the chondroitin sulfate (CS)-binding domains CSα and CSβ [V0 ∼ 370 kDa, due to the presence of CSα and CSβ; V1 ∼ 260 kDa (only CSβ), V2 ∼ 180 kDa (only CSα), and V3 ∼ 70 kDa (neither CSα nor CSβ)]. Samples were treated with chondroitinase ABC before SDS-PAGE, to remove attached CS chains, which would otherwise confound analysis. HCAECs cultured under 1% O_2_ showed immunoreactivity at ∼460 kDa (V0), ∼270 kDa (V1), ∼170 kDa (V2), ∼100 kDa, ∼65 kDa (possibly V3, as a previous study has reported V3 to migrate at ∼65 kDa (33)) and <55 kDa (Fig. 5*B*), whereas HCAECs cultured under 20% O_2_ only showed immunoreactivity at 460 kDa. These data are consistent with increased expression of multiple different isoforms of VCAN (V0-V2, and probably V3) under 1% O_2_ (Fig. 5*B*).

To investigate deposition of VCAN into the ECM, HCAECs were cultured at 20% or 1% O_2_ followed by decellularization. The VCAN content in the ECM remaining on the plate was evaluated by ELISA using a G1 domain antibody. The decellularized ECM from the 1% O_2_ condition contained ∼3 times more VCAN than ECM produced under 20% O_2_ (Fig. 5*C*). Together, these data demonstrate that HCAECs synthesize and deposit increased amounts of VCAN into the ECM under 1% O_2_.

### Hypoxia alters the synthesis of chondroitin sulfate glycosaminoglycan chains by HCAECs

**Figure 6:**
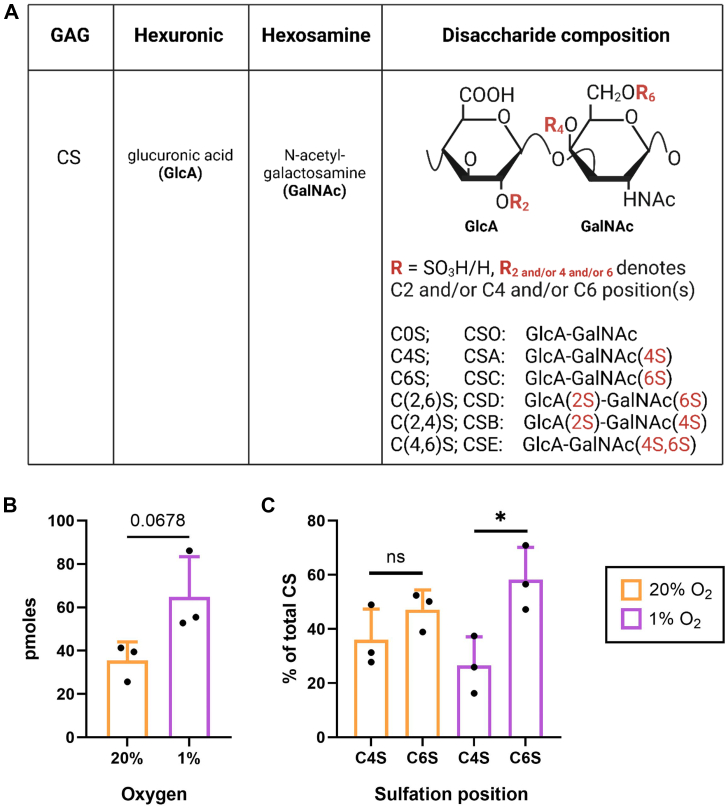
**Chondroitin sulfate (CS) disaccharide content in HCAECs cultured under 20% or 1% O_2_**. HCAECs were cultured for 7 days in T-175 flasks (density of 1 × 10^6^ cells/flask) under 20% or 1% O_2_ before isolation and digest of CS. The disaccharides originating from CS were quantified by fluorescence chromatography. (*A*) structure and nomenclature of CS disaccharide species, where R denotes the position of sulfation (shown in *red*). (*B*) total CS disaccharides in HCAECs presented as mean picomoles ± SD and analyzed for statistical difference by an unpaired *t* test. (*C*) GlcA-GalNAc(4S) and GlcA-GalNAc(6S) content (denoted as C4S and C6S respectively) in HCAECs presented as mean percentage of the total CS content ± SD and analyzed for statistical difference by Tukey’s tests, with *p* < 0.05 (∗) accepted as significant. Data from three independent experiments.

CS chains covalently attached to VCAN V0, V1, and V2 consist of repeating disaccharide units of glucuronic acid (GlcA) and N-acetylgalactosamine (GalNAc) (34). In contrast, heparan sulfate (HS) is associated with HS proteoglycans (*e.g.* perlecan). These disaccharide units can be sulfated at different sites and to different extents (Fig. 6*A* for CS, Fig. S2*A* for HS), conferring functional differences. The content of CS and HS, as well as their sulfation patterns, was quantified in HCAECs cultured under 1% or 20% O_2_ by fluorescence chromatography. No statistical differences were detected in the total HS content, nor HS sulfation pattern (Fig. S2, *B* and *C*). A trend toward increased CS disaccharides was detected for HCAECs under 1% compared to 20% O_2_ (Fig. 6*B*), as well as a significantly increased C6S: C4S ratio for HCAECs cultured under 1% (Fig. 6*C*). This indicates that HCAECs cultured under 1% O_2_ generate more C6S- than C4S-containing disaccharides than under 20% O_2_ (Fig. S3).

### HCAEC-ECM derived from HCAECs cultured under 1% O_2_ have a higher capacity for hyaluronan binding

**Figure 7:**
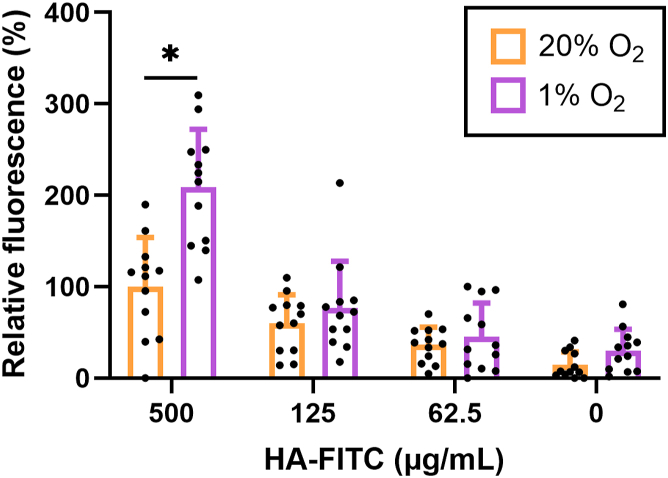
**Hyaluronan (HA) binding to ECM laid down by HCAECs cultured under 20% or 1% O_2_**. HCAECs were cultured for 7 days in 96-well plates (density of 1 × 10^4^ cells/well) under 20% or 1% O_2_ to produce ECM prior to decellularization. HA-FITC (50 kDa) was added to the ECM at three different concentrations (62.5, 125 and 500 μg mL^−1^). Negative control wells received no HA (0 μg mL^−1^). Bound HA-FITC was detected by fluorescence from the FITC tag using λ_ex_ 480 nm, λ_em_ 535 nm. Data (from three independent experiment with four replicates), minus medium only (no ECM) background controls, are presented as means ± SD relative to the 500 μg mL^−1^ + 20% O_2_ condition. Statistical significance between the two O_2_ conditions was analyzed using t-tests corrected for multiple comparisons by the Holm-Sidak method, with *p* < 0.05 (∗) accepted as significant.

Hyaluronan (HA) is a non-sulfated GAG that associates non-covalently with hyalectins including VCAN (32). Consequently, we hypothesized that HCAEC-ECM generated under 1% O_2_ would have an increased HA-binding capacity due to its increased VCAN content. To investigate this, FITC-labeled HA (at three different concentrations, with a HA-FITC standard curve presented in Fig. S4*A*) was incubated with decellularized ECM (to avoid binding to cell-surface receptors) produced under 1% or 20% O_2_. HA-FITC binding was detected by fluorescence, with this being significantly increased to ECM generated under 1% compared to 20% O_2_ at the highest HA-FITC concentration used (500 μg mL^−1^; Fig. 7), where saturation of fluorescence signal for hypoxic ECM was reached at 500 μg mL^−1^ (Fig. S4*B*)

### HCAEC-ECM generated under 1% O_2_ is less adhesive and alters the proliferation of HCAECs

**Figure 8:**
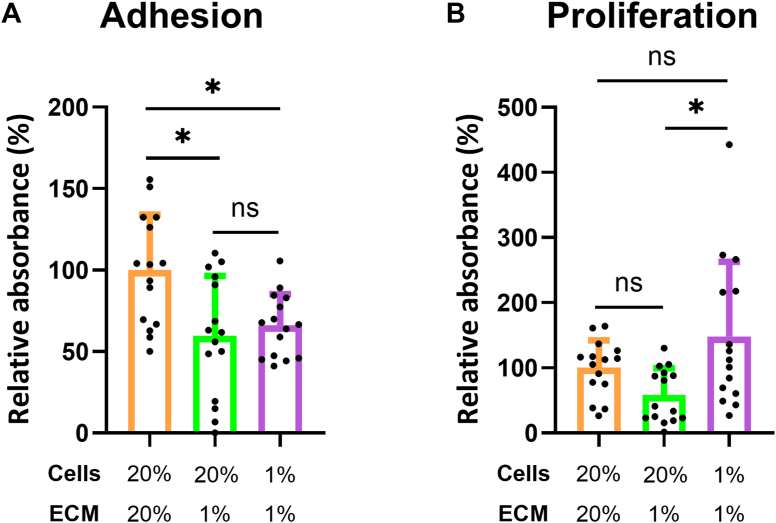
**Effects of hypoxia-induced HCAEC-ECM on HCAEC adhesion and proliferation.** (*A*) HCAECs were cultured for 7 days in black, clear-bottom 96-well plates (density of 1 × 10^4^ cells/well) under 20% or 1% O_2_ to produce ECM. A new passage of HCAECs (cultured at either 20% or 1% O_2_) stained with calcein-AM were seeded onto the decellularized HCAEC-ECM and adhesion after 1.5 h quantified by the fluorescence intensity from adherent cells after washing to remove unattached cells. (*B*) Adherent HCAECs from (*A*) were subsequently cultured for a further 72 h under either 20% or 1% O_2_ before measurement of metabolic activity using the MTS assay as a proxy for proliferation. Data (from three independent experiments with five replicates in each), minus medium only (no ECM) background controls, are presented as means ± SD and are expressed relative to HCAECs cultured at 20% O_2_ on HCAEC-ECM produced under 20% O_2_. Statistical significance was analyzed by one-way ANOVA and corrected for multiple comparisons with Tukey’s *post hoc* test with *p* < 0.05 (∗) accepted as significant.

VCAN isoforms carrying CS chains are anti-adhesive and pro-proliferative (35), and consequently, we hypothesized that the 1% O_2_ HCAEC-ECM enriched in VCAN, and particularly CS-carrying isoforms, would modulate HCAEC adhesion and proliferation. HCAECs were pre-stained with fluorescent calcein-AM dye and incubated on decellularized ECM synthesized under either 1% or 20% O_2_. The hypoxia-induced ECM gave rise to significantly decreased adhesion when compared to that generated under 20% O_2_, regardless of the O_2_ tension at which the HCAECs were incubated during the experiment (Fig. 8*A*). These data indicate that the hypoxia-induced ECM is less adhesive than that generated under 20% O_2_, potentially because of its altered VCAN content. The cells that adhered to the decellularized ECM under these conditions were then cultured for a further 72 h, with the metabolic activity of the cells then measured, to assess their proliferation in response to the different ECM compositions. HCAECs cultured under 1% O_2_ on the VCAN-rich ECM generated by HCAECs under 1% O_2_ proliferated significantly more rapidly than HCAECs cultured under 20% O_2_ on the same matrix (Fig. 8*B*). However, proliferation of the HCAECs cultured under 20% O_2_ and 1% O_2_ on the 1% O_2_-generated ECM, was not significantly different from HCAECs cultured under 20% O_2_ on the ECM generated at 20% O_2_ (Fig. 8*B*).

## Discussion

Hypoxia is associated with several pathologies including atherosclerosis, where ECs are involved in both the initiation and progression of the disease. EC dysfunction occurs prior to plaque formation, whereas sprouting angiogenesis (new blood vessel formation from existing vessels), which occurs in the advanced plaques, involves the migration and proliferation of ECs, with hypoxia regulating both processes. To model key aspects of human vascular pathology, we utilized primary HCAECs isolated directly from coronary vessels, preserving vessel- and organ-specific characteristics critical to cardiovascular physiology. These cells were cultured under 1% O_2_ to replicate the hypoxic microenvironment characteristic of advanced atherosclerotic plaques (6), where oxygen diffusion is impaired due to decreased blood flow through the smaller arterial lumen and the heightened metabolic demand from infiltrating immune cells. The physiological relevance of this model is supported by the observed hypoxia-induced stabilization of HIF-1α and downstream transcriptional and functional responses reflective of endothelial dysfunction *in vivo* (7), including reduced eNOS expression, elevated IL-6 and ICAM-1 levels, and increased intracellular oxidative stress. These features are hallmarks of the inflamed and dysfunctional endothelium observed in human atherosclerosis. In addition, our proteomic analysis revealed upregulation of extracellular matrix proteins such as versican, fibulin-1/2, biglycan, and TGFβ-related proteins, which are molecules implicated in vascular remodeling and plaque development. Notably, many of these changes parallel those reported in proteomic studies of human atherosclerotic plaques (36), further reinforcing the physiological relevance of our *in vitro* system in recapitulating some of the key features of the cellular and extracellular landscape during plaque progression.

In this study, we show that 7-day exposure to 1% O_2_ induces the production of a VCAN-rich ECM by HCAECs, which is structurally and functionally distinct to that produced under 20% O_2_, with this resulting in increased HA binding and decreased cell adhesiveness.

Hypoxia has been reported previously to increase mRNA levels of VCAN isoforms V0 and V1 in VSMCs, but decrease V2 mRNA, consistent with selective splicing between the exons encoding the CSα and CSβ domains under hypoxia (20). Hypoxia has also been reported to increase VCAN expression in macrophages in a HIF-1α-dependent manner (19). However, there is limited data on the effects of hypoxia on the ECM produced by ECs. The present study indicates that ECs may be a significant source of VCAN when exposed to low O_2_ levels, with the mRNA levels for all four VCAN isoforms (V0-V3) increased in HCAECs exposed to 1% compared to 20% O_2_. An increase in the CS-carrying isoforms (V0-V2) was confirmed at the protein level, and indicative evidence for the V3 isoform by immunoblotting. A previous study reported transcription of V0-V2 in resting ECs but without protein expression (33). However, upon activation with TNFα or VEGFA, ECs also transcribed V3 and expressed all four isoforms at the protein level. We detected increased *VEGFA* gene expression in HAECs exposed to 1% O_2_, which may link decreased O_2_ levels and the induction of VCAN gene and protein expression, including the V3 isoform.

VCAN is present in the ECM of normal blood vessels but increases dramatically upon arterial injury and disease (9). The intimal thickening that occurs prior to the development of an atherosclerotic plaque is enriched in proteoglycans, including VCAN, biglycan, and lumican (37). This increase in proteoglycans has been associated with intimal VSMCs, but our current data indicate that ECs may contribute under hypoxic conditions. VCAN, biglycan, and lumican are all CS-proteoglycans. CS is known to interact with apolipoprotein B100 of LDL (12), leading to retention of LDL in the artery wall (13, 38) and linking increased VCAN to the progression of atherosclerosis. Conditions that promote CS chain elongation or enhance the extent or sites of sulfation (c.f. data indicating that C6S binds LDL, but not C4S (39)) would therefore increase arterial LDL retention and promote atherosclerosis. In our study, HCAECs exposed to hypoxia showed a trend towards higher amounts of CS disaccharides, with increased C6S and decreased C4S. This trend is supported by our proteomic data, where the enzymes predominantly responsible for CS elongation (CHSY1) and 6-O-sulfation (CHST3 (34)) were more abundant in HCAECs exposed to 1% O_2_ (Table S2); a similar effect has been observed in macrophages (40). This suggests that HCAECs subjected to hypoxia not only generate more CS-proteoglycans but also synthesize CS-species with a higher affinity for LDL, providing additional links between hypoxia and plaque development.

ECM content and composition are not only determined by ECM synthesis but also remodeling and ECM degradation. Multiple ECM proteases and their inhibitors were altered in the proteome of HCAECs exposed to 1% O_2_. Among these was “a disintegrin and metalloproteinase with thrombospondin motifs-4” (ADAMTS4), a metalloproteinase that cleaves VCAN (41), which was less abundant in hypoxic HCAECs (Table 4). Decreased ADAMTS4 implies that decreased degradation may contribute, in addition to increased synthesis, to the increased VCAN content of hypoxic ECM.

VCAN can regulate the ability of cells to adhere, proliferate, migrate, and remodel the ECM (42), in an isoform- and CS-chain dependent manner. The ability to decrease cell adhesion, while increasing proliferation and migration has been ascribed to the larger CS-carrying isoforms (V0-V2; except for V2 in neuronal tissue) (35, 43, 44). In contrast, V3 which carries no CS chains, has a positive effect on cell adhesion and an inhibitory effect on proliferation and migration (42, 45, 46). In the present study, the hypoxia-induced VCAN-rich ECM decreased HCAEC adhesion regardless of O_2_ levels, which could be a result of the increased levels of CS-carrying isoforms of VCAN (or other CS-proteoglycans) present in hypoxic conditions.

VCAN and its binding partner HA, accumulate in proliferating tissues, where they form an anti-adhesive malleable pericellular ECM, which allows changes in cell shape required for division (47). An enhanced rate of proliferation was detected for HCAECs under 1% O_2_ on hypoxia-induced VCAN-rich ECM. However, this was not observed for HCAECs under 20% O_2_ on the same ECM. This could be due to lack of specific growth factors or mitogens under 20% O_2_, as previous data indicate that VCAN accumulation is necessary but not sufficient to stimulate proliferation (42). VEGFA is an important mitogen in ECs (48), and in the present study, hypoxic HCAECs had increased levels of VEGFA mRNA. It is possible that VEGFA produced under 1% O_2_, together with the VCAN-rich ECM, enabled increased proliferation, whereas the ECM was not permissive for enhanced proliferation under 20% O_2_ due to insufficient VEGFA.

VCAN is known to regulate migration and invasiveness in tumors (49), another hypoxic environment. We identified *cell migration* as an upregulated process in the hypoxic HCAECs based on our proteomic data. Under these conditions, fibulin-1 (FBLN1) and −2 (FBLN2) was more abundant. These proteins are binding partners of VCAN, and are associated with vascular development and remodeling (50). FBLN2 and VCAN have been reported to co-localize in both aortic atherosclerotic plaques and mechanically injured carotid arteries in mice, and FBLN2 has been reported to be upregulated in migrating murine VSMCs *in vitro*, where interference with FBLN2-VCAN interactions inhibited migration (51). It is possible that the increase in FBLN2 (and FBLN1) supports HCAEC migration together with VCAN. To our knowledge, FBLN1 and FBLN2 have not previously been associated with hypoxia. Instead, fibulin five expression has been reported to be increased in ECs by hypoxia in a HIF-1α-dependent manner (52), though fibulin five was not detected in the current study.

Angiogenesis is a common hypoxia-induced response, with this involving EC proliferation and migration, sprouting from existing vessels, and ECM remodeling (53). In atherosclerotic plaques, new vessels sprouting from the vasa vasorum are linked to disease progression and plaque instability (54). The observed effects of VCAN on cell adhesion, proliferation, and migration could indicate an involvement in angiogenic processes, consistent with tumor data. Reduction of VCAN expression in mice can attenuate tumor angiogenesis (55), and transfection of tumor cells with the V2 isoform results in extensive tumor vasculature (56). The VCAN G3 domain is implicated directly in angiogenesis, with tumor cells expressing a G3 construct showing increased expression of fibronectin and VEGFA, and the ability to increase EC proliferation and migration (57). Consistent with these reports, our data show consistent upregulation of VCAN, fibronectin (Table 3), and VEGFA under 1% O_2_.

Other matrisome proteins detected by proteomics in this study have also been linked to angiogenesis, with biglycan, collagen VI and transforming growth factor beta (TGFβ) reported as regulators of vascular cell migration and angiogenesis (58, 59, 60, 61). Each of these were increased by hypoxia (Tables 3 and 4). Additionally, lysyl oxidase (LOX), which was the most upregulated matrisome-associated protein under 1% O_2_ (Table 4), has been shown to be upregulated in hypoxic ECs and to stimulate angiogenesis *in vitro* and *in vivo* (62). Angiopoietin-like 4 (ANGPTL4), the second most upregulated matrisome-associated protein (Table 4), also regulates angiogenic activation through changes in EC metabolism (63), and its increased expression in response to chronic intermittent hypoxia has been proposed as a mechanistic link between obstructive sleep apnea and increased cardiovascular risk (64). Finally, the matrisome protein EGF-like repeats and discoidin I-like domains 3 (EDIL3; also known as Del-1) has pro-angiogenic properties (65), and was the second most upregulated core matrisome protein under 1% O_2_ (Table 3). ECs overexpressing EDIL3 exhibit increased expression of matrix metalloproteinase 9 (MMP9), plasminogen activator urokinase-type (PLAU), VEGFA, and ICAM1 and decreased expression of angiopoietin-2 (ANG2) (66). Apart from MMP9 (which was not detected here), this gene expression profile matches that of the hypoxic HCAECs examined here (Table S2), suggesting that hypoxia induces matrisome changes in ECs that support angiogenesis to restore physiological O_2_ levels.

In summary, this study presents novel data indicating that a VCAN-rich ECM is generated by HCAECs cultured under 1% O_2_. This ECM has an altered GAG profile with increased CS disaccharides, including C6S, which bind LDL avidly, providing mechanistic links between low O_2_ levels and atherogenesis, with these hypoxia-driven changes potentially contributing to increased LDL binding and retention within the artery wall. The data obtained, and particularly the increase in VCAN and other ECM proteins, correlate with a recently published plaque proteome database (36). Culture under 1% O_2_ also resulted in altered HCAEC behavior, with decreased adhesion, increased binding of exogenous HA, and increased proliferation and migration, all processes linked to hypoxia-driven angiogenic responses. Whether increased VCAN expression is a requirement for these processes remains to be established. Finally, in addition to VCAN, we have identified, using proteomic methods, many differentially abundant proteins in HCAECs cultured under 1% O_2_. A list of these differentially expressed species is provided in the Supporting Information.

## Experimental procedures

All chemicals were purchased from Sigma, unless otherwise stated in Table S3 and all solutions were prepared with Nano-pure H_2_O from a MilliQ system (Millipore). All absorbance and fluorescence readings in plates were performed with a Spectra Max i3x microplate reader from Molecular Devices.

### Cell culture

Primary human coronary artery endothelial cells (HCAECs) were isolated from normal coronary arteries by a commercial supplier (Cell Applications) who characterized the cells by Factor VIII-related antigen expression and DiI-Ac-LDL uptake. The donor (#2366) of the HCAECs was a 60-year-old Caucasian male and the cells had a doubling time of 25.6 h. HCAECs were thawed from frozen stocks and cultured (passage 3–6) in a humidified atmosphere with 5% CO_2_ and 37 °C using commercial MesoEndo growth medium (Cell Applications), with this changed every 2 to 3 days. For experiments, HCAECs were cultured for 24 h or 7 days at 20% or 1% O_2_, with hypoxia maintained using an InVivoO2 physiological cell culture workstation from Baker Ruskinn. To minimize dissolved O_2_ in the medium used for hypoxic culture, separate flasks of medium were used for culture at 20% and 1% O_2_, and the hypoxic medium was equilibrated at 1% O_2_ before use.

### Cell viability and proliferation

HCAECs were cultured for 1, 3, 5, and 7 days under 20% or 1% O_2_. Bromodeoxyuridine (BrdU) was added 24 h prior to analysis. At the appropriate time point, conditioned medium was removed and PrestoBlue (1:10) in growth medium was added to each well. After 1 h, the absorbance was read at 570 and 600 nm (absorbance at 600 nm was subtracted from 570 nm). The wells were washed once with PBS before detection of incorporated BrdU in cellular DNA using the BrdU Cell Proliferation ELISA Kit. Finally, absorbance was read at 450 and 550 nm (absorbance at 550 nm was subtracted from 450 nm).

### Immunocytochemistry

HCAECs were cultured for 24 h at 20% or 1% O_2_. CellROX Deep Red reagent (5 μM) was added for 30 min prior to fixation for investigation of ROS production, otherwise cells were fixed directly with 4% (v/v) formaldehyde in PBS for 15 min at 37 °C under the relevant O_2_ level. This was followed by permeabilization (0.3 M sucrose, 0.05 M NaCl, 6.3 mM MgCl2, 0.02 M HEPES, and 0.5% Triton X-100) on ice for 5 min and blocking with 1% (w/v) BSA in PBS for 1 h at 21 °C. Incubation with a mAb against HIF-1α (1:50) was carried out overnight at 4 °C. The next day, wells were washed twice with PBST (0.5% Tween-20), before incubation with Alexa-Fluor 488-goat anti-mouse secondary antibody (1:2000) for 1 h in the dark at 21 °C. Wells were washed twice with PBST, before counter staining in the dark for 30 min at 21 °C with Hoechst (8.1 μM in PBS; λ_ex/em_: 350/461 nm) for nuclei and Actin-Red 555 Ready Probes Reagent for F-actin fibers (λ_ex/em_: 540/565 nm) prepared as per the manufacturer’s instruction followed by 3 PBS washes. Cell treated with CellROX were only stained additionally with Hoechst. Cells were imaged using a fluorescence microscope (Olympus Life Science).

### Flow cytometry

HCAECs were cultured for 7 days under 20% or 1% O_2_, washed twice with PBS and incubated with CellROX Deep Red Reagent (5 μM) in growth medium for 30 min at 37 °C. Cells were washed three times with PBS and detached with trypsin. Cells were pelleted at 220*g* for 5 min at 4 °C before re-suspension in basal medium and kept on ice until analysis using an LSRFortessa cell analyser (Becton Dickinson Bioscience) to determine the percentage of CellROX Deep Red-positive cells. Staining with DAPI allowed separation between live and dead cells.

### Gene expression (quantitative PCR)

HCAECs were cultured for 7 days at 20% or 1% O_2_. RNA was extracted using the RNeasy Mini Kit with DNase I treatment. The concentration and purity of RNA was determined spectrophotometrically, and cDNA was synthesized from 600 ng RNA/reaction using the SensiFAST cDNA Synthesis Kit. Quantitative PCR was carried out using the QuantStudioTM 5 with the SensiFAST SYBR Hi-ROX Kit. The reaction mixture (10 μl) contained 4 μl cDNA (10 ng), 5 μl SYBR Hi-ROX and 1 μl primer (from a 10 μM stock). Actin-β and 18S were used as housekeeping genes for relative comparison of expression levels using the ΔΔCt-method. Primer sequences, where available, are presented in Table S4.

### Proteomics analysis by LC-MS/MS

HCAECs were cultured for 7 days at 20% or 1% O_2_. After 7 days, cells were lysed in 4% SDS with 10 mM tris(2-carbozyethyl)phosphine and 40 mM 2-chloroacetamide to reduce disulfides and subsequently alkylate thiols (70 °C, 20 min). Protein concentrations were determined by tryptophan fluorescence spectroscopy using λ_ex/em_: 295/350 nm, and an estimated frequency of tryptophan in human proteins of 1.3%. Aliquots of protein extracts corresponding to 20 μg of protein were transferred to fresh low-binding tubes and digested following an adapted PAC protocol (67). Briefly, 100 μg of magnetic beads were added to the samples, followed by acetonitrile (70% v/v). After mixing, the solution was incubated for 20 min at 21 °C. The tubes were then placed in a magnetic rack, allowing the beads to separate for 1 min before the supernatant was aspirated. The beads were then washed three times (without release from the magnet) with 100% acetonitrile and once with 70% (v/v) ethanol. Then the tubes were removed from the magnetic rack and the beads resuspended in 100 mM triethylammonium bicarbonate (pH 8.5) containing trypsin (0.013 μg μL^−1^), corresponding to a final 1:50 ratio of enzyme to protein. Trypsin digestion occurred overnight at 37 °C. The tubes were placed back in the magnetic rack and beads were allowed to separate for 1 min. The peptide samples were then acidified by adding TFA to 0.5% final concentration before desalting using self-packed StageTips. The eluates were dried down and stored at −80 °C until analysis by LC-MS/MS.

Before analysis, peptide digests were reconstituted in 0.1% trifluoroacetic acid to a concentration of 0.2 μg μL^−1^ and 1 μl peptide solution wase separated using a Dionex Ultimate RSLCnano system (Thermo Scientific) with an Aurora series reversed-phase C18 column (25 cm × 75 μm i.d., 1.6 μm C18, IonOpticks), maintained at 45 °C, coupled to a timsTOF Pro (Bruker) operated in data-independent acquisition with parallel accumulation and serial fragmentation (DIA-PASEF) mode. Samples were eluted using a gradient consisting of 2 to 30% mobile phase B over 100 min, followed by washing and re-equilibration of the column. Mobile phase A was 0.1% (v/v) formic acid in water, and B was 0.1 (v/v) formic acid in acetonitrile. In the DIA-PASEF mode, the mass spectrometer was operated in windows spanning 345 to 1201 *m/z* and 0.67 to 1.44 1/K0 [V s cm^−2^], which corresponds to an estimated cycle time of 1.16 s. DIA-PASEF windows and collision energy were also left to default with a base of 0.85 1/K0 [V s cm^−2^] set at 20 eV and 1.30 1/K0 [V s cm^−2^] set at 59 eV.

All spectral data were processed in DIA-NN (version 1.8.1) (68). Raw files were searched against an *in silico* predicted spectral library of tryptic peptides from all reviewed *Homo sapiens* sequences in the UniProtKB protein database (UP000005640, downloaded 2023–01–19) with Match-between-run (MBR) enabled. The precursor charge range was restricted to 2 to 3, and one missed cleavage was allowed. Mass accuracies were set to 15 ppm, and the scan window was set to 0 (automatic) for all analyses. Further data processing and analysis were then performed using the programming software R. First, the DIA-NN identifications were filtered at a 1% false discovery rate (FDR) threshold at both precursor and protein levels, then precursor intensities were log2-transformed, normalized, and summarized into protein expression values with robust model-based summarization (69). Peptides mapping to multiple proteins were summarized into groups of indistinguishable proteins, and the data was filtered for redundant protein groups and proteins only represented by a single peptide.

### Immunoblotting

HCAECs were cultured for 7 days at 20% or 1% O_2_. Cells were lysed with 6 M urea containing protease inhibitors overnight at 4 °C with rocking. Cell lysates were centrifuged at 220*g* at 4 °C for 5 min to pellet cell debris, and the supernatant was collected for protein precipitation with ammonium sulfate (226 g mL^−1^) overnight at 4 °C with rocking. The solution was then centrifuged at 4500*g* at 4 °C for 40 min to obtain protein pellets that were resuspended in PBS. A 21-gauge needle was used to break up DNA in cell lysate samples, followed by sonication for 1 to 2 min. The protein concentration was determined by BCA. Samples were treated with chondroitinase ABC (0.05 U mL^−1^) for 2 h at 37 °C and heated for 10 min at 70 °C prior to SDS-PAGE as previously described (70). 10 μg of protein was loaded into each well and immunoblotting was carried out as previously described (70) with an anti-versican G1 domain mAb (12C5, 1:1000).

## ELISA of decellularized ECM

HCAECs were cultured for 7 days at 20% or 1% O_2_ to generate ECM prior to decellularization with deoxycholate and detection of VCAN by ELISA with an anti-versican G1 domain mAb (12C5, 1:500) as described in (71).

### GAG analysis by fluorescence chromatography

HCAECs were cultured for 7 days at 20% or 1% O_2_, followed by cell lysis in a solution of 50 mM Tris pH 7.6, 10 mM CaCl_2_, 0.1% Triton, and 1 mg (w/v) Pronase. The mixture was vortexed briefly and incubated overnight at 37 °C with rotation. The next day, pronase was heat inactivated at 98 °C for 10 min and cooled down to 37 °C. Benzonase (250 U) and 2 mM MgCl_2_ was added to digest DNA and the samples were incubated for 4 h at 37 °C with rotation. The samples were then acidified to a pH of 4 to 5 with acetic acid, centrifuged at 20,000*g* for 20 min, filtered through 0.45-μm filters, and the GAGs were isolated on HiTrap DEAE FF columns (5 ml). The columns were equilibrated with 20 mM NaOAc and 0.4 M NaCl (pH 5) and the samples were eluted with 1.25 M NaCl. GAGs were precipitated by the addition of cold NaOAc-saturated 100% ethanol (3:1, v/v) and centrifuged at 20,000*g* for 20 min at 4 °C. The resulting pellets were dried, re-suspended in deionized water and further purified using Discovery BIO Wide Pore C5-5 and desalted on 1-mL HiTrap desalting columns. At this point, samples were split into two; one portion was digested with heparinases and the other with chondroitinase ABC. For the enzymatic degradation of heparan sulfate (HS), samples were incubated at 37 °C with 10 mU heparinase I for 2 h, followed by 10 mU heparinase III for 2 h, and finally 10 mU heparinase II overnight. The buffer for heparinase digestion was 50 mM sodium acetate and 5 mM calcium acetate (pH 6.5). For chondroitin sulfate (CS) digestion, samples were incubated with chondroitinase ABC (20 mU mL^−1^) overnight at 37 °C. The buffer for chondroitinase ABC digestion was 40 mM sodium acetate with 1 μM calcium chloride (pH 7). Digested HS and CS was lyophilized and disaccharide products were labelled with 2-Aminoacridone (AMAC) by re-suspending them in 10 μl of solution containing 0.1 M AMAC in 3:17 (v/v) acetic acid/dimethyl sulfoxide. The mixture was incubated at 21 °C for 15 min, followed by the addition of 10 μl of 1 M NaCNBH_3_, and was further incubated at 45 °C for 3 h. The product was again lyophilized and any excess AMAC was removed through two rounds of re-suspension in acetone, with subsequent pelleting by centrifugation at 20,000*g* for 20 min at 4 °C. Next, the samples were dissolved in 2% acetonitrile and subjected to analysis using Waters Acquity UPLC system equipped with a fluorescence detector and a BEH C18 column (2.1 × 150 mm, 1.7 μm; Waters), with fluorescence detected at 525 nm. A standard mix of AMAC-labeled disaccharides (20 pmol of each) was used for calibration.

### HA binding assay

HCAECs were cultured for 7 days at 20% or 1% O_2_ to generate ECM. The ECM was decellularized with deoxycholate as described previously (71). The wells were blocked with 0.1% (w/v) casein in PBS for 1 h at 21 °C followed by two washes with PBS and incubated overnight at 4 °C with three different concentrations of HA-FITC (50 kDa); 62.5, 125 and 500 μg mL^−1^. The next day, the wells were washed twice with PBS and filled with PBS for measurement of fluorescence from adherent HA (λ_ex/em_: 480/535 nm).

### Cell adhesion and proliferation assays

HCAECs were cultured for 7 days at 20% or 1% O_2_, with medium-only wells without cells as background controls. The ECM was decellularized with deoxycholate, blocked, and cells stained with calcein-AM were seeded on top as described in (71). After measuring the adhered cells by their fluorescence, the cells were re-incubated with growth medium for assessment of proliferation by MTS assay after 72 h as described in (71).

## Statistics and errors

Data are presented as mean ± SD. Statistical tests, including unpaired *t* test corrected with the Sidak-Holm method in case of multiple tests and one-way ANOVA corrected by Tukey’s *post hoc* test, were performed using Prism 8/9. To test for differentially expressed proteins from proteomic data, a protein-wise robust linear model with oxygen concentration included as a fixed factor (69). To account for potential batch effects, passage number and processing batch (plate ID) were included as random factors. A Bayes-moderated t-statistic was then calculated to give a *p*-value for each protein, and *p*-values were adjusted for multiple testing using the Benjamini-Hochberg procedure.

## Data availability

The mass spectrometry proteomics raw data files have been deposited in the ProteomeXchange (https://www.ebi.ac.uk/pride/archive) *via* the PRIDE partner repository with the dataset identifier PXD052078.

## Supporting information

This article contains supplementary data including supporting information Figs. S1–S4 and Tables S1–S10.

## Conflict of interest

The authors declare the following financial interests/personal relationships which may be considered as potential competing interests: M. J. D. declares consultancy contracts with Novo Nordisk A/S, and is a Director and major shareholder in the start-up company Seleno Therapeutics plc. These funders had no role in the design of the study; in the collection, analyses, or interpretation of data; in the writing of the manuscript, or in the decision to publish the results.

## References

[1] Medina-Leyte D.J., Zepeda-García O., Domínguez-Pérez M., González-Garrido A., Villarreal-Molina T., Jacobo-Albavera L. (2021). Endothelial dysfunction, inflammation and coronary artery disease: potential biomarkers and promising therapeutical approaches. Int. J. Mol. Sci..

[2] Lusis A.J. (2000). Atherosclerosis. Nature.

[3] Libby P., Buring J.E., Badimon L., Hansson G.K., Deanfield J., Bittencourt M.S. (2019). Atherosclerosis. Nat. Rev. Dis. Primers.

[4] Libby P., Ridker P.M., Maseri A. (2002). Inflammation and atherosclerosis. Circulation.

[5] Janaszak-Jasiecka A., Siekierzycka A., Płoska A., Dobrucki I.T., Kalinowski L. (2021). Endothelial dysfunction driven by hypoxia-the influence of oxygen deficiency on NO bioavailability. Biomolecules.

[6] Sluimer J.C., Gasc J.M., van Wanroij J.L., Kisters N., Groeneweg M., Sollewijn Gelpke M.D. (2008). Hypoxia, hypoxia-inducible transcription factor, and macrophages in human atherosclerotic plaques are correlated with intraplaque angiogenesis. J. Am. Coll. Cardiol..

[7] Weidemann A., Johnson R.S. (2008). Biology of HIF-1alpha. Cell Death Differ..

[8] Wagenseil J.E., Mecham R.P. (2009). Vascular extracellular matrix and arterial mechanics. Physiol. Rev..

[9] Wight T.N. (2018). A role for proteoglycans in vascular disease. Matrix Biol..

[10] Mohindra R., Agrawal D.K., Thankam F.G. (2021). Altered vascular extracellular matrix in the pathogenesis of atherosclerosis. J. Cardiovasc. Transl. Res..

[11] Gialeli C., Shami A., Goncalves I. (2021). Extracellular matrix: paving the way to the newest trends in atherosclerosis. Curr. Opin. Lipidol..

[12] Chait A., Wight T.N. (2000). Interaction of native and modified low-density lipoproteins with extracellular matrix. Curr. Opin. Lipidol..

[13] Nakashima Y., Fujii H., Sumiyoshi S., Wight T.N., Sueishi K. (2007). Early human atherosclerosis: accumulation of lipid and proteoglycans in intimal thickenings followed by macrophage infiltration. Arterioscler. Thromb. Vasc. Biol..

[14] Bennett M.R., Sinha S., Owens G.K. (2016). Vascular smooth muscle cells in atherosclerosis. Circ. Res..

[15] Adiguzel E., Ahmad P.J., Franco C., Bendeck M.P. (2009). Collagens in the progression and complications of atherosclerosis. Vasc. Med..

[16] Holm Nielsen S., Jonasson L., Kalogeropoulos K., Karsdal M.A., Reese-Petersen A.L., Auf dem Keller U. (2020). Exploring the role of extracellular matrix proteins to develop biomarkers of plaque vulnerability and outcome. J. Intern. Med..

[17] Fahed A.C., Jang I.K. (2021). Plaque erosion and acute coronary syndromes: phenotype, molecular characteristics and future directions. Nat. Rev. Cardiol..

[18] Myllyharju J., Schipani E. (2010). Extracellular matrix genes as hypoxia-inducible targets. Cell Tissue Res..

[19] Asplund A., Stillemark-Billton P., Larsson E., Rydberg E.K., Moses J., Hultén L.M. (2010). Hypoxic regulation of secreted proteoglycans in macrophages. Glycobiology.

[20] Chang Y.T., Chan C.K., Eriksson I., Johnson P.Y., Cao X., Westöö C. (2016). Versican accumulates in vascular lesions in pulmonary arterial hypertension. Pulm. Circ..

[21] Wang V., Davis D.A., Yarchoan R. (2017). Identification of functional hypoxia inducible factor response elements in the human lysyl oxidase gene promoter. Biochem. Biophys. Res. Commun..

[22] Hofbauer K.H., Gess B., Lohaus C., Meyer H.E., Katschinski D., Kurtz A. (2003). Oxygen tension regulates the expression of a group of procollagen hydroxylases. Eur. J. Biochem..

[23] Gilkes D.M., Bajpai S., Chaturvedi P., Wirtz D., Semenza G.L. (2013). Hypoxia-inducible factor 1 (HIF-1) promotes extracellular matrix remodeling under hypoxic conditions by inducing P4HA1, P4HA2, and PLOD2 expression in fibroblasts. J. Biol. Chem..

[24] Liu Y., Zhang H., Yan L., Du W., Zhang M., Chen H. (2018). MMP-2 and MMP-9 contribute to the angiogenic effect produced by hypoxia/15-HETE in pulmonary endothelial cells. J. Mol. Cell Cardiol..

[25] Lin J.L., Wang M.J., Lee D., Liang C.C., Lin S. (2008). Hypoxia-inducible factor-1alpha regulates matrix metalloproteinase-1 activity in human bone marrow-derived mesenchymal stem cells. FEBS Lett..

[26] Kim J.Y., Cathepsin L. (2021). A target of hypoxia-inducible factor-1-alpha, is involved in melanosome degradation in melanocytes. Int. J. Mol. Sci..

[27] Xiaofei C., Yanqing L., Dongkai Z., Dong C., Feng Z., Weilin W. (2018). Identification of cathepsin B as a novel target of hypoxia-inducible factor-1-alpha in HepG2 cells. Biochem. Biophys. Res. Commun..

[28] Bartoszewski R., Moszyńska A., Serocki M., Cabaj A., Polten A., Ochocka R. (2019). Primary endothelial cell-specific regulation of hypoxia-inducible factor (HIF)-1 and HIF-2 and their target gene expression profiles during hypoxia. FASEB J..

[29] Wong B.W., Marsch E., Treps L., Baes M., Carmeliet P. (2017). Endothelial cell metabolism in health and disease: impact of hypoxia. EMBO J..

[30] Hynes R.O., Naba A. (2012). Overview of the matrisome--an inventory of extracellular matrix constituents and functions. Cold Spring Harb. Perspect. Biol..

[31] Wight T.N., Merrilees M.J. (2004). Proteoglycans in atherosclerosis and restenosis: key roles for versican. Circ. Res..

[32] Wight T.N. (2002). Versican: a versatile extracellular matrix proteoglycan in cell biology. Curr. Opin. Cell Biol..

[33] Cattaruzza S., Schiappacassi M., Ljungberg-Rose A., Spessotto P., Perissinotto D., Mörgelin M. (2002). Distribution of PG-M/versican variants in human tissues and de novo expression of isoform V3 upon endothelial cell activation, migration, and neoangiogenesis in vitro. J. Biol. Chem..

[34] Mikami T., Kitagawa H. (2013). Biosynthesis and function of chondroitin sulfate. Biochim. Biophys. Acta.

[35] Yamagata M., Suzuki S., Akiyama S.K., Yamada K.M., Kimata K. (1989). Regulation of cell-substrate adhesion by proteoglycans immobilized on extracellular substrates. J. Biol. Chem..

[36] Lorentzen L.G., Yeung K., Eldrup N., Eiberg J.P., Sillesen H.H., Davies M.J. (2024). Proteomic analysis of the extracellular matrix of human atherosclerotic plaques shows marked changes between plaque types. Matrix Biol. Plus.

[37] Talusan P., Bedri S., Yang S., Kattapuram T., Silva N., Roughley P.J., Stone J.R. (2005). Analysis of intimal proteoglycans in atherosclerosis-prone and atherosclerosis-resistant human arteries by mass spectrometry. Mol. Cell Proteomics.

[38] Nakashima Y., Wight T.N., Sueishi K. (2008). Early atherosclerosis in humans: role of diffuse intimal thickening and extracellular matrix proteoglycans. Cardiovasc. Res..

[39] Mourao P.A., Pillai S., Di Ferrante N. (1981). The binding of chondroitin 6-sulfate to plasma low density lipoprotein. Biochim. Biophys. Acta.

[40] Asplund A., Fridén V., Stillemark-Billton P., Camejo G., Bondjers G. (2011). Macrophages exposed to hypoxia secrete proteoglycans for which LDL has higher affinity. Atherosclerosis.

[41] Sandy J.D., Westling J., Kenagy R.D., Iruela-Arispe M.L., Verscharen C., Rodriguez-Mazaneque J.C. (2001). Versican V1 proteolysis in human aorta in vivo occurs at the Glu441-Ala442 bond, a site that is cleaved by recombinant ADAMTS-1 and ADAMTS-4. J. Biol. Chem..

[42] Wight T.N., Kinsella M.G., Evanko S.P., Potter-Perigo S., Merrilees M.J. (2014). Versican and the regulation of cell phenotype in disease. Biochim. Biophys. Acta.

[43] Sheng W., Wang G., Wang Y., Liang J., Wen J., Zheng P.S. (2005). The roles of versican V1 and V2 isoforms in cell proliferation and apoptosis. Mol. Biol. Cell.

[44] Yamagata M., Saga S., Kato M., Bernfield M., Kimata K. (1993). Selective distributions of proteoglycans and their ligands in pericellular matrix of cultured fibroblasts. Implications for their roles in cell-substratum adhesion. J. Cell Sci..

[45] Serra M., Miquel L., Domenzain C., Docampo M.J., Fabra A., Wight T.N. (2005). V3 versican isoform expression alters the phenotype of melanoma cells and their tumorigenic potential. Int. J. Cancer.

[46] Lemire J.M., Merrilees M.J., Braun K.R., Wight T.N. (2002). Overexpression of the V3 variant of versican alters arterial smooth muscle cell adhesion, migration, and proliferation in vitro. J. Cell Physiol..

[47] Wight T.N. (2017). Provisional matrix: a role for versican and hyaluronan. Matrix Biol..

[48] Olsson A.K., Dimberg A., Kreuger J., Claesson-Welsh L. (2006). VEGF receptor signalling - in control of vascular function. Nat. Rev. Mol. Cell Biol..

[49] Papadas A., Arauz G., Cicala A., Wiesner J., Asimakopoulos F. (2020). Versican and versican-matrikines in cancer progression, inflammation, and immunity. J. Histochem. Cytochem..

[50] Tsuda T., Wang H., Timpl R., Chu M.L. (2001). Fibulin-2 expression marks transformed mesenchymal cells in developing cardiac valves, aortic arch vessels, and coronary vessels. Dev. Dyn..

[51] Strom A., Olin A.I., Aspberg A., Hultgardh-Nilsson A. (2006). Fibulin-2 is present in murine vascular lesions and is important for smooth muscle cell migration. Cardiovasc. Res..

[52] Guadall A., Orriols M., Rodríguez-Calvo R., Calvayrac O., Crespo J., Aledo R. (2011). Fibulin-5 is up-regulated by hypoxia in endothelial cells through a hypoxia-inducible factor-1 (HIF-1alpha)-dependent mechanism. J. Biol. Chem..

[53] Senger D.R., Davis G.E. (2011). Angiogenesis. Cold Spring Harb. Perspect. Biol..

[54] Sedding D.G., Boyle E.C., Demandt J.A.F., Sluimer J.C., Dutzmann J., Haverich A. (2018). Vasa vasorum angiogenesis: key player in the initiation and progression of atherosclerosis and potential target for the treatment of cardiovascular disease. Front. Immunol..

[55] Asano K., Nelson C.M., Nandadasa S., Aramaki-Hattori N., Lindner D.J., Alban T. (2017). Stromal versican regulates tumor growth by promoting angiogenesis. Sci. Rep..

[56] Yang W., Yee A.J. (2013). Versican V2 isoform enhances angiogenesis by regulating endothelial cell activities and fibronectin expression. FEBS Lett..

[57] Zheng P.S., Wen J., Ang L.C., Sheng W., Viloria-Petit A., Wang Y. (2004). Versican/PG-M G3 domain promotes tumor growth and angiogenesis. FASEB J..

[58] Kinsella M.G., Bressler S.L., Wight T.N. (2004). The regulated synthesis of versican, decorin, and biglycan: extracellular matrix proteoglycans that influence cellular phenotype. Crit. Rev. Eukaryot. Gene Expr..

[59] Yu L., Maishi N., Matsuda A., Hida K. (2023). The functional network of biglycan: a new frontier in tumor progression. Proteoglycan Res..

[60] Kinsella M.G., Tsoi C.K., Jarvelainen H.T., Wight T.N. (1997). Selective expression and processing of biglycan during migration of bovine aortic endothelial cells. The role of endogenous basic fibroblast growth factor. J. Biol. Chem..

[61] Wiberg C., Hedbom E., Khairullina A., Lamandé S.R., Oldberg A., Timpl R. (2001). Biglycan and decorin bind close to the n-terminal region of the collagen VI triple helix. J. Biol. Chem..

[62] Baker A.M., Bird D., Welti J.C., Gourlaouen M., Lang G., Murray G.I. (2013). Lysyl oxidase plays a critical role in endothelial cell stimulation to drive tumor angiogenesis. Cancer Res..

[63] Chaube B., Citrin K.M., Sahraei M., Singh A.K., de Urturi D.S., Ding W. (2023). Suppression of angiopoietin-like 4 reprograms endothelial cell metabolism and inhibits angiogenesis. Nat. Commun..

[64] Drager L.F., Yao Q., Hernandez K.L., Shin M.K., Bevans-Fonti S., Gay J. (2013). Chronic intermittent hypoxia induces atherosclerosis via activation of adipose angiopoietin-like 4. Am. J. Respir. Crit. Care Med..

[65] Niu X., Li X., Feng Z., Han Q., Li J., Liu Y. (2023). EDIL3 and VEGF synergistically affect angiogenesis in endothelial cells. Clin. Cosmet. Investig. Dermatol..

[66] Ciucurel E.C., Vlahos A.E., Sefton M.V. (2014). Using Del-1 to tip the angiogenic balance in endothelial cells in modular constructs. Tissue Eng. Part A..

[67] Batth T.S., Tollenaere M.X., Rüther P., Gonzalez-Franquesa A., Prabhakar B.S., Bekker-Jensen S. (2019). Protein aggregation capture on microparticles enables multipurpose proteomics sample preparation. Mol. Cell Proteomics.

[68] Demichev V., Messner C.B., Vernardis S.I., Lilley K.S., Ralser M. (2020). DIA-NN: neural networks and interference correction enable deep proteome coverage in high throughput. Nat. Methods.

[69] Sticker A., Goeminne L., Martens L., Clement L. (2020). Robust summarization and inference in proteome-wide label-free quantification. Mol. Cell Proteomics.

[70] Jorgensen S.M., Lorentzen L.G., Hammer A., Hoefler G., Malle E., Chuang C.Y. (2023). The inflammatory oxidant peroxynitrous acid modulates the structure and function of the recombinant human V3 isoform of the extracellular matrix proteoglycan versican. Redox Biol..

[71] Jorgensen S.M., Lorentzen L.G., Chuang C.Y., Davies M.J. (2022). Peroxynitrous acid-modified extracellular matrix alters gene and protein expression in human coronary artery smooth muscle cells and induces a pro-inflammatory phenotype. Free Radic. Biol. Med..

