# Supplementary material for: Hypoxia promotes the generation of a versican-rich extracellular matrix by human coronary artery endothelial cells
Source: J Biol Chem. 2025 Jul 5;301(8):110459. doi: 10.1016/j.jbc.2025.110459 (PMC12355077; doi:10.1016/j.jbc.2025.110459)
Supplement: Supporting information Figures S1-S4 [file mmc1.docx]

**Hypoxia promotes the generation of a versican-rich extracellular matrix by human coronary artery endothelial cells**

Sara M. Jørgensen^ab^, Song Huang^a^, Lasse G. Lorentzen^ab^, Fallen K.Y. Teoh^c^, Richard Karlsson^c^, John R. Harkness^a^, Rebecca L. Miller^c^, Michael J. Davies^a^, Christine Y. Chuang^a^*

^a^ Panum Institute, Department of Biomedical Sciences, University of Copenhagen, Copenhagen, Denmark

^b^ Department of Vascular Surgery, Rigshospitalet, Copenhagen, Denmark

^c^ Copenhagen Center for Glycomics, Department of Cellular and Molecular Medicine, University of Copenhagen, Copenhagen, Denmark

* Corresponding author. Department of Biomedical Sciences, Panum Institute, Blegdamsvej 3, University of Copenhagen, Copenhagen, 2200, Denmark.

*E-mail address:* [cchuang@sund.ku.dk](mailto:cchuang@sund.ku.dk) (CYC).

**Supporting Information – Figures S1-S4**

**Figure S1**. Original immunoblot detection of versican (anti-G1 domain).

**Figure S2**. Analysis of heparan sulfate (HS) disaccharide content in HCAECs O_2_ by fluorescence chromatography.

**Figure S3**. Analysis of chondroitin sulfate (CS) disaccharide distribution in HCAECs by fluorescence chromatography.

**Figure S4.** Hyaluronan (HA)-FITC standard curve and test fluorescence signal saturation.


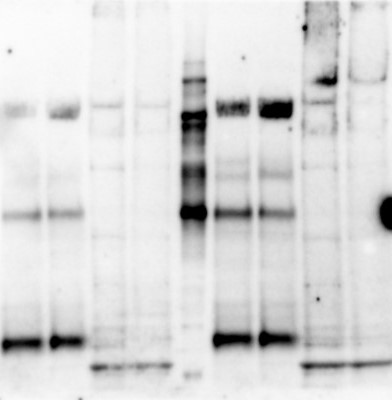
Lanes 1 2 3 4 5 6 7 8 9

**Figure S1.** Original immunoblot detection of versican (anti-G1 domain). Detection of versican in the total precipitated proteins from either HCAEC-derived conditioned medium or HCAECs (including ECM on culture plate) collected from two independent experiments (lanes 1-4, lanes 6-7) cultured for 7 days under 1% or 20% O_2_. Lanes 1,6: conditioned medium of HCAECs cultured at 1% O_2._ Lanes 2,7: conditioned medium of HCAECs cultured at 20% O_2_. Lanes 3, 8: ECM extract of HCAECs cultured at 1% O_2_. Lanes 3, 9: ECM extract of HCAECs cultured at 20% O_2_. Lane 5: protein molecular weight marker from top to bottom 460, 268, 238, 171, 117, 71, 55 and 41 kDa.


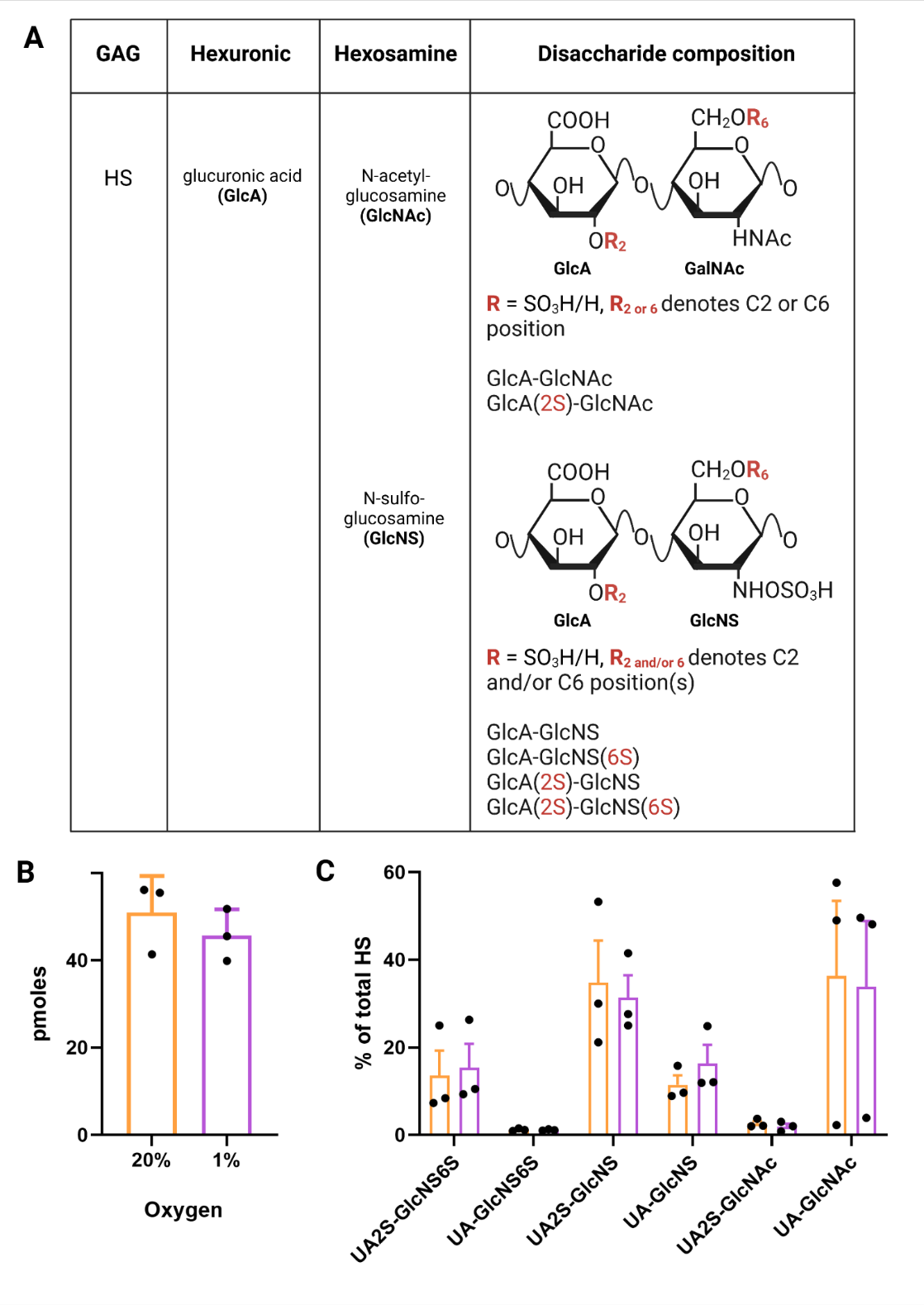


**Figure S2**. Analysis of heparan sulfate (HS) disaccharide content in HCAECs by fluorescence chromatography. HCAECs were cultured for 7 days under 1% or 20% O_2_. (**A**) Structure and nomenclature of monosaccharides that give rise to HS disaccharides where R denotes the position of sulfation (indicated in red). (**B**) Total HS disaccharide content from the HCAECs cultured under the indicated conditions, presented as mean picomoles ± SD and analyzed for statistical difference by an unpaired t-test. (**C**) Quantification of specific disaccharide species in HCAECs presented as mean percentage of the total HS content ± SD. No statistical differences were detected between the conditions using t-tests with p < 0.05 (*) taken as significant. Data from three independent experiments.


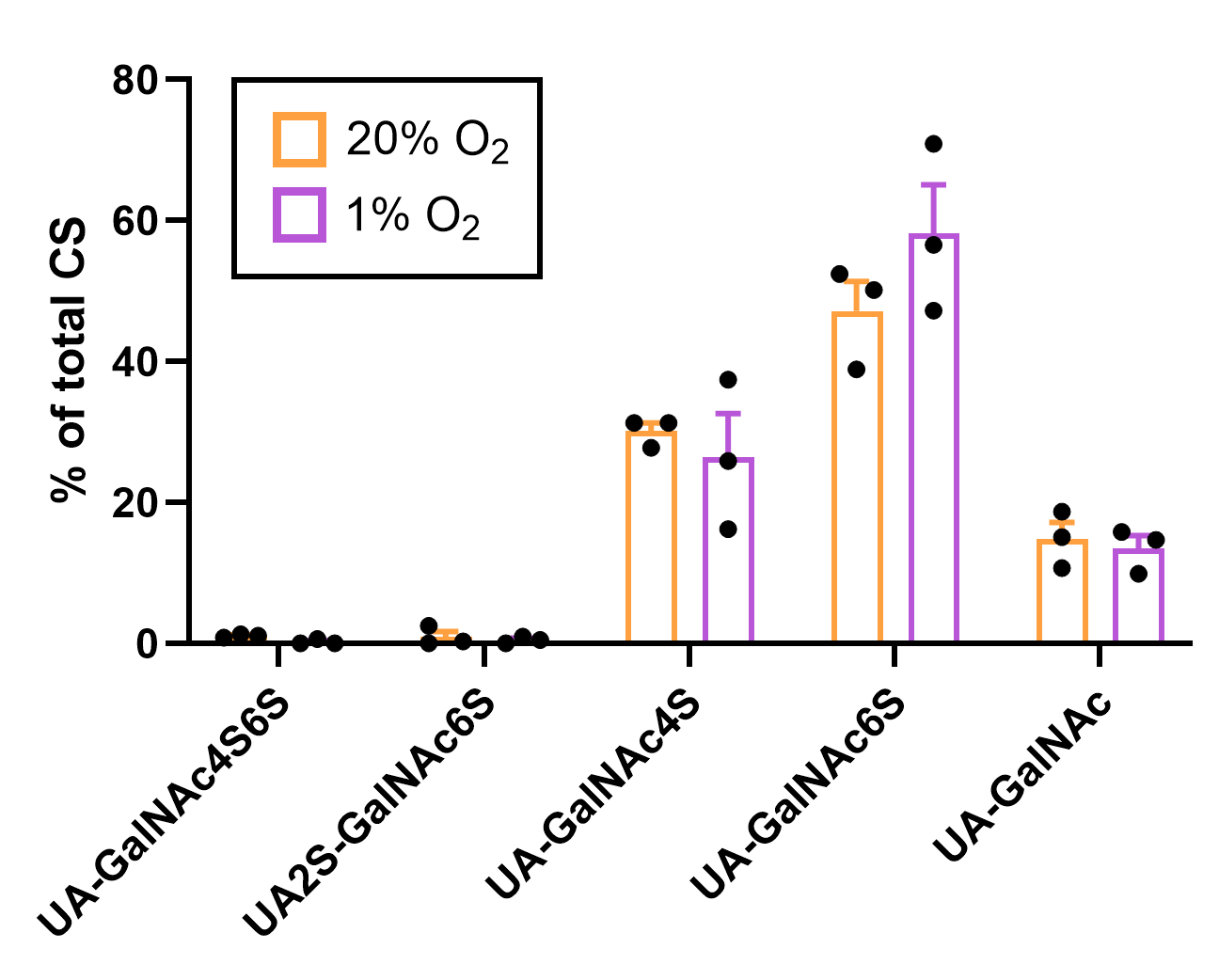


**Figure S3**. Analysis of chondroitin sulfate (CS) disaccharide distribution in HCAECs by fluorescence chromatography. HCAECs were cultured for 7 days under 1% or 20% O_2_. Detected disaccharide species are presented as the mean percentage of the total CS content ± SD from three independent experiments and analyzed for statistical differences by t-tests. No significant differences were detected with p < 0.05 (*) taken as significant. Data from three independent experiments.


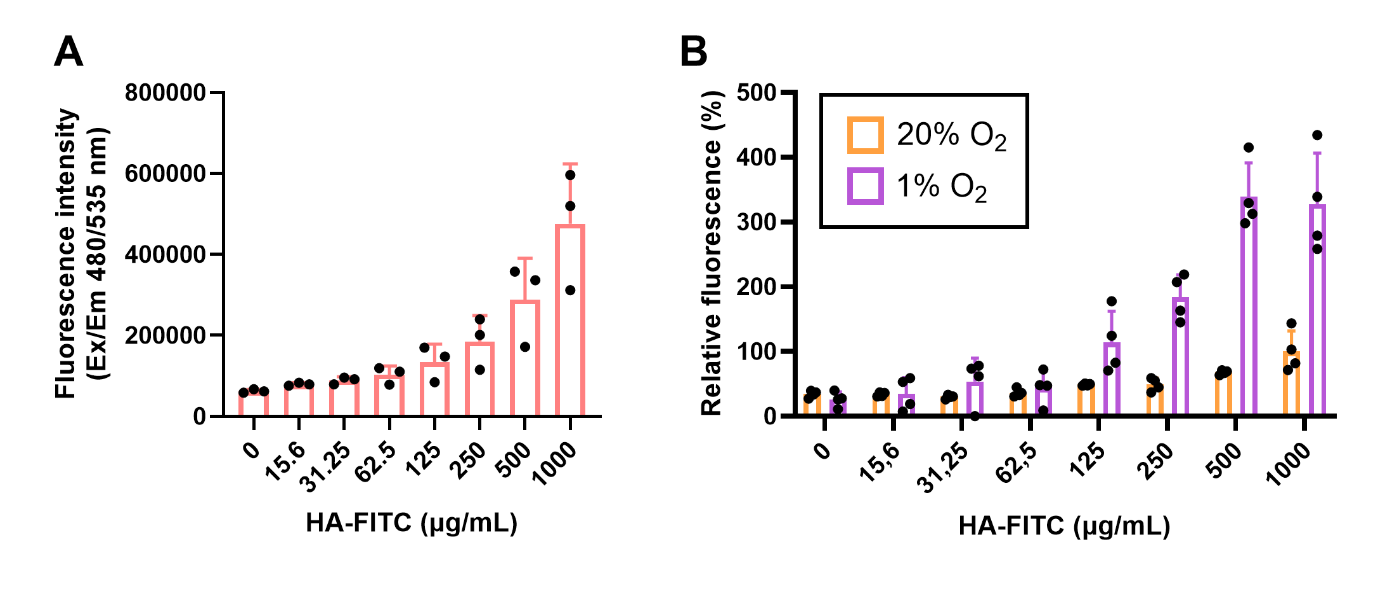


**Figure S4.** Hyaluronan (HA)-FITC standard curve and HA-FITC optimal concentration test. **(A)** Various concentrations of HA-FITC were incubated on plastic (black clear-bottom 96-well plates) for 2 h at 37^o^C. Data are presented as means ± SD of three replicate wells. **(B)** Increasing concentrations of HA-FITC used to detect optimal binding to HCAEC-ECM generated under 1% compared to 20% O_2_. Data are presented as means ± SD of three replicate wells.
