# Supplementary material for: Hypoxia promotes the generation of a versican-rich extracellular matrix by human coronary artery endothelial cells
Source: J Biol Chem. 2025 Jul 5;301(8):110459. doi: 10.1016/j.jbc.2025.110459 (PMC12355077; doi:10.1016/j.jbc.2025.110459)
Supplement: Supporting information tables S1-S10 [file mmc2.docx]

**Hypoxia promotes the generation of a versican-rich extracellular matrix by human coronary artery endothelial cells**

Sara M. Jørgensen^ab^, Song Huang^a^, Lasse G. Lorentzen^ab^, Fallen K.Y. Teoh^c^, Richard Karlsson^c^, John R. Harkness^a^, Rebecca L. Miller^c^, Michael J. Davies^a^, Christine Y. Chuang^a^*

^a^ Panum Institute, Department of Biomedical Sciences, University of Copenhagen, Copenhagen, Denmark

^b^ Department of Vascular Surgery, Rigshospitalet, Copenhagen, Denmark

^c^ Copenhagen Center for Glycomics, Department of Cellular and Molecular Medicine, University of Copenhagen, Copenhagen, Denmark

* Corresponding author. Department of Biomedical Sciences, Panum Institute, Blegdamsvej 3, University of Copenhagen, Copenhagen, 2200, Denmark.

*E-mail address:* [cchuang@sund.ku.dk](mailto:cchuang@sund.ku.dk) (CYC).

**Supporting Information – Tables S1-S10**

**Table S1**. IBAQ values for all detected proteins

**Table S2** Significantly differentially abundant proteins in hypoxic HCAECs compared to 20% O_2_

**Table S3**. Gene set enrichment analysis (biological process) for hypoxic HCAECs compared to 20% O_2_

**Table S4** Disease ontology enrichment analysis for hypoxic HCAECs compared to 20% O_2_

**Table S5**. Matrisome proteins detected in HCAECs

**Table S6**. IBAQ values for all detected core matrisome proteins

**Table S7**. IBAQ values for all detected matrisome-associated proteins

**Table S8**. Significantly differentially expressed matrisome proteins in hypoxic HCAECs compared to 20% O_2_ (p < 0.05)

**Table S9.** Materials and reagents

**Table S10**. Primer sequences used for quantitative PCR

**Table S1**. IBAQ values for all detected proteins

| **Rank** | **Gene Symbol** | **Uniprot** | **iBaq** |
| --- | --- | --- | --- |
| 1 | H2BU1 | Q8N257 | 14008177.26 |
| 2 | PFN1 | P07737 | 12504129.72 |
| 3 | VIM | P08670 | 12503579.23 |
| 4 | H4C1 | P62805 | 9836016.28 |
| 5 | PPIA | P62937 | 9806663.699 |
| 6 | GAPDH | P04406 | 9511921.998 |
| 7 | LGALS1 | P09382 | 8924194.704 |
| 8 | ANXA2 | P07355 | 8594592.322 |
| 9 | LDHA | P00338 | 6433155.514 |
| 10 | #N/A | P00761 | 5484880.818 |
| 11 | ENO1 | P06733 | 5313462.73 |
| 12 | TAGLN2 | P37802 | 5056506.382 |
| 13 | PTMA | P06454 | 4918158.66 |
| 14 | FKBP1A | P62942 | 4901514.503 |
| 15 | PDIA3 | P30101 | 4678630.895 |
| 16 | RPS25 | P62851 | 4652999.324 |
| 17 | PRDX1 | Q06830 | 4628381.525 |
| 18 | HSPA5 | P11021 | 4620567.727 |
| 19 | ANXA1 | P04083 | 4578138.229 |
| 20 | RPS9 | P46781 | 4257346.753 |
| 21 | RPL23A | P62750 | 4139645.527 |
| 22 | EEF1A2 | Q05639 | 3958761.082 |
| 23 | S100A6 | P06703 | 3894282.038 |
| 24 | PKM | P14618 | 3719877.675 |
| 25 | ANXA5 | P08758 | 3671297.091 |
| 26 | MYH9 | P35579 | 3649526.496 |
| 27 | LDHB | P07195 | 3606400.189 |
| 28 | PPIB | P23284 | 3524780.961 |
| 29 | RPS15A | P62244 | 3465731.325 |
| 30 | CAVIN1 | Q6NZI2 | 3458611.855 |
| 31 | YWHAZ | P63104 | 3364877.407 |
| 32 | RPL13A | P40429 | 3295895.986 |
| 33 | RAN | P62826 | 3282083.172 |
| 34 | RPL6 | Q02878 | 3259791.747 |
| 35 | RPS20 | P60866 | 3112508.78 |
| 36 | TXNDC5 | Q8NBS9 | 3058095.713 |
| 37 | HSP90B1 | P14625 | 3039942.282 |
| 38 | SERPINE1 | P05121 | 3006745.182 |
| 39 | RPS18 | P62269 | 2910493.935 |
| 40 | MSN | P26038 | 2850040.899 |
| 41 | ATP5F1A | P25705 | 2823151.149 |
| 42 | LMNA | P02545 | 2813228.403 |
| 43 | RPLP2 | P05387 | 2802466.595 |
| 44 | RPL18 | Q07020 | 2796286.617 |
| 45 | TMSB4X | P62328 | 2747836.278 |
| 46 | RPL7 | P18124 | 2718457.653 |
| 47 | TAGLN | Q01995 | 2658089.77 |
| 48 | H1-5 | P16401 | 2648096.95 |
| 49 | RPS6 | P62753 | 2597276.266 |
| 50 | TUBA1C | Q9BQE3 | 2564630.708 |
| 51 | HSP90AB1 | P08238 | 2552607.702 |
| 52 | RPL22 | P35268 | 2485510.795 |
| 53 | RPSA | P08865 | 2472442.282 |
| 54 | HSPE1 | P61604 | 2460109.882 |
| 55 | RPS8 | P62241 | 2450692.506 |
| 56 | RPL36 | Q9Y3U8 | 2438846.65 |
| 57 | GSTP1 | P09211 | 2432879.943 |
| 58 | ATP5F1B | P06576 | 2383557.273 |
| 59 | VAT1 | Q99536 | 2381716.605 |
| 60 | RPS3 | P23396 | 2351071.665 |
| 61 | TMSB10 | P63313 | 2348353.337 |
| 62 | RPS3A | P61247 | 2324876.136 |
| 63 | ARHGDIA | P52565 | 2323592.818 |
| 64 | H3C15 | Q71DI3 | 2305721.626 |
| 65 | TPI1 | P60174 | 2297002.479 |
| 66 | RPL13 | P26373 | 2278606.872 |
| 67 | RPS16 | P62249 | 2238016.991 |
| 68 | EEF2 | P13639 | 2204414.768 |
| 69 | P4HB | P07237 | 2192223.599 |
| 70 | FABP5 | Q01469 | 2164749.449 |
| 71 | TKT | P29401 | 2154084.591 |
| 72 | RPS13 | P62277 | 2150858.384 |
| 73 | RACK1 | P63244 | 2117582.389 |
| 74 | MYL6 | P60660 | 2110064.097 |
| 75 | RPL7A | P62424 | 2076144.937 |
| 76 | HSPA8 | P11142 | 2070745.141 |
| 77 | FAU | P62861 | 2033325.925 |
| 78 | TPM4 | P67936 | 2014079.377 |
| 79 | RPL27 | P61353 | 2001187.889 |
| 80 | RPL35A | P18077 | 1994373.621 |
| 81 | CTSD | P07339 | 1981642.706 |
| 82 | EEF1D | P29692 | 1919232.551 |
| 83 | HSPB1 | P04792 | 1908360.307 |
| 84 | RPS28 | P62857 | 1872070.671 |
| 85 | YWHAE | P62258 | 1866373.666 |
| 86 | ALDOA | P04075 | 1863017.293 |
| 87 | FN1 | P02751 | 1857983.844 |
| 88 | VDAC2 | P45880 | 1854542.904 |
| 89 | RPS14 | P62263 | 1850901.629 |
| 90 | VCP | P55072 | 1811994.208 |
| 91 | FLNA | P21333 | 1809627.784 |
| 92 | RPL10A | P62906 | 1797876.726 |
| 93 | CAP1 | Q01518 | 1781463.928 |
| 94 | ARPC4 | P59998 | 1753585.666 |
| 95 | RPL35 | P42766 | 1741475.557 |
| 96 | RPL4 | P36578 | 1730754.301 |
| 97 | WDR1 | O75083 | 1707359.057 |
| 98 | ARL6IP5 | O75915 | 1680650.873 |
| 99 | AHNAK | Q09666 | 1677101.11 |
| 100 | HNRNPA2B1 | P22626 | 1671446.512 |
| 101 | TALDO1 | P37837 | 1671379.279 |
| 102 | MYDGF | Q969H8 | 1651861.717 |
| 103 | RPL11 | P62913 | 1629080.634 |
| 104 | SH3BGRL3 | Q9H299 | 1628423.646 |
| 105 | PGK1 | P00558 | 1569500.066 |
| 106 | HBA1 | P69905 | 1553301.49 |
| 107 | H3-2 | Q5TEC6 | 1549906.153 |
| 108 | MIF | P14174 | 1516650.248 |
| 109 | RPL24 | P83731 | 1516549.506 |
| 110 | PDIA4 | P13667 | 1492075.846 |
| 111 | HNRNPK | P61978 | 1479892.473 |
| 112 | RPLP1 | P05386 | 1471362.426 |
| 113 | CKAP4 | Q07065 | 1455791.507 |
| 114 | CFL1 | P23528 | 1452164.588 |
| 115 | NCL | P19338 | 1445294.735 |
| 116 | CCT8 | P50990 | 1438037.117 |
| 117 | PDIA6 | Q15084 | 1436105.754 |
| 118 | ANXA6 | P08133 | 1400716.483 |
| 119 | RPL21 | P46778 | 1391799.679 |
| 120 | VDAC1 | P21796 | 1387635.207 |
| 121 | ACTR2 | P61160 | 1377700.488 |
| 122 | CLIC1 | O00299 | 1377665.682 |
| 123 | KRT7 | P08729 | 1374512.252 |
| 124 | RPL5 | P46777 | 1372339.848 |
| 125 | RPS11 | P62280 | 1369450.626 |
| 126 | TUBB6 | Q9BUF5 | 1366919.938 |
| 127 | EEF1B2 | P24534 | 1364734.307 |
| 128 | RPS19 | P39019 | 1362364.6 |
| 129 | UCHL1 | P09936 | 1353783.034 |
| 130 | RCN1 | Q15293 | 1346552.855 |
| 131 | HSPD1 | P10809 | 1335623.649 |
| 132 | TXN | P10599 | 1320868.78 |
| 133 | CAPNS1 | P04632 | 1305640.461 |
| 134 | FLNB | O75369 | 1299531.707 |
| 135 | RPL27A | P46776 | 1297056.006 |
| 136 | APRT | P07741 | 1286737.171 |
| 137 | RPL9 | P32969 | 1264328.058 |
| 138 | ATP5PO | P48047 | 1251299.284 |
| 139 | CDC37 | Q16543 | 1245244.183 |
| 140 | MDH2 | P40926 | 1226297.138 |
| 141 | ANXA3 | P12429 | 1225268.624 |
| 142 | RAB7A | P51149 | 1224322.56 |
| 143 | SERPINH1 | P50454 | 1221309.718 |
| 144 | GSTO1 | P78417 | 1218325.761 |
| 145 | CS | O75390 | 1211003.972 |
| 146 | PRDX2 | P32119 | 1205562.392 |
| 147 | NEDD8 | Q15843 | 1204091.796 |
| 148 | RPL12 | P30050 | 1171971.158 |
| 149 | RPL14 | P50914 | 1169308.207 |
| 150 | RPS2 | P15880 | 1165788.734 |
| 151 | PSMA7 | O14818 | 1149011.495 |
| 152 | PRKCSH | P14314 | 1137953.318 |
| 153 | ERP29 | P30040 | 1128676.207 |
| 154 | VCL | P18206 | 1125613.007 |
| 155 | EEF1G | P26641 | 1123279.636 |
| 156 | COPZ1 | P61923 | 1113753.615 |
| 157 | DAD1 | P61803 | 1110013.138 |
| 158 | HMGA1 | P17096 | 1109452.701 |
| 159 | RPL31 | P62899 | 1090057.849 |
| 160 | EIF5A | P63241 | 1089315.324 |
| 161 | PGAM1 | P18669 | 1088345.647 |
| 162 | TUBB | P07437 | 1072288.927 |
| 163 | ARPC3 | O15145 | 1070184.718 |
| 164 | RPL19 | P84098 | 1064268.385 |
| 165 | PFKP | Q01813 | 1061833.016 |
| 166 | PSMA1 | P25786 | 1054868.612 |
| 167 | ITGB1 | P05556 | 1049022 |
| 168 | SNCA | P37840 | 1035948.191 |
| 169 | LRRC59 | Q96AG4 | 1031758.413 |
| 170 | NQO1 | P15559 | 1020953.629 |
| 171 | ATP5MG | O75964 | 1014638.594 |
| 172 | ALB | P02769 | 1011909.561 |
| 173 | PRDX6 | P30041 | 1007463.261 |
| 174 | TUBB4B | P68371 | 995435.75 |
| 175 | TMEM109 | Q9BVC6 | 983771.5283 |
| 176 | PGD | P52209 | 979419.6329 |
| 177 | RPS7 | P62081 | 978516.0313 |
| 178 | DPYSL2 | Q16555 | 976538.854 |
| 179 | HNRNPU | Q00839 | 974917.311 |
| 180 | HNRNPA1 | P09651 | 974644.8745 |
| 181 | CAPZB | P47756 | 964619.3695 |
| 182 | TGM2 | P21980 | 963841.0686 |
| 183 | MVP | Q14764 | 963244.8992 |
| 184 | EIF6 | P56537 | 956304.1199 |
| 185 | PLOD2 | O00469 | 954752.8681 |
| 186 | NONO | Q15233 | 954522.3953 |
| 187 | DYNLRB1 | Q9NP97 | 954264.2636 |
| 188 | HSP90AA1 | P07900 | 940387.7579 |
| 189 | VDAC3 | Q9Y277 | 938141.5919 |
| 190 | CCT2 | P78371 | 936309.938 |
| 191 | TMEM123 | Q8N131 | 932153.15 |
| 192 | SERPINB9 | P50453 | 931158.9149 |
| 193 | CD9 | P21926 | 929753.3667 |
| 194 | CSTB | P04080 | 928192.7462 |
| 195 | MCAM | P43121 | 927317.6707 |
| 196 | ATP5ME | P56385 | 925657.347 |
| 197 | PRDX5 | P30044 | 925094.9892 |
| 198 | COX4I1 | P13073 | 915159.0813 |
| 199 | ACTR3 | P61158 | 913139.7885 |
| 200 | RBX1 | P62877 | 905124.786 |
| 201 | COTL1 | Q14019 | 902840.8673 |
| 202 | PSMA5 | P28066 | 900974.9096 |
| 203 | GNG12 | Q9UBI6 | 899603.7028 |
| 204 | AHCY | P23526 | 894658.5348 |
| 205 | RPS29 | P62273 | 888025.2 |
| 206 | H2AX | P16104 | 886921.5371 |
| 207 | RPN1 | P04843 | 885873.9969 |
| 208 | S100A10 | P60903 | 885446.7658 |
| 209 | HSPA9 | P38646 | 885116.1639 |
| 210 | RPL23 | P62829 | 884235.9201 |
| 211 | APMAP | Q9HDC9 | 883854.9996 |
| 212 | ZYX | Q15942 | 881998.2076 |
| 213 | LASP1 | Q14847 | 878110.329 |
| 214 | CCT3 | P49368 | 869355.6896 |
| 215 | KRT18 | P05783 | 863644.2944 |
| 216 | PHB | P35232 | 858053.4046 |
| 217 | TRAP1 | Q12931 | 857990.9118 |
| 218 | CALU | O43852 | 857983.6788 |
| 219 | PDLIM1 | O00151 | 855051.1694 |
| 220 | ILF3 | Q12906 | 851474.0766 |
| 221 | ACTN1 | P12814 | 851372.064 |
| 222 | GPI | P06744 | 851226.0207 |
| 223 | RPS10 | P46783 | 850919.6581 |
| 224 | TLN1 | Q9Y490 | 847684.9472 |
| 225 | ST13P5 | Q8NFI4 | 843378.9619 |
| 226 | S100A11 | P31949 | 840761.5492 |
| 227 | PNP | P00491 | 840425.3973 |
| 228 | NNMT | P40261 | 837195.9813 |
| 229 | CAV1 | Q03135 | 833495.8176 |
| 230 | GDI2 | P50395 | 832836.1444 |
| 231 | LSM3 | P62310 | 830968.1525 |
| 232 | STOM | P27105 | 829843.5051 |
| 233 | HADHB | P55084 | 829551.0624 |
| 234 | PARK7 | Q99497 | 825221.0452 |
| 235 | TCP1 | P17987 | 822278.2018 |
| 236 | HADHA | P40939 | 808095.9493 |
| 237 | PHPT1 | Q9NRX4 | 807640.3354 |
| 238 | DDOST | P39656 | 802113.1605 |
| 239 | HNRNPC | P07910 | 801888.4977 |
| 240 | EEF1A1 | P68104 | 800613.6676 |
| 241 | SLC25A3 | Q00325 | 798727.6726 |
| 242 | ACTN4 | O43707 | 798001.4894 |
| 243 | CCT4 | P50991 | 797020.7892 |
| 244 | PLS3 | P13797 | 796322.3296 |
| 245 | HNRNPM | P52272 | 790560.6613 |
| 246 | MYADM | Q96S97 | 779635.0958 |
| 247 | CAPZA1 | P52907 | 775507.9299 |
| 248 | TAX1BP3 | O14907 | 771718.928 |
| 249 | SSR1 | P43307 | 770189.3902 |
| 250 | SARNP | P82979 | 768482.2956 |
| 251 | HINT1 | P49773 | 768373.1901 |
| 252 | SURF4 | O15260 | 765883.9322 |
| 253 | PGLS | O95336 | 765316.1719 |
| 254 | SND1 | Q7KZF4 | 765171.1301 |
| 255 | CSRP1 | P21291 | 761769.1721 |
| 256 | GANAB | Q14697 | 761707.2049 |
| 257 | FSCN1 | Q16658 | 759474.4759 |
| 258 | NES | P48681 | 757926.7228 |
| 259 | AKR1B1 | P15121 | 757743.2359 |
| 260 | G6PD | P11413 | 756699.7548 |
| 261 | SH3BGRL | O75368 | 753579.05 |
| 262 | SOD1 | P00441 | 747411.616 |
| 263 | RPL10 | P27635 | 745358.8654 |
| 264 | TXNDC17 | Q9BRA2 | 745299.2653 |
| 265 | NUTF2 | P61970 | 743196.0704 |
| 266 | RPN2 | P04844 | 740213.7937 |
| 267 | CCT5 | P48643 | 736643.9503 |
| 268 | PHB2 | Q99623 | 734493.5265 |
| 269 | RPL17 | P18621 | 734166.0369 |
| 270 | PA2G4 | Q9UQ80 | 734047.6433 |
| 271 | SET | Q01105 | 729273.6854 |
| 272 | RPS24 | P62847 | 727958.413 |
| 273 | TPM3 | P06753 | 727766.9111 |
| 274 | PTMS | P20962 | 725995.6823 |
| 275 | ITGA5 | P08648 | 724338.1691 |
| 276 | IKBIP | Q70UQ0 | 724007.0805 |
| 277 | MAP4 | P27816 | 721166.1236 |
| 278 | SEC22B | O75396 | 720690.1717 |
| 279 | ANP32A | P39687 | 720122.2028 |
| 280 | XRCC6 | P12956 | 719418.7248 |
| 281 | MT-CO2 | P00403 | 718601.472 |
| 282 | SRSF3 | P84103 | 715136.1051 |
| 283 | S100A13 | Q99584 | 713491.855 |
| 284 | EIF4A1 | P60842 | 711167.0549 |
| 285 | KCTD12 | Q96CX2 | 710053.1626 |
| 286 | SNRPE | P62304 | 703811.654 |
| 287 | RPL32 | P62910 | 703536.3273 |
| 288 | PPP2R1A | P30153 | 702266.9945 |
| 289 | CALD1 | Q05682 | 701585.3956 |
| 290 | EHD4 | Q9H223 | 701578.5947 |
| 291 | SRSF1 | Q07955 | 699638.4738 |
| 292 | MACROH2A1 | O75367 | 699323.0026 |
| 293 | YWHAB | P31946 | 693280.0014 |
| 294 | SSR4 | P51571 | 692833.7241 |
| 295 | PEBP1 | P30086 | 692654.6001 |
| 296 | ATP5MF | P56134 | 691937.0803 |
| 297 | TMED10 | P49755 | 688353.5814 |
| 298 | GLO1 | Q04760 | 687522.4834 |
| 299 | RPL29 | P47914 | 683664.4473 |
| 300 | RPS21 | P63220 | 683482.5498 |
| 301 | GOT2 | P00505 | 683420.7371 |
| 302 | ACAT1 | P24752 | 680337.0854 |
| 303 | NAP1L1 | P55209 | 678926.8994 |
| 304 | UBA1 | P22314 | 677313.009 |
| 305 | PTBP1 | P26599 | 677234.6289 |
| 306 | TMA7 | Q9Y2S6 | 675715.563 |
| 307 | ISG15 | P05161 | 670883.9887 |
| 308 | PECAM1 | P16284 | 670455.0525 |
| 309 | PLEC | Q15149 | 667928.7942 |
| 310 | RPS27L | Q71UM5 | 661336.5 |
| 311 | SUB1 | P53999 | 660343.3513 |
| 312 | ATP1A1 | P05023 | 659423.5725 |
| 313 | STIP1 | P31948 | 656710.7435 |
| 314 | PSMB1 | P20618 | 656613.1003 |
| 315 | RPL8 | P62917 | 655399.9066 |
| 316 | ARPC2 | O15144 | 653363.2042 |
| 317 | BOLA2 | Q9H3K6 | 651005.4373 |
| 318 | ELAVL1 | Q15717 | 649470.865 |
| 319 | PDLIM5 | Q96HC4 | 648963.1391 |
| 320 | PSMD11 | O00231 | 648330.032 |
| 321 | TMEM258 | P61165 | 646109.1965 |
| 322 | IQGAP1 | P46940 | 644151.8236 |
| 323 | NPM1 | P06748 | 643367.4615 |
| 324 | PSMB2 | P49721 | 636332.5675 |
| 325 | CCT7 | Q99832 | 635098.0705 |
| 326 | CANX | P27824 | 634006.657 |
| 327 | PRDX4 | Q13162 | 629791.6934 |
| 328 | MDH1 | P40925 | 628149.3283 |
| 329 | PSMB7 | Q99436 | 627779.7963 |
| 330 | PDLIM7 | Q9NR12 | 627128.5966 |
| 331 | PAICS | P22234 | 626371.6091 |
| 332 | RPS4X | P62701 | 626350.3633 |
| 333 | PCBP2 | Q15366 | 626346.4096 |
| 334 | SNRPD2 | P62316 | 625283.9734 |
| 335 | PSMA6 | P60900 | 625056.7275 |
| 336 | PDCD6IP | Q8WUM4 | 624919.4654 |
| 337 | ATP5F1C | P36542 | 623007.1434 |
| 338 | PSMA3 | P25788 | 619448.1073 |
| 339 | MAPRE1 | Q15691 | 618092.2717 |
| 340 | EIF4H | Q15056 | 617750.1356 |
| 341 | SSBP1 | Q04837 | 616576.0688 |
| 342 | TBCA | O75347 | 614515.7582 |
| 343 | PDAP1 | Q13442 | 613702.2516 |
| 344 | PSMC2 | P35998 | 612881.1552 |
| 345 | GNAI2 | P04899 | 611837.7793 |
| 346 | SFPQ | P23246 | 609400.6764 |
| 347 | BCAP31 | P51572 | 608750.3463 |
| 348 | CNN3 | Q15417 | 607011.2742 |
| 349 | PRXL2A | Q9BRX8 | 603696.1175 |
| 350 | RPLP0 | P05388 | 602166.854 |
| 351 | NAMPT | P43490 | 601487.6315 |
| 352 | ACOT7 | O00154 | 594366.2313 |
| 353 | ARF4 | P18085 | 593385.2353 |
| 354 | ACTA1 | P68133 | 593328.7201 |
| 355 | CDC42 | P60953 | 590752.819 |
| 356 | PSMB3 | P49720 | 590116.5574 |
| 357 | TPD52L2 | O43399 | 589678.831 |
| 358 | RPS5 | P46782 | 586554.9693 |
| 359 | NUDC | Q9Y266 | 586213.375 |
| 360 | CYB5R3 | P00387 | 584435.7662 |
| 361 | TPM1 | P09493 | 580753.7113 |
| 362 | CALR | P27797 | 577920.7556 |
| 363 | ELOB | Q15370 | 575884.0083 |
| 364 | CCT6A | P40227 | 575497.2752 |
| 365 | SNRPD3 | P62318 | 575121.8782 |
| 366 | CTSB | P07858 | 572339.9531 |
| 367 | DPYSL3 | Q14195 | 570999.3622 |
| 368 | PSMA2 | P25787 | 570093.6142 |
| 369 | SEPTIN2 | Q15019 | 569179.1873 |
| 370 | CYCS | P99999 | 563892.2568 |
| 371 | RPS26 | P62854 | 562596.1934 |
| 372 | SERPINB6 | P35237 | 562541.3712 |
| 373 | UBE2I | P63279 | 561615.641 |
| 374 | FDPS | P14324 | 560667.7492 |
| 375 | SRI | P30626 | 560544.411 |
| 376 | PFDN4 | Q9NQP4 | 559923.3983 |
| 377 | RPL15 | P61313 | 558021.6136 |
| 378 | RSU1 | Q15404 | 557597.5787 |
| 379 | TUFM | P49411 | 555245.2912 |
| 380 | ELOC | Q15369 | 554044.5536 |
| 381 | XRCC5 | P13010 | 553851.7095 |
| 382 | UBE2N | P61088 | 551528.0245 |
| 383 | PSMC1 | P62191 | 550727.819 |
| 384 | CBR1 | P16152 | 549432.6049 |
| 385 | PCBP1 | Q15365 | 547982.9664 |
| 386 | SLC25A5 | P05141 | 546963.5988 |
| 387 | CLIC4 | Q9Y696 | 546026.6263 |
| 388 | RPL3 | P39023 | 545995.1134 |
| 389 | ARCN1 | P48444 | 544896.7738 |
| 390 | DYNC1H1 | Q14204 | 544183.2137 |
| 391 | ETFB | P38117 | 542221.5597 |
| 392 | RPL30 | P62888 | 541727.595 |
| 393 | TXNRD1 | Q16881 | 541192.2621 |
| 394 | RUVBL1 | Q9Y265 | 539620.6408 |
| 395 | SNRPD1 | P62314 | 538643.4663 |
| 396 | ILF2 | Q12905 | 538463.1388 |
| 397 | BAX | Q07812 | 536644.6214 |
| 398 | HMGB3 | O15347 | 535699.0357 |
| 399 | RPS17 | P08708 | 535159.9268 |
| 400 | CAPZA2 | P47755 | 534923.7285 |
| 401 | DCTN2 | Q13561 | 533817.7717 |
| 402 | RAD23B | P54727 | 531096.1995 |
| 403 | EHD2 | Q9NZN4 | 528305.963 |
| 404 | TMX1 | Q9H3N1 | 526666.9717 |
| 405 | GPX1 | P07203 | 524852.2159 |
| 406 | CAVIN2 | O95810 | 521436.5406 |
| 407 | HSD17B12 | Q53GQ0 | 521287.7323 |
| 408 | CRIP2 | P52943 | 518908.4323 |
| 409 | PSMB4 | P28070 | 518564.2701 |
| 410 | RNH1 | P13489 | 518441.7158 |
| 411 | PLIN3 | O60664 | 516660.3935 |
| 412 | SDCBP | O00560 | 516288.583 |
| 413 | KPNB1 | Q14974 | 514635.8969 |
| 414 | MGST2 | Q99735 | 512590.5755 |
| 415 | CDV3 | Q9UKY7 | 508714.6311 |
| 416 | TBCC | Q15814 | 508699.8868 |
| 417 | ANPEP | P15144 | 506620.9749 |
| 418 | CLTC | Q00610 | 506074.8538 |
| 419 | PSMA4 | P25789 | 504279.1916 |
| 420 | ACTG1 | P63261 | 498987.3762 |
| 421 | RALA | P11233 | 498758.1533 |
| 422 | SRSF7 | Q16629 | 498296.3211 |
| 423 | ADH5 | P11766 | 497680.1828 |
| 424 | SRM | P19623 | 494852.181 |
| 425 | FKBP3 | Q00688 | 494477.0738 |
| 426 | TPT1 | P13693 | 493566.8855 |
| 427 | MYL1 | P05976 | 490491.5267 |
| 428 | DNAJB4 | Q9UDY4 | 487313.8999 |
| 429 | STRAP | Q9Y3F4 | 486151.5768 |
| 430 | H2AC18 | Q6FI13 | 485838.8032 |
| 431 | HYOU1 | Q9Y4L1 | 483119.6024 |
| 432 | KRT1 | P04264 | 482306.5856 |
| 433 | PPA1 | Q15181 | 481999.9414 |
| 434 | ATP5PD | O75947 | 480875.3299 |
| 435 | RPS23 | P62266 | 476612.9043 |
| 436 | RAB18 | Q9NP72 | 476607.8893 |
| 437 | EIF3F | O00303 | 476536.0207 |
| 438 | PSMB5 | P28074 | 476043.3368 |
| 439 | ATP5F1D | P30049 | 474766.7935 |
| 440 | STMN1 | P16949 | 474689.0478 |
| 441 | CAVIN3 | Q969G5 | 474276.3761 |
| 442 | PSAP | P07602 | 473729.3749 |
| 443 | MAP1LC3A | Q9H492 | 471941.2667 |
| 444 | CTNNA1 | P35221 | 470604.0033 |
| 445 | LMNB1 | P20700 | 470278.8343 |
| 446 | NPEPPS | P55786 | 469056.7086 |
| 447 | SNX3 | O60493 | 467621.8105 |
| 448 | CTTN | Q14247 | 466813.5768 |
| 449 | PCNA | P12004 | 466098.4171 |
| 450 | HNRNPH1 | P31943 | 466044.8363 |
| 451 | RPL37A | P61513 | 465021.2408 |
| 452 | SERBP1 | Q8NC51 | 464977.4987 |
| 453 | RDX | P35241 | 464804.4612 |
| 454 | SBDS | Q9Y3A5 | 464280.0684 |
| 455 | HDLBP | Q00341 | 459547.9693 |
| 456 | PSMC3 | P17980 | 459296.219 |
| 457 | FERMT3 | Q86UX7 | 458757.3103 |
| 458 | ETFA | P13804 | 458315.7792 |
| 459 | ARHGDIB | P52566 | 457414.3556 |
| 460 | ALDH2 | P05091 | 457229.429 |
| 461 | PLP2 | Q04941 | 455805.94 |
| 462 | THBS1 | P07996 | 455565.6106 |
| 463 | YWHAH | Q04917 | 454189.2112 |
| 464 | NUDT5 | Q9UKK9 | 453356.3821 |
| 465 | HK1 | P19367 | 453276.1236 |
| 466 | ACLY | P53396 | 452161.7163 |
| 467 | LAMP1 | P11279 | 451744.107 |
| 468 | PFDN2 | Q9UHV9 | 451343.7047 |
| 469 | GARS1 | P41250 | 449753.3726 |
| 470 | COPA | P53621 | 449510.7043 |
| 471 | PSMD14 | O00487 | 448258.5166 |
| 472 | ADSS2 | P30520 | 447191.8574 |
| 473 | GSN | P06396 | 446860.1952 |
| 474 | EIF4A3 | P38919 | 445863.5946 |
| 475 | RPL28 | P46779 | 445722.846 |
| 476 | RTCB | Q9Y3I0 | 444229.3693 |
| 477 | COX5B | P10606 | 443114.5365 |
| 478 | HNRNPA3 | P51991 | 441227.8727 |
| 479 | YWHAQ | P27348 | 440375.6383 |
| 480 | LMNB2 | Q03252 | 438993.0003 |
| 481 | DBN1 | Q16643 | 437927.4644 |
| 482 | EIF3E | P60228 | 437801.0629 |
| 483 | REXO2 | Q9Y3B8 | 437400.1546 |
| 484 | PSMD3 | O43242 | 436472.4395 |
| 485 | RUVBL2 | Q9Y230 | 436213.5453 |
| 486 | SHMT2 | P34897 | 435916.6599 |
| 487 | SUMO1 | P63165 | 435848.0248 |
| 488 | ARPC5 | O15511 | 434402.6239 |
| 489 | BZW1 | Q7L1Q6 | 433820.0267 |
| 490 | ACOT9 | Q9Y305 | 433501.7354 |
| 491 | HNRNPD | Q14103 | 432810.7782 |
| 492 | RALY | Q9UKM9 | 432306.8094 |
| 493 | CMPK1 | P30085 | 429943.4985 |
| 494 | IMPDH2 | P12268 | 428196.3261 |
| 495 | RPL38 | P63173 | 427615.1977 |
| 496 | RAC1 | P63000 | 427215.7364 |
| 497 | SRP9 | P49458 | 423068.3843 |
| 498 | G3BP1 | Q13283 | 420859.9681 |
| 499 | RANBP1 | P43487 | 420369.455 |
| 500 | IDS | P22304 | 419562.7143 |
| 501 | SNAP23 | O00161 | 417608.1438 |
| 502 | FBL | P22087 | 416978.8443 |
| 503 | MYOF | Q9NZM1 | 416316.6556 |
| 504 | BUB3 | O43684 | 415724.097 |
| 505 | SSR3 | Q9UNL2 | 414048.0655 |
| 506 | SCP2 | P22307 | 413881.096 |
| 507 | ANXA7 | P20073 | 413498.2699 |
| 508 | ANXA4 | P09525 | 413076.8078 |
| 509 | COPB1 | P53618 | 412331.7785 |
| 510 | DSTN | P60981 | 411584.8502 |
| 511 | DLST | P36957 | 411488.6199 |
| 512 | PSMD4 | P55036 | 411441.0765 |
| 513 | YWHAG | P61981 | 410483.615 |
| 514 | POSTN | Q15063 | 409655.7852 |
| 515 | SNRNP70 | P08621 | 408370.0752 |
| 516 | FH | P07954 | 407596.8269 |
| 517 | LSM5 | Q9Y4Y9 | 406486.6563 |
| 518 | PSME1 | Q06323 | 406246.8792 |
| 519 | CD59 | P13987 | 405221.5295 |
| 520 | CNDP2 | Q96KP4 | 405009.6517 |
| 521 | GLOD4 | Q9HC38 | 404962.6455 |
| 522 | RAB5C | P51148 | 404642.9246 |
| 523 | DCTN3 | O75935 | 403791.7911 |
| 524 | DARS1 | P14868 | 403244.7271 |
| 525 | RTN4 | Q9NQC3 | 403150.5619 |
| 526 | ARPC1B | O15143 | 402528.4706 |
| 527 | MMP14 | P50281 | 400699.4906 |
| 528 | AKAP12 | Q02952 | 400625.5446 |
| 529 | EIF2S1 | P05198 | 399962.993 |
| 530 | WARS1 | P23381 | 398949.4096 |
| 531 | HNRNPF | P52597 | 398643.0695 |
| 532 | PGRMC2 | O15173 | 397078.0479 |
| 533 | PSMC4 | P43686 | 396151.4696 |
| 534 | RRBP1 | Q9P2E9 | 392440.6936 |
| 535 | PRDX3 | P30048 | 390448.4016 |
| 536 | ILK | Q13418 | 390196.023 |
| 537 | NAGK | Q9UJ70 | 389637.7376 |
| 538 | FBLIM1 | Q8WUP2 | 389139.8886 |
| 539 | SYNCRIP | O60506 | 388680.8707 |
| 540 | UFM1 | P61960 | 388625.1523 |
| 541 | OAT | P04181 | 388227.1757 |
| 542 | ANXA11 | P50995 | 385529.2078 |
| 543 | SEPTIN7 | Q16181 | 384391.3422 |
| 544 | RPL34 | P49207 | 382910.5483 |
| 545 | GBE1 | Q04446 | 378368.2214 |
| 546 | HSD17B10 | Q99714 | 378084.5668 |
| 547 | SEC31A | O94979 | 377061.6651 |
| 548 | HDGF | P51858 | 376657.7032 |
| 549 | CAPRIN1 | Q14444 | 374202.7412 |
| 550 | PSMD8 | P48556 | 373490.8326 |
| 551 | FKBP2 | P26885 | 372314.589 |
| 552 | PRAF2 | O60831 | 370831.2575 |
| 553 | YARS1 | P54577 | 370334.9989 |
| 554 | AK3 | Q9UIJ7 | 370032.5551 |
| 555 | UBE2L3 | P68036 | 367843.8274 |
| 556 | PSMD12 | O00232 | 367497.2964 |
| 557 | PFDN1 | O60925 | 367041.9063 |
| 558 | SSB | P05455 | 365903.0754 |
| 559 | SNCG | O76070 | 364838.9982 |
| 560 | PSMC5 | P62195 | 364514.4758 |
| 561 | EFHD2 | Q96C19 | 364504.6895 |
| 562 | TMEM43 | Q9BTV4 | 363513.8224 |
| 563 | SULT1B1 | O43704 | 363488.9532 |
| 564 | MYO1C | O00159 | 360486.5731 |
| 565 | FKBP10 | Q96AY3 | 360231.2897 |
| 566 | STX12 | Q86Y82 | 359849.8723 |
| 567 | HEXB | P07686 | 359822.5473 |
| 568 | H1-2 | P16403 | 359443.1307 |
| 569 | MAP1B | P46821 | 359202.7268 |
| 570 | HSPA4 | P34932 | 359084.2112 |
| 571 | SEC61B | P60468 | 358903.7677 |
| 572 | CORO1C | Q9ULV4 | 358759.034 |
| 573 | PSMD7 | P51665 | 358395.8257 |
| 574 | MGP | P08493 | 357836.118 |
| 575 | HSPG2 | P98160 | 355357.2254 |
| 576 | BCAT1 | P54687 | 354585.9503 |
| 577 | SNRPA1 | P09661 | 354000.7459 |
| 578 | RHEB | Q15382 | 352924.3012 |
| 579 | CNN2 | Q99439 | 352621.2231 |
| 580 | ERP44 | Q9BS26 | 352546.5178 |
| 581 | CAPN1 | P07384 | 352024.1285 |
| 582 | SH3GLB1 | Q9Y371 | 350987.8488 |
| 583 | PAK2 | Q13177 | 350884.8768 |
| 584 | COPE | O14579 | 350437.4851 |
| 585 | GLRX3 | O76003 | 349166.8841 |
| 586 | NIBAN2 | Q96TA1 | 348956.579 |
| 587 | FLNC | Q14315 | 348349.6711 |
| 588 | TXNL1 | O43396 | 348072.778 |
| 589 | RAB15 | P59190 | 347906.2467 |
| 590 | FASN | P49327 | 347211.5934 |
| 591 | BTF3 | P20290 | 346549.5785 |
| 592 | VASP | P50552 | 345810.4262 |
| 593 | UGP2 | Q16851 | 344889.0756 |
| 594 | CPNE1 | Q99829 | 344200.213 |
| 595 | CFL2 | Q9Y281 | 344030.1778 |
| 596 | ESYT1 | Q9BSJ8 | 343526.4314 |
| 597 | NAPA | P54920 | 343078.7334 |
| 598 | SH3GL1 | Q99961 | 342960.8081 |
| 599 | ERO1A | Q96HE7 | 342375.5684 |
| 600 | PSME3 | P61289 | 341666.8149 |
| 601 | KHSRP | Q92945 | 340897.5232 |
| 602 | COPB2 | P35606 | 339629.5729 |
| 603 | VPS26A | O75436 | 338527.6421 |
| 604 | NIFK | Q9BYG3 | 338402.6491 |
| 605 | DHX15 | O43143 | 338303.0869 |
| 606 | CTSZ | Q9UBR2 | 338046.3523 |
| 607 | CAPN2 | P17655 | 337459.4221 |
| 608 | RARS1 | P54136 | 337274.5503 |
| 609 | CNPY2 | Q9Y2B0 | 336507.6727 |
| 610 | PAPSS2 | O95340 | 335576.3307 |
| 611 | LGALS3 | P17931 | 334885.5952 |
| 612 | GSTM3 | P21266 | 334820.9904 |
| 613 | BSG | P35613 | 334669.6916 |
| 614 | ATP6V1E1 | P36543 | 334206.0585 |
| 615 | LSM6 | P62312 | 333977.449 |
| 616 | ACADVL | P49748 | 333394.6166 |
| 617 | TMEM263 | Q8WUH6 | 332923.5078 |
| 618 | ATIC | P31939 | 332680.3346 |
| 619 | OTUB1 | Q96FW1 | 331211.9371 |
| 620 | EHD1 | Q9H4M9 | 331200.3453 |
| 621 | PSMD13 | Q9UNM6 | 331097.6472 |
| 622 | MTPN | P58546 | 330421.3287 |
| 623 | EIF4E | P06730 | 329781.46 |
| 624 | SEC61A1 | P61619 | 328702.9894 |
| 625 | MARCKS | P29966 | 327836.0101 |
| 626 | ACP1 | P24666 | 327175.9496 |
| 627 | DHX9 | Q08211 | 327117.2238 |
| 628 | VPS29 | Q9UBQ0 | 326670.4066 |
| 629 | RBM14 | Q96PK6 | 326240.0641 |
| 630 | ATP2A2 | P16615 | 326090.3335 |
| 631 | ARPC5L | Q9BPX5 | 325984.0444 |
| 632 | FABP4 | P15090 | 323447.8886 |
| 633 | RPL22L1 | Q6P5R6 | 322539.0193 |
| 634 | COX7A2 | P14406 | 321551.852 |
| 635 | USP14 | P54578 | 321374.8436 |
| 636 | RANGAP1 | P46060 | 321112.1352 |
| 637 | PEA15 | Q15121 | 320909.1271 |
| 638 | DTYMK | P23919 | 320633.1438 |
| 639 | ALYREF | Q86V81 | 319249.8864 |
| 640 | EIF2S2 | P20042 | 319156.9632 |
| 641 | SLC25A24 | Q6NUK1 | 318687.0527 |
| 642 | PRMT1 | Q99873 | 318555.415 |
| 643 | ALDH1A1 | P00352 | 318381.2276 |
| 644 | PSPC1 | Q8WXF1 | 318170.4633 |
| 645 | HARS | P12081 | 316801.2984 |
| 646 | ITGA2 | P17301 | 316715.9956 |
| 647 | TRIM28 | Q13263 | 316162.9485 |
| 648 | COPG1 | Q9Y678 | 315986.1732 |
| 649 | PDHB | P11177 | 315781.2032 |
| 650 | KRT10 | P13645 | 314908.4293 |
| 651 | AP2S1 | P53680 | 314721.7408 |
| 652 | CAND1 | Q86VP6 | 314501.1243 |
| 653 | AKR1A1 | P14550 | 313748.7113 |
| 654 | ACOT13 | Q9NPJ3 | 312440.3763 |
| 655 | SPTBN1 | Q01082 | 312277.2963 |
| 656 | GFUS | Q13630 | 311821.0228 |
| 657 | BASP1 | P80723 | 311792.9876 |
| 658 | GYG1 | P46976 | 309854.7814 |
| 659 | GNS | P15586 | 309226.6505 |
| 660 | NT5E | P21589 | 309003.0289 |
| 661 | DBNL | Q9UJU6 | 308015.0775 |
| 662 | DDX5 | P17844 | 307749.8371 |
| 663 | PSMC6 | P62333 | 306922.4783 |
| 664 | RAB22A | Q9UL26 | 306683.5953 |
| 665 | HNRNPL | P14866 | 306672.4559 |
| 666 | CPPED1 | Q9BRF8 | 306303.8405 |
| 667 | SRSF9 | Q13242 | 306161.1973 |
| 668 | EIF3K | Q9UBQ5 | 305754.1828 |
| 669 | UCHL3 | P15374 | 305236.2119 |
| 670 | KIF5B | P33176 | 304965.225 |
| 671 | DDX17 | Q92841 | 304414.424 |
| 672 | RAB6A | P20340 | 303315.4743 |
| 673 | CACYBP | Q9HB71 | 302661.0905 |
| 674 | CORO1B | Q9BR76 | 302457.9478 |
| 675 | IDH1 | O75874 | 302135.3925 |
| 676 | NME1 | P15531 | 301972.8297 |
| 677 | RPS27 | P42677 | 301762.0868 |
| 678 | PLOD1 | Q02809 | 301735.7268 |
| 679 | BLVRA | P53004 | 301069.8736 |
| 680 | HEXIM1 | O94992 | 301066.4293 |
| 681 | EIF3H | O15372 | 300768.981 |
| 682 | GRHPR | Q9UBQ7 | 300588.7763 |
| 683 | RPS15 | P62841 | 299998.3945 |
| 684 | RAB28 | P51157 | 299216.3113 |
| 685 | EIF3B | P55884 | 298104.7584 |
| 686 | CAST | P20810 | 297720.1708 |
| 687 | USP5 | P45974 | 297514.4534 |
| 688 | AK1 | P00568 | 297347.571 |
| 689 | SLC25A6 | P12236 | 296991.3052 |
| 690 | ACAA2 | P42765 | 296168.5568 |
| 691 | LMAN1 | P49257 | 295389.2179 |
| 692 | SNAP29 | O95721 | 295266.8783 |
| 693 | MATR3 | P43243 | 295211.53 |
| 694 | STOML2 | Q9UJZ1 | 293844.6824 |
| 695 | HP1BP3 | Q5SSJ5 | 291215.5241 |
| 696 | LYPLA1 | O75608 | 290523.6513 |
| 697 | NSFL1C | Q9UNZ2 | 290505.836 |
| 698 | PFDN6 | O15212 | 290213.8528 |
| 699 | TXNDC12 | O95881 | 289806.2391 |
| 700 | EZR | P15311 | 289689.3182 |
| 701 | UBE2M | P61081 | 288501.7952 |
| 702 | HMGB1 | P09429 | 288080.5059 |
| 703 | HMOX2 | P30519 | 287499.4019 |
| 704 | PCMT1 | P22061 | 287235.0838 |
| 705 | PFN2 | P35080 | 286782.0111 |
| 706 | YBX3 | P16989 | 286756.3995 |
| 707 | CSE1L | P55060 | 285831.5085 |
| 708 | ECHS1 | P30084 | 285693.2776 |
| 709 | CRKL | P46109 | 285614.2039 |
| 710 | SPTAN1 | Q13813 | 284531.1898 |
| 711 | CRK | P46108 | 282798.5744 |
| 712 | FAM114A1 | Q8IWE2 | 282778.9049 |
| 713 | PLD3 | Q8IV08 | 282487.6191 |
| 714 | EIF5 | P55010 | 282458.2909 |
| 715 | NEK6 | Q9HC98 | 282273.4772 |
| 716 | LAMTOR4 | Q0VGL1 | 281971.126 |
| 717 | AK2 | P54819 | 281348.2076 |
| 718 | EEF1E1 | O43324 | 280168.6298 |
| 719 | PPP1R7 | Q15435 | 279573.1197 |
| 720 | NARS1 | O43776 | 279535.4622 |
| 721 | EIF3I | Q13347 | 279317.8928 |
| 722 | MANF | P55145 | 279112.4409 |
| 723 | EIF3A | Q14152 | 278726.139 |
| 724 | TP53I3 | Q53FA7 | 278447.038 |
| 725 | AKR1C3 | P42330 | 278357.1004 |
| 726 | GDI1 | P31150 | 278170.5647 |
| 727 | CLTA | P09496 | 277678.0486 |
| 728 | F11R | Q9Y624 | 277657.1814 |
| 729 | RCN3 | Q96D15 | 277217.9504 |
| 730 | TFG | Q92734 | 276902.4587 |
| 731 | EPRS1 | P07814 | 276569.2629 |
| 732 | EDF1 | O60869 | 275674.4071 |
| 733 | API5 | Q9BZZ5 | 275500.7243 |
| 734 | HTRA1 | Q92743 | 274932.2006 |
| 735 | NAP1L4 | Q99733 | 274930.0893 |
| 736 | EIF2S3 | P41091 | 274671.7501 |
| 737 | LRRC47 | Q8N1G4 | 273892.7074 |
| 738 | PSME2 | Q9UL46 | 272771.8969 |
| 739 | XPNPEP3 | Q9NQH7 | 272149.7322 |
| 740 | AARS1 | P49588 | 270005.9729 |
| 741 | ESD | P10768 | 269461.4623 |
| 742 | PROCR | Q9UNN8 | 269454.5394 |
| 743 | NME2 | P22392 | 269453.8026 |
| 744 | HSD17B4 | P51659 | 268774.9475 |
| 745 | RPL18A | Q02543 | 268258.0709 |
| 746 | KARS1 | Q15046 | 266162.5838 |
| 747 | SNX12 | Q9UMY4 | 266009.0748 |
| 748 | SUMF2 | Q8NBJ7 | 265927.8047 |
| 749 | MTHFD1 | P11586 | 265821.6498 |
| 750 | TMED9 | Q9BVK6 | 264662.8177 |
| 751 | LARS1 | Q9P2J5 | 264054.188 |
| 752 | LAP3 | P28838 | 263845.9139 |
| 753 | TMOD3 | Q9NYL9 | 263581.4203 |
| 754 | EIF3J | O75822 | 263371.3075 |
| 755 | ATL3 | Q6DD88 | 263248.6355 |
| 756 | RAB1B | Q9H0U4 | 263185.9403 |
| 757 | GOLT1B | Q9Y3E0 | 262730.9743 |
| 758 | HNRNPH3 | P31942 | 262679.8407 |
| 759 | ABCE1 | P61221 | 261578.0107 |
| 760 | GSTK1 | Q9Y2Q3 | 261109.999 |
| 761 | SRP14 | P37108 | 261022.1404 |
| 762 | PSMD2 | Q13200 | 260166.0843 |
| 763 | PTGR1 | Q14914 | 259052.6198 |
| 764 | GOT1 | P17174 | 259021.5282 |
| 765 | MAT2A | P31153 | 258738.0707 |
| 766 | LTA4H | P09960 | 257650.6042 |
| 767 | SKP1 | P63208 | 257595.0587 |
| 768 | RAB14 | P61106 | 257477.8305 |
| 769 | ALDH18A1 | P54886 | 257098.8821 |
| 770 | IARS1 | P41252 | 256313.738 |
| 771 | EIF4G1 | Q04637 | 256132.2398 |
| 772 | MMRN2 | Q9H8L6 | 255892.3061 |
| 773 | SNU13 | P55769 | 255703.0175 |
| 774 | SPCS3 | P61009 | 255405.1124 |
| 775 | ACO2 | Q99798 | 255368.4709 |
| 776 | ETF1 | P62495 | 255313.3424 |
| 777 | PSMD5 | Q16401 | 255061.1985 |
| 778 | TUBA4B | Q9H853 | 253844.19 |
| 779 | C1QBP | Q07021 | 253588.2659 |
| 780 | CEP170 | Q5SW79 | 253488.518 |
| 781 | TRAPPC3 | O43617 | 253471.5991 |
| 782 | BLVRB | P30043 | 253122.7459 |
| 783 | HEXA | P06865 | 252809.595 |
| 784 | ENG | P17813 | 252774.0594 |
| 785 | GPX8 | Q8TED1 | 252507.3631 |
| 786 | FKBP9 | O95302 | 252207.4017 |
| 787 | UBE2K | P61086 | 251993.4652 |
| 788 | FUS | P35637 | 251777.4402 |
| 789 | JPT2 | Q9H910 | 251097.9043 |
| 790 | DDB1 | Q16531 | 250446.1176 |
| 791 | ATP1B3 | P54709 | 249343.6529 |
| 792 | EIF3D | O15371 | 249237.8058 |
| 793 | PSMB6 | P28072 | 248052.8801 |
| 794 | PDCD5 | O14737 | 247997.9966 |
| 795 | MAPK1 | P28482 | 247821.7275 |
| 796 | OLA1 | Q9NTK5 | 247756.4503 |
| 797 | TARS1 | P26639 | 247422.7785 |
| 798 | YKT6 | O15498 | 247346.1321 |
| 799 | ACADM | P11310 | 247254.9317 |
| 800 | HEBP2 | Q9Y5Z4 | 246948.2323 |
| 801 | PAFAH1B1 | P43034 | 246755.6319 |
| 802 | ERLIN2 | O94905 | 246701.0078 |
| 803 | TRA2B | P62995 | 246596.7106 |
| 804 | DDAH2 | O95865 | 246267.9651 |
| 805 | KHDRBS1 | Q07666 | 246102.1645 |
| 806 | P4HA1 | P13674 | 245993.2524 |
| 807 | PEPD | P12955 | 245348.1707 |
| 808 | PMM2 | O15305 | 244729.0951 |
| 809 | SFXN1 | Q9H9B4 | 244163.3824 |
| 810 | FERMT2 | Q96AC1 | 243773.165 |
| 811 | SFXN3 | Q9BWM7 | 243762.2059 |
| 812 | FARSA | Q9Y285 | 243442.7542 |
| 813 | PRKDC | P78527 | 243184.4066 |
| 814 | KRT9 | P35527 | 243077.9225 |
| 815 | AP2M1 | Q96CW1 | 242945.2373 |
| 816 | VTN | P04004 | 242584.2475 |
| 817 | MAOA | P21397 | 242488.7316 |
| 818 | AP2B1 | P63010 | 242407.291 |
| 819 | MRPL12 | P52815 | 241396.3991 |
| 820 | VAMP5 | O95183 | 241349.0979 |
| 821 | IDH2 | P48735 | 240430.659 |
| 822 | VAPA | Q9P0L0 | 240381.4439 |
| 823 | ATP6V1G1 | O75348 | 239835.7913 |
| 824 | MAT2B | Q9NZL9 | 239358.1014 |
| 825 | CCN2 | P29279 | 239293.8148 |
| 826 | SARS1 | P49591 | 237987.7695 |
| 827 | CBX3 | Q13185 | 237503.5965 |
| 828 | FLOT2 | Q14254 | 237488.6622 |
| 829 | EIF3C | Q99613 | 237387.0919 |
| 830 | SUGT1 | Q9Y2Z0 | 236542.6876 |
| 831 | PRKAR1A | P10644 | 236405.4758 |
| 832 | PGM3 | O95394 | 236090.0142 |
| 833 | HEBP1 | Q9NRV9 | 235997.7647 |
| 834 | DYNC1LI1 | Q9Y6G9 | 235971.3583 |
| 835 | VARS1 | P26640 | 235142.5797 |
| 836 | PGRMC1 | O00264 | 234914.8514 |
| 837 | P4HA2 | O15460 | 234102.6248 |
| 838 | SF3B3 | Q15393 | 234060.3756 |
| 839 | PSMD1 | Q99460 | 233946.2162 |
| 840 | RECQL | P46063 | 233457.3902 |
| 841 | EIF3L | Q9Y262 | 232826.0869 |
| 842 | DDX1 | Q92499 | 232556.4802 |
| 843 | DDT | P30046 | 232437.9659 |
| 844 | VPS35 | Q96QK1 | 232017.2671 |
| 845 | FLOT1 | O75955 | 231933.0698 |
| 846 | AHSA1 | O95433 | 231579.0461 |
| 847 | NDUFS3 | O75489 | 231028.5809 |
| 848 | ASPH | Q12797 | 230776.7771 |
| 849 | SNRPB | P14678 | 230738.1511 |
| 850 | IGF2BP2 | Q9Y6M1 | 229283.8864 |
| 851 | SF3B2 | Q13435 | 228397.9863 |
| 852 | TJP2 | Q9UDY2 | 228146.11 |
| 853 | GOSR2 | O14653 | 227650.8457 |
| 854 | YIPF5 | Q969M3 | 227285.155 |
| 855 | SMAGP | Q0VAQ4 | 227225.93 |
| 856 | PITPNB | P48739 | 226760.3442 |
| 857 | SRPX | P78539 | 226334.5351 |
| 858 | KTN1 | Q86UP2 | 226230.7931 |
| 859 | DYNC1LI2 | O43237 | 225399.3348 |
| 860 | PYCR1 | P32322 | 225270.2642 |
| 861 | NECAP2 | Q9NVZ3 | 224529.3274 |
| 862 | RCC1 | P18754 | 224445.2572 |
| 863 | MTDH | Q86UE4 | 224368.1113 |
| 864 | GSR | P00390 | 224089.4042 |
| 865 | PGM1 | P36871 | 223779.7251 |
| 866 | UAP1 | Q16222 | 222867.0432 |
| 867 | EIF4G2 | P78344 | 222764.696 |
| 868 | PSMD6 | Q15008 | 222380.4773 |
| 869 | NDRG1 | Q92597 | 222322.4282 |
| 870 | KPNA2 | P52292 | 221873.9531 |
| 871 | ADI1 | Q9BV57 | 221802.8321 |
| 872 | ALDH7A1 | P49419 | 221765.4973 |
| 873 | NUDT21 | O43809 | 221757.8728 |
| 874 | MAPK3 | P27361 | 221641.0918 |
| 875 | RNPS1 | Q15287 | 220989.7212 |
| 876 | MOGS | Q13724 | 220963.503 |
| 877 | AP2A1 | O95782 | 220493.6804 |
| 878 | SF3A3 | Q12874 | 220483.291 |
| 879 | CPSF6 | Q16630 | 219982.3641 |
| 880 | PUF60 | Q9UHX1 | 219855.8992 |
| 881 | IMPA1 | P29218 | 219371.6544 |
| 882 | FAM107B | Q9H098 | 219244.2073 |
| 883 | OSTF1 | Q92882 | 218759.8456 |
| 884 | RASIP1 | Q5U651 | 218735.6529 |
| 885 | OCIAD1 | Q9NX40 | 218682.983 |
| 886 | SPARC | P09486 | 218368.8309 |
| 887 | AHNAK2 | Q8IVF2 | 218135.7135 |
| 888 | PCNP | Q8WW12 | 217561.6955 |
| 889 | FTH1 | P02794 | 217470.1933 |
| 890 | RHOG | P84095 | 216857.4831 |
| 891 | UGDH | O60701 | 216746.5537 |
| 892 | PFKL | P17858 | 216693.1339 |
| 893 | EIF2A | Q9BY44 | 216478.0452 |
| 894 | ATP6V1A | P38606 | 216224.9693 |
| 895 | STX7 | O15400 | 216209.5058 |
| 896 | CTNNB1 | P35222 | 216185.4561 |
| 897 | ALDH9A1 | P49189 | 214687.2041 |
| 898 | VMA21 | Q3ZAQ7 | 214379.7978 |
| 899 | PLGRKT | Q9HBL7 | 214338.2369 |
| 900 | NNT | Q13423 | 214088.1737 |
| 901 | REEP5 | Q00765 | 213703.6987 |
| 902 | RAP1B | P61224 | 213218.0259 |
| 903 | CNRIP1 | Q96F85 | 212629.4781 |
| 904 | CRTAP | O75718 | 211754.5876 |
| 905 | SEC61G | P60059 | 211384.2853 |
| 906 | RSL1D1 | O76021 | 210947.8603 |
| 907 | PFDN5 | Q99471 | 210464.1993 |
| 908 | SRSF2 | Q01130 | 210267.4992 |
| 909 | EPB41L3 | Q9Y2J2 | 210084.4116 |
| 910 | S100A16 | Q96FQ6 | 209734.487 |
| 911 | CDH5 | P33151 | 209300.9549 |
| 912 | PBDC1 | Q9BVG4 | 208888.8843 |
| 913 | USO1 | O60763 | 208456.8268 |
| 914 | EEA1 | Q15075 | 208007.0206 |
| 915 | ZNF185 | O15231 | 207943.4593 |
| 916 | LMAN2 | Q12907 | 207390.9933 |
| 917 | ARF5 | P84085 | 207170.0878 |
| 918 | NOP58 | Q9Y2X3 | 206642.4108 |
| 919 | NDUFS1 | P28331 | 206198.9854 |
| 920 | GNA13 | Q14344 | 205719.4575 |
| 921 | FKBP4 | Q02790 | 205095.0253 |
| 922 | SEC24C | P53992 | 205042.4781 |
| 923 | SF1 | Q15637 | 204647.1678 |
| 924 | UFD1 | Q92890 | 204446.1544 |
| 925 | DYNC1I2 | Q13409 | 204118.3196 |
| 926 | U2AF2 | P26368 | 203794.1419 |
| 927 | SWAP70 | Q9UH65 | 203401.9238 |
| 928 | PODXL | O00592 | 203350.7377 |
| 929 | TMX3 | Q96JJ7 | 202601.2714 |
| 930 | SEC23A | Q15436 | 202511.7397 |
| 931 | ARL2 | P36404 | 202507.5977 |
| 932 | HIGD1A | Q9Y241 | 202377.6691 |
| 933 | PDLIM4 | P50479 | 202075.4413 |
| 934 | ARF6 | P62330 | 201805.2637 |
| 935 | UBA2 | Q9UBT2 | 201115.795 |
| 936 | RABIF | P47224 | 200701.8908 |
| 937 | PLOD3 | O60568 | 200573.1207 |
| 938 | MCTS1 | Q9ULC4 | 200237.5272 |
| 939 | MYCT1 | Q8N699 | 200146.841 |
| 940 | MPDU1 | O75352 | 199906.8152 |
| 941 | NOP56 | O00567 | 199786.1705 |
| 942 | AIMP1 | Q12904 | 199407.743 |
| 943 | DECR1 | Q16698 | 199259.41 |
| 944 | IPO5 | O00410 | 199238.3904 |
| 945 | STT3A | P46977 | 199168.8544 |
| 946 | LLPH | Q9BRT6 | 199136.8707 |
| 947 | ZNF207 | O43670 | 198849.6563 |
| 948 | COPS4 | Q9BT78 | 198594.323 |
| 949 | CLTB | P09497 | 198425.4413 |
| 950 | BZW2 | Q9Y6E2 | 198209.8629 |
| 951 | QARS1 | P47897 | 197954.2494 |
| 952 | ACO1 | P21399 | 197923.6006 |
| 953 | SYNGR2 | O43760 | 197835.6422 |
| 954 | RTRAF | Q9Y224 | 197808.4886 |
| 955 | NUP93 | Q8N1F7 | 197448.1819 |
| 956 | SEPTIN11 | Q9NVA2 | 197396.6645 |
| 957 | PVR | P15151 | 196206.8689 |
| 958 | CLINT1 | Q14677 | 195922.0689 |
| 959 | SNX1 | Q13596 | 195740.4008 |
| 960 | RAB2A | P61019 | 195677.01 |
| 961 | TSN | Q15631 | 195673.9258 |
| 962 | FUBP1 | Q96AE4 | 195553.2524 |
| 963 | AP1M1 | Q9BXS5 | 195366.5949 |
| 964 | TARDBP | Q13148 | 195350.7092 |
| 965 | ADD1 | P35611 | 194951.5825 |
| 966 | TXNDC9 | O14530 | 194809.0094 |
| 967 | PGM2 | Q96G03 | 194737.3468 |
| 968 | IST1 | P53990 | 194578.0158 |
| 969 | DPP3 | Q9NY33 | 194250.4194 |
| 970 | LIMA1 | Q9UHB6 | 193238.999 |
| 971 | IMMT | Q16891 | 193078.7207 |
| 972 | MYH10 | P35580 | 192325.3389 |
| 973 | ECE1 | P42892 | 192133.2174 |
| 974 | PRKAR2A | P13861 | 191624.2051 |
| 975 | ARL3 | P36405 | 191432.4605 |
| 976 | PTX3 | P26022 | 191082.4891 |
| 977 | ERGIC1 | Q969X5 | 190949.3194 |
| 978 | GART | P22102 | 190868.3456 |
| 979 | HSPH1 | Q92598 | 190779.8422 |
| 980 | CREB1 | P16220 | 190655.938 |
| 981 | DNM1L | O00429 | 190528.6999 |
| 982 | ARL1 | P40616 | 189823.0473 |
| 983 | PABPC1 | P11940 | 189760.077 |
| 984 | TUBB3 | Q13509 | 189561.8172 |
| 985 | GSS | P48637 | 189442.2528 |
| 986 | PACSIN2 | Q9UNF0 | 189261.4883 |
| 987 | DNAJC3 | Q13217 | 188989.6702 |
| 988 | SNX2 | O60749 | 188617.7172 |
| 989 | COMT | P21964 | 188375.8446 |
| 990 | PYGB | P11216 | 188370.3948 |
| 991 | PCBD1 | P61457 | 188247.1059 |
| 992 | LAMTOR1 | Q6IAA8 | 188145.8326 |
| 993 | MTCH2 | Q9Y6C9 | 188000.4294 |
| 994 | PPP1R14B | Q96C90 | 187353.672 |
| 995 | COPS6 | Q7L5N1 | 186971.4196 |
| 996 | H1-4 | P10412 | 186456.0128 |
| 997 | AP1B1 | Q10567 | 186342.4189 |
| 998 | DNAJC8 | O75937 | 186263.6576 |
| 999 | SRPRA | P08240 | 186233.8207 |
| 1000 | SDHB | P21912 | 185858.9706 |
| 1001 | AP3S1 | Q92572 | 185460.4327 |
| 1002 | DYNLT1 | P63172 | 185196.1373 |
| 1003 | SEPTIN9 | Q9UHD8 | 185056.9823 |
| 1004 | URM1 | Q9BTM9 | 185026.622 |
| 1005 | SCRN1 | Q12765 | 184360.0567 |
| 1006 | PPP1CB | P62140 | 184281.5181 |
| 1007 | RAB35 | Q15286 | 183948.0798 |
| 1008 | COX6C | P09669 | 183940.382 |
| 1009 | COPS8 | Q99627 | 183396.0791 |
| 1010 | UGGT1 | Q9NYU2 | 183302.5924 |
| 1011 | EIF4B | P23588 | 182949.9024 |
| 1012 | PSMF1 | Q92530 | 182171.6243 |
| 1013 | SLC25A11 | Q02978 | 182080.2127 |
| 1014 | TST | Q16762 | 181647.8731 |
| 1015 | SUCLG2 | Q96I99 | 181601.9974 |
| 1016 | SRSF6 | Q13247 | 181353.8936 |
| 1017 | DCTN1 | Q14203 | 181018.2588 |
| 1018 | CUTA | O60888 | 180572.1713 |
| 1019 | LRPPRC | P42704 | 179987.354 |
| 1020 | DERA | Q9Y315 | 179780.8133 |
| 1021 | PPP1CA | P62136 | 179417.0771 |
| 1022 | RBM8A | Q9Y5S9 | 178053.1433 |
| 1023 | PSMD9 | O00233 | 177937.9945 |
| 1024 | DRG1 | Q9Y295 | 177750.7883 |
| 1025 | PITRM1 | Q5JRX3 | 177610.4917 |
| 1026 | CASP3 | P42574 | 177448.7387 |
| 1027 | SEC11A | P67812 | 177329.6607 |
| 1028 | GPX4 | P36969 | 177111.2899 |
| 1029 | VWF | P04275 | 177053.7496 |
| 1030 | DNAJB11 | Q9UBS4 | 176837.8028 |
| 1031 | DDAH1 | O94760 | 176533.3382 |
| 1032 | GFPT1 | Q06210 | 176447.5854 |
| 1033 | PPM1G | O15355 | 176219.0277 |
| 1034 | RNPEP | Q9H4A4 | 175974.0505 |
| 1035 | SAE1 | Q9UBE0 | 175931.6859 |
| 1036 | TIMM13 | Q9Y5L4 | 175773.8703 |
| 1037 | MARS1 | P56192 | 175697.8114 |
| 1038 | ARHGEF1 | Q92888 | 175652.1394 |
| 1039 | SNX6 | Q9UNH7 | 175397.3308 |
| 1040 | STAT1 | P42224 | 175384.1453 |
| 1041 | TOMM40 | O96008 | 175281.1572 |
| 1042 | PABPC4 | Q13310 | 175225.6384 |
| 1043 | DCTN4 | Q9UJW0 | 174499.9193 |
| 1044 | VTA1 | Q9NP79 | 174176.9817 |
| 1045 | HNRNPDL | O14979 | 173456.0219 |
| 1046 | PCYOX1 | Q9UHG3 | 173311.4902 |
| 1047 | CTNND1 | O60716 | 173193.2854 |
| 1048 | DR1 | Q01658 | 172930.7627 |
| 1049 | H2AC21 | Q8IUE6 | 172893.04 |
| 1050 | NCLN | Q969V3 | 172692.4707 |
| 1051 | UCHL5 | Q9Y5K5 | 172398.3756 |
| 1052 | GALK1 | P51570 | 172256.3946 |
| 1053 | FIS1 | Q9Y3D6 | 172226.6579 |
| 1054 | EWSR1 | Q01844 | 172200.441 |
| 1055 | POLR1C | O15160 | 171757.2507 |
| 1056 | EFTUD2 | Q15029 | 171736.7402 |
| 1057 | ARHGAP1 | Q07960 | 171466.7451 |
| 1058 | CSDE1 | O75534 | 171457.3059 |
| 1059 | RAB32 | Q13637 | 171348.2045 |
| 1060 | QKI | Q96PU8 | 171033.7275 |
| 1061 | MBNL1 | Q9NR56 | 170646.9483 |
| 1062 | #N/A | Q6ZSR9 | 170297.5682 |
| 1063 | CPT1A | P50416 | 169986.7143 |
| 1064 | CHMP2A | O43633 | 169687.0282 |
| 1065 | HMGB2 | P26583 | 169600.3756 |
| 1066 | SEC14L2 | O76054 | 169287.6376 |
| 1067 | FUBP3 | Q96I24 | 169186.99 |
| 1068 | DNAJC9 | Q8WXX5 | 168960.8308 |
| 1069 | RAB13 | P51153 | 168797.164 |
| 1070 | TMEM33 | P57088 | 168668.6759 |
| 1071 | PSAT1 | Q9Y617 | 168461.4834 |
| 1072 | APEX1 | P27695 | 168085.2643 |
| 1073 | PTPN1 | P18031 | 168016.9711 |
| 1074 | AAMDC | Q9H7C9 | 167859.1321 |
| 1075 | CD81 | P60033 | 167805.8037 |
| 1076 | P3H1 | Q32P28 | 167703.4006 |
| 1077 | PTPA | Q15257 | 167676.7956 |
| 1078 | GMPS | P49915 | 167664.9924 |
| 1079 | SCCPDH | Q8NBX0 | 167307.9436 |
| 1080 | PRPF19 | Q9UMS4 | 167195.3296 |
| 1081 | CDH13 | P55290 | 166889.0049 |
| 1082 | NUCB1 | Q02818 | 166873.6534 |
| 1083 | PRPF31 | Q8WWY3 | 166728.138 |
| 1084 | NDUFB3 | O43676 | 166369.9545 |
| 1085 | ARSA | P15289 | 166369.1996 |
| 1086 | PDCD10 | Q9BUL8 | 166157.9379 |
| 1087 | MT-ATP6 | P00846 | 166036.9593 |
| 1088 | NDUFA13 | Q9P0J0 | 165912.303 |
| 1089 | DUSP23 | Q9BVJ7 | 165869.9792 |
| 1090 | RALYL | Q86SE5 | 165097.0467 |
| 1091 | WDR77 | Q9BQA1 | 165086.0977 |
| 1092 | NDUFA2 | O43678 | 164838.8978 |
| 1093 | HNRNPH2 | P55795 | 164736.1347 |
| 1094 | PDIA5 | Q14554 | 164607.3238 |
| 1095 | GNA11 | P29992 | 164396.4733 |
| 1096 | NIT2 | Q9NQR4 | 164346.56 |
| 1097 | SERPINB8 | P50452 | 164170.7446 |
| 1098 | MAP2K1 | Q02750 | 164123.6197 |
| 1099 | FARSB | Q9NSD9 | 163640.5594 |
| 1100 | GORASP2 | Q9H8Y8 | 163533.1879 |
| 1101 | CCDC50 | Q8IVM0 | 163471.3031 |
| 1102 | DLAT | P10515 | 163423.1035 |
| 1103 | HDGFL3 | Q9Y3E1 | 163366.8832 |
| 1104 | ADPRS | Q9NX46 | 163298.2092 |
| 1105 | RAI14 | Q9P0K7 | 163124.4839 |
| 1106 | PTBP2 | Q9UKA9 | 163078.4233 |
| 1107 | BAG2 | O95816 | 163057.0996 |
| 1108 | MAPK1IP1L | Q8NDC0 | 162887.24 |
| 1109 | SGTA | O43765 | 162426.0206 |
| 1110 | SRSF5 | Q13243 | 162350.9519 |
| 1111 | HMOX1 | P09601 | 161606.3173 |
| 1112 | ATP1B1 | P05026 | 161565.9689 |
| 1113 | GRPEL1 | Q9HAV7 | 161319.9901 |
| 1114 | LPP | Q93052 | 161219.4846 |
| 1115 | TSG101 | Q99816 | 160826.7213 |
| 1116 | GET3 | O43681 | 160607.5698 |
| 1117 | RAB1A | P62820 | 160543.3988 |
| 1118 | PSPH | P78330 | 160313.4653 |
| 1119 | PPP2R2A | P63151 | 159943.5967 |
| 1120 | NOP2 | P46087 | 159676.5751 |
| 1121 | SF3A1 | Q15459 | 159599.0513 |
| 1122 | CAT | P04040 | 159499.2412 |
| 1123 | ARL8B | Q9NVJ2 | 158180.5483 |
| 1124 | NAA50 | Q9GZZ1 | 158064.2783 |
| 1125 | GMPPB | Q9Y5P6 | 157912.9365 |
| 1126 | ENO2 | P09104 | 157887.6935 |
| 1127 | HNRNPUL2 | Q1KMD3 | 157696.9401 |
| 1128 | RO60 | P10155 | 157640.1411 |
| 1129 | PITPNA | Q00169 | 157325.0379 |
| 1130 | NCEH1 | Q6PIU2 | 156987.8495 |
| 1131 | SNTB2 | Q13425 | 156792.9596 |
| 1132 | KPNA3 | O00505 | 156604.0876 |
| 1133 | PALMD | Q9NP74 | 156508.538 |
| 1134 | CCAR2 | Q8N163 | 156422.1621 |
| 1135 | RAD23A | P54725 | 156250.7937 |
| 1136 | PLPBP | O94903 | 156221.0218 |
| 1137 | TOMM22 | Q9NS69 | 156198.764 |
| 1138 | EMC7 | Q9NPA0 | 155950.0144 |
| 1139 | PABPN1 | Q86U42 | 155676.0224 |
| 1140 | FKBP11 | Q9NYL4 | 155673.6359 |
| 1141 | HSPA6 | P17066 | 155185.0659 |
| 1142 | ATP5MK | Q96IX5 | 155158.4993 |
| 1143 | COLGALT1 | Q8NBJ5 | 155118.4387 |
| 1144 | ARPC1A | Q92747 | 155043.3782 |
| 1145 | NANS | Q9NR45 | 154905.8298 |
| 1146 | DEK | P35659 | 154678.3809 |
| 1147 | GLIPR2 | Q9H4G4 | 154633.6704 |
| 1148 | HNRNPR | O43390 | 154582.6636 |
| 1149 | NUP62 | P37198 | 154448.5411 |
| 1150 | LSM2 | Q9Y333 | 154423.457 |
| 1151 | DBI | P07108 | 154122.5848 |
| 1152 | PIR | O00625 | 154036.5779 |
| 1153 | UQCRC1 | P31930 | 153976.5527 |
| 1154 | NDUFV2 | P19404 | 153874.4546 |
| 1155 | FKBP5 | Q13451 | 153743.3752 |
| 1156 | DDX6 | P26196 | 153593.639 |
| 1157 | CHMP6 | Q96FZ7 | 153022.1381 |
| 1158 | DERL1 | Q9BUN8 | 152961.0236 |
| 1159 | PRRC1 | Q96M27 | 152869.8767 |
| 1160 | CAPG | P40121 | 152698.1379 |
| 1161 | SERPINB1 | P30740 | 152679.9382 |
| 1162 | ECH1 | Q13011 | 152425.0717 |
| 1163 | LSM7 | Q9UK45 | 152064.1448 |
| 1164 | CYB5B | O43169 | 151937.9939 |
| 1165 | DRAP1 | Q14919 | 151878.3033 |
| 1166 | PICALM | Q13492 | 151570.9715 |
| 1167 | ADRM1 | Q16186 | 151564.5977 |
| 1168 | XPNPEP1 | Q9NQW7 | 151491.0273 |
| 1169 | C11orf68 | Q9H3H3 | 151474.5341 |
| 1170 | C12orf57 | Q99622 | 151391.1944 |
| 1171 | ACTR1A | P61163 | 151386.1535 |
| 1172 | SRP68 | Q9UHB9 | 151013.8126 |
| 1173 | RAB5A | P20339 | 150618.1924 |
| 1174 | MPST | P25325 | 150415.9234 |
| 1175 | LONP1 | P36776 | 150400.7277 |
| 1176 | XPO1 | O14980 | 150299.0171 |
| 1177 | PYCARD | Q9ULZ3 | 150130.6914 |
| 1178 | VBP1 | P61758 | 149780.1659 |
| 1179 | TNPO1 | Q92973 | 149742.9858 |
| 1180 | GCN1 | Q92616 | 149686.5705 |
| 1181 | GPS1 | Q13098 | 149201.8904 |
| 1182 | UQCRC2 | P22695 | 148980.2531 |
| 1183 | LXN | Q9BS40 | 148973.3658 |
| 1184 | EPHX1 | P07099 | 148793.8287 |
| 1185 | NHP2 | Q9NX24 | 148771.6373 |
| 1186 | LAMB1 | P07942 | 148491.0738 |
| 1187 | COA3 | Q9Y2R0 | 148230.3467 |
| 1188 | CD63 | P08962 | 148128.0979 |
| 1189 | CARS1 | P49589 | 148115.1137 |
| 1190 | PARP1 | P09874 | 147833.3929 |
| 1191 | TMCO1 | Q9UM00 | 147777.7147 |
| 1192 | UFC1 | Q9Y3C8 | 147215.5673 |
| 1193 | TFRC | P02786 | 147179.1981 |
| 1194 | CARM1 | Q86X55 | 146988.8155 |
| 1195 | DNAJC7 | Q99615 | 146690.9726 |
| 1196 | TBCB | Q99426 | 146579.6334 |
| 1197 | HCLS1 | P14317 | 146453.383 |
| 1198 | NASP | P49321 | 146437.5388 |
| 1199 | DAB2 | P98082 | 146356.0552 |
| 1200 | SCAMP3 | O14828 | 146141.4995 |
| 1201 | UROD | P06132 | 145842.3244 |
| 1202 | COX20 | Q5RI15 | 145728.2917 |
| 1203 | CSNK2A1 | P68400 | 145342.1266 |
| 1204 | PYGL | P06737 | 145313.184 |
| 1205 | CZIB | Q9NWV4 | 145269.1529 |
| 1206 | CNIH4 | Q9P003 | 145134.585 |
| 1207 | ERLIN1 | O75477 | 145080.4782 |
| 1208 | CCT6B | Q92526 | 144748.92 |
| 1209 | CFAP20 | Q9Y6A4 | 144472.8825 |
| 1210 | SLC3A2 | P08195 | 144153.6282 |
| 1211 | RAP1GDS1 | P52306 | 144056.7041 |
| 1212 | EMD | P50402 | 143907.3577 |
| 1213 | LRRC40 | Q9H9A6 | 143860.9781 |
| 1214 | IPO7 | O95373 | 143749.3753 |
| 1215 | TTC1 | Q99614 | 143666.2513 |
| 1216 | TCEA1 | P23193 | 143287.7701 |
| 1217 | NAA10 | P41227 | 142656.9055 |
| 1218 | ISYNA1 | Q9NPH2 | 142566.2589 |
| 1219 | TFAM | Q00059 | 142505.7679 |
| 1220 | BTF3L4 | Q96K17 | 142444.3582 |
| 1221 | RPS12 | P25398 | 142216.0974 |
| 1222 | VPS25 | Q9BRG1 | 142215.0796 |
| 1223 | FDXR | P22570 | 142061.5212 |
| 1224 | PDE6D | O43924 | 142023.5383 |
| 1225 | GRB2 | P62993 | 141985.5715 |
| 1226 | NDUFB10 | O96000 | 141910.8603 |
| 1227 | NDUFS7 | O75251 | 141470.8515 |
| 1228 | SEC24D | O94855 | 141435.757 |
| 1229 | MOB4 | Q9Y3A3 | 141257.803 |
| 1230 | QDPR | P09417 | 141008.0986 |
| 1231 | UNC45A | Q9H3U1 | 140876.5465 |
| 1232 | CSNK2B | P67870 | 140596.5754 |
| 1233 | ITGAV | P06756 | 140324.0892 |
| 1234 | EIF4EBP1 | Q13541 | 139970.9958 |
| 1235 | NDUFS5 | O43920 | 139906.0883 |
| 1236 | TOP1 | P11387 | 139637.8932 |
| 1237 | RBM12 | Q9NTZ6 | 139566.9657 |
| 1238 | LAMTOR2 | Q9Y2Q5 | 139533.2935 |
| 1239 | CIAPIN1 | Q6FI81 | 139464.8799 |
| 1240 | CDC42SE2 | Q9NRR3 | 139312.055 |
| 1241 | WDR61 | Q9GZS3 | 139310.9099 |
| 1242 | ANP32B | Q92688 | 139216.4998 |
| 1243 | AP2A2 | O94973 | 139210.7856 |
| 1244 | PGAM5 | Q96HS1 | 139102.8707 |
| 1245 | GGH | Q92820 | 138811.7082 |
| 1246 | LYPLA2 | O95372 | 138690.526 |
| 1247 | YBX1 | P67809 | 138522.399 |
| 1248 | TMPO | P42167 | 138230.9494 |
| 1249 | CD44 | P16070 | 138186.406 |
| 1250 | TWF2 | Q6IBS0 | 138149.9292 |
| 1251 | AP3B1 | O00203 | 137456.6216 |
| 1252 | CNPY4 | Q8N129 | 137437.3945 |
| 1253 | PXN | P49023 | 137294.5066 |
| 1254 | MTHFD1L | Q6UB35 | 137286.8227 |
| 1255 | NDUFB4 | O95168 | 137159.8342 |
| 1256 | GLB1 | P16278 | 136778.6133 |
| 1257 | HADH | Q16836 | 136486.4796 |
| 1258 | MAGED2 | Q9UNF1 | 136379.9671 |
| 1259 | MCM6 | Q14566 | 136127.2213 |
| 1260 | UBA6 | A0AVT1 | 135970.6253 |
| 1261 | RAB8B | Q92930 | 135874.2316 |
| 1262 | AIFM1 | O95831 | 135746.4011 |
| 1263 | CSK | P41240 | 135610.5118 |
| 1264 | FKBPL | Q9UIM3 | 135546.1357 |
| 1265 | CDIPT | O14735 | 135479.0569 |
| 1266 | SMU1 | Q2TAY7 | 135467.8571 |
| 1267 | NUP54 | Q7Z3B4 | 135238.4901 |
| 1268 | UQCR10 | Q9UDW1 | 135021.2093 |
| 1269 | EIF3M | Q7L2H7 | 134932.2555 |
| 1270 | MRTO4 | Q9UKD2 | 134825.9732 |
| 1271 | TGFBI | Q15582 | 134773.3632 |
| 1272 | GNPDA1 | P46926 | 134749.9706 |
| 1273 | SF3B1 | O75533 | 134680.0651 |
| 1274 | PRMT5 | O14744 | 134493.1416 |
| 1275 | IRGQ | Q8WZA9 | 134329.4549 |
| 1276 | NSF | P46459 | 134195.0128 |
| 1277 | IPO9 | Q96P70 | 134107.4663 |
| 1278 | UPF1 | Q92900 | 133964.4458 |
| 1279 | MGST1 | P10620 | 133851.7111 |
| 1280 | NDUFA12 | Q9UI09 | 133770.5203 |
| 1281 | SLIRP | Q9GZT3 | 133525.8915 |
| 1282 | GGCT | O75223 | 133135.9575 |
| 1283 | ZMPSTE24 | O75844 | 133072.6942 |
| 1284 | MRPL41 | Q8IXM3 | 132988.62 |
| 1285 | PCDH1 | Q08174 | 132764.5852 |
| 1286 | ATP6V0D1 | P61421 | 132759.857 |
| 1287 | SVIP | Q8NHG7 | 132672.8075 |
| 1288 | MTAP | Q13126 | 132664.4079 |
| 1289 | CISD2 | Q8N5K1 | 131975.3429 |
| 1290 | JPT1 | Q9UK76 | 131754.4952 |
| 1291 | UBFD1 | O14562 | 131602.6853 |
| 1292 | PSMG1 | O95456 | 131576.7443 |
| 1293 | ITPA | Q9BY32 | 131563.9549 |
| 1294 | EPHA2 | P29317 | 131498.9226 |
| 1295 | SSRP1 | Q08945 | 131484.4446 |
| 1296 | CMTM6 | Q9NX76 | 131423.3565 |
| 1297 | PPT1 | P50897 | 131112.9604 |
| 1298 | SCARB2 | Q14108 | 130949.8123 |
| 1299 | CPOX | P36551 | 130924.1604 |
| 1300 | TIGAR | Q9NQ88 | 130842.5442 |
| 1301 | IGFBP7 | Q16270 | 130715.0174 |
| 1302 | PHGDH | O43175 | 130580.9579 |
| 1303 | DPM1 | O60762 | 130401.7546 |
| 1304 | FXR1 | P51114 | 130314.3917 |
| 1305 | EIF2AK2 | P19525 | 130237.5984 |
| 1306 | TOMM20 | Q15388 | 130202.1326 |
| 1307 | FAM3C | Q92520 | 129667.7241 |
| 1308 | CTPS1 | P17812 | 129477.42 |
| 1309 | DDX21 | Q9NR30 | 129459.7101 |
| 1310 | CPNE3 | O75131 | 129110.8654 |
| 1311 | ACTL6A | O96019 | 129051.3003 |
| 1312 | DIABLO | Q9NR28 | 128729.309 |
| 1313 | ABCF1 | Q8NE71 | 128688.4949 |
| 1314 | ABI1 | Q8IZP0 | 128478.2721 |
| 1315 | TUBB4A | P04350 | 128164.5117 |
| 1316 | PDHA1 | P08559 | 128056.2066 |
| 1317 | APEH | P13798 | 128034.9284 |
| 1318 | DNM2 | P50570 | 127729.9961 |
| 1319 | UBAP2L | Q14157 | 127439.7829 |
| 1320 | IGF2BP3 | O00425 | 127237.6534 |
| 1321 | SMS | P52788 | 126999.9937 |
| 1322 | SRP54 | P61011 | 126496.6725 |
| 1323 | ADSL | P30566 | 126496.3514 |
| 1324 | CABP7 | Q86V35 | 126399.76 |
| 1325 | PPP1R13L | Q8WUF5 | 126123.6166 |
| 1326 | ABCF2 | Q9UG63 | 125968.4282 |
| 1327 | NOS3 | P29474 | 125949.1529 |
| 1328 | JUP | P14923 | 125603.1393 |
| 1329 | MYCBP | Q99417 | 125303.8812 |
| 1330 | ARHGAP18 | Q8N392 | 124974.0853 |
| 1331 | CCDC124 | Q96CT7 | 124960.1743 |
| 1332 | VAPB | O95292 | 124651.6761 |
| 1333 | MYG1 | Q9HB07 | 124239.0899 |
| 1334 | HYI | Q5T013 | 124212.0407 |
| 1335 | SEC13 | P55735 | 124094.2245 |
| 1336 | RPA3 | P35244 | 123991.434 |
| 1337 | CHCHD3 | Q9NX63 | 123857.1712 |
| 1338 | TMED5 | Q9Y3A6 | 123667.637 |
| 1339 | ERH | P84090 | 123461.8043 |
| 1340 | ALCAM | Q13740 | 123185.7271 |
| 1341 | SCFD1 | Q8WVM8 | 122969.5499 |
| 1342 | PPA2 | Q9H2U2 | 122928.5655 |
| 1343 | RER1 | O15258 | 122915.9213 |
| 1344 | ABHD14B | Q96IU4 | 122890.3642 |
| 1345 | HNRNPAB | Q99729 | 122640.0239 |
| 1346 | RPE | Q96AT9 | 122523.9093 |
| 1347 | NCSTN | Q92542 | 122185.4221 |
| 1348 | SUCLA2 | Q9P2R7 | 121955.7929 |
| 1349 | HMGN1 | P05114 | 121899.8525 |
| 1350 | PPIH | O43447 | 121878.9103 |
| 1351 | PDCD4 | Q53EL6 | 121862.9773 |
| 1352 | SORD | Q00796 | 121354.9635 |
| 1353 | ACAA1 | P09110 | 121327.4422 |
| 1354 | ACBD3 | Q9H3P7 | 121279.9473 |
| 1355 | SORBS3 | O60504 | 121268.0501 |
| 1356 | HAGH | Q16775 | 121132.2611 |
| 1357 | COPS2 | P61201 | 120980.4072 |
| 1358 | SNX5 | Q9Y5X3 | 120938.6106 |
| 1359 | TPR | P12270 | 120776.1043 |
| 1360 | PDCD6 | O75340 | 120687.0227 |
| 1361 | CAD | P27708 | 120315.8165 |
| 1362 | RELA | Q04206 | 120213.8841 |
| 1363 | AKR7A2 | O43488 | 120188.3803 |
| 1364 | CISD1 | Q9NZ45 | 120181.1952 |
| 1365 | ALDOC | P09972 | 120134.6263 |
| 1366 | ATXN2L | Q8WWM7 | 120005.8814 |
| 1367 | ATP5PF | P18859 | 119867.2705 |
| 1368 | CD99 | P14209 | 119690.3188 |
| 1369 | RABL3 | Q5HYI8 | 119589.5159 |
| 1370 | IFI16 | Q16666 | 119453.6986 |
| 1371 | PIN4 | Q9Y237 | 119404.1477 |
| 1372 | NDUFS8 | O00217 | 119342.4861 |
| 1373 | SGSH | P51688 | 119168.7008 |
| 1374 | NAE1 | Q13564 | 118878.0686 |
| 1375 | WASL | O00401 | 118839.1267 |
| 1376 | DNASE1L1 | P49184 | 118759.7262 |
| 1377 | ACTBL2 | Q562R1 | 118276.142 |
| 1378 | NUMB | P49757 | 118192.2601 |
| 1379 | AKAP2 | Q9Y2D5 | 118097.305 |
| 1380 | CSNK1A1 | P48729 | 117817.8091 |
| 1381 | RAB11B | Q15907 | 117813.934 |
| 1382 | DDX23 | Q9BUQ8 | 117803.2287 |
| 1383 | MYO6 | Q9UM54 | 117735.3754 |
| 1384 | SRPRB | Q9Y5M8 | 117184.4947 |
| 1385 | ATOX1 | O00244 | 117134.0078 |
| 1386 | AP3M1 | Q9Y2T2 | 117100.6683 |
| 1387 | SERPINC1 | P01008 | 117027.4044 |
| 1388 | SNW1 | Q13573 | 116927.0828 |
| 1389 | LETM1 | O95202 | 116875.9 |
| 1390 | PPIC | P45877 | 116819.0562 |
| 1391 | SRP72 | O76094 | 116740.1969 |
| 1392 | AHCYL1 | O43865 | 116469.0788 |
| 1393 | SNRNP200 | O75643 | 116383.2171 |
| 1394 | RAP1A | P62834 | 116216.21 |
| 1395 | SLC25A13 | Q9UJS0 | 116143.7392 |
| 1396 | UFL1 | O94874 | 116030.6176 |
| 1397 | LSM1 | O15116 | 115655.9667 |
| 1398 | ICAM2 | P13598 | 115621.104 |
| 1399 | EIF3G | O75821 | 115563.9613 |
| 1400 | RBM39 | Q14498 | 115551.9427 |
| 1401 | MRPL11 | Q9Y3B7 | 115454.6244 |
| 1402 | NDUFA5 | Q16718 | 115370.326 |
| 1403 | RAC2 | P15153 | 114733.448 |
| 1404 | ATP6V1B2 | P21281 | 114450.0697 |
| 1405 | CHMP1A | Q9HD42 | 114379.7987 |
| 1406 | ERAP1 | Q9NZ08 | 114333.8974 |
| 1407 | NDUFA10 | O95299 | 114312.4817 |
| 1408 | PGP | A6NDG6 | 114282.0043 |
| 1409 | PARVA | Q9NVD7 | 114224.1161 |
| 1410 | COPS5 | Q92905 | 114115.0413 |
| 1411 | TPP1 | O14773 | 113966.1271 |
| 1412 | TIMM44 | O43615 | 113533.759 |
| 1413 | STAU1 | O95793 | 113532.0258 |
| 1414 | DUSP3 | P51452 | 113391.2801 |
| 1415 | ECHDC1 | Q9NTX5 | 112951.9976 |
| 1416 | MACF1 | Q9UPN3 | 112898.2685 |
| 1417 | TMEM165 | Q9HC07 | 112815.4644 |
| 1418 | PYCR2 | Q96C36 | 112585.3095 |
| 1419 | PPIL1 | Q9Y3C6 | 112575.68 |
| 1420 | IARS2 | Q9NSE4 | 112493.4695 |
| 1421 | MAP7D1 | Q3KQU3 | 112432.0486 |
| 1422 | G3BP2 | Q9UN86 | 112377.7485 |
| 1423 | TSPO | P30536 | 112324.2545 |
| 1424 | LPCAT2 | Q7L5N7 | 112265.0219 |
| 1425 | PFKM | P08237 | 112262.4846 |
| 1426 | POLR2E | P19388 | 112255.7058 |
| 1427 | STAT3 | P40763 | 112196.4678 |
| 1428 | SMC2 | O95347 | 112162.1793 |
| 1429 | SLC35B2 | Q8TB61 | 111854.0479 |
| 1430 | CUL4B | Q13620 | 111581.7345 |
| 1431 | SPR | P35270 | 111504.7075 |
| 1432 | TES | Q9UGI8 | 111504.5108 |
| 1433 | AP1G1 | O43747 | 111430.6185 |
| 1434 | CNP | P09543 | 111336.3869 |
| 1435 | MAP1S | Q66K74 | 111336.2396 |
| 1436 | KLC1 | Q07866 | 111248.7758 |
| 1437 | PPP5C | P53041 | 111163.6212 |
| 1438 | RAB10 | P61026 | 111154.8928 |
| 1439 | TIAL1 | Q01085 | 111095.9954 |
| 1440 | ATP5PB | P24539 | 110965.5128 |
| 1441 | ADK | P55263 | 110890.7332 |
| 1442 | POFUT1 | Q9H488 | 110770.7735 |
| 1443 | MYL9 | P24844 | 110586.9982 |
| 1444 | DDX3X | O00571 | 110532.8179 |
| 1445 | HPRT1 | P00492 | 110479.2238 |
| 1446 | DLD | P09622 | 110461.0577 |
| 1447 | IDE | P14735 | 110400.5184 |
| 1448 | POLR2L | P62875 | 110309.3567 |
| 1449 | RAB33B | Q9H082 | 110289.0698 |
| 1450 | TCERG1 | O14776 | 110288.3602 |
| 1451 | ATG3 | Q9NT62 | 109957.8759 |
| 1452 | CHMP4B | Q9H444 | 109815.9772 |
| 1453 | PRCP | P42785 | 109661.947 |
| 1454 | RPA2 | P15927 | 109637.4856 |
| 1455 | SQOR | Q9Y6N5 | 109611.1944 |
| 1456 | BLMH | Q13867 | 109463.164 |
| 1457 | HPCAL1 | P37235 | 109419.4997 |
| 1458 | NSDHL | Q15738 | 109416.476 |
| 1459 | RPA1 | P27694 | 109265.5366 |
| 1460 | UBE2E1 | P51965 | 109246.5406 |
| 1461 | BPNT1 | O95861 | 108816.0452 |
| 1462 | H1-10 | Q92522 | 108764.8701 |
| 1463 | L3HYPDH | Q96EM0 | 108753.1629 |
| 1464 | HGS | O14964 | 108720.5986 |
| 1465 | PTPN23 | Q9H3S7 | 108670.243 |
| 1466 | NEK7 | Q8TDX7 | 108540.5726 |
| 1467 | DAPK3 | O43293 | 108285.4104 |
| 1468 | PRPF8 | Q6P2Q9 | 108186.5689 |
| 1469 | PSMG2 | Q969U7 | 108185.5797 |
| 1470 | NDUFB9 | Q9Y6M9 | 108067.3713 |
| 1471 | MLEC | Q14165 | 108065.188 |
| 1472 | PEF1 | Q9UBV8 | 107804.8216 |
| 1473 | CST3 | P01034 | 107421.2483 |
| 1474 | CBFB | Q13951 | 107160.3946 |
| 1475 | CPSF7 | Q8N684 | 107145.0923 |
| 1476 | ITIH2 | P19823 | 106734.4945 |
| 1477 | C4orf3 | Q8WVX3 | 106543.82 |
| 1478 | STT3B | Q8TCJ2 | 106386.0766 |
| 1479 | LUZP1 | Q86V48 | 106281.7869 |
| 1480 | NUCB2 | P80303 | 106219.8188 |
| 1481 | POLR1D | P0DPB6 | 106218.5294 |
| 1482 | LAMC1 | P11047 | 106152.592 |
| 1483 | TIMM50 | Q3ZCQ8 | 106098.3857 |
| 1484 | SLC25A1 | P53007 | 106028.7618 |
| 1485 | AP3D1 | O14617 | 105715.8545 |
| 1486 | TM9SF4 | Q92544 | 105711.8708 |
| 1487 | GIPC1 | O14908 | 105461.6994 |
| 1488 | NOMO1 | Q15155 | 105443.1808 |
| 1489 | CENPB | P07199 | 105441.6941 |
| 1490 | MESD | Q14696 | 105384.4233 |
| 1491 | PAPSS1 | O43252 | 105195.3413 |
| 1492 | CYB5R1 | Q9UHQ9 | 105074.8772 |
| 1493 | PPP1R12A | O14974 | 105018.8206 |
| 1494 | UQCRFS1 | P47985 | 104768.3519 |
| 1495 | PPP3CA | Q08209 | 104744.7262 |
| 1496 | NME3 | Q13232 | 104651.5443 |
| 1497 | LBR | Q14739 | 104647.5371 |
| 1498 | DDX39B | Q13838 | 104562.8749 |
| 1499 | P3H4 | Q92791 | 104537.6222 |
| 1500 | NECTIN2 | Q92692 | 104508.4512 |
| 1501 | CIRBP | Q14011 | 104148.7327 |
| 1502 | DBT | P11182 | 104073.0104 |
| 1503 | NLN | Q9BYT8 | 104063.6166 |
| 1504 | TOMM70 | O94826 | 103975.7991 |
| 1505 | MRPL22 | Q9NWU5 | 103908.7389 |
| 1506 | EXOC7 | Q9UPT5 | 103904.9991 |
| 1507 | DNAJA1 | P31689 | 103902.6096 |
| 1508 | UBE2R2 | Q712K3 | 103727.944 |
| 1509 | FHL2 | Q14192 | 103430.3406 |
| 1510 | GMFB | P60983 | 103410.5225 |
| 1511 | DIPK1B | Q5VUD6 | 102764.2422 |
| 1512 | CCS | O14618 | 102572.885 |
| 1513 | SNRPF | P62306 | 102566.501 |
| 1514 | ARL8A | Q96BM9 | 102294.5314 |
| 1515 | GBA | P04062 | 102240.7797 |
| 1516 | DTD1 | Q8TEA8 | 102191.6922 |
| 1517 | RCN2 | Q14257 | 102159.3603 |
| 1518 | TOM1 | O60784 | 102148.9727 |
| 1519 | SLC41A3 | Q96GZ6 | 102109.9808 |
| 1520 | EBNA1BP2 | Q99848 | 102075.349 |
| 1521 | GAA | P10253 | 102039.4227 |
| 1522 | LSM4 | Q9Y4Z0 | 101982.9471 |
| 1523 | BET1 | O15155 | 101819.6805 |
| 1524 | SORBS2 | O94875 | 101693.4906 |
| 1525 | NAT10 | Q9H0A0 | 101585.2652 |
| 1526 | TTLL12 | Q14166 | 101571.4649 |
| 1527 | TCAF2 | A6NFQ2 | 101481.0949 |
| 1528 | LSM8 | O95777 | 101240.928 |
| 1529 | CLNS1A | P54105 | 101211.7331 |
| 1530 | NRP1 | O14786 | 100942.9087 |
| 1531 | P3H3 | Q8IVL6 | 100892.8386 |
| 1532 | RIC8A | Q9NPQ8 | 100792.2627 |
| 1533 | TOR1AIP1 | Q5JTV8 | 100700.027 |
| 1534 | TRIP10 | Q15642 | 100682.4627 |
| 1535 | EPN1 | Q9Y6I3 | 100357.9929 |
| 1536 | POLR2H | P52434 | 100357.2393 |
| 1537 | PYM1 | Q9BRP8 | 100178.1257 |
| 1538 | PLAA | Q9Y263 | 100157.9019 |
| 1539 | ARFIP1 | P53367 | 100054.6524 |
| 1540 | PAFAH1B2 | P68402 | 100034.2881 |
| 1541 | BCAR1 | P56945 | 99938.87181 |
| 1542 | MMRN1 | Q13201 | 99889.37603 |
| 1543 | IDH3A | P50213 | 99879.9789 |
| 1544 | UBXN1 | Q04323 | 99704.70024 |
| 1545 | MICOS13 | Q5XKP0 | 99656.5798 |
| 1546 | DDX46 | Q7L014 | 99592.67148 |
| 1547 | SEC23IP | Q9Y6Y8 | 99573.02286 |
| 1548 | CRYL1 | Q9Y2S2 | 99571.49724 |
| 1549 | EOGT | Q5NDL2 | 99502.96408 |
| 1550 | NCKAP1 | Q9Y2A7 | 99409.51205 |
| 1551 | HIBADH | P31937 | 99328.94793 |
| 1552 | SEPTIN10 | Q9P0V9 | 99293.45719 |
| 1553 | UBL4A | P11441 | 99235.10067 |
| 1554 | MRPS28 | Q9Y2Q9 | 99040.11564 |
| 1555 | POLR2K | P53803 | 99035.3 |
| 1556 | TMEM30A | Q9NV96 | 98912.85006 |
| 1557 | SQSTM1 | Q13501 | 98575.81694 |
| 1558 | TUBA4A | P68366 | 98575.57127 |
| 1559 | ACTB | P60709 | 98547.6771 |
| 1560 | UBA3 | Q8TBC4 | 98523.13062 |
| 1561 | FAM120A | Q9NZB2 | 98355.22942 |
| 1562 | ITGA3 | P26006 | 98072.27024 |
| 1563 | SCAMP2 | O15127 | 97985.52129 |
| 1564 | NDUFAF3 | Q9BU61 | 97694.14333 |
| 1565 | RAB3A | P20336 | 97658.06846 |
| 1566 | ZC3HAV1 | Q7Z2W4 | 97299.0563 |
| 1567 | ACYP1 | P07311 | 97052.14338 |
| 1568 | AGPS | O00116 | 97013.37081 |
| 1569 | CUL5 | Q93034 | 96978.90891 |
| 1570 | OXSR1 | O95747 | 96969.54281 |
| 1571 | POR | P16435 | 96958.96724 |
| 1572 | SMNDC1 | O75940 | 96745.985 |
| 1573 | RAB23 | Q9ULC3 | 96730.63212 |
| 1574 | GTF2I | P78347 | 96467.5057 |
| 1575 | FGF2 | P09038 | 96334.9211 |
| 1576 | DDX42 | Q86XP3 | 96322.18798 |
| 1577 | EMC2 | Q15006 | 96306.80647 |
| 1578 | GET4 | Q7L5D6 | 96170.16953 |
| 1579 | WDR46 | O15213 | 95945.97545 |
| 1580 | LZIC | Q8WZA0 | 95861.3612 |
| 1581 | CCDC47 | Q96A33 | 95784.08717 |
| 1582 | ETHE1 | O95571 | 95641.23857 |
| 1583 | NAPG | Q99747 | 95630.79129 |
| 1584 | ARFGAP1 | Q8N6T3 | 95426.439 |
| 1585 | NAA15 | Q9BXJ9 | 95379.98337 |
| 1586 | KPNA4 | O00629 | 95121.61582 |
| 1587 | STK25 | O00506 | 95052.0036 |
| 1588 | MMP1 | P03956 | 94987.19457 |
| 1589 | BAG6 | P46379 | 94857.22083 |
| 1590 | LAMTOR5 | O43504 | 94780.9288 |
| 1591 | VAMP2 | P63027 | 94756.6062 |
| 1592 | NPM3 | O75607 | 94723.6025 |
| 1593 | PRKAR2B | P31323 | 94577.91595 |
| 1594 | AK4 | P27144 | 94457.299 |
| 1595 | SLC16A3 | O15427 | 94196.32122 |
| 1596 | RBBP4 | Q09028 | 94161.94088 |
| 1597 | C1GALT1 | Q9NS00 | 94004.46788 |
| 1598 | TNKS1BP1 | Q9C0C2 | 93988.03977 |
| 1599 | SAMM50 | Q9Y512 | 93921.84411 |
| 1600 | PGM2L1 | Q6PCE3 | 93822.63688 |
| 1601 | ME2 | P23368 | 93666.3918 |
| 1602 | FKBP8 | Q14318 | 93379.11083 |
| 1603 | ACAD9 | Q9H845 | 93295.17525 |
| 1604 | PRPF40A | O75400 | 93144.95077 |
| 1605 | CDC5L | Q99459 | 93076.92792 |
| 1606 | ECI1 | P42126 | 93062.787 |
| 1607 | SULT1A1 | P50225 | 92993.59324 |
| 1608 | HACD3 | Q9P035 | 92924.54175 |
| 1609 | DNAJB1 | P25685 | 92568.4644 |
| 1610 | METTL26 | Q96S19 | 92536.70143 |
| 1611 | HNRNPUL1 | Q9BUJ2 | 92515.92267 |
| 1612 | TIMM9 | Q9Y5J7 | 92067.355 |
| 1613 | NDUFV1 | P49821 | 91889.27736 |
| 1614 | YIF1B | Q5BJH7 | 91776.12322 |
| 1615 | ICAM1 | P05362 | 91712.73729 |
| 1616 | DAZAP1 | Q96EP5 | 91530.9145 |
| 1617 | SUN2 | Q9UH99 | 91397.44337 |
| 1618 | RBBP7 | Q16576 | 91117.55761 |
| 1619 | FHL1 | Q13642 | 90944.90765 |
| 1620 | PPIL4 | Q8WUA2 | 90914.96367 |
| 1621 | CD2AP | Q9Y5K6 | 90913.52873 |
| 1622 | BCL2L13 | Q9BXK5 | 90864.76419 |
| 1623 | TRAPPC5 | Q8IUR0 | 90832.49336 |
| 1624 | STXBP3 | O00186 | 90825.55342 |
| 1625 | TMED2 | Q15363 | 90752.506 |
| 1626 | MYEF2 | Q9P2K5 | 90720.9937 |
| 1627 | ECI2 | O75521 | 90682.54939 |
| 1628 | ERAP2 | Q6P179 | 90643.60857 |
| 1629 | MRPS7 | Q9Y2R9 | 90642.97177 |
| 1630 | NIPSNAP2 | O75323 | 90588.24633 |
| 1631 | GNAI3 | P08754 | 90479.215 |
| 1632 | PAWR | Q96IZ0 | 90377.62718 |
| 1633 | COMMD5 | Q9GZQ3 | 90344.75083 |
| 1634 | FBLL1 | A6NHQ2 | 90316.66316 |
| 1635 | LSM12 | Q3MHD2 | 90305.2885 |
| 1636 | FAH | P16930 | 90275.35905 |
| 1637 | MIEN1 | Q9BRT3 | 90207.11675 |
| 1638 | STK24 | Q9Y6E0 | 90189.9648 |
| 1639 | SNX9 | Q9Y5X1 | 90120.55257 |
| 1640 | LZTFL1 | Q9NQ48 | 90089.88913 |
| 1641 | NPLOC4 | Q8TAT6 | 90001.89963 |
| 1642 | MYO1E | Q12965 | 89998.63107 |
| 1643 | COMMD3 | Q9UBI1 | 89994.8562 |
| 1644 | EXOSC3 | Q9NQT5 | 89978.35292 |
| 1645 | RABL6 | Q3YEC7 | 89813.2345 |
| 1646 | NRCAM | Q92823 | 89670.89988 |
| 1647 | NPC2 | P61916 | 89622.2471 |
| 1648 | STAM2 | O75886 | 89506.32723 |
| 1649 | AGFG1 | P52594 | 89470.08301 |
| 1650 | THYN1 | Q9P016 | 89417.82906 |
| 1651 | TRIP13 | Q15645 | 89302.90204 |
| 1652 | CTSC | P53634 | 89291.71547 |
| 1653 | PPP1R18 | Q6NYC8 | 89235.99846 |
| 1654 | COL18A1 | P39060 | 89039.97833 |
| 1655 | BCAP29 | Q9UHQ4 | 88942.36768 |
| 1656 | MARCKSL1 | P49006 | 88854.37949 |
| 1657 | MTMR2 | Q13614 | 88790.24288 |
| 1658 | DRG2 | P55039 | 88602.42748 |
| 1659 | DCTD | P32321 | 88578.34636 |
| 1660 | SH3BP2 | P78314 | 88439.90404 |
| 1661 | PQBP1 | O60828 | 88393.468 |
| 1662 | NTPCR | Q9BSD7 | 88332.85336 |
| 1663 | IMPDH1 | P20839 | 88276.06091 |
| 1664 | GM2A | P17900 | 88256.196 |
| 1665 | CYB5A | P00167 | 87927.8599 |
| 1666 | MRPL15 | Q9P015 | 87844.75747 |
| 1667 | RPS4Y1 | P22090 | 87823.75179 |
| 1668 | ATXN10 | Q9UBB4 | 87746.61975 |
| 1669 | NOP16 | Q9Y3C1 | 87705.4145 |
| 1670 | ASL | P04424 | 87651.26188 |
| 1671 | EIF4A2 | Q14240 | 87596.90135 |
| 1672 | COPS3 | Q9UNS2 | 87327.49446 |
| 1673 | TMEM230 | Q96A57 | 87298.05157 |
| 1674 | SAR1B | Q9Y6B6 | 87169.13718 |
| 1675 | BNIP1 | Q12981 | 87078.37071 |
| 1676 | DPP7 | Q9UHL4 | 87000.15568 |
| 1677 | PSMG3 | Q9BT73 | 86992.02467 |
| 1678 | PHF5A | Q7RTV0 | 86948.94125 |
| 1679 | UQCRB | P14927 | 86915.45433 |
| 1680 | MRPS31 | Q92665 | 86893.91532 |
| 1681 | HOOK3 | Q86VS8 | 86772.34561 |
| 1682 | AMDHD2 | Q9Y303 | 86771.061 |
| 1683 | NAPRT | Q6XQN6 | 86680.77604 |
| 1684 | MYO1D | O94832 | 86661.75476 |
| 1685 | IER3IP1 | Q9Y5U9 | 86654.9925 |
| 1686 | MAGT1 | Q9H0U3 | 86644.19333 |
| 1687 | IGBP1 | P78318 | 86641.71753 |
| 1688 | LSS | P48449 | 86237.11098 |
| 1689 | CYFIP1 | Q7L576 | 86199.87311 |
| 1690 | XPOT | O43592 | 86174.13943 |
| 1691 | VPS4A | Q9UN37 | 86109.79532 |
| 1692 | SF3A2 | Q15428 | 86063.57607 |
| 1693 | ARFGAP3 | Q9NP61 | 86009.30603 |
| 1694 | AIP | O00170 | 85813.53259 |
| 1695 | EIF2B5 | Q13144 | 85776.93857 |
| 1696 | DKC1 | O60832 | 85747.55052 |
| 1697 | NUP43 | Q8NFH3 | 85704.88617 |
| 1698 | HAS1 | Q92839 | 85459.84914 |
| 1699 | TMEM214 | Q6NUQ4 | 85331.97592 |
| 1700 | SLC2A1 | P11166 | 85096.80976 |
| 1701 | GLS | O94925 | 85086.70788 |
| 1702 | BCL10 | O95999 | 85045.7749 |
| 1703 | CYRIB | Q9NUQ9 | 84954.40086 |
| 1704 | APOM | O95445 | 84753.153 |
| 1705 | LUC7L2 | Q9Y383 | 84740.91628 |
| 1706 | FCGRT | P55899 | 84539.67207 |
| 1707 | SELENOH | Q8IZQ5 | 84470.06671 |
| 1708 | TUBB2A | Q13885 | 84281.38735 |
| 1709 | UMPS | P11172 | 84201.40836 |
| 1710 | GJA1 | P17302 | 84107.98215 |
| 1711 | DDX27 | Q96GQ7 | 84029.84938 |
| 1712 | CDK5RAP3 | Q96JB5 | 83974.06077 |
| 1713 | RMDN1 | Q96DB5 | 83965.49818 |
| 1714 | TPRG1L | Q5T0D9 | 83929.16118 |
| 1715 | SRRT | Q9BXP5 | 83927.73825 |
| 1716 | TXLNA | P40222 | 83923.98276 |
| 1717 | DYSF | O75923 | 83874.50256 |
| 1718 | IVD | P26440 | 83798.57243 |
| 1719 | RPL7L1 | Q6DKI1 | 83745.50893 |
| 1720 | ZW10 | O43264 | 83667.73517 |
| 1721 | PTRHD1 | Q6GMV3 | 83634.84125 |
| 1722 | HIP1R | O75146 | 83378.20025 |
| 1723 | PPID | Q08752 | 83371.20346 |
| 1724 | ATP6V1D | Q9Y5K8 | 83287.37715 |
| 1725 | RDH11 | Q8TC12 | 83224.36233 |
| 1726 | SLC30A7 | Q8NEW0 | 83160.62257 |
| 1727 | MRPS17 | Q9Y2R5 | 83080.684 |
| 1728 | NEU1 | Q99519 | 83072.1635 |
| 1729 | KRT2 | P35908 | 83045.52433 |
| 1730 | GALNT2 | Q10471 | 82899.51276 |
| 1731 | ALDH6A1 | Q02252 | 82762.92768 |
| 1732 | NCK1 | P16333 | 82722.35036 |
| 1733 | NDUFS2 | O75306 | 82674.9362 |
| 1734 | ENDOD1 | O94919 | 82622.27984 |
| 1735 | ECPAS | Q5VYK3 | 82616.88015 |
| 1736 | RBMX | P38159 | 82553.29104 |
| 1737 | SMC3 | Q9UQE7 | 82498.39905 |
| 1738 | CARHSP1 | Q9Y2V2 | 82476.949 |
| 1739 | MFNG | O00587 | 82362.99105 |
| 1740 | PURA | Q00577 | 82321.429 |
| 1741 | OXCT1 | P55809 | 82205.90396 |
| 1742 | DECR2 | Q9NUI1 | 81809.04689 |
| 1743 | TJP1 | Q07157 | 81757.18276 |
| 1744 | FLII | Q13045 | 81673.82634 |
| 1745 | CBR3 | O75828 | 81666.6385 |
| 1746 | MAPRE2 | Q15555 | 81634.52912 |
| 1747 | CORO7 | P57737 | 81628.41171 |
| 1748 | WASF2 | Q9Y6W5 | 81575.987 |
| 1749 | DGKA | P23743 | 81503.29826 |
| 1750 | ATXN7L3B | Q96GX2 | 81498.5742 |
| 1751 | CLPP | Q16740 | 81481.40056 |
| 1752 | TMED7 | Q9Y3B3 | 81404.87238 |
| 1753 | STAM | Q92783 | 81317.75535 |
| 1754 | SUPT16H | Q9Y5B9 | 81285.69077 |
| 1755 | NDUFA6 | P56556 | 81239.65475 |
| 1756 | EIF2B2 | P49770 | 81124.72724 |
| 1757 | ATP6AP2 | O75787 | 81067.71727 |
| 1758 | STING1 | Q86WV6 | 80999.71738 |
| 1759 | UBLCP1 | Q8WVY7 | 80998.43488 |
| 1760 | STX5 | Q13190 | 80962.59872 |
| 1761 | CHORDC1 | Q9UHD1 | 80868.60228 |
| 1762 | ATP6AP1 | Q15904 | 80643.37832 |
| 1763 | PPP4R1 | Q8TF05 | 80599.64343 |
| 1764 | TRADD | Q15628 | 80538.45392 |
| 1765 | HLA-C | P10321 | 80372.72625 |
| 1766 | RAB8A | P61006 | 80335.57 |
| 1767 | EMC3 | Q9P0I2 | 80293.69543 |
| 1768 | TRIM25 | Q14258 | 80073.44632 |
| 1769 | WDR12 | Q9GZL7 | 79975.31078 |
| 1770 | CBX5 | P45973 | 79900.41827 |
| 1771 | SPCS2 | Q15005 | 79855.25045 |
| 1772 | CMAS | Q8NFW8 | 79771.09861 |
| 1773 | SEC63 | Q9UGP8 | 79736.25954 |
| 1774 | ANP32E | Q9BTT0 | 79699.75738 |
| 1775 | GIMAP4 | Q9NUV9 | 79683.13135 |
| 1776 | GNL3 | Q9BVP2 | 79615.99146 |
| 1777 | PSMD10 | O75832 | 79527.1439 |
| 1778 | EXOSC4 | Q9NPD3 | 79512.6013 |
| 1779 | GPD2 | P43304 | 79462.85007 |
| 1780 | DCPS | Q96C86 | 79441.95375 |
| 1781 | ADAR | P55265 | 79431.22268 |
| 1782 | MPZL1 | O95297 | 79406.27667 |
| 1783 | DERL2 | Q9GZP9 | 79404.2155 |
| 1784 | LAMTOR3 | Q9UHA4 | 79200.08167 |
| 1785 | PNPT1 | Q8TCS8 | 79195.59195 |
| 1786 | PKN2 | Q16513 | 79151.42322 |
| 1787 | GNPNAT1 | Q96EK6 | 79101.8695 |
| 1788 | FAM177A1 | Q8N128 | 79079.35292 |
| 1789 | ADA | P00813 | 78936.4487 |
| 1790 | PDXDC1 | Q6P996 | 78929.15722 |
| 1791 | LYPLAL1 | Q5VWZ2 | 78695.12869 |
| 1792 | WNK1 | Q9H4A3 | 78670.15987 |
| 1793 | AMPD2 | Q01433 | 78646.45488 |
| 1794 | MCM7 | P33993 | 78606.88108 |
| 1795 | AGK | Q53H12 | 78594.88409 |
| 1796 | MRPL45 | Q9BRJ2 | 78357.215 |
| 1797 | AP1S1 | P61966 | 78325.1667 |
| 1798 | TOMM34 | Q15785 | 78323.37894 |
| 1799 | ST13 | P50502 | 78257.54836 |
| 1800 | LAMP2 | P13473 | 78124.50289 |
| 1801 | TOLLIP | Q9H0E2 | 78110.18607 |
| 1802 | MIX23 | Q4VC31 | 77948.03163 |
| 1803 | BDH2 | Q9BUT1 | 77711.28979 |
| 1804 | COMMD10 | Q9Y6G5 | 77677.4763 |
| 1805 | TRMT112 | Q9UI30 | 77562.31157 |
| 1806 | CHRAC1 | Q9NRG0 | 77427.87367 |
| 1807 | CHMP1B | Q7LBR1 | 77418.10375 |
| 1808 | EBP | Q15125 | 77398.55117 |
| 1809 | FAF2 | Q96CS3 | 77358.75058 |
| 1810 | H1-0 | P07305 | 77358.25277 |
| 1811 | TOR1A | O14656 | 77329.92494 |
| 1812 | PTGES3 | Q15185 | 77248.564 |
| 1813 | RAB27A | P51159 | 77176.24336 |
| 1814 | TMED4 | Q7Z7H5 | 77031.1335 |
| 1815 | PLCB3 | Q01970 | 76912.55822 |
| 1816 | PRKAA1 | Q13131 | 76911.17335 |
| 1817 | MRPL23 | Q16540 | 76903.65 |
| 1818 | SCPEP1 | Q9HB40 | 76888.95653 |
| 1819 | NCBP2 | P52298 | 76848.01556 |
| 1820 | EIF2B3 | Q9NR50 | 76845.1941 |
| 1821 | PPP1R11 | O60927 | 76648.96478 |
| 1822 | SEPHS1 | P49903 | 76581.00831 |
| 1823 | SPAG7 | O75391 | 76498.42073 |
| 1824 | DHRS4 | Q9BTZ2 | 76402.49357 |
| 1825 | SART1 | O43290 | 76377.36657 |
| 1826 | BCAT2 | O15382 | 76335.47761 |
| 1827 | PAIP1 | Q9H074 | 76274.00284 |
| 1828 | GFM1 | Q96RP9 | 76273.48136 |
| 1829 | NCOA5 | Q9HCD5 | 76094.67229 |
| 1830 | RAB3B | P20337 | 76083.37482 |
| 1831 | MYO5A | Q9Y4I1 | 76079.88128 |
| 1832 | SH3BP4 | Q9P0V3 | 76045.96376 |
| 1833 | MRPL37 | Q9BZE1 | 76035.87014 |
| 1834 | CCN1 | O00622 | 75889.80376 |
| 1835 | DCXR | Q7Z4W1 | 75851.64127 |
| 1836 | ARMCX3 | Q9UH62 | 75843.3499 |
| 1837 | C7orf50 | Q9BRJ6 | 75838.71578 |
| 1838 | MGAT1 | P26572 | 75713.55479 |
| 1839 | CKAP5 | Q14008 | 75706.16979 |
| 1840 | C11orf98 | E9PRG8 | 75579.67633 |
| 1841 | NDUFA11 | Q86Y39 | 75549.22614 |
| 1842 | DNAJC13 | O75165 | 75489.4798 |
| 1843 | DESI1 | Q6ICB0 | 75459.194 |
| 1844 | CEPT1 | Q9Y6K0 | 75380.84545 |
| 1845 | PML | P29590 | 75368.1548 |
| 1846 | TMEM11 | P17152 | 75287.864 |
| 1847 | TMBIM6 | P55061 | 75223.98325 |
| 1848 | COPS7A | Q9UBW8 | 75090.92588 |
| 1849 | NDUFS4 | O43181 | 75078.76111 |
| 1850 | PPP2R5D | Q14738 | 75066.52724 |
| 1851 | SYNPO | Q8N3V7 | 74932.02602 |
| 1852 | NUFIP2 | Q7Z417 | 74919.401 |
| 1853 | OXA1L | Q15070 | 74806.85186 |
| 1854 | EIF5B | O60841 | 74802.45193 |
| 1855 | DNAJC10 | Q8IXB1 | 74802.0588 |
| 1856 | PTPRB | P23467 | 74794.32321 |
| 1857 | RTCA | O00442 | 74773.699 |
| 1858 | ATN1 | P54259 | 74753.16157 |
| 1859 | ENOPH1 | Q9UHY7 | 74738.877 |
| 1860 | RPRD1B | Q9NQG5 | 74703.65338 |
| 1861 | CCDC22 | O60826 | 74695.52419 |
| 1862 | RABGGTA | Q92696 | 74683.87847 |
| 1863 | PRKRA | O75569 | 74679.48797 |
| 1864 | SEPTIN8 | Q92599 | 74674.78419 |
| 1865 | MRPL24 | Q96A35 | 74664.57786 |
| 1866 | ARFGAP2 | Q8N6H7 | 74638.53069 |
| 1867 | UTRN | P46939 | 74587.89429 |
| 1868 | NOVA2 | Q9UNW9 | 74482.64268 |
| 1869 | CD47 | Q08722 | 74358.11667 |
| 1870 | EPS15L1 | Q9UBC2 | 74354.38106 |
| 1871 | CDK1 | P06493 | 74328.80947 |
| 1872 | PPM1F | P49593 | 74107.06342 |
| 1873 | GABARAPL2 | P60520 | 74092.00338 |
| 1874 | PIN1 | Q13526 | 74081.45167 |
| 1875 | PPP6C | O00743 | 73979.71513 |
| 1876 | SAR1A | Q9NR31 | 73933.82736 |
| 1877 | CHP1 | Q99653 | 73928.25078 |
| 1878 | MRPS23 | Q9Y3D9 | 73884.04492 |
| 1879 | SRSF10 | O75494 | 73695.2109 |
| 1880 | FBXO22 | Q8NEZ5 | 73658.72384 |
| 1881 | PMPCA | Q10713 | 73636.87797 |
| 1882 | RAB12 | Q6IQ22 | 73636.07211 |
| 1883 | SOD2 | P04179 | 73627.55417 |
| 1884 | ATP6V1C1 | P21283 | 73589.74064 |
| 1885 | SETD7 | Q8WTS6 | 73517.6645 |
| 1886 | CTSA | P10619 | 73477.033 |
| 1887 | CTTNBP2NL | Q9P2B4 | 73432.90119 |
| 1888 | ABHD10 | Q9NUJ1 | 73274.681 |
| 1889 | PBXIP1 | Q96AQ6 | 73269.713 |
| 1890 | HMGN2 | P05204 | 73142.34943 |
| 1891 | TIMM23 | O14925 | 73129.35178 |
| 1892 | BCL2L1 | Q07817 | 73127.91778 |
| 1893 | FHOD1 | Q9Y613 | 72925.9125 |
| 1894 | MRPL17 | Q9NRX2 | 72874.40192 |
| 1895 | TWF1 | Q12792 | 72817.43314 |
| 1896 | RAB43 | Q86YS6 | 72754.13724 |
| 1897 | LARP1 | Q6PKG0 | 72718.68945 |
| 1898 | MAPK14 | Q16539 | 72685.13879 |
| 1899 | NDUFB5 | O43674 | 72467.45956 |
| 1900 | ACSL3 | O95573 | 72406.94859 |
| 1901 | THRAP3 | Q9Y2W1 | 72318.3174 |
| 1902 | NDUFA9 | Q16795 | 72297.8356 |
| 1903 | KPNA6 | O60684 | 72163.50813 |
| 1904 | NRDC | O43847 | 72133.71103 |
| 1905 | GADD45GIP1 | Q8TAE8 | 72096.77991 |
| 1906 | YIPF4 | Q9BSR8 | 71836.40625 |
| 1907 | NOP10 | Q9NPE3 | 71813.00967 |
| 1908 | NFU1 | Q9UMS0 | 71800.6627 |
| 1909 | NENF | Q9UMX5 | 71423.91636 |
| 1910 | BAP18 | Q8IXM2 | 71366.78688 |
| 1911 | ESYT2 | A0FGR8 | 71259.70813 |
| 1912 | EXOC5 | O00471 | 71258.00064 |
| 1913 | GCDH | Q92947 | 71245.78995 |
| 1914 | SDHA | P31040 | 71222.81558 |
| 1915 | EMG1 | Q92979 | 71167.1142 |
| 1916 | LGMN | Q99538 | 71140.90715 |
| 1917 | LANCL1 | O43813 | 71034.17247 |
| 1918 | SON | P18583 | 71033.07288 |
| 1919 | STIM1 | Q13586 | 70960.472 |
| 1920 | SNRPA | P09012 | 70935.31336 |
| 1921 | COX5A | P20674 | 70916.539 |
| 1922 | THOP1 | P52888 | 70871.02639 |
| 1923 | LEMD2 | Q8NC56 | 70772.3667 |
| 1924 | ALDH3A2 | P51648 | 70710.18502 |
| 1925 | DIAPH1 | O60610 | 70697.90289 |
| 1926 | SLC25A22 | Q9H936 | 70659.85697 |
| 1927 | CLPX | O76031 | 70634.54338 |
| 1928 | NEXN | Q0ZGT2 | 70628.19972 |
| 1929 | RAB3GAP2 | Q9H2M9 | 70621.84389 |
| 1930 | DAP3 | P51398 | 70611.57318 |
| 1931 | MRPS26 | Q9BYN8 | 70532.18308 |
| 1932 | NUP133 | Q8WUM0 | 70519.61388 |
| 1933 | WASHC3 | Q9Y3C0 | 70477.59367 |
| 1934 | SLC29A1 | Q99808 | 70460.86894 |
| 1935 | NDRG3 | Q9UGV2 | 70459.06044 |
| 1936 | KATNAL2 | Q8IYT4 | 70398.36667 |
| 1937 | HS1BP3 | Q53T59 | 69996.50375 |
| 1938 | GLG1 | Q92896 | 69951.7016 |
| 1939 | SLK | Q9H2G2 | 69936.665 |
| 1940 | PNPO | Q9NVS9 | 69888.00892 |
| 1941 | LIMS1 | P48059 | 69869.13 |
| 1942 | PTRH2 | Q9Y3E5 | 69838.00888 |
| 1943 | NACA | E9PAV3 | 69818.84805 |
| 1944 | NRBP1 | Q9UHY1 | 69795.99541 |
| 1945 | PLIN2 | Q99541 | 69787.38912 |
| 1946 | FAHD2A | Q96GK7 | 69731.26882 |
| 1947 | DDX47 | Q9H0S4 | 69622.28645 |
| 1948 | PACS1 | Q6VY07 | 69524.43869 |
| 1949 | VPS28 | Q9UK41 | 69452.41738 |
| 1950 | PLBD2 | Q8NHP8 | 69399.50469 |
| 1951 | APOL2 | Q9BQE5 | 69260.19989 |
| 1952 | PIK3C2A | O00443 | 69187.6395 |
| 1953 | NUDCD1 | Q96RS6 | 69160.7666 |
| 1954 | PARP4 | Q9UKK3 | 69146.66773 |
| 1955 | MRPL44 | Q9H9J2 | 69122.73832 |
| 1956 | OTUD6B | Q8N6M0 | 69002.8046 |
| 1957 | PRR15 | Q8IV56 | 68870.169 |
| 1958 | CAMK1 | Q14012 | 68681.68506 |
| 1959 | FTL | P02792 | 68667.26122 |
| 1960 | FAM241A | Q8N8J7 | 68622.7331 |
| 1961 | TBK1 | Q9UHD2 | 68622.33343 |
| 1962 | MRPS21 | P82921 | 68554.558 |
| 1963 | NFS1 | Q9Y697 | 68478.05625 |
| 1964 | RCC2 | Q9P258 | 68332.06907 |
| 1965 | PLXNA2 | O75051 | 68281.31663 |
| 1966 | SEC24A | O95486 | 68253.51874 |
| 1967 | NUP214 | P35658 | 68123.9351 |
| 1968 | PREP | P48147 | 68070.68187 |
| 1969 | SNAPIN | O95295 | 68055.6505 |
| 1970 | HUWE1 | Q7Z6Z7 | 68022.56843 |
| 1971 | TIPRL | O75663 | 68010.09919 |
| 1972 | MRPL43 | Q8N983 | 67916.50436 |
| 1973 | CPD | O75976 | 67901.03644 |
| 1974 | LIMD2 | Q9BT23 | 67704.15676 |
| 1975 | MRPL21 | Q7Z2W9 | 67692.81233 |
| 1976 | SF3B6 | Q9Y3B4 | 67632.715 |
| 1977 | CRYZ | Q08257 | 67597.82562 |
| 1978 | PDLIM3 | Q53GG5 | 67414.96041 |
| 1979 | FEN1 | P39748 | 67395.11461 |
| 1980 | ARL6IP6 | Q8N6S5 | 67381.82857 |
| 1981 | IK | Q13123 | 67368.94659 |
| 1982 | PFAS | O15067 | 67349.58773 |
| 1983 | CHTOP | Q9Y3Y2 | 67304.32211 |
| 1984 | EXOC3 | O60645 | 67273.30775 |
| 1985 | WDR44 | Q5JSH3 | 67255.62943 |
| 1986 | IPO4 | Q8TEX9 | 67232.03974 |
| 1987 | PITHD1 | Q9GZP4 | 67157.43656 |
| 1988 | GAR1 | Q9NY12 | 67128.19567 |
| 1989 | OGDH | Q02218 | 67105.61959 |
| 1990 | RAE1 | P78406 | 67061.17438 |
| 1991 | ELOVL1 | Q9BW60 | 67020.34725 |
| 1992 | MBOAT7 | Q96N66 | 67017.09233 |
| 1993 | TCIRG1 | Q13488 | 66961.49584 |
| 1994 | SRGN | P10124 | 66945.2475 |
| 1995 | CNOT9 | Q92600 | 66925.16456 |
| 1996 | MAGOHB | Q96A72 | 66848.106 |
| 1997 | NXF1 | Q9UBU9 | 66841.99093 |
| 1998 | CCDC6 | Q16204 | 66666.31024 |
| 1999 | PLEKHO2 | Q8TD55 | 66633.54836 |
| 2000 | CNTNAP1 | P78357 | 66581.87663 |
| 2001 | CDKN2A | P42771 | 66574.1315 |
| 2002 | ALDH16A1 | Q8IZ83 | 66554.3385 |
| 2003 | XRN2 | Q9H0D6 | 66509.09935 |
| 2004 | INF2 | Q27J81 | 66434.46422 |
| 2005 | GHITM | Q9H3K2 | 66408.29879 |
| 2006 | NDUFAF2 | Q8N183 | 66407.358 |
| 2007 | RBM17 | Q96I25 | 66347.00392 |
| 2008 | PPIL3 | Q9H2H8 | 66300.82473 |
| 2009 | STUB1 | Q9UNE7 | 66298.70995 |
| 2010 | RRAGC | Q9HB90 | 66283.96486 |
| 2011 | RAB9A | P51151 | 66226.19083 |
| 2012 | TSNAX | Q99598 | 66163.57206 |
| 2013 | ALDH1A3 | P47895 | 66106.77552 |
| 2014 | RUFY1 | Q96T51 | 66037.5931 |
| 2015 | PMVK | Q15126 | 66030.72915 |
| 2016 | PXDN | Q92626 | 66026.57604 |
| 2017 | SLC25A20 | O43772 | 66019.40826 |
| 2018 | MAN2A1 | Q16706 | 65991.48883 |
| 2019 | NIPSNAP3A | Q9UFN0 | 65981.78314 |
| 2020 | EPS15 | P42566 | 65979.47534 |
| 2021 | FAR1 | Q8WVX9 | 65928.12186 |
| 2022 | FKBP7 | Q9Y680 | 65876.18725 |
| 2023 | RRS1 | Q15050 | 65853.04815 |
| 2024 | NDUFA4 | O00483 | 65812.908 |
| 2025 | VKORC1L1 | Q8N0U8 | 65715.32186 |
| 2026 | CELF1 | Q92879 | 65584.27731 |
| 2027 | PMPCB | O75439 | 65579.46011 |
| 2028 | RALB | P11234 | 65490.96067 |
| 2029 | RHOC | P08134 | 65488.55425 |
| 2030 | FBLN1 | P23142 | 65413.2177 |
| 2031 | TNS1 | Q9HBL0 | 65371.59682 |
| 2032 | ARPP19 | P56211 | 65349.48417 |
| 2033 | TMEM65 | Q6PI78 | 65158.0106 |
| 2034 | BCAS2 | O75934 | 65082.90115 |
| 2035 | LRRFIP1 | Q32MZ4 | 65078.2625 |
| 2036 | PCDH10 | Q9P2E7 | 65077.01007 |
| 2037 | RBMS2 | Q15434 | 65076.16725 |
| 2038 | BRK1 | Q8WUW1 | 65051.53 |
| 2039 | MAP2 | P11137 | 65049.1656 |
| 2040 | MCM2 | P49736 | 65044.11491 |
| 2041 | DUT | P33316 | 64983.89087 |
| 2042 | VTI1B | Q9UEU0 | 64863.3775 |
| 2043 | VPS4B | O75351 | 64815.96713 |
| 2044 | NUP98 | P52948 | 64811.2757 |
| 2045 | DOCK9 | Q9BZ29 | 64761.56582 |
| 2046 | PPP1R8 | Q12972 | 64749.82658 |
| 2047 | TGFB1I1 | O43294 | 64658.60758 |
| 2048 | UBE2V1 | Q13404 | 64647.4056 |
| 2049 | SYAP1 | Q96A49 | 64467.45075 |
| 2050 | RRM1 | P23921 | 64450.06943 |
| 2051 | SRRM2 | Q9UQ35 | 64417.38867 |
| 2052 | MAVS | Q7Z434 | 64295.0292 |
| 2053 | DHRS7 | Q9Y394 | 64230.05711 |
| 2054 | NIPSNAP1 | Q9BPW8 | 64226.68207 |
| 2055 | NMT1 | P30419 | 64123.57359 |
| 2056 | MRPS10 | P82664 | 64116.78775 |
| 2057 | HSPA1L | P34931 | 64102.93291 |
| 2058 | QTRT1 | Q9BXR0 | 64040.4805 |
| 2059 | EMC1 | Q8N766 | 63958.3014 |
| 2060 | RBM22 | Q9NW64 | 63854.81649 |
| 2061 | FAM50A | Q14320 | 63697.54288 |
| 2062 | PDCL3 | Q9H2J4 | 63685.95643 |
| 2063 | GMFG | O60234 | 63660.176 |
| 2064 | MPG | P29372 | 63465.05429 |
| 2065 | NT5DC2 | Q9H857 | 63380.4476 |
| 2066 | FAM98A | Q8NCA5 | 63316.89417 |
| 2067 | NDUFA3 | O95167 | 63291.4685 |
| 2068 | MICAL1 | Q8TDZ2 | 63269.26704 |
| 2069 | TMEM205 | Q6UW68 | 63240.31133 |
| 2070 | STRN3 | Q13033 | 63201.94176 |
| 2071 | SLC25A12 | O75746 | 63092.32514 |
| 2072 | PPCS | Q9HAB8 | 63083.73347 |
| 2073 | WDR18 | Q9BV38 | 62956.2347 |
| 2074 | NID1 | P14543 | 62942.55245 |
| 2075 | TP53RK | Q96S44 | 62895.2212 |
| 2076 | TMED1 | Q13445 | 62832.78671 |
| 2077 | RAB4A | P20338 | 62812.69886 |
| 2078 | THEM6 | Q8WUY1 | 62795.27357 |
| 2079 | ARHGAP17 | Q68EM7 | 62787.10355 |
| 2080 | CARS2 | Q9HA77 | 62703.10604 |
| 2081 | PDLIM2 | Q96JY6 | 62668.19032 |
| 2082 | GMPPA | Q96IJ6 | 62594.50756 |
| 2083 | NUDCD2 | Q8WVJ2 | 62492.759 |
| 2084 | SMC1A | Q14683 | 62480.34157 |
| 2085 | SMAP | O00193 | 62414.1481 |
| 2086 | FBXO42 | Q6P3S6 | 62379.35677 |
| 2087 | PURB | Q96QR8 | 62350.91575 |
| 2088 | GNB1 | P62873 | 62308.83647 |
| 2089 | ERG | P11308 | 62117.39039 |
| 2090 | BCLAF1 | Q9NYF8 | 62078.93451 |
| 2091 | SMAP1 | Q8IYB5 | 62016.3939 |
| 2092 | DNPEP | Q9ULA0 | 61950.23492 |
| 2093 | TOR1B | O14657 | 61861.47412 |
| 2094 | LOXL2 | Q9Y4K0 | 61774.88945 |
| 2095 | CDC42EP2 | O14613 | 61757.58371 |
| 2096 | UBA5 | Q9GZZ9 | 61698.26731 |
| 2097 | RABGGTB | P53611 | 61614.73827 |
| 2098 | SELENOS | Q9BQE4 | 61558.00331 |
| 2099 | MCRIP1 | C9JLW8 | 61461.76179 |
| 2100 | DARS2 | Q6PI48 | 61460.24856 |
| 2101 | ESAM | Q96AP7 | 61434.52572 |
| 2102 | STMN2 | Q93045 | 61340.88 |
| 2103 | COMMD9 | Q9P000 | 61314.5575 |
| 2104 | TPP2 | P29144 | 61293.88783 |
| 2105 | MYBBP1A | Q9BQG0 | 61268.55753 |
| 2106 | EXOC8 | Q8IYI6 | 61153.81799 |
| 2107 | GTPBP4 | Q9BZE4 | 61142.21925 |
| 2108 | USP7 | Q93009 | 61122.55342 |
| 2109 | MOB2 | Q70IA6 | 61117.87273 |
| 2110 | MPI | P34949 | 61117.54073 |
| 2111 | EIF2B1 | Q14232 | 61103.08259 |
| 2112 | MCM3 | P25205 | 61077.95035 |
| 2113 | TNFSF4 | P23510 | 61021.07456 |
| 2114 | ACSL4 | O60488 | 60978.53792 |
| 2115 | MTA2 | O94776 | 60883.0931 |
| 2116 | BCR | P11274 | 60869.12942 |
| 2117 | LMAN2L | Q9H0V9 | 60851.86541 |
| 2118 | EMC8 | O43402 | 60756.487 |
| 2119 | DNAJC19 | Q96DA6 | 60723.89422 |
| 2120 | MRPS25 | P82663 | 60681.19044 |
| 2121 | GCLM | P48507 | 60640.86744 |
| 2122 | GBP1 | P32455 | 60627.34746 |
| 2123 | HNRNPA0 | Q13151 | 60573.1694 |
| 2124 | AAK1 | Q2M2I8 | 60450.86835 |
| 2125 | GOLGA3 | Q08378 | 60431.356 |
| 2126 | SLC1A5 | Q15758 | 60383.55721 |
| 2127 | RANBP3 | Q9H6Z4 | 60343.54991 |
| 2128 | MRPL47 | Q9HD33 | 60340.70813 |
| 2129 | SACM1L | Q9NTJ5 | 60331.51206 |
| 2130 | ATG4B | Q9Y4P1 | 60330.10438 |
| 2131 | ENY2 | Q9NPA8 | 60305.662 |
| 2132 | USP17L10 | C9JJH3 | 60272.896 |
| 2133 | DDX18 | Q9NVP1 | 60265.88212 |
| 2134 | CLIP1 | P30622 | 60201.97681 |
| 2135 | DNAJA2 | O60884 | 60154.93599 |
| 2136 | PLXND1 | Q9Y4D7 | 60139.82898 |
| 2137 | CSTF1 | Q05048 | 60112.66347 |
| 2138 | FNDC3B | Q53EP0 | 60103.40109 |
| 2139 | VAMP3 | Q15836 | 60099.4624 |
| 2140 | SH3PXD2B | A1X283 | 60008.81876 |
| 2141 | NUP88 | Q99567 | 59976.23806 |
| 2142 | RPL37 | P61927 | 59903.13475 |
| 2143 | ARPIN | Q7Z6K5 | 59874.44446 |
| 2144 | MEMO1 | Q9Y316 | 59826.04818 |
| 2145 | RRAGA | Q7L523 | 59801.15243 |
| 2146 | APOO | Q9BUR5 | 59763.364 |
| 2147 | C14orf119 | Q9NWQ9 | 59700.9 |
| 2148 | PPP2R5E | Q16537 | 59654.8377 |
| 2149 | VPS26B | Q4G0F5 | 59631.67977 |
| 2150 | PHLDB1 | Q86UU1 | 59629.37548 |
| 2151 | GINM1 | Q9NU53 | 59618.68264 |
| 2152 | SRSF11 | Q05519 | 59569.49431 |
| 2153 | TM9SF3 | Q9HD45 | 59514.96209 |
| 2154 | DIS3 | Q9Y2L1 | 59497.0642 |
| 2155 | GALE | Q14376 | 59470.76583 |
| 2156 | DCK | P27707 | 59459.77462 |
| 2157 | HM13 | Q8TCT9 | 59446.26387 |
| 2158 | UBE2A | P49459 | 59394.802 |
| 2159 | SLC9A3R2 | Q15599 | 59334.05789 |
| 2160 | IMP3 | Q9NV31 | 59324.0539 |
| 2161 | CCAR1 | Q8IX12 | 59267.77349 |
| 2162 | MXRA7 | P84157 | 59267.69387 |
| 2163 | BAG3 | O95817 | 59240.23076 |
| 2164 | PPP4C | P60510 | 59132.99813 |
| 2165 | RRAS | P10301 | 59117.91713 |
| 2166 | LNPEP | Q9UIQ6 | 59095.58262 |
| 2167 | GYS1 | P13807 | 59060.38718 |
| 2168 | UTP14A | Q9BVJ6 | 59044.30859 |
| 2169 | DPYD | Q12882 | 58978.48902 |
| 2170 | AFAP1 | Q8N556 | 58916.44332 |
| 2171 | SEC62 | Q99442 | 58842.93719 |
| 2172 | NUMA1 | Q14980 | 58773.27315 |
| 2173 | SELENOT | P62341 | 58711.54491 |
| 2174 | SPART | Q8N0X7 | 58638.96342 |
| 2175 | STX4 | Q12846 | 58632.7353 |
| 2176 | NEK9 | Q8TD19 | 58621.08959 |
| 2177 | MRPL58 | Q14197 | 58533.6812 |
| 2178 | UBXN4 | Q92575 | 58377.53469 |
| 2179 | DPYSL4 | O14531 | 58364.81633 |
| 2180 | OSTC | Q9NRP0 | 58353.7772 |
| 2181 | C20orf27 | Q9GZN8 | 58343.26089 |
| 2182 | NDUFB1 | O75438 | 58310.29767 |
| 2183 | GRSF1 | Q12849 | 58244.10204 |
| 2184 | MGAT2 | Q10469 | 58214.49176 |
| 2185 | MFSD10 | Q14728 | 58184.82953 |
| 2186 | TPM2 | P07951 | 58169.27759 |
| 2187 | KRAS | P01116 | 58130.84778 |
| 2188 | GSDMD | P57764 | 58105.31595 |
| 2189 | OPTN | Q96CV9 | 58103.12665 |
| 2190 | PDXK | O00764 | 58051.50121 |
| 2191 | TRMT10C | Q7L0Y3 | 58047.44377 |
| 2192 | PPFIBP1 | Q86W92 | 58011.56511 |
| 2193 | DIAPH2 | O60879 | 58000.59384 |
| 2194 | ZNF74 | Q16587 | 57925.2025 |
| 2195 | FAM162A | Q96A26 | 57891.02133 |
| 2196 | PRPF6 | O94906 | 57783.39501 |
| 2197 | KANK1 | Q14678 | 57782.71723 |
| 2198 | NECAP1 | Q8NC96 | 57738.73133 |
| 2199 | ISOC1 | Q96CN7 | 57707.16754 |
| 2200 | NAT14 | Q8WUY8 | 57687.57488 |
| 2201 | NUMBL | Q9Y6R0 | 57686.48342 |
| 2202 | CTNNBL1 | Q8WYA6 | 57665.49975 |
| 2203 | FTSJ3 | Q8IY81 | 57630.10011 |
| 2204 | GALNT4 | Q8N4A0 | 57627.43103 |
| 2205 | CACNA2D1 | P54289 | 57588.43235 |
| 2206 | LARP7 | Q4G0J3 | 57538.14432 |
| 2207 | PAFAH1B3 | Q15102 | 57500.74113 |
| 2208 | ROCK2 | O75116 | 57450.60806 |
| 2209 | CNOT3 | O75175 | 57437.30415 |
| 2210 | DCAF7 | P61962 | 57416.62506 |
| 2211 | LRRC8A | Q8IWT6 | 57409.80859 |
| 2212 | YTHDF2 | Q9Y5A9 | 57371.94428 |
| 2213 | NSUN2 | Q08J23 | 57334.35365 |
| 2214 | EDC4 | Q6P2E9 | 57332.78116 |
| 2215 | EXOC2 | Q96KP1 | 57321.90053 |
| 2216 | NHLRC2 | Q8NBF2 | 57274.49731 |
| 2217 | NTMT1 | Q9BV86 | 57241.5117 |
| 2218 | MYH14 | Q7Z406 | 57132.0694 |
| 2219 | SAFB | Q15424 | 57130.09358 |
| 2220 | VAMP7 | P51809 | 56797.82857 |
| 2221 | POLDIP3 | Q9BY77 | 56785.60513 |
| 2222 | APOE | P02649 | 56771.69782 |
| 2223 | MRE11 | P49959 | 56761.77566 |
| 2224 | GIMAP8 | Q8ND71 | 56760.22722 |
| 2225 | NUDT3 | O95989 | 56731.95022 |
| 2226 | TSC22D4 | Q9Y3Q8 | 56708.71355 |
| 2227 | NDUFB8 | O95169 | 56684.47975 |
| 2228 | IAH1 | Q2TAA2 | 56667.36573 |
| 2229 | NOSIP | Q9Y314 | 56639.85006 |
| 2230 | RTF1 | Q92541 | 56616.0345 |
| 2231 | AIDA | Q96BJ3 | 56470.62853 |
| 2232 | WBP11 | Q9Y2W2 | 56393.72863 |
| 2233 | COL4A2 | P08572 | 56344.16244 |
| 2234 | SUMF1 | Q8NBK3 | 56310.52662 |
| 2235 | PKN1 | Q16512 | 56243.27695 |
| 2236 | SEC16A | O15027 | 56233.89151 |
| 2237 | APH1A | Q96BI3 | 56232.0966 |
| 2238 | SDF4 | Q9BRK5 | 56188.83035 |
| 2239 | MAP2K2 | P36507 | 56102.032 |
| 2240 | SKIV2L | Q15477 | 56080.8416 |
| 2241 | SERF2 | P84101 | 56003.819 |
| 2242 | LYRM4 | Q9HD34 | 55973.85025 |
| 2243 | ALG5 | Q9Y673 | 55931.13153 |
| 2244 | ZNRD2 | O60232 | 55881.397 |
| 2245 | ACTR1B | P42025 | 55879.93253 |
| 2246 | STRN | O43815 | 55868.49392 |
| 2247 | MCU | Q8NE86 | 55855.38856 |
| 2248 | SETD3 | Q86TU7 | 55794.72051 |
| 2249 | ITGB3 | P05106 | 55761.45074 |
| 2250 | CDK17 | Q00537 | 55672.11118 |
| 2251 | DFFA | O00273 | 55646.12184 |
| 2252 | RAD50 | Q92878 | 55561.05801 |
| 2253 | CDK5 | Q00535 | 55485.48219 |
| 2254 | POLR2B | P30876 | 55372.29967 |
| 2255 | VCAN | P13611 | 55354.48998 |
| 2256 | OCIAD2 | Q56VL3 | 55338.21482 |
| 2257 | MRPS33 | Q9Y291 | 55286.103 |
| 2258 | MFF | Q9GZY8 | 55247.06524 |
| 2259 | BPNT2 | Q9NX62 | 55241.41213 |
| 2260 | TM9SF2 | Q99805 | 55192.2888 |
| 2261 | SSU72 | Q9NP77 | 55190.21245 |
| 2262 | NEDD4L | Q96PU5 | 55155.37581 |
| 2263 | EXOSC6 | Q5RKV6 | 55088.39109 |
| 2264 | SNF8 | Q96H20 | 55063.35278 |
| 2265 | NUDT4 | Q9NZJ9 | 55041.59875 |
| 2266 | POLE3 | Q9NRF9 | 55009.16775 |
| 2267 | EFNB1 | P98172 | 54918.91371 |
| 2268 | GNPDA2 | Q8TDQ7 | 54825.2295 |
| 2269 | ATXN2 | Q99700 | 54759.69021 |
| 2270 | SUCLG1 | P53597 | 54701.73669 |
| 2271 | HGH1 | Q9BTY7 | 54662.21913 |
| 2272 | CUL2 | Q13617 | 54590.6513 |
| 2273 | MPRIP | Q6WCQ1 | 54563.94027 |
| 2274 | TRAPPC4 | Q9Y296 | 54540.72313 |
| 2275 | ACIN1 | Q9UKV3 | 54394.51133 |
| 2276 | TOR4A | Q9NXH8 | 54352.04512 |
| 2277 | PLCG1 | P19174 | 54203.23653 |
| 2278 | MAP7D3 | Q8IWC1 | 54114.40933 |
| 2279 | PON2 | Q15165 | 54104.50219 |
| 2280 | MAP3K3 | Q99759 | 54101.50862 |
| 2281 | NUDT1 | P36639 | 54044.54871 |
| 2282 | TYMS | P04818 | 54029.5562 |
| 2283 | CHERP | Q8IWX8 | 54026.37197 |
| 2284 | APP | P05067 | 54011.75903 |
| 2285 | NFYB | P25208 | 53993.225 |
| 2286 | HSPA13 | P48723 | 53973.23996 |
| 2287 | IFT27 | Q9BW83 | 53913.78408 |
| 2288 | TRIQK | Q629K1 | 53874.70667 |
| 2289 | CDC73 | Q6P1J9 | 53816.85474 |
| 2290 | SYPL1 | Q16563 | 53750.316 |
| 2291 | USP39 | Q53GS9 | 53700.15241 |
| 2292 | PAM16 | Q9Y3D7 | 53664.82543 |
| 2293 | OPA1 | O60313 | 53650.5104 |
| 2294 | RP2 | O75695 | 53634.72619 |
| 2295 | ATAD3A | Q9NVI7 | 53619.17076 |
| 2296 | AFG3L2 | Q9Y4W6 | 53610.43117 |
| 2297 | SH3GLB2 | Q9NR46 | 53571.66922 |
| 2298 | ALAD | P13716 | 53564.15263 |
| 2299 | PRSS23 | O95084 | 53552.66233 |
| 2300 | PPIE | Q9UNP9 | 53495.98311 |
| 2301 | NUDCD3 | Q8IVD9 | 53484.43082 |
| 2302 | RMDN3 | Q96TC7 | 53474.33424 |
| 2303 | MTCH1 | Q9NZJ7 | 53413.38233 |
| 2304 | DPY19L1 | Q2PZI1 | 53389.17537 |
| 2305 | TIMM22 | Q9Y584 | 53329.345 |
| 2306 | DDI2 | Q5TDH0 | 53327.72938 |
| 2307 | PDE12 | Q6L8Q7 | 53327.00108 |
| 2308 | NDUFC2 | O95298 | 53324.98 |
| 2309 | MRPL1 | Q9BYD6 | 53217.81306 |
| 2310 | LPCAT1 | Q8NF37 | 53194.43364 |
| 2311 | CYRIA | Q9H0Q0 | 53194.0955 |
| 2312 | MRRF | Q96E11 | 53181.45639 |
| 2313 | CCDC25 | Q86WR0 | 53158.68407 |
| 2314 | TMEM63B | Q5T3F8 | 53114.33477 |
| 2315 | RAB11FIP5 | Q9BXF6 | 53004.43937 |
| 2316 | SNRNP40 | Q96DI7 | 52986.25461 |
| 2317 | EPB41L2 | O43491 | 52983.52533 |
| 2318 | RAPH1 | Q70E73 | 52969.87497 |
| 2319 | AKR1D1 | P51857 | 52871.0619 |
| 2320 | SMN1 | Q16637 | 52870.79844 |
| 2321 | NEGR1 | Q7Z3B1 | 52802.41543 |
| 2322 | PTPN11 | Q06124 | 52719.69964 |
| 2323 | CBX1 | P83916 | 52689.1664 |
| 2324 | POLDIP2 | Q9Y2S7 | 52677.95363 |
| 2325 | RIPOR1 | Q6ZS17 | 52619.28218 |
| 2326 | AP1S2 | P56377 | 52613.705 |
| 2327 | B2M | P61769 | 52586.75617 |
| 2328 | NUP153 | P49790 | 52522.33747 |
| 2329 | ARRB1 | P49407 | 52497.7287 |
| 2330 | CPNE2 | Q96FN4 | 52440.84284 |
| 2331 | NAGLU | P54802 | 52438.57335 |
| 2332 | DPCD | Q9BVM2 | 52380.99533 |
| 2333 | ZC3H15 | Q8WU90 | 52373.17721 |
| 2334 | C1QTNF3 | Q9BXJ4 | 52241.60688 |
| 2335 | LSG1 | Q9H089 | 52194.94546 |
| 2336 | MRPS34 | P82930 | 52151.96931 |
| 2337 | INPPL1 | O15357 | 52131.37428 |
| 2338 | PTPN12 | Q05209 | 52110.46766 |
| 2339 | USP15 | Q9Y4E8 | 52094.51221 |
| 2340 | GLCE | O94923 | 52059.67268 |
| 2341 | PTK2 | Q05397 | 52058.06988 |
| 2342 | UBE2G1 | P62253 | 52045.44036 |
| 2343 | ARHGAP24 | Q8N264 | 51944.38424 |
| 2344 | STAB1 | Q9NY15 | 51848.73351 |
| 2345 | EXOSC2 | Q13868 | 51796.58357 |
| 2346 | STXBP1 | P61764 | 51779.25839 |
| 2347 | TMEM115 | Q12893 | 51759.68118 |
| 2348 | ZNF598 | Q86UK7 | 51750.40838 |
| 2349 | POP7 | O75817 | 51715.674 |
| 2350 | CCDC93 | Q567U6 | 51611.90162 |
| 2351 | SEC22A | Q96IW7 | 51503.76 |
| 2352 | EML1 | O00423 | 51474.04805 |
| 2353 | ENAH | Q8N8S7 | 51466.49995 |
| 2354 | HINT2 | Q9BX68 | 51379.14255 |
| 2355 | FAM114A2 | Q9NRY5 | 51176.55029 |
| 2356 | SNRNP27 | Q8WVK2 | 51154.26483 |
| 2357 | GRIPAP1 | Q4V328 | 51142.35445 |
| 2358 | CUL1 | Q13616 | 51127.176 |
| 2359 | ENSA | O43768 | 51119.60266 |
| 2360 | TSFM | P43897 | 51117.64061 |
| 2361 | CDC123 | O75794 | 51111.91625 |
| 2362 | SGTB | Q96EQ0 | 51056.02617 |
| 2363 | FNTA | P49354 | 51048.33281 |
| 2364 | NUP50 | Q9UKX7 | 51046.54815 |
| 2365 | BROX | Q5VW32 | 51028.1006 |
| 2366 | RGS10 | O43665 | 51012.06638 |
| 2367 | GALM | Q96C23 | 50960.01 |
| 2368 | ABRAXAS2 | Q15018 | 50946.02152 |
| 2369 | PRKAG1 | P54619 | 50881.522 |
| 2370 | RNF181 | Q9P0P0 | 50861.25444 |
| 2371 | VRK1 | Q99986 | 50830.16005 |
| 2372 | ABI3 | Q9P2A4 | 50781.46748 |
| 2373 | STX8 | Q9UNK0 | 50711.715 |
| 2374 | ANO10 | Q9NW15 | 50698.00477 |
| 2375 | GSPT1 | P15170 | 50506.39196 |
| 2376 | MRPL53 | Q96EL3 | 50491.492 |
| 2377 | MRPS22 | P82650 | 50478.07383 |
| 2378 | SEC24B | O95487 | 50389.51962 |
| 2379 | MCM5 | P33992 | 50352.41963 |
| 2380 | AFP | P02771 | 50332.44209 |
| 2381 | EXOSC8 | Q96B26 | 50319.63008 |
| 2382 | BMX | P51813 | 50317.76328 |
| 2383 | KIFBP | Q96EK5 | 50234.32852 |
| 2384 | DPM3 | Q9P2X0 | 50223.785 |
| 2385 | PPM1A | P35813 | 50136.67084 |
| 2386 | RPAP3 | Q9H6T3 | 50122.73309 |
| 2387 | AKT1 | P31749 | 50122.02541 |
| 2388 | MRPS35 | P82673 | 50114.88273 |
| 2389 | GBF1 | Q92538 | 50111.95194 |
| 2390 | ARHGEF2 | Q92974 | 50108.60422 |
| 2391 | CCNY | Q8ND76 | 50107.102 |
| 2392 | GTF2A1 | P52655 | 50085.768 |
| 2393 | PLSCR4 | Q9NRQ2 | 50084.41527 |
| 2394 | CDC42BPB | Q9Y5S2 | 50059.31846 |
| 2395 | CDH2 | P19022 | 49997.59221 |
| 2396 | MRPL19 | P49406 | 49997.0793 |
| 2397 | TBL2 | Q9Y4P3 | 49971.21489 |
| 2398 | SPECC1L | Q69YQ0 | 49967.10575 |
| 2399 | RAB34 | Q9BZG1 | 49950.28317 |
| 2400 | VAT1L | Q9HCJ6 | 49941.48359 |
| 2401 | MRPL48 | Q96GC5 | 49898.2208 |
| 2402 | KRTCAP2 | Q8N6L1 | 49886.749 |
| 2403 | AACS | Q86V21 | 49796.58645 |
| 2404 | MRPL49 | Q13405 | 49772.43791 |
| 2405 | GNAQ | P50148 | 49691.91795 |
| 2406 | PNPLA6 | Q8IY17 | 49687.52308 |
| 2407 | SAP30BP | Q9UHR5 | 49678.57849 |
| 2408 | GSTZ1 | O43708 | 49659.70789 |
| 2409 | VPS13C | Q709C8 | 49652.54719 |
| 2410 | NAXE | Q8NCW5 | 49572.31181 |
| 2411 | TOR1AIP2 | Q8NFQ8 | 49552.18267 |
| 2412 | MRPL13 | Q9BYD1 | 49513.20408 |
| 2413 | OGT | O15294 | 49426.13388 |
| 2414 | IKBKB | O14920 | 49395.46472 |
| 2415 | TBC1D15 | Q8TC07 | 49353.72257 |
| 2416 | MTREX | P42285 | 49349.21235 |
| 2417 | POGLUT3 | Q7Z4H8 | 49273.9139 |
| 2418 | GLRX | P35754 | 49223.1198 |
| 2419 | USE1 | Q9NZ43 | 49192.10828 |
| 2420 | SLC35A4 | L0R6Q1 | 49183.10683 |
| 2421 | MTMR6 | Q9Y217 | 49029.30183 |
| 2422 | ASNS | P08243 | 48969.68506 |
| 2423 | RRAS2 | P62070 | 48961.26167 |
| 2424 | TAPBP | O15533 | 48945.945 |
| 2425 | PALLD | Q8WX93 | 48944.82856 |
| 2426 | MRPL14 | Q6P1L8 | 48940.6655 |
| 2427 | GLT8D1 | Q68CQ7 | 48909.5237 |
| 2428 | PRKCA | P17252 | 48891.44515 |
| 2429 | LACTB | P83111 | 48856.90032 |
| 2430 | TMBIM1 | Q969X1 | 48853.89571 |
| 2431 | GOLGA2 | Q08379 | 48777.83965 |
| 2432 | ABCF3 | Q9NUQ8 | 48731.46619 |
| 2433 | APPL1 | Q9UKG1 | 48710.16453 |
| 2434 | GGA1 | Q9UJY5 | 48688.75296 |
| 2435 | GSK3B | P49841 | 48654.22571 |
| 2436 | NUDT2 | P50583 | 48628.3758 |
| 2437 | ATP13A1 | Q9HD20 | 48619.6993 |
| 2438 | ZFR | Q96KR1 | 48590.19975 |
| 2439 | BCS1L | Q9Y276 | 48561.99979 |
| 2440 | FAHD1 | Q6P587 | 48525.83317 |
| 2441 | PDCL | Q13371 | 48482.3182 |
| 2442 | HIBCH | Q6NVY1 | 48264.50983 |
| 2443 | KPNA1 | P52294 | 48196.9752 |
| 2444 | TMX4 | Q9H1E5 | 48184.86733 |
| 2445 | GALNT1 | Q10472 | 48116.38641 |
| 2446 | AGTRAP | Q6RW13 | 48101.676 |
| 2447 | MRPL18 | Q9H0U6 | 48076.471 |
| 2448 | PNN | Q9H307 | 48072.58643 |
| 2449 | SPP2 | Q13103 | 47990.438 |
| 2450 | NCS1 | P62166 | 47962.631 |
| 2451 | SLC25A10 | Q9UBX3 | 47922.0148 |
| 2452 | EIF1 | P41567 | 47845.09 |
| 2453 | NELFE | P18615 | 47816.43968 |
| 2454 | MAOB | P27338 | 47802.66667 |
| 2455 | CD93 | Q9NPY3 | 47743.2495 |
| 2456 | LAMA4 | Q16363 | 47691.26092 |
| 2457 | SNX17 | Q15036 | 47618.13217 |
| 2458 | NUP155 | O75694 | 47560.94279 |
| 2459 | FECH | P22830 | 47545.66088 |
| 2460 | NDUFAF4 | Q9P032 | 47545.04707 |
| 2461 | ADIRF | Q15847 | 47506.39908 |
| 2462 | NAGA | P17050 | 47410.47774 |
| 2463 | LRSAM1 | Q6UWE0 | 47346.28173 |
| 2464 | WDR5 | P61964 | 47341.42431 |
| 2465 | MVB12A | Q96EY5 | 47336.62525 |
| 2466 | PTP4A1 | Q93096 | 47322.37 |
| 2467 | ERC1 | Q8IUD2 | 47255.75369 |
| 2468 | EI24 | O14681 | 47251.1085 |
| 2469 | RABEP2 | Q9H5N1 | 47139.72626 |
| 2470 | CARD19 | Q96LW7 | 47090.4428 |
| 2471 | TMEM50A | O95807 | 47039.59671 |
| 2472 | CASP7 | P55210 | 46969.37061 |
| 2473 | POLR2G | P62487 | 46949.51782 |
| 2474 | PIK3C3 | Q8NEB9 | 46921.30857 |
| 2475 | OR5K3 | A6NET4 | 46916.025 |
| 2476 | ZPR1 | O75312 | 46779.84692 |
| 2477 | IKBKG | Q9Y6K9 | 46759.76774 |
| 2478 | RIDA | P52758 | 46759.76243 |
| 2479 | ARSF | P54793 | 46742.21414 |
| 2480 | RRP9 | O43818 | 46711.63568 |
| 2481 | TMTC3 | Q6ZXV5 | 46694.89174 |
| 2482 | CHURC1 | Q8WUH1 | 46689.382 |
| 2483 | MEA1 | Q16626 | 46673.7368 |
| 2484 | FIP1L1 | Q6UN15 | 46632.81768 |
| 2485 | HMGA2 | P52926 | 46616.24534 |
| 2486 | CHMP5 | Q9NZZ3 | 46581.6795 |
| 2487 | UCKL1 | Q9NWZ5 | 46566.50225 |
| 2488 | ACP2 | P11117 | 46502.307 |
| 2489 | ACADS | P16219 | 46432.57548 |
| 2490 | CHMP2B | Q9UQN3 | 46278.063 |
| 2491 | SS18 | Q15532 | 46230.66567 |
| 2492 | RFC5 | P40937 | 46196.91713 |
| 2493 | CDC34 | P49427 | 46187.7387 |
| 2494 | NRM | Q8IXM6 | 46182.851 |
| 2495 | ASDURF | L0R819 | 46168.05317 |
| 2496 | ARMT1 | Q9H993 | 46143.69989 |
| 2497 | DPP9 | Q86TI2 | 46121.62279 |
| 2498 | NUP35 | Q8NFH5 | 46077.02457 |
| 2499 | HSDL2 | Q6YN16 | 46035.50864 |
| 2500 | SRSF4 | Q08170 | 45996.41964 |
| 2501 | RWDD4 | Q6NW29 | 45979.47863 |
| 2502 | EXOC4 | Q96A65 | 45951.72929 |
| 2503 | ATG7 | O95352 | 45922.85442 |
| 2504 | CPSF1 | Q10570 | 45907.94413 |
| 2505 | SLC27A4 | Q6P1M0 | 45877.63149 |
| 2506 | STRN4 | Q9NRL3 | 45850.80646 |
| 2507 | PTCD3 | Q96EY7 | 45722.45221 |
| 2508 | FAM98B | Q52LJ0 | 45677.30291 |
| 2509 | NEMF | O60524 | 45560.99035 |
| 2510 | MSH2 | P43246 | 45528.3522 |
| 2511 | ILVBL | A1L0T0 | 45514.66089 |
| 2512 | GAPVD1 | Q14C86 | 45510.98687 |
| 2513 | U2SURP | O15042 | 45455.84402 |
| 2514 | STXBP2 | Q15833 | 45444.17461 |
| 2515 | RIT1 | Q92963 | 45436.34159 |
| 2516 | SYNJ2BP | P57105 | 45415.1705 |
| 2517 | ERI1 | Q8IV48 | 45402.79423 |
| 2518 | RBM25 | P49756 | 45392.06211 |
| 2519 | HNRNPLL | Q8WVV9 | 45381.28856 |
| 2520 | BYSL | Q13895 | 45338.61158 |
| 2521 | HAT1 | O14929 | 45317.67259 |
| 2522 | BSDC1 | Q9NW68 | 45239.52583 |
| 2523 | SLC12A4 | Q9UP95 | 45237.92773 |
| 2524 | COMMD6 | Q7Z4G1 | 45183.73871 |
| 2525 | LUC7L | Q9NQ29 | 45177.34239 |
| 2526 | SHTN1 | A0MZ66 | 45174.66451 |
| 2527 | MRPL38 | Q96DV4 | 45125.80465 |
| 2528 | PPME1 | Q9Y570 | 45112.91589 |
| 2529 | BLOC1S1 | P78537 | 45096.01163 |
| 2530 | ACOX1 | Q15067 | 45095.42948 |
| 2531 | ELMO2 | Q96JJ3 | 45072.51018 |
| 2532 | RANBP2 | P49792 | 45062.086 |
| 2533 | SMTN | P53814 | 45040.38598 |
| 2534 | UBE2H | P62256 | 45036.64429 |
| 2535 | NUP107 | P57740 | 45012.86323 |
| 2536 | RRM2B | Q7LG56 | 45010.37721 |
| 2537 | CAP2 | P40123 | 44953.64219 |
| 2538 | JAGN1 | Q8N5M9 | 44921.71857 |
| 2539 | RAB21 | Q9UL25 | 44697.90854 |
| 2540 | RRP1B | Q14684 | 44695.51044 |
| 2541 | ATG5 | Q9H1Y0 | 44663.01887 |
| 2542 | TP53I11 | O14683 | 44662.11867 |
| 2543 | ADAM10 | O14672 | 44655.37303 |
| 2544 | HOMER3 | Q9NSC5 | 44619.15481 |
| 2545 | TIMM8A | O60220 | 44602.39767 |
| 2546 | BICD2 | Q8TD16 | 44435.02646 |
| 2547 | AXL | P30530 | 44402.34063 |
| 2548 | MRPL54 | Q6P161 | 44349.9024 |
| 2549 | COASY | Q13057 | 44313.92595 |
| 2550 | AK6 | Q9Y3D8 | 44292.479 |
| 2551 | RCL1 | Q9Y2P8 | 44207.30375 |
| 2552 | TRIP6 | Q15654 | 44206.78271 |
| 2553 | NUCKS1 | Q9H1E3 | 44185.92244 |
| 2554 | COMMD2 | Q86X83 | 44147.071 |
| 2555 | TRIM21 | P19474 | 44142.56227 |
| 2556 | NFKB2 | Q00653 | 44064.43544 |
| 2557 | CPT2 | P23786 | 44039.06823 |
| 2558 | OGA | O60502 | 44038.61541 |
| 2559 | DDA1 | Q9BW61 | 43962.13014 |
| 2560 | YME1L1 | Q96TA2 | 43960.5628 |
| 2561 | MVD | P53602 | 43952.07415 |
| 2562 | NQO2 | P16083 | 43940.46764 |
| 2563 | RRM2 | P31350 | 43873.41892 |
| 2564 | RAB3D | O95716 | 43783.7685 |
| 2565 | MYD88 | Q99836 | 43756.24818 |
| 2566 | SMARCB1 | Q12824 | 43732.8325 |
| 2567 | GLUD1 | P00367 | 43665.639 |
| 2568 | DYNLL1 | P63167 | 43631.401 |
| 2569 | ASCC3 | Q8N3C0 | 43624.85717 |
| 2570 | MRPL4 | Q9BYD3 | 43598.56993 |
| 2571 | PWP2 | Q15269 | 43593.34045 |
| 2572 | IMP4 | Q96G21 | 43560.66286 |
| 2573 | HCFC1 | P51610 | 43557.45476 |
| 2574 | GSK3A | P49840 | 43540.9215 |
| 2575 | COMMD1 | Q8N668 | 43536.15864 |
| 2576 | GATD1 | Q8NB37 | 43536.00222 |
| 2577 | EIPR1 | Q53HC9 | 43508.3325 |
| 2578 | RTL8C | A6ZKI3 | 43488.88738 |
| 2579 | CAB39 | Q9Y376 | 43467.02692 |
| 2580 | NXN | Q6DKJ4 | 43452.9941 |
| 2581 | SMC4 | Q9NTJ3 | 43419.33303 |
| 2582 | SLC27A3 | Q5K4L6 | 43414.7685 |
| 2583 | SLC16A1 | P53985 | 43359.3428 |
| 2584 | H6PD | O95479 | 43347.20627 |
| 2585 | EIF2B4 | Q9UI10 | 43318.21348 |
| 2586 | IFIT3 | O14879 | 43219.1636 |
| 2587 | VPS33B | Q9H267 | 43149.38683 |
| 2588 | RFTN1 | Q14699 | 43112.78714 |
| 2589 | ENDOG | Q14249 | 43061.23889 |
| 2590 | NOL4L | Q96MY1 | 42930.99754 |
| 2591 | ARAP3 | Q8WWN8 | 42927.61201 |
| 2592 | CPSF3 | Q9UKF6 | 42926.28549 |
| 2593 | SCYL1 | Q96KG9 | 42878.56649 |
| 2594 | BCCIP | Q9P287 | 42872.68199 |
| 2595 | PARVB | Q9HBI1 | 42834.22716 |
| 2596 | WASH6P | Q9NQA3 | 42802.27627 |
| 2597 | APOC2 | P02655 | 42773.3075 |
| 2598 | PES1 | O00541 | 42762.29023 |
| 2599 | RNF40 | O75150 | 42725.77486 |
| 2600 | YAP1 | P46937 | 42698.54048 |
| 2601 | CSTF3 | Q12996 | 42690.33337 |
| 2602 | IDI1 | Q13907 | 42669.78408 |
| 2603 | PRPF3 | O43395 | 42632.3643 |
| 2604 | DOHH | Q9BU89 | 42615.26907 |
| 2605 | NBAS | A2RRP1 | 42612.48692 |
| 2606 | WRNIP1 | Q96S55 | 42567.82156 |
| 2607 | SULT1E1 | P49888 | 42479.40326 |
| 2608 | PALS2 | Q9NZW5 | 42447.91609 |
| 2609 | TCOF1 | Q13428 | 42412.25589 |
| 2610 | AAAS | Q9NRG9 | 42368.12918 |
| 2611 | RNASEH2C | Q8TDP1 | 42357.7091 |
| 2612 | ARHGAP29 | Q52LW3 | 42316.00896 |
| 2613 | THOC6 | Q86W42 | 42313.39332 |
| 2614 | HDAC2 | Q92769 | 42248.69565 |
| 2615 | STX11 | O75558 | 42214.71765 |
| 2616 | CTBP1 | Q13363 | 42206.72548 |
| 2617 | TXNL4A | P83876 | 42191.698 |
| 2618 | MINPP1 | Q9UNW1 | 42171.2735 |
| 2619 | ZNF622 | Q969S3 | 42125.47912 |
| 2620 | CSNK2A2 | P19784 | 42113.92853 |
| 2621 | NGDN | Q8NEJ9 | 42091.30406 |
| 2622 | JUN | P05412 | 42067.3322 |
| 2623 | ADD3 | Q9UEY8 | 42056.03476 |
| 2624 | TACC1 | O75410 | 42054.5212 |
| 2625 | BIN3 | Q9NQY0 | 42040.00273 |
| 2626 | BANF1 | O75531 | 41971.8996 |
| 2627 | SLC9A3R1 | O14745 | 41920.50636 |
| 2628 | SELENON | Q9NZV5 | 41893.76891 |
| 2629 | MRI1 | Q9BV20 | 41888.70948 |
| 2630 | RETSAT | Q6NUM9 | 41824.44466 |
| 2631 | MRPL9 | Q9BYD2 | 41819.24025 |
| 2632 | MECP2 | P51608 | 41806.59998 |
| 2633 | ABCD3 | P28288 | 41726.30711 |
| 2634 | GSTM1 | P09488 | 41706.07163 |
| 2635 | COPG2 | Q9UBF2 | 41668.75689 |
| 2636 | ZC3H14 | Q6PJT7 | 41666.29437 |
| 2637 | RFC4 | P35249 | 41653.59406 |
| 2638 | TOP2B | Q02880 | 41599.12143 |
| 2639 | UBE2V2 | Q15819 | 41581.087 |
| 2640 | RDH14 | Q9HBH5 | 41546.96032 |
| 2641 | DIMT1 | Q9UNQ2 | 41489.3351 |
| 2642 | NUP205 | Q92621 | 41477.68484 |
| 2643 | RPP30 | P78346 | 41477.05613 |
| 2644 | RSL24D1 | Q9UHA3 | 41459.9325 |
| 2645 | KIF13B | Q9NQT8 | 41458.44206 |
| 2646 | USP9X | Q93008 | 41438.86307 |
| 2647 | CAMK2D | Q13557 | 41425.65788 |
| 2648 | SMARCA5 | O60264 | 41423.81879 |
| 2649 | SRPK1 | Q96SB4 | 41346.92929 |
| 2650 | GAK | O14976 | 41286.05863 |
| 2651 | SAMHD1 | Q9Y3Z3 | 41273.05063 |
| 2652 | BIN1 | O00499 | 41219.93064 |
| 2653 | TBC1D9B | Q66K14 | 41196.53548 |
| 2654 | SPCS1 | Q9Y6A9 | 41191.835 |
| 2655 | YARS2 | Q9Y2Z4 | 41180.69819 |
| 2656 | MRPS2 | Q9Y399 | 41167.56795 |
| 2657 | HK2 | P52789 | 41129.88284 |
| 2658 | SPATS2 | Q86XZ4 | 41121.18856 |
| 2659 | PEX14 | O75381 | 41047.12256 |
| 2660 | SMAD3 | P84022 | 41008.15395 |
| 2661 | RPIA | P49247 | 40923.90211 |
| 2662 | PTGES2 | Q9H7Z7 | 40906.04938 |
| 2663 | TIMM8B | Q9Y5J9 | 40875.41929 |
| 2664 | IDH3B | O43837 | 40858.74786 |
| 2665 | KYAT3 | Q6YP21 | 40826.10909 |
| 2666 | DLG1 | Q12959 | 40773.57361 |
| 2667 | EML2 | O95834 | 40762.68006 |
| 2668 | XPO7 | Q9UIA9 | 40757.62839 |
| 2669 | BOP1 | Q14137 | 40734.53697 |
| 2670 | PPAT | Q06203 | 40725.23278 |
| 2671 | SHFL | Q9NUL5 | 40718.59308 |
| 2672 | TRPV2 | Q9Y5S1 | 40666.78887 |
| 2673 | UBR4 | Q5T4S7 | 40592.66929 |
| 2674 | EML3 | Q32P44 | 40587.48868 |
| 2675 | SCO2 | O43819 | 40565.71375 |
| 2676 | PUM3 | Q15397 | 40544.93515 |
| 2677 | EMC4 | Q5J8M3 | 40533.93267 |
| 2678 | FNTB | P49356 | 40504.30246 |
| 2679 | TRA2A | Q13595 | 40473.01167 |
| 2680 | PRPF4 | O43172 | 40415.63945 |
| 2681 | GPAA1 | O43292 | 40413.6459 |
| 2682 | RAB3GAP1 | Q15042 | 40392.46244 |
| 2683 | EPN2 | O95208 | 40293.74726 |
| 2684 | TOM1L2 | Q6ZVM7 | 40291.32805 |
| 2685 | TUBGCP2 | Q9BSJ2 | 40281.88786 |
| 2686 | BABAM2 | Q9NXR7 | 40264.54983 |
| 2687 | SF3B4 | Q15427 | 40261.57675 |
| 2688 | SPRYD7 | Q5W111 | 40215.8245 |
| 2689 | DOCK6 | Q96HP0 | 40122.10276 |
| 2690 | SPRYD4 | Q8WW59 | 40092.03413 |
| 2691 | PAXX | Q9BUH6 | 40084.49854 |
| 2692 | ARMC1 | Q9NVT9 | 40073.82825 |
| 2693 | INPP1 | P49441 | 40064.2684 |
| 2694 | RRP15 | Q9Y3B9 | 40050.73631 |
| 2695 | VPS11 | Q9H270 | 40047.06991 |
| 2696 | HDHD5 | Q9BXW7 | 40045.42545 |
| 2697 | NCBP3 | Q53F19 | 40029.868 |
| 2698 | MSRB3 | Q8IXL7 | 40017.19088 |
| 2699 | PDCD1LG2 | Q9BQ51 | 39997.61546 |
| 2700 | LOX | P28300 | 39933.90017 |
| 2701 | AARSD1 | Q9BTE6 | 39918.26705 |
| 2702 | TPRKB | Q9Y3C4 | 39893.403 |
| 2703 | ALDH4A1 | P30038 | 39765.98717 |
| 2704 | VPS36 | Q86VN1 | 39753.38421 |
| 2705 | GTF2F1 | P35269 | 39749.71395 |
| 2706 | NMRAL1 | Q9HBL8 | 39712.52159 |
| 2707 | GIT1 | Q9Y2X7 | 39613.29695 |
| 2708 | SELENOF | O60613 | 39574.05444 |
| 2709 | PCYT1A | P49585 | 39480.97348 |
| 2710 | PCCA | P05165 | 39402.19967 |
| 2711 | MRPS6 | P82932 | 39386.765 |
| 2712 | SRC | P12931 | 39375.21223 |
| 2713 | RBMXL2 | O75526 | 39367.78476 |
| 2714 | HBS1L | Q9Y450 | 39351.51048 |
| 2715 | C15orf40 | Q8WUR7 | 39247.96 |
| 2716 | SAFB2 | Q14151 | 39170.26052 |
| 2717 | WWTR1 | Q9GZV5 | 39163.78848 |
| 2718 | GBP2 | P32456 | 39159.97221 |
| 2719 | LRRC57 | Q8N9N7 | 39155.18367 |
| 2720 | STX6 | O43752 | 39117.61854 |
| 2721 | TMEM126A | Q9H061 | 39092.27964 |
| 2722 | OSBPL3 | Q9H4L5 | 39086.90425 |
| 2723 | PDGFB | P01127 | 39070.06685 |
| 2724 | FRMD8 | Q9BZ67 | 39060.18278 |
| 2725 | PIGS | Q96S52 | 39023.09165 |
| 2726 | DNAJC1 | Q96KC8 | 38993.84856 |
| 2727 | PIGU | Q9H490 | 38978.56455 |
| 2728 | UBAC2 | Q8NBM4 | 38969.90767 |
| 2729 | DDRGK1 | Q96HY6 | 38952.95015 |
| 2730 | WASHC5 | Q12768 | 38943.37908 |
| 2731 | MRPS36 | P82909 | 38926.3495 |
| 2732 | KRT19 | P08727 | 38924.16674 |
| 2733 | MAGOH | P61326 | 38869.4211 |
| 2734 | NADSYN1 | Q6IA69 | 38860.54143 |
| 2735 | FMNL3 | Q8IVF7 | 38819.54253 |
| 2736 | WDFY1 | Q8IWB7 | 38792.08132 |
| 2737 | MRPL28 | Q13084 | 38736.06367 |
| 2738 | BLOC1S2 | Q6QNY1 | 38732.09871 |
| 2739 | AUP1 | Q9Y679 | 38694.25984 |
| 2740 | TBL1XR1 | Q9BZK7 | 38656.07645 |
| 2741 | GIGYF2 | Q6Y7W6 | 38618.03388 |
| 2742 | CDKN2AIPNL | Q96HQ2 | 38614.21633 |
| 2743 | RBM42 | Q9BTD8 | 38610.77321 |
| 2744 | NPEPL1 | Q8NDH3 | 38577.03071 |
| 2745 | TBL3 | Q12788 | 38568.24964 |
| 2746 | ARMC10 | Q8N2F6 | 38562.9102 |
| 2747 | DPF2 | Q92785 | 38516.55238 |
| 2748 | MAP4K4 | O95819 | 38512.37541 |
| 2749 | CFDP1 | Q9UEE9 | 38499.78407 |
| 2750 | SUPT4H1 | P63272 | 38490.82 |
| 2751 | PRPSAP1 | Q14558 | 38459.17671 |
| 2752 | PEX19 | P40855 | 38452.52078 |
| 2753 | A2M | P01023 | 38375.85274 |
| 2754 | PDHX | O00330 | 38368.83436 |
| 2755 | ACACA | Q13085 | 38365.7886 |
| 2756 | CDK7 | P50613 | 38341.59763 |
| 2757 | MMGT1 | Q8N4V1 | 38333.07333 |
| 2758 | RBBP9 | O75884 | 38312.503 |
| 2759 | LRRFIP2 | Q9Y608 | 38270.45544 |
| 2760 | CUL3 | Q13618 | 38258.54968 |
| 2761 | ATXN3 | P54252 | 38246.60664 |
| 2762 | KLHL4 | Q9C0H6 | 38228.80356 |
| 2763 | CDC42EP1 | Q00587 | 38215.44689 |
| 2764 | CHMP3 | Q9Y3E7 | 38207.53091 |
| 2765 | TGFB1 | P01137 | 38170.01576 |
| 2766 | TTC37 | Q6PGP7 | 38141.16443 |
| 2767 | PSIP1 | O75475 | 38137.09345 |
| 2768 | COPS7B | Q9H9Q2 | 38077.35064 |
| 2769 | CHD4 | Q14839 | 38043.26575 |
| 2770 | CC2D1A | Q6P1N0 | 38034.15067 |
| 2771 | LCMT1 | Q9UIC8 | 38023.51689 |
| 2772 | MRPS27 | Q92552 | 37998.12858 |
| 2773 | RANBP9 | Q96S59 | 37973.256 |
| 2774 | SUN1 | O94901 | 37954.63855 |
| 2775 | CHAMP1 | Q96JM3 | 37940.75038 |
| 2776 | RNMT | O43148 | 37915.4556 |
| 2777 | SART3 | Q15020 | 37914.6222 |
| 2778 | HTATIP2 | Q9BUP3 | 37894.30413 |
| 2779 | CD276 | Q5ZPR3 | 37864.18121 |
| 2780 | GOLGA5 | Q8TBA6 | 37807.2916 |
| 2781 | TGFBR2 | P37173 | 37739.06147 |
| 2782 | TMEM70 | Q9BUB7 | 37735.49267 |
| 2783 | NOC2L | Q9Y3T9 | 37666.63107 |
| 2784 | GOLGB1 | Q14789 | 37664.4478 |
| 2785 | UBTF | P17480 | 37661.27665 |
| 2786 | YRDC | Q86U90 | 37651.85192 |
| 2787 | POGLUT2 | Q6UW63 | 37582.66309 |
| 2788 | GPALPP1 | Q8IXQ4 | 37542.56181 |
| 2789 | ELP1 | O95163 | 37397.21506 |
| 2790 | RBM10 | P98175 | 37396.81371 |
| 2791 | ERLEC1 | Q96DZ1 | 37353.4048 |
| 2792 | NCBP1 | Q09161 | 37347.81488 |
| 2793 | DNAJA3 | Q96EY1 | 37272.46961 |
| 2794 | RHOB | P62745 | 37260.56956 |
| 2795 | AKT1S1 | Q96B36 | 37232.70689 |
| 2796 | UAP1L1 | Q3KQV9 | 37229.27624 |
| 2797 | PRRC2C | Q9Y520 | 37178.4154 |
| 2798 | PPP6R3 | Q5H9R7 | 37111.22991 |
| 2799 | MRPS16 | Q9Y3D3 | 37093.1304 |
| 2800 | DDX3Y | O15523 | 37078.23705 |
| 2801 | SUDS3 | Q9H7L9 | 37060.57369 |
| 2802 | SYNJ2 | O15056 | 37034.26712 |
| 2803 | PUS7 | Q96PZ0 | 37013.46671 |
| 2804 | FAM207A | Q9NSI2 | 36993.0645 |
| 2805 | CYSTM1 | Q9H1C7 | 36964.785 |
| 2806 | EDIL3 | O43854 | 36955.89792 |
| 2807 | PPAN | Q9NQ55 | 36948.85548 |
| 2808 | LARP4 | Q71RC2 | 36945.45932 |
| 2809 | OSGEP | Q9NPF4 | 36938.86283 |
| 2810 | OSBP | P22059 | 36843.02719 |
| 2811 | ATP5MJ | P56378 | 36805.375 |
| 2812 | PDCD11 | Q14690 | 36752.75012 |
| 2813 | NUBP2 | Q9Y5Y2 | 36746.03708 |
| 2814 | MRPS11 | P82912 | 36726.54433 |
| 2815 | MTG1 | Q9BT17 | 36676.0128 |
| 2816 | MMTAG2 | Q9BU76 | 36664.48133 |
| 2817 | AHSG | P02765 | 36602.48793 |
| 2818 | NFYC | Q13952 | 36602.12771 |
| 2819 | PUM1 | Q14671 | 36578.06603 |
| 2820 | FSTL1 | Q12841 | 36410.15105 |
| 2821 | PIGT | Q969N2 | 36370.49262 |
| 2822 | CNOT1 | A5YKK6 | 36366.73791 |
| 2823 | MRPL20 | Q9BYC9 | 36327.1425 |
| 2824 | UCK2 | Q9BZX2 | 36311.03277 |
| 2825 | SOS1 | Q07889 | 36278.32317 |
| 2826 | ALDH1B1 | P30837 | 36258.26148 |
| 2827 | CLPB | Q9H078 | 36233.33728 |
| 2828 | ITPRID2 | P28290 | 36225.88422 |
| 2829 | PSMB8 | P28062 | 36181.64594 |
| 2830 | NAA30 | Q147X3 | 36127.81833 |
| 2831 | RHOT2 | Q8IXI1 | 36113.39722 |
| 2832 | WASF1 | Q92558 | 36108.46811 |
| 2833 | SPTLC1 | O15269 | 36076.24809 |
| 2834 | TRMT44 | Q8IYL2 | 36060.03951 |
| 2835 | MGST3 | O14880 | 36032.84557 |
| 2836 | SUPT5H | O00267 | 36013.73065 |
| 2837 | SVIL | O95425 | 35993.58856 |
| 2838 | CRAT | P43155 | 35939.244 |
| 2839 | FNBP1 | Q96RU3 | 35927.56451 |
| 2840 | THOC7 | Q6I9Y2 | 35923.91008 |
| 2841 | CTPS2 | Q9NRF8 | 35870.92832 |
| 2842 | SNRK | Q9NRH2 | 35868.63156 |
| 2843 | ATP6V1H | Q9UI12 | 35827.97454 |
| 2844 | YIPF3 | Q9GZM5 | 35819.72667 |
| 2845 | ARFIP2 | P53365 | 35808.09075 |
| 2846 | VPS45 | Q9NRW7 | 35773.91688 |
| 2847 | KDSR | Q06136 | 35771.31662 |
| 2848 | CIAO2B | Q9Y3D0 | 35717.109 |
| 2849 | ARIH1 | Q9Y4X5 | 35690.16927 |
| 2850 | MRPL16 | Q9NX20 | 35679.1235 |
| 2851 | RAC3 | P60763 | 35624 |
| 2852 | CHMP4A | Q9BY43 | 35594.6495 |
| 2853 | TBC1D13 | Q9NVG8 | 35553.65292 |
| 2854 | PAK4 | O96013 | 35502.7088 |
| 2855 | SCAMP1 | O15126 | 35463.15169 |
| 2856 | SLC25A4 | P12235 | 35422.1535 |
| 2857 | IL33 | O95760 | 35344.17434 |
| 2858 | CDC27 | P30260 | 35311.32468 |
| 2859 | SLC12A2 | P55011 | 35304.03294 |
| 2860 | OCRL | Q01968 | 35264.02048 |
| 2861 | SNX4 | O95219 | 35224.53626 |
| 2862 | MTHFD2 | P13995 | 35224.46575 |
| 2863 | CLPTM1L | Q96KA5 | 35196.21729 |
| 2864 | TP53BP1 | Q12888 | 35170.33151 |
| 2865 | AMFR | Q9UKV5 | 35134.52446 |
| 2866 | DHX30 | Q7L2E3 | 35134.4179 |
| 2867 | GTF2B | Q00403 | 35100.39228 |
| 2868 | YIF1A | O95070 | 35084.00289 |
| 2869 | SNX18 | Q96RF0 | 35062.84259 |
| 2870 | ACSL1 | P33121 | 35030.99439 |
| 2871 | SPATS2L | Q9NUQ6 | 34954.70836 |
| 2872 | PPP1R2 | P41236 | 34949.11913 |
| 2873 | ADGRL4 | Q9HBW9 | 34948.7339 |
| 2874 | RBM34 | P42696 | 34939.23431 |
| 2875 | CTU2 | Q2VPK5 | 34917.92408 |
| 2876 | KIF2A | O00139 | 34897.7096 |
| 2877 | ARHGEF10 | O15013 | 34872.3288 |
| 2878 | YLPM1 | P49750 | 34856.09585 |
| 2879 | ACTR10 | Q9NZ32 | 34854.31733 |
| 2880 | CDC42BPA | Q5VT25 | 34852.46847 |
| 2881 | GMDS | O60547 | 34846.90373 |
| 2882 | TSR1 | Q2NL82 | 34804.232 |
| 2883 | ALG2 | Q9H553 | 34792.19797 |
| 2884 | SAP18 | O00422 | 34781.209 |
| 2885 | DUSP12 | Q9UNI6 | 34776.5414 |
| 2886 | FHIP2A | Q5W0V3 | 34774.41744 |
| 2887 | MRPS15 | P82914 | 34742.95407 |
| 2888 | ANKRD50 | Q9ULJ7 | 34742.80656 |
| 2889 | RETREG3 | Q86VR2 | 34739.98639 |
| 2890 | DENR | O43583 | 34694.50653 |
| 2891 | WDR82 | Q6UXN9 | 34689.59271 |
| 2892 | UBE2Z | Q9H832 | 34614.17033 |
| 2893 | DHFR | P00374 | 34593.60823 |
| 2894 | SNRPC | P09234 | 34562.536 |
| 2895 | PLEKHJ1 | Q9NW61 | 34504.71778 |
| 2896 | HSPBP1 | Q9NZL4 | 34480.15615 |
| 2897 | MOB3A | Q96BX8 | 34469.68158 |
| 2898 | CMBL | Q96DG6 | 34449.03713 |
| 2899 | CSTF2 | P33240 | 34444.75091 |
| 2900 | FTO | Q9C0B1 | 34435.71103 |
| 2901 | BAG5 | Q9UL15 | 34409.49179 |
| 2902 | TMPO | P42166 | 34390.89126 |
| 2903 | HSDL1 | Q3SXM5 | 34375.00658 |
| 2904 | UBXN6 | Q9BZV1 | 34319.91668 |
| 2905 | CCDC86 | Q9H6F5 | 34313.67155 |
| 2906 | TRIOBP | Q9H2D6 | 34265.9474 |
| 2907 | FAM136A | Q96C01 | 34264.99445 |
| 2908 | ELMOD2 | Q8IZ81 | 34254.43513 |
| 2909 | IDUA | P35475 | 34250.56881 |
| 2910 | HCCS | P53701 | 34199.21306 |
| 2911 | WASHC4 | Q2M389 | 34188.00529 |
| 2912 | PPP3R1 | P63098 | 34177.67989 |
| 2913 | SAMD1 | Q6SPF0 | 34169.42615 |
| 2914 | ORMDL2 | Q53FV1 | 34141.49017 |
| 2915 | THUMPD1 | Q9NXG2 | 34076.31005 |
| 2916 | NUP160 | Q12769 | 34013.59108 |
| 2917 | NBN | O60934 | 34012.77069 |
| 2918 | ABCC1 | P33527 | 33976.98201 |
| 2919 | CNOT6 | Q9ULM6 | 33921.645 |
| 2920 | ANKFY1 | Q9P2R3 | 33914.00995 |
| 2921 | ANGPTL4 | Q9BY76 | 33912.52865 |
| 2922 | RAP2B | P61225 | 33904.76273 |
| 2923 | PSME3IP1 | Q9GZU8 | 33904.59314 |
| 2924 | XPO5 | Q9HAV4 | 33880.47075 |
| 2925 | TM9SF1 | O15321 | 33798.19417 |
| 2926 | IWS1 | Q96ST2 | 33764.57834 |
| 2927 | DDB2 | Q92466 | 33755.54557 |
| 2928 | ATP6V1F | Q16864 | 33744.29857 |
| 2929 | FLYWCH2 | Q96CP2 | 33710.35338 |
| 2930 | RNF214 | Q8ND24 | 33707.00835 |
| 2931 | RPS6KA3 | P51812 | 33693.72184 |
| 2932 | ARMC8 | Q8IUR7 | 33669.73137 |
| 2933 | USP47 | Q96K76 | 33668.27773 |
| 2934 | POLR3K | Q9Y2Y1 | 33626.51167 |
| 2935 | DDX24 | Q9GZR7 | 33603.30998 |
| 2936 | RPS6KA4 | O75676 | 33589.63258 |
| 2937 | ILKAP | Q9H0C8 | 33587.64057 |
| 2938 | THOC1 | Q96FV9 | 33568.23111 |
| 2939 | ARGLU1 | Q9NWB6 | 33537.08818 |
| 2940 | NDUFA8 | P51970 | 33531.12975 |
| 2941 | MAD1L1 | Q9Y6D9 | 33496.19944 |
| 2942 | ACTN2 | P35609 | 33454.82535 |
| 2943 | EPS8 | Q12929 | 33400.07235 |
| 2944 | CDKAL1 | Q5VV42 | 33383.9921 |
| 2945 | HHIP | Q96QV1 | 33310.90395 |
| 2946 | VPS37A | Q8NEZ2 | 33290.19757 |
| 2947 | BCAM | P50895 | 33288.55213 |
| 2948 | ASCC2 | Q9H1I8 | 33257.17842 |
| 2949 | VPS52 | Q8N1B4 | 33221.41926 |
| 2950 | CETN2 | P41208 | 33202.26333 |
| 2951 | MAP2K3 | P46734 | 33183.83717 |
| 2952 | KRT5 | P13647 | 33175.31619 |
| 2953 | PGGT1B | P53609 | 33159.23748 |
| 2954 | CCDC43 | Q96MW1 | 33126.90727 |
| 2955 | METAP1 | P53582 | 33061.14335 |
| 2956 | WDR36 | Q8NI36 | 33034.85018 |
| 2957 | TAB1 | Q15750 | 33004.69776 |
| 2958 | RAB4B | P61018 | 32982.27393 |
| 2959 | MT-ND1 | P03886 | 32931.283 |
| 2960 | GATAD2A | Q86YP4 | 32919.35803 |
| 2961 | CYP51A1 | Q16850 | 32872.045 |
| 2962 | PPP1R12C | Q9BZL4 | 32842.23382 |
| 2963 | CLCC1 | Q96S66 | 32842.16923 |
| 2964 | VPS16 | Q9H269 | 32836.8012 |
| 2965 | AFDN | P55196 | 32817.79949 |
| 2966 | ZNF346 | Q9UL40 | 32770.47067 |
| 2967 | NAP1L5 | Q96NT1 | 32714.96 |
| 2968 | FKBP15 | Q5T1M5 | 32703.43835 |
| 2969 | NAA25 | Q14CX7 | 32686.60944 |
| 2970 | DNAAF5 | Q86Y56 | 32675.01804 |
| 2971 | DENND10 | Q8TCE6 | 32666.22379 |
| 2972 | MAPK9 | P45984 | 32661.22681 |
| 2973 | MRPL39 | Q9NYK5 | 32660.99152 |
| 2974 | CCDC12 | Q8WUD4 | 32658.41189 |
| 2975 | POFUT2 | Q9Y2G5 | 32644.06596 |
| 2976 | POLR2C | P19387 | 32611.99647 |
| 2977 | PLRG1 | O43660 | 32594.50707 |
| 2978 | BST1 | Q10588 | 32572.88105 |
| 2979 | TIA1 | P31483 | 32558.83181 |
| 2980 | TMEM179B | Q7Z7N9 | 32540.23286 |
| 2981 | TBC1D24 | Q9ULP9 | 32518.17183 |
| 2982 | UQCC2 | Q9BRT2 | 32510.92071 |
| 2983 | RAB29 | O14966 | 32460.51775 |
| 2984 | USP10 | Q14694 | 32444.63414 |
| 2985 | DDX56 | Q9NY93 | 32419.95455 |
| 2986 | TRNT1 | Q96Q11 | 32418.16015 |
| 2987 | NOP53 | Q9NZM5 | 32415.01158 |
| 2988 | GNL1 | P36915 | 32346.50361 |
| 2989 | COX17 | Q14061 | 32339.16275 |
| 2990 | GOSR1 | O95249 | 32328.34944 |
| 2991 | MRPL46 | Q9H2W6 | 32316.34156 |
| 2992 | ASMTL | O95671 | 32279.64103 |
| 2993 | PLD1 | Q13393 | 32252.28472 |
| 2994 | VPS37B | Q9H9H4 | 32210.095 |
| 2995 | POLR1G | O15446 | 32201.33672 |
| 2996 | SCRIB | Q14160 | 32186.49426 |
| 2997 | LIMCH1 | Q9UPQ0 | 32179.19517 |
| 2998 | AARS2 | Q5JTZ9 | 32178.61898 |
| 2999 | TFB1M | Q8WVM0 | 32168.80452 |
| 3000 | PAAF1 | Q9BRP4 | 32159.72 |
| 3001 | SP100 | P23497 | 32132.95807 |
| 3002 | ERI3 | O43414 | 32118.615 |
| 3003 | ARHGEF7 | Q14155 | 32101.49135 |
| 3004 | TMEM167A | Q8TBQ9 | 32043.385 |
| 3005 | COMMD4 | Q9H0A8 | 32009.44458 |
| 3006 | NUP85 | Q9BW27 | 31979.89245 |
| 3007 | COMMD7 | Q86VX2 | 31959.6805 |
| 3008 | WLS | Q5T9L3 | 31959.3508 |
| 3009 | NAV1 | Q8NEY1 | 31937.19086 |
| 3010 | SPAG9 | O60271 | 31928.22986 |
| 3011 | TERF2IP | Q9NYB0 | 31925.53184 |
| 3012 | NUDT11 | Q96G61 | 31909.6437 |
| 3013 | UBASH3B | Q8TF42 | 31882.29256 |
| 3014 | SRP19 | P09132 | 31864.038 |
| 3015 | BABAM1 | Q9NWV8 | 31849.40761 |
| 3016 | TMED8 | Q6PL24 | 31843.00317 |
| 3017 | ELP3 | Q9H9T3 | 31815.91387 |
| 3018 | CLPTM1 | O96005 | 31805.58907 |
| 3019 | STAU2 | Q9NUL3 | 31804.23315 |
| 3020 | MRPL33 | O75394 | 31746.78733 |
| 3021 | TEK | Q02763 | 31732.09633 |
| 3022 | UBE3A | Q05086 | 31712.72242 |
| 3023 | DNPH1 | O43598 | 31701.9119 |
| 3024 | ERGIC3 | Q9Y282 | 31678.96308 |
| 3025 | POGLUT1 | Q8NBL1 | 31671.21624 |
| 3026 | APOOL | Q6UXV4 | 31648.95353 |
| 3027 | SARS2 | Q9NP81 | 31646.59441 |
| 3028 | LRPAP1 | P30533 | 31641.84152 |
| 3029 | MRPL3 | P09001 | 31637.13012 |
| 3030 | RAB24 | Q969Q5 | 31636.48591 |
| 3031 | MAK16 | Q9BXY0 | 31622.7265 |
| 3032 | RBM26 | Q5T8P6 | 31601.41563 |
| 3033 | PLSCR3 | Q9NRY6 | 31598.0027 |
| 3034 | OXNAD1 | Q96HP4 | 31578.16764 |
| 3035 | FKBP1B | P68106 | 31538.14475 |
| 3036 | GOPC | Q9HD26 | 31529.08352 |
| 3037 | RSRC2 | Q7L4I2 | 31518.73157 |
| 3038 | ACOX3 | O15254 | 31514.71164 |
| 3039 | RABEP1 | Q15276 | 31432.2945 |
| 3040 | EXOSC7 | Q15024 | 31410.74772 |
| 3041 | UTP18 | Q9Y5J1 | 31405.68496 |
| 3042 | ABR | Q12979 | 31390.63486 |
| 3043 | TKFC | Q3LXA3 | 31375.48967 |
| 3044 | DHCR7 | Q9UBM7 | 31336.94069 |
| 3045 | MBD2 | Q9UBB5 | 31335.02617 |
| 3046 | PDPK1 | O15530 | 31326.95772 |
| 3047 | MCCC2 | Q9HCC0 | 31309.42246 |
| 3048 | FILIP1L | Q4L180 | 31268.45356 |
| 3049 | AASDHPPT | Q9NRN7 | 31264.2958 |
| 3050 | BPGM | P07738 | 31229.29707 |
| 3051 | TRIP12 | Q14669 | 31222.56373 |
| 3052 | RHOA | P61586 | 31204.48833 |
| 3053 | MMUT | P22033 | 31163.59361 |
| 3054 | CLUH | O75153 | 31142.90894 |
| 3055 | ZRANB2 | O95218 | 31140.37614 |
| 3056 | IMUP | Q9GZP8 | 31126.9326 |
| 3057 | GTF3C5 | Q9Y5Q8 | 31103.30088 |
| 3058 | CTHRC1 | Q96CG8 | 31063.48727 |
| 3059 | CCDC134 | Q9H6E4 | 31050.4659 |
| 3060 | SHPK | Q9UHJ6 | 31042.2599 |
| 3061 | NOL10 | Q9BSC4 | 31030.29077 |
| 3062 | NF2 | P35240 | 31022.81217 |
| 3063 | ARL6IP4 | Q66PJ3 | 30957.1568 |
| 3064 | C19orf53 | Q9UNZ5 | 30952.51283 |
| 3065 | SGPL1 | O95470 | 30908.43974 |
| 3066 | CD151 | P48509 | 30899.89008 |
| 3067 | ETFDH | Q16134 | 30887.47347 |
| 3068 | GSDME | O60443 | 30874.2936 |
| 3069 | DDX41 | Q9UJV9 | 30874.23892 |
| 3070 | MAP4K5 | Q9Y4K4 | 30841.45332 |
| 3071 | SLAIN2 | Q9P270 | 30812.4277 |
| 3072 | NMT2 | O60551 | 30774.56167 |
| 3073 | PNMA2 | Q9UL42 | 30767.15306 |
| 3074 | SHANK3 | Q9BYB0 | 30750.91436 |
| 3075 | CCNT1 | O60563 | 30732.22767 |
| 3076 | GUK1 | Q16774 | 30730.00908 |
| 3077 | ROCK1 | Q13464 | 30703.26027 |
| 3078 | TMEM106B | Q9NUM4 | 30682.82314 |
| 3079 | LEO1 | Q8WVC0 | 30660.82329 |
| 3080 | RBM3 | P98179 | 30656.71867 |
| 3081 | TUBGCP3 | Q96CW5 | 30599.1417 |
| 3082 | ALG11 | Q2TAA5 | 30587.2192 |
| 3083 | GMPR2 | Q9P2T1 | 30569.18 |
| 3084 | RFLNB | Q8N5W9 | 30489.21875 |
| 3085 | GSKIP | Q9P0R6 | 30485.97267 |
| 3086 | MCFD2 | Q8NI22 | 30485.2515 |
| 3087 | BGN | P21810 | 30449.97835 |
| 3088 | FBXL18 | Q96ME1 | 30435.88321 |
| 3089 | UBE3C | Q15386 | 30411.01445 |
| 3090 | NOL6 | Q9H6R4 | 30386.9903 |
| 3091 | CHMP7 | Q8WUX9 | 30370.89504 |
| 3092 | RAB5B | P61020 | 30317.56036 |
| 3093 | NDC80 | O14777 | 30282.65996 |
| 3094 | SHOC2 | Q9UQ13 | 30271.58721 |
| 3095 | M6PR | P20645 | 30266.28613 |
| 3096 | GOLPH3L | Q9H4A5 | 30237.37688 |
| 3097 | TRAM1 | Q15629 | 30219.55558 |
| 3098 | UBXN7 | O94888 | 30207.58443 |
| 3099 | MEAK7 | Q6P9B6 | 30206.59484 |
| 3100 | MCMBP | Q9BTE3 | 30203.7897 |
| 3101 | ACAT2 | Q9BWD1 | 30202.46817 |
| 3102 | LUC7L3 | O95232 | 30162.84178 |
| 3103 | EXOSC5 | Q9NQT4 | 30087.2417 |
| 3104 | TNFRSF10B | O14763 | 30076.4635 |
| 3105 | SEH1L | Q96EE3 | 30041.44755 |
| 3106 | LARP4B | Q92615 | 30017.98738 |
| 3107 | HTRA2 | O43464 | 30014.80178 |
| 3108 | RFC3 | P40938 | 29978.35809 |
| 3109 | PDP1 | Q9P0J1 | 29919.24083 |
| 3110 | GALNT7 | Q86SF2 | 29889.05297 |
| 3111 | LTF | P02788 | 29887.23696 |
| 3112 | MYO9B | Q13459 | 29871.53959 |
| 3113 | RBM7 | Q9Y580 | 29838.19933 |
| 3114 | RASA1 | P20936 | 29806.2448 |
| 3115 | GJA5 | P36382 | 29792.04441 |
| 3116 | FAF1 | Q9UNN5 | 29773.13204 |
| 3117 | EXOC6B | Q9Y2D4 | 29771.49226 |
| 3118 | ABHD16A | O95870 | 29771.13169 |
| 3119 | EML4 | Q9HC35 | 29759.24355 |
| 3120 | PCCB | P05166 | 29746.57726 |
| 3121 | EXOC1 | Q9NV70 | 29746.54851 |
| 3122 | FOXK1 | P85037 | 29721.51706 |
| 3123 | AIMP2 | Q13155 | 29711.95456 |
| 3124 | CYS1 | Q717R9 | 29702.93571 |
| 3125 | ADAM17 | P78536 | 29693.77964 |
| 3126 | GLYR1 | Q49A26 | 29652.29825 |
| 3127 | CYP20A1 | Q6UW02 | 29642.33266 |
| 3128 | WBP2 | Q969T9 | 29639.08609 |
| 3129 | CKLF | Q9UBR5 | 29628.79667 |
| 3130 | STAT6 | P42226 | 29612.50848 |
| 3131 | MFAP1 | P55081 | 29607.26823 |
| 3132 | COG6 | Q9Y2V7 | 29541.53312 |
| 3133 | DDX54 | Q8TDD1 | 29523.92577 |
| 3134 | PLA2G15 | Q8NCC3 | 29521.3164 |
| 3135 | PTPRF | P10586 | 29467.14592 |
| 3136 | MFN2 | O95140 | 29415.23635 |
| 3137 | RGL2 | O15211 | 29410.66872 |
| 3138 | GRWD1 | Q9BQ67 | 29395.28779 |
| 3139 | MCM4 | P33991 | 29371.51204 |
| 3140 | YTHDF1 | Q9BYJ9 | 29329.01439 |
| 3141 | KDM1A | O60341 | 29322.06703 |
| 3142 | GTPBP1 | O00178 | 29219.17562 |
| 3143 | BRIX1 | Q8TDN6 | 29203.58584 |
| 3144 | ACSS2 | Q9NR19 | 29197.19089 |
| 3145 | MAN2B1 | O00754 | 29186.66092 |
| 3146 | NFKB1 | P19838 | 29179.5325 |
| 3147 | GIMAP1 | Q8WWP7 | 29045.96268 |
| 3148 | UBAP2 | Q5T6F2 | 29044.38463 |
| 3149 | TECR | Q9NZ01 | 29032.20607 |
| 3150 | RFC2 | P35250 | 29024.59673 |
| 3151 | STK10 | O94804 | 28970.10724 |
| 3152 | TYMP | P19971 | 28967.91008 |
| 3153 | PAF1 | Q8N7H5 | 28954.4384 |
| 3154 | CNPY3 | Q9BT09 | 28942.84593 |
| 3155 | CD2BP2 | O95400 | 28922.82781 |
| 3156 | PREB | Q9HCU5 | 28891.24978 |
| 3157 | SHC1 | P29353 | 28884.0495 |
| 3158 | STYX | Q8WUJ0 | 28882.11693 |
| 3159 | ACOT1 | Q86TX2 | 28865.93964 |
| 3160 | PPM1B | O75688 | 28848.31448 |
| 3161 | CD200 | P41217 | 28830.9724 |
| 3162 | TMEM181 | Q9P2C4 | 28826.7726 |
| 3163 | TBCE | Q15813 | 28727.52265 |
| 3164 | QRICH1 | Q2TAL8 | 28726.19642 |
| 3165 | SNX27 | Q96L92 | 28692.19652 |
| 3166 | ELMO1 | Q92556 | 28682.11098 |
| 3167 | PRCC | Q92733 | 28681.7002 |
| 3168 | TBC1D5 | Q92609 | 28674.37868 |
| 3169 | HLA-A | P04439 | 28653.3482 |
| 3170 | HSF1 | Q00613 | 28634.18822 |
| 3171 | MRFAP1 | Q9Y605 | 28626.6848 |
| 3172 | TGFB2 | P61812 | 28591.38616 |
| 3173 | GRAP | Q13588 | 28589.54146 |
| 3174 | CYB561D2 | O14569 | 28533.681 |
| 3175 | SH3KBP1 | Q96B97 | 28505.91849 |
| 3176 | ZC3H7B | Q9UGR2 | 28482.10469 |
| 3177 | PHF6 | Q8IWS0 | 28451.95605 |
| 3178 | SNX15 | Q9NRS6 | 28430.7572 |
| 3179 | TRAPPC2L | Q9UL33 | 28424.88167 |
| 3180 | VPS35L | Q7Z3J2 | 28418.93154 |
| 3181 | LAPTM4A | Q15012 | 28414.49 |
| 3182 | FYTTD1 | Q96QD9 | 28402.07867 |
| 3183 | DCUN1D1 | Q96GG9 | 28380.891 |
| 3184 | HERC4 | Q5GLZ8 | 28379.02304 |
| 3185 | AKR1E2 | Q96JD6 | 28376.40118 |
| 3186 | GSTT1 | P30711 | 28373.04242 |
| 3187 | SURF1 | Q15526 | 28290.06765 |
| 3188 | GORASP1 | Q9BQQ3 | 28259.366 |
| 3189 | YES1 | P07947 | 28245.4226 |
| 3190 | TRABD | Q9H4I3 | 28203.14265 |
| 3191 | STK38L | Q9Y2H1 | 28182.8756 |
| 3192 | RABGAP1 | Q9Y3P9 | 28170.98948 |
| 3193 | UBE2D3 | P61077 | 28104.00625 |
| 3194 | ARHGAP31 | Q2M1Z3 | 28073.01041 |
| 3195 | LGALSL | Q3ZCW2 | 28043.033 |
| 3196 | LAS1L | Q9Y4W2 | 28023.30466 |
| 3197 | BECN1 | Q14457 | 28000.16582 |
| 3198 | CLCN7 | P51798 | 27991.52083 |
| 3199 | SELENOO | Q9BVL4 | 27984.31043 |
| 3200 | NMD3 | Q96D46 | 27938.08108 |
| 3201 | ISCA1 | Q9BUE6 | 27908.71163 |
| 3202 | CTDSP1 | Q9GZU7 | 27893.8009 |
| 3203 | GPKOW | Q92917 | 27872.72017 |
| 3204 | ITPR2 | Q14571 | 27867.34171 |
| 3205 | FMR1 | Q06787 | 27845.60532 |
| 3206 | ZDHHC13 | Q8IUH4 | 27822.72273 |
| 3207 | MRPS14 | O60783 | 27817.73286 |
| 3208 | TANC1 | Q9C0D5 | 27801.46049 |
| 3209 | NMNAT1 | Q9HAN9 | 27792.047 |
| 3210 | AGFG2 | O95081 | 27789.94586 |
| 3211 | SCYL2 | Q6P3W7 | 27759.11002 |
| 3212 | CALHM5 | Q8N5C1 | 27755.90292 |
| 3213 | USP8 | P40818 | 27744.51025 |
| 3214 | TMX2 | Q9Y320 | 27727.17759 |
| 3215 | ZNF326 | Q5BKZ1 | 27719.69064 |
| 3216 | ALKBH5 | Q6P6C2 | 27718.53483 |
| 3217 | ITSN1 | Q15811 | 27686.51118 |
| 3218 | GPHN | Q9NQX3 | 27684.8237 |
| 3219 | IRAK4 | Q9NWZ3 | 27676.50793 |
| 3220 | EGLN1 | Q9GZT9 | 27651.01272 |
| 3221 | CD58 | P19256 | 27632.72763 |
| 3222 | TSPAN9 | O75954 | 27617.12978 |
| 3223 | FGD5 | Q6ZNL6 | 27590.33986 |
| 3224 | TPMT | P51580 | 27469.75421 |
| 3225 | EFEMP1 | Q12805 | 27463.43135 |
| 3226 | LAMB2 | P55268 | 27449.34689 |
| 3227 | ATG16L1 | Q676U5 | 27448.06913 |
| 3228 | SELENOI | Q9C0D9 | 27383.78089 |
| 3229 | LEMD3 | Q9Y2U8 | 27360.73078 |
| 3230 | TMEM50B | P56557 | 27354.02667 |
| 3231 | CUTC | Q9NTM9 | 27352.39323 |
| 3232 | CCNYL1 | Q8N7R7 | 27309.23094 |
| 3233 | NRF1 | Q16656 | 27278.078 |
| 3234 | LRP10 | Q7Z4F1 | 27184.58492 |
| 3235 | EIF2D | P41214 | 27176.78906 |
| 3236 | SRGAP2 | O75044 | 27136.24528 |
| 3237 | MMS19 | Q96T76 | 27070.15248 |
| 3238 | INTS11 | Q5TA45 | 27033.20955 |
| 3239 | ATP2C1 | P98194 | 26998.52717 |
| 3240 | AHCTF1 | Q8WYP5 | 26996.30952 |
| 3241 | ACADSB | P45954 | 26941.05178 |
| 3242 | CAAP1 | Q9H8G2 | 26909.71 |
| 3243 | ASAP1 | Q9ULH1 | 26907.25531 |
| 3244 | FGD4 | Q96M96 | 26907.06229 |
| 3245 | YIPF6 | Q96EC8 | 26892.07444 |
| 3246 | DCTPP1 | Q9H773 | 26878.30964 |
| 3247 | APPL2 | Q8NEU8 | 26852.91558 |
| 3248 | MOV10 | Q9HCE1 | 26827.5324 |
| 3249 | COMMD8 | Q9NX08 | 26815.0797 |
| 3250 | NIT1 | Q86X76 | 26770.22111 |
| 3251 | STAMBP | O95630 | 26724.2869 |
| 3252 | RBM5 | P52756 | 26686.48137 |
| 3253 | ARL2BP | Q9Y2Y0 | 26680.05713 |
| 3254 | HSD17B7 | P56937 | 26676.77133 |
| 3255 | S100A7 | P31151 | 26619.74143 |
| 3256 | CUSTOS | Q96C57 | 26612.50657 |
| 3257 | ZNF593 | O00488 | 26611.0045 |
| 3258 | ITGA10 | O75578 | 26585.54663 |
| 3259 | SLC12A9 | Q9BXP2 | 26573.51394 |
| 3260 | FNBP1L | Q5T0N5 | 26569.54076 |
| 3261 | APOC3 | P02656 | 26556.1945 |
| 3262 | GTF2F2 | P13984 | 26547.63157 |
| 3263 | EEF2K | O00418 | 26547.37843 |
| 3264 | TRUB1 | Q8WWH5 | 26543.30433 |
| 3265 | CAMK2G | Q13555 | 26537.57885 |
| 3266 | CDK4 | P11802 | 26518.85425 |
| 3267 | UBE2O | Q9C0C9 | 26517.05615 |
| 3268 | CWC15 | Q9P013 | 26507.72138 |
| 3269 | MKRN2 | Q9H000 | 26504.42661 |
| 3270 | RIPK1 | Q13546 | 26475.57588 |
| 3271 | CLASP1 | Q7Z460 | 26442.43523 |
| 3272 | PPP1CC | P36873 | 26427.40088 |
| 3273 | B4GALT7 | Q9UBV7 | 26418.67163 |
| 3274 | ANO6 | Q4KMQ2 | 26376.6906 |
| 3275 | ISCA2 | Q86U28 | 26359.70117 |
| 3276 | BORCS5 | Q969J3 | 26339.10615 |
| 3277 | CC2D1B | Q5T0F9 | 26327.12868 |
| 3278 | ATP2B4 | P23634 | 26316.68042 |
| 3279 | RFLNA | Q6ZTI6 | 26287.22845 |
| 3280 | SDHD | O14521 | 26272.9115 |
| 3281 | RNASEH2A | O75792 | 26262.32673 |
| 3282 | NUDT14 | O95848 | 26260.41627 |
| 3283 | DNAJB12 | Q9NXW2 | 26256.93406 |
| 3284 | VWA5A | O00534 | 26236.23013 |
| 3285 | MRC2 | Q9UBG0 | 26214.50888 |
| 3286 | JMJD6 | Q6NYC1 | 26212.99214 |
| 3287 | LGALS8 | O00214 | 26197.62584 |
| 3288 | NACC1 | Q96RE7 | 26192.5543 |
| 3289 | B4GALT1 | P15291 | 26157.43838 |
| 3290 | KANK2 | Q63ZY3 | 26152.69557 |
| 3291 | CYC1 | P08574 | 26145.38719 |
| 3292 | BID | P55957 | 26132.27735 |
| 3293 | PIP5K1C | O60331 | 26102.26278 |
| 3294 | POLR1E | Q9GZS1 | 26075.80748 |
| 3295 | COG3 | Q96JB2 | 26046.25304 |
| 3296 | GGCX | P38435 | 26041.67247 |
| 3297 | TNPO3 | Q9Y5L0 | 26041.01449 |
| 3298 | DNAJC11 | Q9NVH1 | 26040.85378 |
| 3299 | MARCHF5 | Q9NX47 | 26028.84294 |
| 3300 | PRTFDC1 | Q9NRG1 | 25954.654 |
| 3301 | DDX19A | Q9NUU7 | 25946.83322 |
| 3302 | CPSF2 | Q9P2I0 | 25930.01784 |
| 3303 | TNFAIP8L3 | Q5GJ75 | 25906.99667 |
| 3304 | MPHOSPH10 | O00566 | 25898.61044 |
| 3305 | TMUB1 | Q9BVT8 | 25858.658 |
| 3306 | EXOSC1 | Q9Y3B2 | 25843.481 |
| 3307 | NT5C2 | P49902 | 25778.9973 |
| 3308 | CCDC127 | Q96BQ5 | 25771.04473 |
| 3309 | CPQ | Q9Y646 | 25756.4069 |
| 3310 | ITGA6 | P23229 | 25750.27682 |
| 3311 | CDK9 | P50750 | 25748.36083 |
| 3312 | DST | Q03001 | 25746.83222 |
| 3313 | ARMC9 | Q7Z3E5 | 25721.31406 |
| 3314 | EDC3 | Q96F86 | 25694.14397 |
| 3315 | ASCC1 | Q8N9N2 | 25687.16031 |
| 3316 | KIF1C | O43896 | 25679.42426 |
| 3317 | RAD21 | O60216 | 25679.17007 |
| 3318 | TIMP1 | P01033 | 25632.73464 |
| 3319 | MED18 | Q9BUE0 | 25607.83857 |
| 3320 | FOCAD | Q5VW36 | 25599.96 |
| 3321 | NEDD1 | Q8NHV4 | 25561.00242 |
| 3322 | CNOT7 | Q9UIV1 | 25548.142 |
| 3323 | GPN3 | Q9UHW5 | 25535.71209 |
| 3324 | PIGG | Q5H8A4 | 25512.17225 |
| 3325 | ALG9 | Q9H6U8 | 25497.77541 |
| 3326 | NPTN | Q9Y639 | 25489.92043 |
| 3327 | NUDT16 | Q96DE0 | 25445.19462 |
| 3328 | DNMBP | Q6XZF7 | 25361.48498 |
| 3329 | PC | P11498 | 25340.40388 |
| 3330 | HSBP1 | O75506 | 25331.0874 |
| 3331 | CDH11 | P55287 | 25325.83354 |
| 3332 | YTHDF3 | Q7Z739 | 25323.32289 |
| 3333 | ADO | Q96SZ5 | 25318.3005 |
| 3334 | NFIB | O00712 | 25314.4759 |
| 3335 | POLR2A | P24928 | 25302.98373 |
| 3336 | VPS51 | Q9UID3 | 25302.23832 |
| 3337 | ALDH1L2 | Q3SY69 | 25290.18139 |
| 3338 | MTA1 | Q13330 | 25283.71558 |
| 3339 | INTS13 | Q9NVM9 | 25267.95521 |
| 3340 | KEAP1 | Q14145 | 25259.35563 |
| 3341 | TSPAN14 | Q8NG11 | 25258.08462 |
| 3342 | EVI5 | O60447 | 25239.58362 |
| 3343 | RBM28 | Q9NW13 | 25216.43225 |
| 3344 | DNAJC2 | Q99543 | 25209.04568 |
| 3345 | HDDC2 | Q7Z4H3 | 25186.52236 |
| 3346 | EPHB4 | P54760 | 25182.58114 |
| 3347 | DOCK1 | Q14185 | 25182.11876 |
| 3348 | RPS19BP1 | Q86WX3 | 25177.61113 |
| 3349 | UBQLN2 | Q9UHD9 | 25153.81019 |
| 3350 | MTX2 | O75431 | 25119.2725 |
| 3351 | EIF4G3 | O43432 | 25117.95895 |
| 3352 | TBC1D23 | Q9NUY8 | 25117.83894 |
| 3353 | PTPMT1 | Q8WUK0 | 25114.20943 |
| 3354 | SPG21 | Q9NZD8 | 25086.72078 |
| 3355 | SLC52A2 | Q9HAB3 | 25062.27375 |
| 3356 | BORCS7 | Q96B45 | 25051.16033 |
| 3357 | PDPR | Q8NCN5 | 25043.00355 |
| 3358 | UBTD1 | Q9HAC8 | 25037.25556 |
| 3359 | SMARCA1 | P28370 | 25028.02866 |
| 3360 | CASP6 | P55212 | 24979.39381 |
| 3361 | DHX37 | Q8IY37 | 24920.16313 |
| 3362 | TBCD | Q9BTW9 | 24909.54172 |
| 3363 | FMC1 | Q96HJ9 | 24863.70667 |
| 3364 | BCKDHA | P12694 | 24843.86469 |
| 3365 | GSTA3 | Q16772 | 24750.42083 |
| 3366 | ME1 | P48163 | 24742.03301 |
| 3367 | HEATR1 | Q9H583 | 24725.35924 |
| 3368 | TRIP11 | Q15643 | 24718.05947 |
| 3369 | IFIT5 | Q13325 | 24692.79522 |
| 3370 | COG4 | Q9H9E3 | 24690.16426 |
| 3371 | DDX39A | O00148 | 24644.86322 |
| 3372 | REPS1 | Q96D71 | 24641.45642 |
| 3373 | RPP38 | P78345 | 24606.17556 |
| 3374 | CGNL1 | Q0VF96 | 24584.99544 |
| 3375 | RNF20 | Q5VTR2 | 24581.25151 |
| 3376 | BET1L | Q9NYM9 | 24575.516 |
| 3377 | LSM14B | Q9BX40 | 24574.00376 |
| 3378 | TRAPPC1 | Q9Y5R8 | 24566.0348 |
| 3379 | CGGBP1 | Q9UFW8 | 24550.51725 |
| 3380 | KXD1 | Q9BQD3 | 24546.7875 |
| 3381 | HABP2 | Q14520 | 24534.67667 |
| 3382 | MRPL50 | Q8N5N7 | 24530.51256 |
| 3383 | DHX16 | O60231 | 24519.1172 |
| 3384 | NDRG4 | Q9ULP0 | 24501.14236 |
| 3385 | SEL1L | Q9UBV2 | 24484.80464 |
| 3386 | PEX11B | O96011 | 24456.39607 |
| 3387 | GEMIN6 | Q8WXD5 | 24447.33933 |
| 3388 | CMC1 | Q7Z7K0 | 24443.115 |
| 3389 | HIGD2A | Q9BW72 | 24440.73533 |
| 3390 | WIPF2 | Q8TF74 | 24430.65959 |
| 3391 | HYPK | Q9NX55 | 24424.12683 |
| 3392 | MSH6 | P52701 | 24402.08964 |
| 3393 | GATC | O43716 | 24368.71 |
| 3394 | METTL3 | Q86U44 | 24367.70515 |
| 3395 | XRN1 | Q8IZH2 | 24359.1192 |
| 3396 | FAM172A | Q8WUF8 | 24348.27775 |
| 3397 | METTL7A | Q9H8H3 | 24321.9998 |
| 3398 | UNC119B | A6NIH7 | 24311.18733 |
| 3399 | EXOSC9 | Q06265 | 24309.31022 |
| 3400 | MRPL55 | Q7Z7F7 | 24296.3875 |
| 3401 | PIK3R4 | Q99570 | 24210.57909 |
| 3402 | DHRS7B | Q6IAN0 | 24209.41835 |
| 3403 | SRA1 | Q9HD15 | 24116.48233 |
| 3404 | ZCCHC8 | Q6NZY4 | 24099.01518 |
| 3405 | AAGAB | Q6PD74 | 24097.31942 |
| 3406 | CTBP2 | P56545 | 24079.01375 |
| 3407 | LMF2 | Q9BU23 | 24032.45684 |
| 3408 | MRPL27 | Q9P0M9 | 23999.5925 |
| 3409 | CBL | P22681 | 23998.64227 |
| 3410 | NOC3L | Q8WTT2 | 23994.26283 |
| 3411 | NUDT16L1 | Q9BRJ7 | 23986.803 |
| 3412 | STEEP1 | Q9H5V9 | 23983.30733 |
| 3413 | XXYLT1 | Q8NBI6 | 23972.96272 |
| 3414 | GPN1 | Q9HCN4 | 23964.7068 |
| 3415 | WDR37 | Q9Y2I8 | 23936.67826 |
| 3416 | WDR26 | Q9H7D7 | 23924.1296 |
| 3417 | MAPK8 | P45983 | 23910.35895 |
| 3418 | LSM14A | Q8ND56 | 23838.14537 |
| 3419 | TMEM201 | Q5SNT2 | 23836.96858 |
| 3420 | ACVRL1 | P37023 | 23828.86388 |
| 3421 | F5 | P12259 | 23816.72543 |
| 3422 | ECSCR | Q19T08 | 23810.33667 |
| 3423 | HPF1 | Q9NWY4 | 23788.47239 |
| 3424 | CHID1 | Q9BWS9 | 23741.93238 |
| 3425 | ATP6V0A1 | Q93050 | 23740.84603 |
| 3426 | ARSB | P15848 | 23722.28462 |
| 3427 | POLR1F | Q3B726 | 23675.29154 |
| 3428 | LTV1 | Q96GA3 | 23674.81705 |
| 3429 | ARMC6 | Q6NXE6 | 23658.30065 |
| 3430 | ATP6V0C | P27449 | 23633.23617 |
| 3431 | ITPR3 | Q14573 | 23631.5023 |
| 3432 | CERS2 | Q96G23 | 23623.87433 |
| 3433 | SDR39U1 | Q9NRG7 | 23616.29864 |
| 3434 | GOLM1 | Q8NBJ4 | 23607.89296 |
| 3435 | RRP12 | Q5JTH9 | 23602.84154 |
| 3436 | P4HTM | Q9NXG6 | 23598.51837 |
| 3437 | PELP1 | Q8IZL8 | 23593.90178 |
| 3438 | TRAF2 | Q12933 | 23590.38896 |
| 3439 | LARS2 | Q15031 | 23549.13167 |
| 3440 | GLA | P06280 | 23537.35008 |
| 3441 | FEZ2 | Q9UHY8 | 23530.17475 |
| 3442 | PLEK2 | Q9NYT0 | 23520.06885 |
| 3443 | NRAS | P01111 | 23519.367 |
| 3444 | HSPA14 | Q0VDF9 | 23513.76336 |
| 3445 | KNTC1 | P50748 | 23512.72478 |
| 3446 | HECTD3 | Q5T447 | 23482.51779 |
| 3447 | VPS26C | O14972 | 23475.15247 |
| 3448 | MRPS9 | P82933 | 23417.82892 |
| 3449 | JMJD8 | Q96S16 | 23402.82 |
| 3450 | TRIM22 | Q8IYM9 | 23397.16465 |
| 3451 | GNB2 | P62879 | 23371.82907 |
| 3452 | SMAP2 | Q8WU79 | 23350.916 |
| 3453 | ARMCX1 | Q9P291 | 23324.2888 |
| 3454 | CLEC3B | P05452 | 23300.1248 |
| 3455 | APLP2 | Q06481 | 23270.12689 |
| 3456 | MLKL | Q8NB16 | 23263.57548 |
| 3457 | HMGCL | P35914 | 23261.6475 |
| 3458 | TOX4 | O94842 | 23219.51287 |
| 3459 | CNOT2 | Q9NZN8 | 23210.18273 |
| 3460 | SH3D19 | Q5HYK7 | 23166.35174 |
| 3461 | TRAPPC6B | Q86SZ2 | 23159.31085 |
| 3462 | SIRT2 | Q8IXJ6 | 23158.25605 |
| 3463 | PPWD1 | Q96BP3 | 23157.58168 |
| 3464 | MRPL2 | Q5T653 | 23113.826 |
| 3465 | PDS5A | Q29RF7 | 23078.83981 |
| 3466 | PRPF4B | Q13523 | 23059.15854 |
| 3467 | PTGIS | Q16647 | 23040.284 |
| 3468 | MOCS2 | O96033 | 23028.765 |
| 3469 | EMC10 | Q5UCC4 | 23015.78556 |
| 3470 | MRPL40 | Q9NQ50 | 23013.4353 |
| 3471 | RPRD1A | Q96P16 | 23007.99717 |
| 3472 | CWF19L1 | Q69YN2 | 22991.7833 |
| 3473 | LYAR | Q9NX58 | 22974.04311 |
| 3474 | NDUFS6 | O75380 | 22934.364 |
| 3475 | FXN | Q16595 | 22925.7988 |
| 3476 | DEGS1 | O15121 | 22922.4872 |
| 3477 | PAK1IP1 | Q9NWT1 | 22906.3366 |
| 3478 | PPIL2 | Q13356 | 22904.67003 |
| 3479 | PRKCD | Q05655 | 22887.89797 |
| 3480 | PTDSS2 | Q9BVG9 | 22878.09095 |
| 3481 | ASB6 | Q9NWX5 | 22867.49656 |
| 3482 | PRKAB1 | Q9Y478 | 22864.97543 |
| 3483 | NOC4L | Q9BVI4 | 22863.17714 |
| 3484 | CNN1 | P51911 | 22825.01963 |
| 3485 | PANK4 | Q9NVE7 | 22769.00374 |
| 3486 | SMG9 | Q9H0W8 | 22762.37826 |
| 3487 | VAC14 | Q08AM6 | 22760.51008 |
| 3488 | ELAC2 | Q9BQ52 | 22751.11376 |
| 3489 | C1QTNF5 | Q9BXJ0 | 22728.60883 |
| 3490 | CCNK | O75909 | 22724.25482 |
| 3491 | NOB1 | Q9ULX3 | 22719.23287 |
| 3492 | TUBG1 | P23258 | 22695.47618 |
| 3493 | SPNS1 | Q9H2V7 | 22616.86835 |
| 3494 | CHPF2 | Q9P2E5 | 22610.88854 |
| 3495 | ERMP1 | Q7Z2K6 | 22601.91861 |
| 3496 | SYF2 | O95926 | 22601.30567 |
| 3497 | ALDH1L1 | O75891 | 22593.18365 |
| 3498 | LYSMD2 | Q8IV50 | 22587.5954 |
| 3499 | COG1 | Q8WTW3 | 22541.46955 |
| 3500 | DCAKD | Q8WVC6 | 22538.05423 |
| 3501 | CDC42EP5 | Q6NZY7 | 22534.86771 |
| 3502 | TBXAS1 | P24557 | 22513.09386 |
| 3503 | CWC27 | Q6UX04 | 22510.66035 |
| 3504 | DCP1A | Q9NPI6 | 22498.78033 |
| 3505 | REEP3 | Q6NUK4 | 22475.44769 |
| 3506 | NOL9 | Q5SY16 | 22465.07091 |
| 3507 | WIZ | O95785 | 22456.43603 |
| 3508 | DPH5 | Q9H2P9 | 22420.15408 |
| 3509 | TRMT2A | Q8IZ69 | 22388.62863 |
| 3510 | NUP58 | Q9BVL2 | 22385.60729 |
| 3511 | GOLGA7 | Q7Z5G4 | 22367.72889 |
| 3512 | KANSL3 | Q9P2N6 | 22362.79103 |
| 3513 | AGPAT1 | Q99943 | 22355.86371 |
| 3514 | MECR | Q9BV79 | 22317.836 |
| 3515 | ARHGEF12 | Q9NZN5 | 22315.72921 |
| 3516 | HDAC7 | Q8WUI4 | 22273.98962 |
| 3517 | DDX58 | O95786 | 22254.4256 |
| 3518 | OS9 | Q13438 | 22237.89261 |
| 3519 | NCK2 | O43639 | 22207.38626 |
| 3520 | RPL26 | P61254 | 22206.08167 |
| 3521 | EHBP1L1 | Q8N3D4 | 22166.58519 |
| 3522 | SLC30A1 | Q9Y6M5 | 22163.24958 |
| 3523 | AGO2 | Q9UKV8 | 22156.91898 |
| 3524 | SIL1 | Q9H173 | 22132.08641 |
| 3525 | PELO | Q9BRX2 | 22123.70749 |
| 3526 | PNO1 | Q9NRX1 | 22112.48571 |
| 3527 | RUFY2 | Q8WXA3 | 22082.53885 |
| 3528 | POP1 | Q99575 | 22072.48881 |
| 3529 | CCDC115 | Q96NT0 | 22071.09356 |
| 3530 | RILPL1 | Q5EBL4 | 22049.67848 |
| 3531 | RNF25 | Q96BH1 | 21995.22265 |
| 3532 | AK5 | Q9Y6K8 | 21993.90881 |
| 3533 | STK4 | Q13043 | 21993.18638 |
| 3534 | PRKD3 | O94806 | 21967.74928 |
| 3535 | ERAL1 | O75616 | 21959.29771 |
| 3536 | DNTTIP2 | Q5QJE6 | 21956.60454 |
| 3537 | PNKP | Q96T60 | 21953.8379 |
| 3538 | VTI1A | Q96AJ9 | 21942.28827 |
| 3539 | SPHK1 | Q9NYA1 | 21941.9815 |
| 3540 | SURF6 | O75683 | 21931.54944 |
| 3541 | NDUFB7 | P17568 | 21910.89143 |
| 3542 | PCYOX1L | Q8NBM8 | 21896.04184 |
| 3543 | MED11 | Q9P086 | 21872.01143 |
| 3544 | TIE1 | P35590 | 21856.87045 |
| 3545 | COG7 | P83436 | 21806.46178 |
| 3546 | ANAPC7 | Q9UJX3 | 21803.8017 |
| 3547 | ERG28 | Q9UKR5 | 21794.245 |
| 3548 | TRIM56 | Q9BRZ2 | 21791.95344 |
| 3549 | RFT1 | Q96AA3 | 21789.455 |
| 3550 | NTN4 | Q9HB63 | 21767.81519 |
| 3551 | USP48 | Q86UV5 | 21761.88915 |
| 3552 | NELFCD | Q8IXH7 | 21743.14767 |
| 3553 | NDUFAB1 | O14561 | 21729.18425 |
| 3554 | CHST14 | Q8NCH0 | 21726.51365 |
| 3555 | RRP1 | P56182 | 21696.77772 |
| 3556 | UBE4A | Q14139 | 21676.69788 |
| 3557 | EHD3 | Q9NZN3 | 21672.87863 |
| 3558 | CNTNAP3 | Q9BZ76 | 21661.36542 |
| 3559 | DNAJC17 | Q9NVM6 | 21660.11047 |
| 3560 | VPS33A | Q96AX1 | 21633.31794 |
| 3561 | FUCA1 | P04066 | 21622.10045 |
| 3562 | RIOX1 | Q9H6W3 | 21612.98716 |
| 3563 | PDS5B | Q9NTI5 | 21606.46888 |
| 3564 | TMEM35B | Q8NCS4 | 21600.402 |
| 3565 | CAB39L | Q9H9S4 | 21590.28286 |
| 3566 | CYBRD1 | Q53TN4 | 21572.69024 |
| 3567 | CDK2 | P24941 | 21567.86339 |
| 3568 | DDX50 | Q9BQ39 | 21561.86347 |
| 3569 | WDR74 | Q6RFH5 | 21553.58591 |
| 3570 | SMPD4 | Q9NXE4 | 21536.90728 |
| 3571 | DIP2B | Q9P265 | 21509.99596 |
| 3572 | C4orf36 | Q96KX1 | 21507.8675 |
| 3573 | SMARCE1 | Q969G3 | 21490.06525 |
| 3574 | PKIG | Q9Y2B9 | 21478.367 |
| 3575 | INPP5K | Q9BT40 | 21435.51442 |
| 3576 | CNOT8 | Q9UFF9 | 21423.05431 |
| 3577 | MIEF1 | L0R8F8 | 21414.275 |
| 3578 | CCDC9 | Q9Y3X0 | 21398.41026 |
| 3579 | ITPK1 | Q13572 | 21397.93544 |
| 3580 | PAK1 | Q13153 | 21375.476 |
| 3581 | HRAS | P01112 | 21363.903 |
| 3582 | CTDNEP1 | O95476 | 21314.48545 |
| 3583 | TTC5 | Q8N0Z6 | 21307.42096 |
| 3584 | EXD2 | Q9NVH0 | 21241.41716 |
| 3585 | SPAST | Q9UBP0 | 21220.09229 |
| 3586 | ADAM9 | Q13443 | 21212.86409 |
| 3587 | ZDHHC20 | Q5W0Z9 | 21208.8125 |
| 3588 | SMPD1 | P17405 | 21178.11852 |
| 3589 | GLE1 | Q53GS7 | 21173.76169 |
| 3590 | UBQLN4 | Q9NRR5 | 21169.75237 |
| 3591 | PWP1 | Q13610 | 21155.18923 |
| 3592 | ADAM15 | Q13444 | 21149.63431 |
| 3593 | DNASE2 | O00115 | 21113.17647 |
| 3594 | FRYL | O94915 | 21076.27012 |
| 3595 | CCDC167 | Q9P0B6 | 21070.96 |
| 3596 | UHRF2 | Q96PU4 | 21063.75413 |
| 3597 | PI4K2A | Q9BTU6 | 21030.8775 |
| 3598 | MAPKAPK2 | P49137 | 21027.61504 |
| 3599 | BMS1 | Q14692 | 21014.04304 |
| 3600 | CCDC88A | Q3V6T2 | 21010.18757 |
| 3601 | TRIM47 | Q96LD4 | 20991.76393 |
| 3602 | GOLGA4 | Q13439 | 20987.47704 |
| 3603 | NELFA | Q9H3P2 | 20985.19022 |
| 3604 | MRPS30 | Q9NP92 | 20960.23806 |
| 3605 | LRRC8C | Q8TDW0 | 20950.6364 |
| 3606 | MAFF | Q9ULX9 | 20932.60675 |
| 3607 | FADS3 | Q9Y5Q0 | 20931.08188 |
| 3608 | CRELD1 | Q96HD1 | 20904.26952 |
| 3609 | GPC1 | P35052 | 20859.74029 |
| 3610 | FLAD1 | Q8NFF5 | 20848.73001 |
| 3611 | SLC4A2 | P04920 | 20841.4232 |
| 3612 | LIG3 | P49916 | 20835.46448 |
| 3613 | EPHB2 | P29323 | 20829.77502 |
| 3614 | FAM20B | O75063 | 20829.34111 |
| 3615 | DNAJB6 | O75190 | 20812.78131 |
| 3616 | NAF1 | Q96HR8 | 20783.181 |
| 3617 | MGLL | Q99685 | 20764.72971 |
| 3618 | METTL5 | Q9NRN9 | 20749.1733 |
| 3619 | LONP2 | Q86WA8 | 20747.61025 |
| 3620 | MRPS24 | Q96EL2 | 20747.331 |
| 3621 | GLMN | Q92990 | 20735.86693 |
| 3622 | ATG101 | Q9BSB4 | 20722.38382 |
| 3623 | RAD17 | O75943 | 20698.57784 |
| 3624 | GOLIM4 | O00461 | 20664.48326 |
| 3625 | CRNKL1 | Q9BZJ0 | 20659.80316 |
| 3626 | TMEM128 | Q5BJH2 | 20657.08733 |
| 3627 | SPATA5 | Q8NB90 | 20627.57719 |
| 3628 | TSSC4 | Q9Y5U2 | 20625.41078 |
| 3629 | STRIP1 | Q5VSL9 | 20625.37763 |
| 3630 | TMF1 | P82094 | 20623.71648 |
| 3631 | INPP5D | Q92835 | 20614.10983 |
| 3632 | CUL4A | Q13619 | 20610.57687 |
| 3633 | GALNS | P34059 | 20583.63957 |
| 3634 | DIPK2A | Q8NDZ4 | 20576.77689 |
| 3635 | OTULIN | Q96BN8 | 20570.93973 |
| 3636 | TATDN1 | Q6P1N9 | 20541.11032 |
| 3637 | PCYT2 | Q99447 | 20541.03078 |
| 3638 | IL6ST | P40189 | 20527.0776 |
| 3639 | ATG4A | Q8WYN0 | 20522.02688 |
| 3640 | UTP15 | Q8TED0 | 20496.15433 |
| 3641 | THUMPD3 | Q9BV44 | 20478.03528 |
| 3642 | VCPIP1 | Q96JH7 | 20465.98666 |
| 3643 | FSD1 | Q9BTV5 | 20455.7916 |
| 3644 | CHD1L | Q86WJ1 | 20449.62481 |
| 3645 | HDAC8 | Q9BY41 | 20448.82013 |
| 3646 | SYMPK | Q92797 | 20444.98033 |
| 3647 | ACBD6 | Q9BR61 | 20442.81577 |
| 3648 | STARD3NL | O95772 | 20417.5355 |
| 3649 | TBC1D22A | Q8WUA7 | 20409.09624 |
| 3650 | POGZ | Q7Z3K3 | 20386.50278 |
| 3651 | VPS18 | Q9P253 | 20367.97392 |
| 3652 | CLN5 | O75503 | 20356.28945 |
| 3653 | VPS53 | Q5VIR6 | 20336.64169 |
| 3654 | EEFSEC | P57772 | 20325.72434 |
| 3655 | DCAF13 | Q9NV06 | 20320.57375 |
| 3656 | NUDT9 | Q9BW91 | 20291.73067 |
| 3657 | NOL11 | Q9H8H0 | 20280.75048 |
| 3658 | COX16 | Q9P0S2 | 20256.34 |
| 3659 | TBC1D4 | O60343 | 20254.98972 |
| 3660 | NAA35 | Q5VZE5 | 20241.85261 |
| 3661 | STAT2 | P52630 | 20228.35216 |
| 3662 | DCUN1D5 | Q9BTE7 | 20211.63354 |
| 3663 | RNF121 | Q9H920 | 20209.47763 |
| 3664 | CANT1 | Q8WVQ1 | 20189.51622 |
| 3665 | GAMT | Q14353 | 20183.91233 |
| 3666 | GPD1L | Q8N335 | 20161.3267 |
| 3667 | SEPTIN6 | Q14141 | 20151.70769 |
| 3668 | SMARCA4 | P51532 | 20132.33324 |
| 3669 | MON2 | Q7Z3U7 | 20130.73681 |
| 3670 | THOC3 | Q96J01 | 20112.52813 |
| 3671 | ASS1 | P00966 | 20075.58323 |
| 3672 | C9orf64 | Q5T6V5 | 20071.27096 |
| 3673 | OVCA2 | Q8WZ82 | 20070.936 |
| 3674 | ITGB5 | P18084 | 20057.81055 |
| 3675 | MAGED1 | Q9Y5V3 | 20036.0765 |
| 3676 | ELOVL5 | Q9NYP7 | 19954.30125 |
| 3677 | PTGFRN | Q9P2B2 | 19931.84729 |
| 3678 | ACAP2 | Q15057 | 19928.60347 |
| 3679 | UFSP2 | Q9NUQ7 | 19927.89762 |
| 3680 | ERCC3 | P19447 | 19926.37876 |
| 3681 | TPK1 | Q9H3S4 | 19925.91675 |
| 3682 | NTAN1 | Q96AB6 | 19918.4829 |
| 3683 | NSA2 | O95478 | 19909.52158 |
| 3684 | AHCYL2 | Q96HN2 | 19906.10274 |
| 3685 | SUOX | P51687 | 19887.70444 |
| 3686 | SSNA1 | O43805 | 19876.44863 |
| 3687 | STEAP3 | Q658P3 | 19865.43357 |
| 3688 | SLC44A1 | Q8WWI5 | 19857.3147 |
| 3689 | HLA-E | P13747 | 19841.00339 |
| 3690 | SCFD2 | Q8WU76 | 19832.60752 |
| 3691 | ATP13A3 | Q9H7F0 | 19832.2759 |
| 3692 | HDDC3 | Q8N4P3 | 19823.28309 |
| 3693 | TCEAL4 | Q96EI5 | 19808.15086 |
| 3694 | PCBD2 | Q9H0N5 | 19802.79625 |
| 3695 | EVA1A | Q9H8M9 | 19775.07883 |
| 3696 | SLTM | Q9NWH9 | 19774.72145 |
| 3697 | RABGEF1 | Q9UJ41 | 19774.22326 |
| 3698 | ATL2 | Q8NHH9 | 19760.08654 |
| 3699 | HDGFL2 | Q7Z4V5 | 19759.68415 |
| 3700 | SLC23A3 | Q6PIS1 | 19727.45833 |
| 3701 | OGFR | Q9NZT2 | 19708.30082 |
| 3702 | IGF2R | P11717 | 19701.73776 |
| 3703 | LYN | P07948 | 19696.17596 |
| 3704 | PRRC2A | P48634 | 19681.50349 |
| 3705 | COG5 | Q9UP83 | 19678.35767 |
| 3706 | MINDY3 | Q9H8M7 | 19674.31556 |
| 3707 | SIRT5 | Q9NXA8 | 19668.78328 |
| 3708 | NXT1 | Q9UKK6 | 19615.05 |
| 3709 | ORMDL3 | Q8N138 | 19613.85917 |
| 3710 | CXADR | P78310 | 19585.62933 |
| 3711 | LYRM7 | Q5U5X0 | 19580.56367 |
| 3712 | GTF2E2 | P29084 | 19555.97383 |
| 3713 | CADM3 | Q8N126 | 19551.21367 |
| 3714 | RNF213 | Q63HN8 | 19543.29548 |
| 3715 | UBL5 | Q9BZL1 | 19528.875 |
| 3716 | TRAPPC12 | Q8WVT3 | 19519.11873 |
| 3717 | CEMIP2 | Q9UHN6 | 19519.03014 |
| 3718 | MAPK11 | Q15759 | 19504.3045 |
| 3719 | HSPA2 | P54652 | 19481.71614 |
| 3720 | ZNF638 | Q14966 | 19463.8379 |
| 3721 | UTP3 | Q9NQZ2 | 19453.24486 |
| 3722 | CLEC14A | Q86T13 | 19448.27404 |
| 3723 | WTAP | Q15007 | 19429.11664 |
| 3724 | TRIR | Q9BQ61 | 19428.10592 |
| 3725 | IDH3G | P51553 | 19427.90153 |
| 3726 | TMEM184C | Q9NVA4 | 19425.47538 |
| 3727 | NOP14 | P78316 | 19418.88335 |
| 3728 | FUT8 | Q9BYC5 | 19378.32316 |
| 3729 | TTC4 | O95801 | 19365.88757 |
| 3730 | CTIF | O43310 | 19365.37859 |
| 3731 | C5orf51 | A6NDU8 | 19352.87282 |
| 3732 | POLD2 | P49005 | 19341.75205 |
| 3733 | GHDC | Q8N2G8 | 19330.6565 |
| 3734 | PIH1D1 | Q9NWS0 | 19294.553 |
| 3735 | TNFRSF10D | Q9UBN6 | 19289.76245 |
| 3736 | STK38 | Q15208 | 19279.4024 |
| 3737 | SMARCC1 | Q92922 | 19267.53271 |
| 3738 | SREK1 | Q8WXA9 | 19265.6387 |
| 3739 | GALNT12 | Q8IXK2 | 19226.83846 |
| 3740 | SMYD3 | Q9H7B4 | 19223.33167 |
| 3741 | UBQLN1 | Q9UMX0 | 19188.77247 |
| 3742 | C1orf122 | Q6ZSJ8 | 19185.21871 |
| 3743 | DDX20 | Q9UHI6 | 19154.83494 |
| 3744 | RINT1 | Q6NUQ1 | 19146.27867 |
| 3745 | LRCH3 | Q96II8 | 19133.28814 |
| 3746 | MIA3 | Q5JRA6 | 19131.55456 |
| 3747 | SPTBN2 | O15020 | 19121.08513 |
| 3748 | MTPAP | Q9NVV4 | 19116.29638 |
| 3749 | QTRT2 | Q9H974 | 19079.83676 |
| 3750 | TMEM192 | Q8IY95 | 19058.81846 |
| 3751 | DENND4C | Q5VZ89 | 19058.38124 |
| 3752 | WDR6 | Q9NNW5 | 19054.76387 |
| 3753 | CLDN11 | O75508 | 19044.70733 |
| 3754 | SPTLC2 | O15270 | 19032.83438 |
| 3755 | GNL2 | Q13823 | 19019.57062 |
| 3756 | MPZL2 | O60487 | 18985.77356 |
| 3757 | MIPEP | Q99797 | 18977.86304 |
| 3758 | NCAPG | Q9BPX3 | 18954.38323 |
| 3759 | DCD | P81605 | 18942.828 |
| 3760 | AGL | P35573 | 18924.0355 |
| 3761 | MICAL2 | O94851 | 18904.69803 |
| 3762 | BUD23 | O43709 | 18902.796 |
| 3763 | PAPOLA | P51003 | 18890.53994 |
| 3764 | HECTD1 | Q9ULT8 | 18868.31649 |
| 3765 | SEMA6B | Q9H3T3 | 18858.72709 |
| 3766 | SNRPB2 | P08579 | 18855.4853 |
| 3767 | CDC16 | Q13042 | 18829.48411 |
| 3768 | EVA1B | Q9NVM1 | 18829.2165 |
| 3769 | KLC2 | Q9H0B6 | 18802.85957 |
| 3770 | ATPAF2 | Q8N5M1 | 18800.6526 |
| 3771 | USP4 | Q13107 | 18781.22158 |
| 3772 | WDR3 | Q9UNX4 | 18748.46987 |
| 3773 | LPCAT4 | Q643R3 | 18746.5507 |
| 3774 | SELENOM | Q8WWX9 | 18736.06114 |
| 3775 | RBM15 | Q96T37 | 18722.63634 |
| 3776 | FAM32A | Q9Y421 | 18714.916 |
| 3777 | C2orf76 | Q3KRA6 | 18696.85238 |
| 3778 | NADK2 | Q4G0N4 | 18680.30004 |
| 3779 | UEVLD | Q8IX04 | 18679.32166 |
| 3780 | GULP1 | Q9UBP9 | 18671.38 |
| 3781 | URI1 | O94763 | 18651.92438 |
| 3782 | AKAP8L | Q9ULX6 | 18647.21996 |
| 3783 | GTF2E1 | P29083 | 18643.03851 |
| 3784 | FBXL8 | Q96CD0 | 18619.3404 |
| 3785 | NIF3L1 | Q9GZT8 | 18618.51074 |
| 3786 | PDRG1 | Q9NUG6 | 18605.23063 |
| 3787 | PAIP2 | Q9BPZ3 | 18596.874 |
| 3788 | PPFIA1 | Q13136 | 18586.31457 |
| 3789 | UBIAD1 | Q9Y5Z9 | 18573.731 |
| 3790 | CMC2 | Q9NRP2 | 18562.65 |
| 3791 | POLR2I | P36954 | 18561.10825 |
| 3792 | ITSN2 | Q9NZM3 | 18559.9549 |
| 3793 | THOC5 | Q13769 | 18536.70517 |
| 3794 | SPC24 | Q8NBT2 | 18536.402 |
| 3795 | ENO3 | P13929 | 18521.67647 |
| 3796 | WDR70 | Q9NW82 | 18519.64911 |
| 3797 | AKAP8 | O43823 | 18518.72048 |
| 3798 | FBXO7 | Q9Y3I1 | 18517.72019 |
| 3799 | SMARCC2 | Q8TAQ2 | 18515.28969 |
| 3800 | SUPT6H | Q7KZ85 | 18507.64616 |
| 3801 | GAS6 | Q14393 | 18467.31203 |
| 3802 | ALG1 | Q9BT22 | 18453.42638 |
| 3803 | CAMLG | P49069 | 18452.91213 |
| 3804 | C1orf50 | Q9BV19 | 18447.96451 |
| 3805 | PLEKHF2 | Q9H8W4 | 18441.84786 |
| 3806 | TBC1D17 | Q9HA65 | 18438.00354 |
| 3807 | COG2 | Q14746 | 18430.38105 |
| 3808 | LYPD1 | Q8N2G4 | 18422.405 |
| 3809 | WDR45B | Q5MNZ6 | 18406.68162 |
| 3810 | C4A | P0C0L4 | 18402.04559 |
| 3811 | ARL15 | Q9NXU5 | 18394.9354 |
| 3812 | NOP9 | Q86U38 | 18379.6309 |
| 3813 | C19orf25 | Q9UFG5 | 18353.60357 |
| 3814 | KCMF1 | Q9P0J7 | 18351.24213 |
| 3815 | RAVER1 | Q8IY67 | 18308.85009 |
| 3816 | PEDS1 | A5PLL7 | 18299.57908 |
| 3817 | KIF3A | Q9Y496 | 18295.88567 |
| 3818 | HARS2 | P49590 | 18261.46941 |
| 3819 | DOCK4 | Q8N1I0 | 18236.69106 |
| 3820 | HS2ST1 | Q7LGA3 | 18215.9785 |
| 3821 | DHX38 | Q92620 | 18213.86414 |
| 3822 | CIAO2A | Q9H5X1 | 18213.58788 |
| 3823 | ABCB10 | Q9NRK6 | 18198.21693 |
| 3824 | PRKACA | P17612 | 18178.80405 |
| 3825 | PEX5 | P50542 | 18162.1293 |
| 3826 | PIK3R1 | P27986 | 18160.86557 |
| 3827 | MCC | P23508 | 18160.69105 |
| 3828 | MYL6B | P14649 | 18142.20477 |
| 3829 | RIPK2 | O43353 | 18137.96293 |
| 3830 | RELL1 | Q8IUW5 | 18122.92138 |
| 3831 | ZBTB8OS | Q8IWT0 | 18096.584 |
| 3832 | NUP37 | Q8NFH4 | 18085.39681 |
| 3833 | PABPC3 | Q9H361 | 18068.56 |
| 3834 | FOXRED1 | Q96CU9 | 18063.28885 |
| 3835 | AKAP13 | Q12802 | 18056.12647 |
| 3836 | DCTN5 | Q9BTE1 | 18046.6815 |
| 3837 | CXorf38 | Q8TB03 | 18036.2746 |
| 3838 | WDR13 | Q9H1Z4 | 18030.64148 |
| 3839 | MTX1 | Q13505 | 18026.82344 |
| 3840 | MDP1 | Q86V88 | 18022.67227 |
| 3841 | TEX264 | Q9Y6I9 | 18017.1464 |
| 3842 | IBA57 | Q5T440 | 18010.62729 |
| 3843 | PUDP | Q08623 | 18000.0211 |
| 3844 | DCHS1 | Q96JQ0 | 17981.21943 |
| 3845 | MED20 | Q9H944 | 17976.56667 |
| 3846 | YIPF2 | Q9BWQ6 | 17939.74875 |
| 3847 | POLR1A | O95602 | 17939.35531 |
| 3848 | CDC23 | Q9UJX2 | 17936.17194 |
| 3849 | B3GLCT | Q6Y288 | 17911.70495 |
| 3850 | TFDP1 | Q14186 | 17900.87321 |
| 3851 | PLEKHA1 | Q9HB21 | 17898.08473 |
| 3852 | BAK1 | Q16611 | 17874.7196 |
| 3853 | TLE5 | Q08117 | 17872.07375 |
| 3854 | PALM | O75781 | 17861.41291 |
| 3855 | MTR | Q99707 | 17813.28988 |
| 3856 | ARID1A | O14497 | 17799.07712 |
| 3857 | ATP2B1 | P20020 | 17790.51635 |
| 3858 | NT5C3A | Q9H0P0 | 17789.61776 |
| 3859 | CDKN2AIP | Q9NXV6 | 17778.189 |
| 3860 | NKRF | O15226 | 17774.81826 |
| 3861 | TMEM68 | Q96MH6 | 17750.868 |
| 3862 | MSI2 | Q96DH6 | 17749.005 |
| 3863 | UTP4 | Q969X6 | 17732.36727 |
| 3864 | FXR2 | P51116 | 17718.83889 |
| 3865 | METTL16 | Q86W50 | 17711.3635 |
| 3866 | PRPSAP2 | O60256 | 17697.70657 |
| 3867 | FAM120B | Q96EK7 | 17661.97366 |
| 3868 | SH3BGRL2 | Q9UJC5 | 17643.793 |
| 3869 | PLCH1 | Q4KWH8 | 17620.83675 |
| 3870 | MPDZ | O75970 | 17611.48916 |
| 3871 | IRF3 | Q14653 | 17589.7644 |
| 3872 | SMARCD2 | Q92925 | 17582.01004 |
| 3873 | TRIP4 | Q15650 | 17566.86731 |
| 3874 | YTHDC2 | Q9H6S0 | 17560.67046 |
| 3875 | VPS39 | Q96JC1 | 17553.66566 |
| 3876 | EXOG | Q9Y2C4 | 17546.16582 |
| 3877 | PCK2 | Q16822 | 17534.69174 |
| 3878 | COL5A1 | P20908 | 17518.77657 |
| 3879 | CAV2 | P51636 | 17518.40011 |
| 3880 | GTF3C3 | Q9Y5Q9 | 17517.56558 |
| 3881 | ARHGAP26 | Q9UNA1 | 17515.6567 |
| 3882 | PKP2 | Q99959 | 17512.22049 |
| 3883 | GABPA | Q06546 | 17506.9507 |
| 3884 | MAIP1 | Q8WWC4 | 17497.16271 |
| 3885 | ZC3HC1 | Q86WB0 | 17485.81567 |
| 3886 | UBA7 | P41226 | 17483.28127 |
| 3887 | SIAE | Q9HAT2 | 17467.73767 |
| 3888 | DOCK7 | Q96N67 | 17458.00798 |
| 3889 | DUS2 | Q9NX74 | 17426.05564 |
| 3890 | GRB10 | Q13322 | 17416.45269 |
| 3891 | NUDT19 | A8MXV4 | 17394.86849 |
| 3892 | DHX8 | Q14562 | 17393.4228 |
| 3893 | CRYZL1 | O95825 | 17388.5678 |
| 3894 | FCHO2 | Q0JRZ9 | 17387.39341 |
| 3895 | UACA | Q9BZF9 | 17361.9217 |
| 3896 | RAB30 | Q15771 | 17332.5164 |
| 3897 | UBR7 | Q8N806 | 17322.67304 |
| 3898 | RIOK2 | Q9BVS4 | 17321.78937 |
| 3899 | MSTO1 | Q9BUK6 | 17317.11229 |
| 3900 | RBPMS | Q93062 | 17307.8177 |
| 3901 | ACY1 | Q03154 | 17304.85135 |
| 3902 | SEC23B | Q15437 | 17283.99497 |
| 3903 | GNE | Q9Y223 | 17274.87919 |
| 3904 | TAOK3 | Q9H2K8 | 17231.34351 |
| 3905 | PCID2 | Q5JVF3 | 17222.80041 |
| 3906 | RASA3 | Q14644 | 17210.65527 |
| 3907 | VRK2 | Q86Y07 | 17201.51515 |
| 3908 | DPY30 | Q9C005 | 17181.818 |
| 3909 | INTS4 | Q96HW7 | 17172.5376 |
| 3910 | MAP2K4 | P45985 | 17156.38209 |
| 3911 | MLST8 | Q9BVC4 | 17155.05167 |
| 3912 | CCDC137 | Q6PK04 | 17124.27654 |
| 3913 | ATAD1 | Q8NBU5 | 17113.67424 |
| 3914 | KANK3 | Q6NY19 | 17105.66082 |
| 3915 | NCAPD2 | Q15021 | 17052.55667 |
| 3916 | EFL1 | Q7Z2Z2 | 17050.59511 |
| 3917 | TINAGL1 | Q9GZM7 | 17030.79483 |
| 3918 | SLC9A1 | P19634 | 17016.39046 |
| 3919 | GTPBP10 | A4D1E9 | 17011.4598 |
| 3920 | NDUFB11 | Q9NX14 | 16982.67222 |
| 3921 | SDF2 | Q99470 | 16982.42556 |
| 3922 | PIGK | Q92643 | 16981.83333 |
| 3923 | KLHL42 | Q9P2K6 | 16966.56013 |
| 3924 | CNEP1R1 | Q8N9A8 | 16949.35671 |
| 3925 | RPL36A | P83881 | 16941.355 |
| 3926 | DHX36 | Q9H2U1 | 16927.66672 |
| 3927 | DHRS1 | Q96LJ7 | 16907.11788 |
| 3928 | MFN1 | Q8IWA4 | 16892.65633 |
| 3929 | WBP4 | O75554 | 16889.74386 |
| 3930 | UTP6 | Q9NYH9 | 16887.77811 |
| 3931 | NDUFB6 | O95139 | 16873.95913 |
| 3932 | CUEDC2 | Q9H467 | 16862.221 |
| 3933 | TSC22D1 | Q15714 | 16854.316 |
| 3934 | SLC4A1AP | Q9BWU0 | 16854.19849 |
| 3935 | NELFB | Q8WX92 | 16850.44393 |
| 3936 | BRD3 | Q15059 | 16848.98339 |
| 3937 | TMEM199 | Q8N511 | 16842.62462 |
| 3938 | GNA12 | Q03113 | 16841.311 |
| 3939 | LANCL2 | Q9NS86 | 16830.46012 |
| 3940 | KLC4 | Q9NSK0 | 16813.57689 |
| 3941 | DBR1 | Q9UK59 | 16812.37671 |
| 3942 | NAXD | Q8IW45 | 16810.71356 |
| 3943 | MRPL10 | Q7Z7H8 | 16763.58227 |
| 3944 | PPP2R1B | P30154 | 16753.3325 |
| 3945 | CDS2 | O95674 | 16732.33171 |
| 3946 | LIN7C | Q9NUP9 | 16729.53055 |
| 3947 | NLE1 | Q9NVX2 | 16728.82728 |
| 3948 | MGAT4B | Q9UQ53 | 16724.5307 |
| 3949 | STARD13 | Q9Y3M8 | 16704.94667 |
| 3950 | PPP2R5A | Q15172 | 16685.8446 |
| 3951 | STX18 | Q9P2W9 | 16680.41995 |
| 3952 | ISY1 | Q9ULR0 | 16618.32686 |
| 3953 | CD109 | Q6YHK3 | 16580.84563 |
| 3954 | SIGMAR1 | Q99720 | 16576.09625 |
| 3955 | LDHAL6B | Q9BYZ2 | 16573.1982 |
| 3956 | AAR2 | Q9Y312 | 16564.79863 |
| 3957 | RHBDD1 | Q8TEB9 | 16547.18622 |
| 3958 | FN3K | Q9H479 | 16545.13486 |
| 3959 | GTF2H3 | Q13889 | 16535.50407 |
| 3960 | FBXO28 | Q9NVF7 | 16524.31062 |
| 3961 | CRELD2 | Q6UXH1 | 16512.26253 |
| 3962 | PLXNB2 | O15031 | 16467.95823 |
| 3963 | ANKLE2 | Q86XL3 | 16461.9274 |
| 3964 | WDR33 | Q9C0J8 | 16460.44871 |
| 3965 | TAF15 | Q92804 | 16431.24905 |
| 3966 | DLGAP4 | Q9Y2H0 | 16428.15494 |
| 3967 | TRIM16 | O95361 | 16427.39027 |
| 3968 | TFCP2 | Q12800 | 16426.03489 |
| 3969 | PHYKPL | Q8IUZ5 | 16417.09735 |
| 3970 | USP25 | Q9UHP3 | 16417.02148 |
| 3971 | SLC27A1 | Q6PCB7 | 16393.648 |
| 3972 | KDR | P35968 | 16392.10805 |
| 3973 | DNMT1 | P26358 | 16382.33394 |
| 3974 | NOLC1 | Q14978 | 16380.73795 |
| 3975 | ATP8B1 | O43520 | 16377.97504 |
| 3976 | ITM2B | Q9Y287 | 16357.06 |
| 3977 | STX17 | P56962 | 16337.11775 |
| 3978 | CSNK1D | P48730 | 16312.92559 |
| 3979 | NRP2 | O60462 | 16307.302 |
| 3980 | MAPK12 | P53778 | 16273.49158 |
| 3981 | SUGP1 | Q8IWZ8 | 16258.65484 |
| 3982 | MTARC2 | Q969Z3 | 16242.98425 |
| 3983 | UXT | Q9UBK9 | 16231.76625 |
| 3984 | UBE2B | P63146 | 16222.85567 |
| 3985 | SFT2D3 | Q587I9 | 16216.68267 |
| 3986 | UBE4B | O95155 | 16210.80358 |
| 3987 | ASH2L | Q9UBL3 | 16190.29772 |
| 3988 | GCC2 | Q8IWJ2 | 16187.9684 |
| 3989 | SYNE3 | Q6ZMZ3 | 16178.9109 |
| 3990 | DDX51 | Q8N8A6 | 16173.50029 |
| 3991 | JAK1 | P23458 | 16170.56242 |
| 3992 | KRT85 | P78386 | 16157.61055 |
| 3993 | ZC3H11A | O75152 | 16127.16529 |
| 3994 | VIPAS39 | Q9H9C1 | 16123.33367 |
| 3995 | COQ9 | O75208 | 16119.57925 |
| 3996 | PPIG | Q13427 | 16117.45487 |
| 3997 | ITIH4 | Q14624 | 16116.87009 |
| 3998 | NPC1 | O15118 | 16113.05371 |
| 3999 | UXS1 | Q8NBZ7 | 16109.47041 |
| 4000 | TTC38 | Q5R3I4 | 16107.20364 |
| 4001 | SELENBP1 | Q13228 | 16104.08871 |
| 4002 | KATNAL1 | Q9BW62 | 16091.98418 |
| 4003 | EIF1AD | Q8N9N8 | 16085.62096 |
| 4004 | ANGPT2 | O15123 | 16070.37272 |
| 4005 | MNAT1 | P51948 | 16066.60693 |
| 4006 | IFT20 | Q8IY31 | 16050.39814 |
| 4007 | INTS7 | Q9NVH2 | 16030.5064 |
| 4008 | PPP4R3A | Q6IN85 | 16023.88121 |
| 4009 | AKT3 | Q9Y243 | 16016.45996 |
| 4010 | TDG | Q13569 | 16001.7936 |
| 4011 | SCRN3 | Q0VDG4 | 15999.99363 |
| 4012 | ARL10 | Q8N8L6 | 15976.89267 |
| 4013 | RBBP5 | Q15291 | 15976.53323 |
| 4014 | PRKCH | P24723 | 15975.31769 |
| 4015 | SMIM20 | Q8N5G0 | 15974.213 |
| 4016 | RNF31 | Q96EP0 | 15973.46319 |
| 4017 | GIT2 | Q14161 | 15966.06175 |
| 4018 | TRIM3 | O75382 | 15960.83197 |
| 4019 | LRRC14 | Q15048 | 15937.60259 |
| 4020 | GRN | P28799 | 15897.58375 |
| 4021 | MTFP1 | Q9UDX5 | 15851.37833 |
| 4022 | C2CD5 | Q86YS7 | 15837.17388 |
| 4023 | NOL7 | Q9UMY1 | 15828.63913 |
| 4024 | L2HGDH | Q9H9P8 | 15826.46868 |
| 4025 | FAM168B | A1KXE4 | 15820.34367 |
| 4026 | PPIP5K2 | O43314 | 15808.0321 |
| 4027 | PPP1R21 | Q6ZMI0 | 15799.26089 |
| 4028 | TOMM7 | Q9P0U1 | 15791.51467 |
| 4029 | FRG1 | Q14331 | 15782.799 |
| 4030 | LMCD1 | Q9NZU5 | 15782.6728 |
| 4031 | SNX7 | Q9UNH6 | 15778.8319 |
| 4032 | PRXL2B | Q8TBF2 | 15772.55242 |
| 4033 | SEPTIN5 | Q99719 | 15770.31976 |
| 4034 | FDFT1 | P37268 | 15769.0415 |
| 4035 | NR2C2AP | Q86WQ0 | 15748.03888 |
| 4036 | POLR3B | Q9NW08 | 15747.94198 |
| 4037 | MICALL1 | Q8N3F8 | 15742.11631 |
| 4038 | DIS3L2 | Q8IYB7 | 15741.96936 |
| 4039 | DDX52 | Q9Y2R4 | 15710.93971 |
| 4040 | ABCD4 | O14678 | 15710.5933 |
| 4041 | COG8 | Q96MW5 | 15636.39391 |
| 4042 | MTM1 | Q13496 | 15606.59925 |
| 4043 | RALGAPA2 | Q2PPJ7 | 15601.13847 |
| 4044 | UPF2 | Q9HAU5 | 15566.40897 |
| 4045 | STX3 | Q13277 | 15561.67017 |
| 4046 | AAMP | Q13685 | 15554.74688 |
| 4047 | WDR75 | Q8IWA0 | 15497.02095 |
| 4048 | KLHL5 | Q96PQ7 | 15459.40263 |
| 4049 | NEDD4 | P46934 | 15453.00235 |
| 4050 | NDE1 | Q9NXR1 | 15411.86608 |
| 4051 | EPB41L5 | Q9HCM4 | 15392.46276 |
| 4052 | RASSF2 | P50749 | 15331.00828 |
| 4053 | ABCD1 | P33897 | 15327.19866 |
| 4054 | TBCEL | Q5QJ74 | 15321.83232 |
| 4055 | CPSF4 | O95639 | 15321.27156 |
| 4056 | UQCC1 | Q9NVA1 | 15311.87467 |
| 4057 | SDAD1 | Q9NVU7 | 15291.44281 |
| 4058 | PHLDA3 | Q9Y5J5 | 15289.16222 |
| 4059 | EXOC6 | Q8TAG9 | 15285.313 |
| 4060 | VPS50 | Q96JG6 | 15282.1469 |
| 4061 | BAZ1B | Q9UIG0 | 15270.98651 |
| 4062 | FYCO1 | Q9BQS8 | 15258.17059 |
| 4063 | IFI35 | P80217 | 15245.87179 |
| 4064 | OTUD4 | Q01804 | 15227.46963 |
| 4065 | MED4 | Q9NPJ6 | 15204.855 |
| 4066 | GRK6 | P43250 | 15204.25096 |
| 4067 | ABI2 | Q9NYB9 | 15192.73061 |
| 4068 | PUS1 | Q9Y606 | 15179.2461 |
| 4069 | E2F4 | Q16254 | 15176.95132 |
| 4070 | RBM27 | Q9P2N5 | 15171.2992 |
| 4071 | PPP3CB | P16298 | 15166.82933 |
| 4072 | KIAA1143 | Q96AT1 | 15165.71411 |
| 4073 | INTS10 | Q9NVR2 | 15157.6699 |
| 4074 | RARS2 | Q5T160 | 15130.48197 |
| 4075 | PYCR3 | Q53H96 | 15120.93669 |
| 4076 | AP4M1 | O00189 | 15110.66692 |
| 4077 | LACTB2 | Q53H82 | 15104.488 |
| 4078 | CLP1 | Q92989 | 15096.76222 |
| 4079 | MCAT | Q8IVS2 | 15095.07182 |
| 4080 | SH2D3C | Q8N5H7 | 15075.61683 |
| 4081 | ID3 | Q02535 | 15075.43375 |
| 4082 | INPP5F | Q9Y2H2 | 15064.98985 |
| 4083 | RWDD1 | Q9H446 | 15046.3775 |
| 4084 | SCAMP4 | Q969E2 | 15043.8116 |
| 4085 | ERCC2 | P18074 | 15024.3468 |
| 4086 | CLASP2 | O75122 | 15019.93613 |
| 4087 | RBM4 | Q9BWF3 | 15000.13105 |
| 4088 | PLPP3 | O14495 | 14992.24386 |
| 4089 | TUT4 | Q5TAX3 | 14981.78964 |
| 4090 | TIMM21 | Q9BVV7 | 14976.23331 |
| 4091 | MORF4L2 | Q15014 | 14966.57918 |
| 4092 | TRIM5 | Q9C035 | 14948.01338 |
| 4093 | WDR48 | Q8TAF3 | 14908.63668 |
| 4094 | HIF1AN | Q9NWT6 | 14900.70106 |
| 4095 | BCKDK | O14874 | 14889.3093 |
| 4096 | MTOR | P42345 | 14875.10263 |
| 4097 | ABCB7 | O75027 | 14844.5745 |
| 4098 | RANBP10 | Q6VN20 | 14843.22205 |
| 4099 | PPOX | P50336 | 14826.37164 |
| 4100 | ATP5IF1 | Q9UII2 | 14821.61314 |
| 4101 | HSP90AB2P | Q58FF8 | 14803.71318 |
| 4102 | PPP1R9B | Q96SB3 | 14799.20219 |
| 4103 | PCDHGB5 | Q9Y5G0 | 14790.37906 |
| 4104 | UBP1 | Q9NZI7 | 14779.00821 |
| 4105 | ARFGEF1 | Q9Y6D6 | 14740.34932 |
| 4106 | PRPF38B | Q5VTL8 | 14729.07571 |
| 4107 | EXOSC10 | Q01780 | 14714.58815 |
| 4108 | TSTD1 | Q8NFU3 | 14703.78333 |
| 4109 | FUNDC1 | Q8IVP5 | 14699.85357 |
| 4110 | UVRAG | Q9P2Y5 | 14699.84658 |
| 4111 | SLC44A2 | Q8IWA5 | 14669.47103 |
| 4112 | PPP4R2 | Q9NY27 | 14635.28619 |
| 4113 | GCLC | P48506 | 14627.4201 |
| 4114 | HDHD2 | Q9H0R4 | 14612.75977 |
| 4115 | RDH13 | Q8NBN7 | 14612.73484 |
| 4116 | TMA16 | Q96EY4 | 14593.00389 |
| 4117 | PIP4K2A | P48426 | 14590.51095 |
| 4118 | WDR4 | P57081 | 14585.9315 |
| 4119 | SFSWAP | Q12872 | 14569.88451 |
| 4120 | CLDND1 | Q9NY35 | 14563.9648 |
| 4121 | CRLF3 | Q8IUI8 | 14534.39365 |
| 4122 | LMO7 | Q8WWI1 | 14518.45867 |
| 4123 | ARHGEF15 | O94989 | 14514.79085 |
| 4124 | AGRN | O00468 | 14510.6004 |
| 4125 | PPP6R1 | Q9UPN7 | 14504.34349 |
| 4126 | PATL1 | Q86TB9 | 14500.86717 |
| 4127 | MPHOSPH6 | Q99547 | 14490.4326 |
| 4128 | BIRC2 | Q13490 | 14486.291 |
| 4129 | SMAD2 | Q15796 | 14481.90056 |
| 4130 | QPCTL | Q9NXS2 | 14466.00347 |
| 4131 | CERT1 | Q9Y5P4 | 14452.09183 |
| 4132 | SUPV3L1 | Q8IYB8 | 14452.02556 |
| 4133 | ASF1A | Q9Y294 | 14426.59963 |
| 4134 | PPL | O60437 | 14425.36219 |
| 4135 | C2orf49 | Q9BVC5 | 14418.6246 |
| 4136 | SLC33A1 | O00400 | 14415.8585 |
| 4137 | RBM45 | Q8IUH3 | 14406.62864 |
| 4138 | GEMIN5 | Q8TEQ6 | 14389.24409 |
| 4139 | PSME4 | Q14997 | 14379.55276 |
| 4140 | AGPAT3 | Q9NRZ7 | 14367.14732 |
| 4141 | PHLDB2 | Q86SQ0 | 14364.18672 |
| 4142 | PDZD11 | Q5EBL8 | 14349.67571 |
| 4143 | OSTM1 | Q86WC4 | 14342.98291 |
| 4144 | SERPINF1 | P36955 | 14339.99586 |
| 4145 | KBTBD2 | Q8IY47 | 14331.09481 |
| 4146 | RANBP6 | O60518 | 14330.2386 |
| 4147 | MAEA | Q7L5Y9 | 14295.37252 |
| 4148 | SPDL1 | Q96EA4 | 14294.57378 |
| 4149 | HSPA4L | O95757 | 14285.30542 |
| 4150 | LTBP2 | Q14767 | 14279.57489 |
| 4151 | ATP11C | Q8NB49 | 14276.20644 |
| 4152 | EIF4E2 | O60573 | 14272.87342 |
| 4153 | WIPF1 | O43516 | 14271.50364 |
| 4154 | KCT2 | Q8NC54 | 14259.12667 |
| 4155 | MED15 | Q96RN5 | 14257.6293 |
| 4156 | MAX | P61244 | 14251.7039 |
| 4157 | PI4KA | P42356 | 14241.65532 |
| 4158 | DNTTIP1 | Q9H147 | 14237.05835 |
| 4159 | PIEZO1 | Q92508 | 14235.80314 |
| 4160 | HIKESHI | Q53FT3 | 14232.95833 |
| 4161 | NTF3 | P20783 | 14225.775 |
| 4162 | KCTD21 | Q4G0X4 | 14219.76067 |
| 4163 | KIRREL1 | Q96J84 | 14204.15273 |
| 4164 | XRCC1 | P18887 | 14194.58661 |
| 4165 | CERS5 | Q8N5B7 | 14176.64113 |
| 4166 | RTN3 | O95197 | 14156.81065 |
| 4167 | TRIM65 | Q6PJ69 | 14144.57635 |
| 4168 | AGPAT5 | Q9NUQ2 | 14132.03279 |
| 4169 | CASP4 | P49662 | 14131.64943 |
| 4170 | BCL3 | P20749 | 14104.91013 |
| 4171 | KCTD10 | Q9H3F6 | 14101.13195 |
| 4172 | SPATA5L1 | Q9BVQ7 | 14097.95679 |
| 4173 | CCDC51 | Q96ER9 | 14091.11859 |
| 4174 | STK3 | Q13188 | 14076.32652 |
| 4175 | SASH1 | O94885 | 14060.90715 |
| 4176 | PALS1 | Q8N3R9 | 14050.33805 |
| 4177 | PEAK1 | Q9H792 | 14045.45054 |
| 4178 | IFITM3 | Q01628 | 14036.6236 |
| 4179 | ABI3BP | Q7Z7G0 | 14031.37245 |
| 4180 | ACYP2 | P14621 | 14029.77189 |
| 4181 | CD274 | Q9NZQ7 | 14028.75456 |
| 4182 | UGGT2 | Q9NYU1 | 14012.78439 |
| 4183 | PDCD2L | Q9BRP1 | 13984.08771 |
| 4184 | POLR1B | Q9H9Y6 | 13982.71742 |
| 4185 | KIAA1671 | Q9BY89 | 13977.60999 |
| 4186 | WDR11 | Q9BZH6 | 13975.21254 |
| 4187 | SENP3 | Q9H4L4 | 13968.86136 |
| 4188 | UBE2S | Q16763 | 13955.79409 |
| 4189 | ETNPPL | Q8TBG4 | 13952.51423 |
| 4190 | CNOT4 | O95628 | 13943.35629 |
| 4191 | EPM2AIP1 | Q7L775 | 13922.65826 |
| 4192 | H1-3 | P16402 | 13912.39333 |
| 4193 | LRRC8D | Q7L1W4 | 13897.71383 |
| 4194 | ZCCHC17 | Q9NP64 | 13892.72 |
| 4195 | STX10 | O60499 | 13886.90138 |
| 4196 | C2CD2L | O14523 | 13879.64465 |
| 4197 | DNAJB14 | Q8TBM8 | 13878.68222 |
| 4198 | OSBPL9 | Q96SU4 | 13870.23768 |
| 4199 | TIMP3 | P35625 | 13830.42091 |
| 4200 | KIF14 | Q15058 | 13819.6683 |
| 4201 | C8orf33 | Q9H7E9 | 13805.98318 |
| 4202 | TLE3 | Q04726 | 13796.05538 |
| 4203 | HDAC1 | Q13547 | 13784.7872 |
| 4204 | TDP2 | O95551 | 13784.43129 |
| 4205 | UPP1 | Q16831 | 13767.69163 |
| 4206 | BRCC3 | P46736 | 13759.61621 |
| 4207 | SMCHD1 | A6NHR9 | 13755.55422 |
| 4208 | NDC1 | Q9BTX1 | 13753.39881 |
| 4209 | PROSER2 | Q86WR7 | 13743.07591 |
| 4210 | SMAD4 | Q13485 | 13738.77826 |
| 4211 | B3GALT6 | Q96L58 | 13737.30271 |
| 4212 | AATF | Q9NY61 | 13722.17814 |
| 4213 | BNIP2 | Q12982 | 13712.06079 |
| 4214 | OTUD7B | Q6GQQ9 | 13711.94006 |
| 4215 | KCNG4 | Q8TDN1 | 13710.203 |
| 4216 | BCL7C | Q8WUZ0 | 13708.1765 |
| 4217 | CHUK | O15111 | 13693.53051 |
| 4218 | HMBS | P08397 | 13685.60747 |
| 4219 | RUSF1 | Q96GQ5 | 13679.16784 |
| 4220 | HINT3 | Q9NQE9 | 13675.63789 |
| 4221 | COA6 | Q5JTJ3 | 13673.89343 |
| 4222 | ECD | O95905 | 13667.88682 |
| 4223 | SCLY | Q96I15 | 13647.59052 |
| 4224 | TCAF1 | Q9Y4C2 | 13626.50754 |
| 4225 | MCCC1 | Q96RQ3 | 13625.8273 |
| 4226 | POMP | Q9Y244 | 13612.26667 |
| 4227 | LNPK | Q9C0E8 | 13607.27838 |
| 4228 | XRCC4 | Q13426 | 13598.38561 |
| 4229 | DNAJC5 | Q9H3Z4 | 13588.84513 |
| 4230 | CARD11 | Q9BXL7 | 13578.46372 |
| 4231 | GATAD2B | Q8WXI9 | 13566.21967 |
| 4232 | NDUFAF7 | Q7L592 | 13565.59573 |
| 4233 | CEBPZ | Q03701 | 13541.26121 |
| 4234 | TTC13 | Q8NBP0 | 13522.71927 |
| 4235 | MTMR9 | Q96QG7 | 13513.72396 |
| 4236 | POLR2M | Q6EEV4 | 13507.70729 |
| 4237 | YTHDC1 | Q96MU7 | 13493.82356 |
| 4238 | BOK | Q9UMX3 | 13490.6095 |
| 4239 | ELP2 | Q6IA86 | 13475.39134 |
| 4240 | NACA4P | Q9BZK3 | 13463.03514 |
| 4241 | ELF1 | P32519 | 13449.99167 |
| 4242 | HAUS4 | Q9H6D7 | 13440.5682 |
| 4243 | PPP1R10 | Q96QC0 | 13439.44816 |
| 4244 | MLLT11 | Q13015 | 13434.2248 |
| 4245 | PREX1 | Q8TCU6 | 13430.69276 |
| 4246 | TCP11L1 | Q9NUJ3 | 13414.50133 |
| 4247 | DBNDD1 | Q9H9R9 | 13390.564 |
| 4248 | TFPI | P10646 | 13375.03285 |
| 4249 | NT5C3B | Q969T7 | 13340.64778 |
| 4250 | SRRM1 | Q8IYB3 | 13292.60899 |
| 4251 | TRMT1 | Q9NXH9 | 13286.06979 |
| 4252 | SLFN5 | Q08AF3 | 13270.73107 |
| 4253 | RBM12B | Q8IXT5 | 13266.46498 |
| 4254 | THOC2 | Q8NI27 | 13257.14154 |
| 4255 | ZNF131 | P52739 | 13256.81293 |
| 4256 | UBE2C | O00762 | 13238.73109 |
| 4257 | TMEM14C | Q9P0S9 | 13212.8028 |
| 4258 | AASS | Q9UDR5 | 13208.64654 |
| 4259 | SESTD1 | Q86VW0 | 13203.10206 |
| 4260 | DKK3 | Q9UBP4 | 13200.44676 |
| 4261 | SNX33 | Q8WV41 | 13197.32858 |
| 4262 | RB1 | P06400 | 13195.26108 |
| 4263 | DHX29 | Q7Z478 | 13191.12929 |
| 4264 | LHPP | Q9H008 | 13191.05018 |
| 4265 | HACL1 | Q9UJ83 | 13163.88139 |
| 4266 | PRPF38A | Q8NAV1 | 13133.33 |
| 4267 | METTL9 | Q9H1A3 | 13101.58559 |
| 4268 | USP24 | Q9UPU5 | 13095.4964 |
| 4269 | GGPS1 | O95749 | 13089.70994 |
| 4270 | DNLZ | Q5SXM8 | 13069.58417 |
| 4271 | NLGN1 | Q8N2Q7 | 13062.7931 |
| 4272 | RIN2 | Q8WYP3 | 13058.3973 |
| 4273 | VEPH1 | Q14D04 | 13055.92598 |
| 4274 | SLC7A1 | P30825 | 13042.58015 |
| 4275 | FAM118B | Q9BPY3 | 13030.235 |
| 4276 | RBFOX2 | O43251 | 13016.32077 |
| 4277 | TUT7 | Q5VYS8 | 13011.65973 |
| 4278 | TSEN34 | Q9BSV6 | 13005.37939 |
| 4279 | HAUS8 | Q9BT25 | 12996.36312 |
| 4280 | HMGN5 | P82970 | 12993.51942 |
| 4281 | AGAP3 | Q96P47 | 12988.79078 |
| 4282 | TRIM32 | Q13049 | 12985.46675 |
| 4283 | GIMAP7 | Q8NHV1 | 12971.37964 |
| 4284 | ASAP2 | O43150 | 12969.24943 |
| 4285 | ABCC11 | Q96J66 | 12968.35217 |
| 4286 | TTC7B | Q86TV6 | 12964.58215 |
| 4287 | NCAPH | Q15003 | 12951.26425 |
| 4288 | ERGIC2 | Q96RQ1 | 12950.90611 |
| 4289 | HSD17B11 | Q8NBQ5 | 12932.93789 |
| 4290 | PLSCR1 | O15162 | 12927.25267 |
| 4291 | MAN2B2 | Q9Y2E5 | 12915.99618 |
| 4292 | KIF20B | Q96Q89 | 12906.71142 |
| 4293 | MMP2 | P08253 | 12868.81347 |
| 4294 | MAP2K6 | P52564 | 12852.13868 |
| 4295 | TMPPE | Q6ZT21 | 12851.80271 |
| 4296 | GUSB | P08236 | 12851.78788 |
| 4297 | C1QTNF6 | Q9BXI9 | 12841.84143 |
| 4298 | RRP8 | O43159 | 12832.1942 |
| 4299 | AIFM2 | Q9BRQ8 | 12830.75711 |
| 4300 | RRP7A | Q9Y3A4 | 12829.81508 |
| 4301 | ITPRIP | Q8IWB1 | 12810.35497 |
| 4302 | SRXN1 | Q9BYN0 | 12790.06911 |
| 4303 | ITIH3 | Q06033 | 12776.91956 |
| 4304 | EDEM3 | Q9BZQ6 | 12766.34802 |
| 4305 | GALNT6 | Q8NCL4 | 12764.92503 |
| 4306 | ISOC2 | Q96AB3 | 12747.82389 |
| 4307 | XAB2 | Q9HCS7 | 12723.00345 |
| 4308 | GSPT2 | Q8IYD1 | 12718.881 |
| 4309 | BCAS3 | Q9H6U6 | 12715.25513 |
| 4310 | PRKD2 | Q9BZL6 | 12712.11716 |
| 4311 | CENPV | Q7Z7K6 | 12683.48123 |
| 4312 | RNF113A | O15541 | 12675.05529 |
| 4313 | COPRS | Q9NQ92 | 12663.66683 |
| 4314 | CEP152 | O94986 | 12660.74498 |
| 4315 | FAM126A | Q9BYI3 | 12659.87131 |
| 4316 | MCL1 | Q07820 | 12657.34267 |
| 4317 | FDX1 | P10109 | 12615.90969 |
| 4318 | AKAP9 | Q99996 | 12612.32815 |
| 4319 | TEX10 | Q9NXF1 | 12602.7202 |
| 4320 | TSPYL1 | Q9H0U9 | 12590.55395 |
| 4321 | WIPI1 | Q5MNZ9 | 12587.80824 |
| 4322 | CELF2 | O95319 | 12576.68361 |
| 4323 | COPS9 | Q8WXC6 | 12575.501 |
| 4324 | PBK | Q96KB5 | 12571.38737 |
| 4325 | RELCH | Q9P260 | 12569.8851 |
| 4326 | SLC30A9 | Q6PML9 | 12560.27941 |
| 4327 | POLD1 | P28340 | 12552.63528 |
| 4328 | DAP | P51397 | 12545.12467 |
| 4329 | APAF1 | O14727 | 12540.50016 |
| 4330 | GGA3 | Q9NZ52 | 12537.28156 |
| 4331 | S1PR1 | P21453 | 12533.90831 |
| 4332 | PDE3A | Q14432 | 12530.52063 |
| 4333 | NAT1 | P18440 | 12494.29456 |
| 4334 | STX16 | O14662 | 12486.45844 |
| 4335 | HAUS1 | Q96CS2 | 12485.08156 |
| 4336 | CHD6 | Q8TD26 | 12481.67393 |
| 4337 | APIP | Q96GX9 | 12475.14765 |
| 4338 | TTC27 | Q6P3X3 | 12438.70815 |
| 4339 | PRPS2 | P11908 | 12421.59381 |
| 4340 | HMGCS1 | Q01581 | 12399.36688 |
| 4341 | MERTK | Q12866 | 12399.2713 |
| 4342 | PPP3CC | P48454 | 12399.22629 |
| 4343 | ANKRD13D | Q6ZTN6 | 12392.80704 |
| 4344 | F8A1 | P23610 | 12392.44685 |
| 4345 | C1orf174 | Q8IYL3 | 12377.71643 |
| 4346 | FMNL2 | Q96PY5 | 12364.70084 |
| 4347 | TTC19 | Q6DKK2 | 12363.53648 |
| 4348 | PTPRK | Q15262 | 12346.76504 |
| 4349 | GNG5 | P63218 | 12339.625 |
| 4350 | RABGAP1L | Q5R372 | 12328.34194 |
| 4351 | SRR | Q9GZT4 | 12315.29188 |
| 4352 | GAS1 | P54826 | 12307.94688 |
| 4353 | TOE1 | Q96GM8 | 12305.4715 |
| 4354 | TANK | Q92844 | 12299.25595 |
| 4355 | NARF | Q9UHQ1 | 12281.26196 |
| 4356 | FAR2 | Q96K12 | 12269.12607 |
| 4357 | PLEKHA5 | Q9HAU0 | 12253.16302 |
| 4358 | TRIM26 | Q12899 | 12253.13286 |
| 4359 | THTPA | Q9BU02 | 12246.60307 |
| 4360 | PCM1 | Q15154 | 12244.95427 |
| 4361 | GOLGA1 | Q92805 | 12239.84639 |
| 4362 | PTRH1 | Q86Y79 | 12237.32233 |
| 4363 | XIAP | P98170 | 12233.91285 |
| 4364 | PHLDA2 | Q53GA4 | 12230.49229 |
| 4365 | SIN3A | Q96ST3 | 12222.23781 |
| 4366 | FNDC3A | Q9Y2H6 | 12218.79048 |
| 4367 | PLCD1 | P51178 | 12208.88909 |
| 4368 | BORCS6 | Q96GS4 | 12202.23663 |
| 4369 | MZT2B | Q6NZ67 | 12198.64086 |
| 4370 | KDELR3 | O43731 | 12197.20222 |
| 4371 | POLR2D | O15514 | 12194.51744 |
| 4372 | ITPKC | Q96DU7 | 12187.35052 |
| 4373 | DOCK5 | Q9H7D0 | 12176.39365 |
| 4374 | AQR | O60306 | 12162.6101 |
| 4375 | XPO4 | Q9C0E2 | 12153.77816 |
| 4376 | LLGL1 | Q15334 | 12144.26276 |
| 4377 | CRADD | P78560 | 12134.25789 |
| 4378 | RPP25 | Q9BUL9 | 12124.2645 |
| 4379 | PDXP | Q96GD0 | 12122.82411 |
| 4380 | HAUS5 | O94927 | 12116.95261 |
| 4381 | SIPA1 | Q96FS4 | 12113.54472 |
| 4382 | SHROOM4 | Q9ULL8 | 12111.09108 |
| 4383 | NISCH | Q9Y2I1 | 12106.07307 |
| 4384 | NMI | Q13287 | 12105.38781 |
| 4385 | SLC35A2 | P78381 | 12096.83967 |
| 4386 | LYRM2 | Q9NU23 | 12091.012 |
| 4387 | RCOR1 | Q9UKL0 | 12086.71448 |
| 4388 | OPA3 | Q9H6K4 | 12084.00933 |
| 4389 | AHR | P35869 | 12066.8704 |
| 4390 | HEATR3 | Q7Z4Q2 | 12063.33829 |
| 4391 | ATG13 | O75143 | 12061.3645 |
| 4392 | INTS12 | Q96CB8 | 12046.00973 |
| 4393 | PTAR1 | Q7Z6K3 | 12045.776 |
| 4394 | ANKZF1 | Q9H8Y5 | 12044.85346 |
| 4395 | URB2 | Q14146 | 12004.95861 |
| 4396 | RFK | Q969G6 | 12003.94986 |
| 4397 | TMEM237 | Q96Q45 | 12003.25617 |
| 4398 | BORCS8 | Q96FH0 | 12002.85857 |
| 4399 | LAMA5 | O15230 | 11998.49772 |
| 4400 | GCC1 | Q96CN9 | 11994.9606 |
| 4401 | TAP1 | Q03518 | 11993.50484 |
| 4402 | MRTFB | Q9ULH7 | 11983.893 |
| 4403 | EXTL2 | Q9UBQ6 | 11980.243 |
| 4404 | ARHGEF28 | Q8N1W1 | 11979.89757 |
| 4405 | MBLAC2 | Q68D91 | 11966.61825 |
| 4406 | NUB1 | Q9Y5A7 | 11962.47793 |
| 4407 | REL | Q04864 | 11955.77809 |
| 4408 | TAF5 | Q15542 | 11939.19211 |
| 4409 | MAN2C1 | Q9NTJ4 | 11938.71455 |
| 4410 | TELO2 | Q9Y4R8 | 11929.37021 |
| 4411 | TUBA1A | Q71U36 | 11912.29968 |
| 4412 | YEATS4 | O95619 | 11898.73293 |
| 4413 | ZCCHC10 | Q8TBK6 | 11895.46717 |
| 4414 | NSMCE4A | Q9NXX6 | 11881.96685 |
| 4415 | EYA3 | Q99504 | 11880.7169 |
| 4416 | GLTP | Q9NZD2 | 11866.75364 |
| 4417 | C8orf82 | Q6P1X6 | 11864.20623 |
| 4418 | SNX8 | Q9Y5X2 | 11855.2783 |
| 4419 | PEX3 | P56589 | 11852.56489 |
| 4420 | BLOC1S4 | Q9NUP1 | 11850.87275 |
| 4421 | FGB | P02675 | 11850.75828 |
| 4422 | HSPB11 | Q9Y547 | 11846.68183 |
| 4423 | TAPBPL | Q9BX59 | 11839.29831 |
| 4424 | ASPSCR1 | Q9BZE9 | 11838.91652 |
| 4425 | P4HA3 | Q7Z4N8 | 11822.95475 |
| 4426 | MPP1 | Q00013 | 11820.90956 |
| 4427 | IRF2BP2 | Q7Z5L9 | 11818.38327 |
| 4428 | METAP2 | P50579 | 11810.98709 |
| 4429 | DIDO1 | Q9BTC0 | 11798.78644 |
| 4430 | NDFIP1 | Q9BT67 | 11783.4858 |
| 4431 | ZMYND8 | Q9ULU4 | 11769.63785 |
| 4432 | CCPG1 | Q9ULG6 | 11762.12567 |
| 4433 | RBM33 | Q96EV2 | 11747.47511 |
| 4434 | CNOT6L | Q96LI5 | 11734.73261 |
| 4435 | GGA2 | Q9UJY4 | 11732.83648 |
| 4436 | KIAA0753 | Q2KHM9 | 11732.15063 |
| 4437 | UPRT | Q96BW1 | 11731.71892 |
| 4438 | FES | P07332 | 11721.24488 |
| 4439 | PHKG2 | P15735 | 11706.41905 |
| 4440 | NAA20 | P61599 | 11694.06633 |
| 4441 | QRSL1 | Q9H0R6 | 11693.1083 |
| 4442 | GDPD1 | Q8N9F7 | 11691.012 |
| 4443 | UBAP1 | Q9NZ09 | 11676.59227 |
| 4444 | SACS | Q9NZJ4 | 11670.32739 |
| 4445 | MEST | Q5EB52 | 11666.53523 |
| 4446 | TMEM209 | Q96SK2 | 11660.42332 |
| 4447 | TLE7 | A0A1W2PR48 | 11651.38571 |
| 4448 | DHODH | Q02127 | 11642.28009 |
| 4449 | C5orf24 | Q7Z6I8 | 11632.9794 |
| 4450 | PPP2R5B | Q15173 | 11629.53431 |
| 4451 | GMPR | P36959 | 11628.80665 |
| 4452 | AGPAT2 | O15120 | 11612.72331 |
| 4453 | PRKCI | P41743 | 11586.98973 |
| 4454 | MANSC1 | Q9H8J5 | 11583.17153 |
| 4455 | MFGE8 | Q08431 | 11576.50646 |
| 4456 | NT5DC1 | Q5TFE4 | 11558.92687 |
| 4457 | B4GAT1 | O43505 | 11544.10882 |
| 4458 | OXSM | Q9NWU1 | 11533.55729 |
| 4459 | COL5A2 | P05997 | 11531.49404 |
| 4460 | ASRGL1 | Q7L266 | 11500.9105 |
| 4461 | BRD4 | O60885 | 11491.69375 |
| 4462 | PARP12 | Q9H0J9 | 11474.11738 |
| 4463 | RNF114 | Q9Y508 | 11468.48 |
| 4464 | ROBO4 | Q8WZ75 | 11467.29019 |
| 4465 | DMAC2L | Q99766 | 11462.98642 |
| 4466 | GFM2 | Q969S9 | 11462.21149 |
| 4467 | DNAAF10 | Q96MX6 | 11459.4756 |
| 4468 | DDX19B | Q9UMR2 | 11422.36077 |
| 4469 | BTD | P43251 | 11418.30639 |
| 4470 | CARD8 | Q9Y2G2 | 11406.13805 |
| 4471 | ITGB6 | P18564 | 11393.89231 |
| 4472 | ME3 | Q16798 | 11393.20366 |
| 4473 | PBRM1 | Q86U86 | 11365.23131 |
| 4474 | RAPGEF2 | Q9Y4G8 | 11350.95695 |
| 4475 | GPX7 | Q96SL4 | 11345.71482 |
| 4476 | MEX3D | Q86XN8 | 11343.31605 |
| 4477 | CASK | O14936 | 11340.57988 |
| 4478 | NUP188 | Q5SRE5 | 11327.49716 |
| 4479 | MARK2 | Q7KZI7 | 11326.05918 |
| 4480 | PXDC1 | Q5TGL8 | 11321.66207 |
| 4481 | NDUFAF5 | Q5TEU4 | 11312.71094 |
| 4482 | PIP4K2C | Q8TBX8 | 11296.9167 |
| 4483 | MCRS1 | Q96EZ8 | 11282.57654 |
| 4484 | AUH | Q13825 | 11272.86437 |
| 4485 | UBR5 | O95071 | 11265.51066 |
| 4486 | PLD2 | O14939 | 11264.53381 |
| 4487 | CDR2L | Q86X02 | 11260.89748 |
| 4488 | CD320 | Q9NPF0 | 11254.51778 |
| 4489 | DDX10 | Q13206 | 11236.12529 |
| 4490 | GNL3L | Q9NVN8 | 11231.2613 |
| 4491 | PARN | O95453 | 11216.68087 |
| 4492 | HPS6 | Q86YV9 | 11216.50779 |
| 4493 | PTPN14 | Q15678 | 11206.88889 |
| 4494 | SH3RF1 | Q7Z6J0 | 11201.55418 |
| 4495 | SLC35E1 | Q96K37 | 11187.8834 |
| 4496 | FPGS | Q05932 | 11182.16582 |
| 4497 | TAOK2 | Q9UL54 | 11174.99443 |
| 4498 | FAM219A | Q8IW50 | 11173.67363 |
| 4499 | DGLUCY | Q7Z3D6 | 11172.53807 |
| 4500 | TPBG | Q13641 | 11167.81539 |
| 4501 | ERBIN | Q96RT1 | 11152.57215 |
| 4502 | TRIM4 | Q9C037 | 11142.01857 |
| 4503 | RSF1 | Q96T23 | 11119.20213 |
| 4504 | PIP4K2B | P78356 | 11119.13243 |
| 4505 | SLC17A5 | Q9NRA2 | 11105.31506 |
| 4506 | MRM3 | Q9HC36 | 11093.45 |
| 4507 | RGN | Q15493 | 11093.24705 |
| 4508 | ABHD11 | Q8NFV4 | 11085.48044 |
| 4509 | CDK6 | Q00534 | 11054.11758 |
| 4510 | MT-ND4 | P03905 | 11049.87644 |
| 4511 | RFX1 | P22670 | 11030.5552 |
| 4512 | BIRC6 | Q9NR09 | 11028.69619 |
| 4513 | EEF1AKMT2 | Q5JPI9 | 11022.70892 |
| 4514 | PNPLA2 | Q96AD5 | 11015.89263 |
| 4515 | LRCH4 | O75427 | 11015.59 |
| 4516 | MAN1A2 | O60476 | 11009.07432 |
| 4517 | SLC4A7 | Q9Y6M7 | 11008.61081 |
| 4518 | SHMT1 | P34896 | 11007.4917 |
| 4519 | MICU1 | Q9BPX6 | 10997.26704 |
| 4520 | ADNP | Q9H2P0 | 10990.49305 |
| 4521 | MAPRE3 | Q9UPY8 | 10987.508 |
| 4522 | CIAO1 | O76071 | 10982.46376 |
| 4523 | FNBP4 | Q8N3X1 | 10975.45031 |
| 4524 | ZNF428 | Q96B54 | 10958.0176 |
| 4525 | SCAF4 | O95104 | 10955.95008 |
| 4526 | CHCHD5 | Q9BSY4 | 10948.53 |
| 4527 | TNRC6A | Q8NDV7 | 10940.41977 |
| 4528 | CALCRL | Q16602 | 10939.19521 |
| 4529 | CYBC1 | Q9BQA9 | 10937.13556 |
| 4530 | FRAS1 | Q86XX4 | 10936.84242 |
| 4531 | IPO8 | O15397 | 10932.07617 |
| 4532 | ZNF512 | Q96ME7 | 10919.65344 |
| 4533 | HAX1 | O00165 | 10917.38794 |
| 4534 | RCC1L | Q96I51 | 10912.01392 |
| 4535 | BLOC1S3 | Q6QNY0 | 10894.41385 |
| 4536 | AKR7A3 | O95154 | 10881.66213 |
| 4537 | MICU2 | Q8IYU8 | 10880.9298 |
| 4538 | EFCAB5 | A4FU69 | 10876.03788 |
| 4539 | HDAC6 | Q9UBN7 | 10871.77703 |
| 4540 | DCTN6 | O00399 | 10851.92914 |
| 4541 | GRAMD1A | Q96CP6 | 10844.29153 |
| 4542 | HEATR6 | Q6AI08 | 10836.00568 |
| 4543 | CALML5 | Q9NZT1 | 10817.16767 |
| 4544 | COQ6 | Q9Y2Z9 | 10804.429 |
| 4545 | AFAP1L1 | Q8TED9 | 10803.86125 |
| 4546 | ZFYVE1 | Q9HBF4 | 10798.20952 |
| 4547 | TRAPPC8 | Q9Y2L5 | 10796.73567 |
| 4548 | ATP6V0A2 | Q9Y487 | 10795.38573 |
| 4549 | GALK2 | Q01415 | 10792.80582 |
| 4550 | SLMAP | Q14BN4 | 10783.78087 |
| 4551 | SNX11 | Q9Y5W9 | 10768.46614 |
| 4552 | CTR9 | Q6PD62 | 10761.47518 |
| 4553 | ARG2 | P78540 | 10761.06353 |
| 4554 | YPEL5 | P62699 | 10708.56371 |
| 4555 | ZZEF1 | O43149 | 10697.95208 |
| 4556 | LIG1 | P18858 | 10696.47916 |
| 4557 | GTF2H1 | P32780 | 10676.22207 |
| 4558 | GID8 | Q9NWU2 | 10674.40817 |
| 4559 | ODR4 | Q5SWX8 | 10661.58372 |
| 4560 | EMC9 | Q9Y3B6 | 10656.71411 |
| 4561 | TOP2A | P11388 | 10655.18215 |
| 4562 | RILPL2 | Q969X0 | 10653.24431 |
| 4563 | SMURF2 | Q9HAU4 | 10629.1268 |
| 4564 | ELP5 | Q8TE02 | 10586.7578 |
| 4565 | ACOT11 | Q8WXI4 | 10585.98039 |
| 4566 | C7orf25 | Q9BPX7 | 10584.62676 |
| 4567 | RNASET2 | O00584 | 10572.73286 |
| 4568 | TAMM41 | Q96BW9 | 10569.58738 |
| 4569 | CDC42EP4 | Q9H3Q1 | 10567.80913 |
| 4570 | VPS8 | Q8N3P4 | 10564.15823 |
| 4571 | ARAF | P10398 | 10561.79394 |
| 4572 | ITCH | Q96J02 | 10559.6228 |
| 4573 | WARS2 | Q9UGM6 | 10528.70089 |
| 4574 | DNAJC30 | Q96LL9 | 10520.35546 |
| 4575 | TYW5 | A2RUC4 | 10519.19475 |
| 4576 | TRIM33 | Q9UPN9 | 10474.89013 |
| 4577 | RABAC1 | Q9UI14 | 10459.95 |
| 4578 | GPSM1 | Q86YR5 | 10449.39903 |
| 4579 | BAG1 | Q99933 | 10432.18019 |
| 4580 | ANKMY2 | Q8IV38 | 10425.10696 |
| 4581 | RETREG2 | Q8NC44 | 10420.04583 |
| 4582 | MTMR12 | Q9C0I1 | 10410.02969 |
| 4583 | UBR1 | Q8IWV7 | 10409.36792 |
| 4584 | PBLD | P30039 | 10398.50865 |
| 4585 | HSPB8 | Q9UJY1 | 10391.307 |
| 4586 | STBD1 | O95210 | 10377.93492 |
| 4587 | KCTD5 | Q9NXV2 | 10370.87871 |
| 4588 | F10 | P00742 | 10335.39908 |
| 4589 | C11orf54 | Q9H0W9 | 10334.13607 |
| 4590 | APOB | P04114 | 10325.62107 |
| 4591 | RNF14 | Q9UBS8 | 10310.19919 |
| 4592 | PPP4R3B | Q5MIZ7 | 10302.75332 |
| 4593 | MMAB | Q96EY8 | 10295.26115 |
| 4594 | CDC42EP3 | Q9UKI2 | 10290.85673 |
| 4595 | PHAF1 | Q9BSU1 | 10282.45632 |
| 4596 | FYN | P06241 | 10280.96959 |
| 4597 | EED | O75530 | 10280.64371 |
| 4598 | ZDHHC3 | Q9NYG2 | 10263.27623 |
| 4599 | CPNE7 | Q9UBL6 | 10250.42443 |
| 4600 | ACBD5 | Q5T8D3 | 10238.21146 |
| 4601 | PTPN9 | P43378 | 10237.09834 |
| 4602 | AVL9 | Q8NBF6 | 10235.75635 |
| 4603 | SLC35F2 | Q8IXU6 | 10231.11281 |
| 4604 | COL8A1 | P27658 | 10221.23355 |
| 4605 | ACP6 | Q9NPH0 | 10219.9612 |
| 4606 | SERPIND1 | P05546 | 10217.04333 |
| 4607 | ABHD12 | Q8N2K0 | 10210.63077 |
| 4608 | KNOP1 | Q1ED39 | 10203.04874 |
| 4609 | NOTCH1 | P46531 | 10202.83626 |
| 4610 | C1orf198 | Q9H425 | 10197.50425 |
| 4611 | TPX2 | Q9ULW0 | 10188.61464 |
| 4612 | DCAF6 | Q58WW2 | 10168.92623 |
| 4613 | GATA4 | P43694 | 10166.3005 |
| 4614 | SLC36A2 | Q495M3 | 10164.911 |
| 4615 | HAL | P42357 | 10158.76962 |
| 4616 | COLEC12 | Q5KU26 | 10157.06659 |
| 4617 | LRBA | P50851 | 10142.87777 |
| 4618 | TUBGCP4 | Q9UGJ1 | 10138.73532 |
| 4619 | ABCC4 | O15439 | 10137.78711 |
| 4620 | TMEM87A | Q8NBN3 | 10126.79671 |
| 4621 | TNIP1 | Q15025 | 10125.93423 |
| 4622 | COPZ2 | Q9P299 | 10116.51956 |
| 4623 | PTK7 | Q13308 | 10113.37048 |
| 4624 | LIX1L | Q8IVB5 | 10109.27088 |
| 4625 | SBF1 | O95248 | 10106.50206 |
| 4626 | APOA1 | P02647 | 10091.27167 |
| 4627 | EIF1B | O60739 | 10078.018 |
| 4628 | GNPTG | Q9UJJ9 | 10068.77167 |
| 4629 | GNB4 | Q9HAV0 | 10053.72188 |
| 4630 | APBB1 | O00213 | 10046.301 |
| 4631 | ACE | P12821 | 10045.08654 |
| 4632 | ETV6 | P41212 | 10043.29095 |
| 4633 | SRFBP1 | Q8NEF9 | 10028.03288 |
| 4634 | TRMT1L | Q7Z2T5 | 10024.90263 |
| 4635 | MYH11 | P35749 | 10018.78191 |
| 4636 | GNB1L | Q9BYB4 | 10012.00556 |
| 4637 | RAPGEF1 | Q13905 | 10011.21444 |
| 4638 | UPF3B | Q9BZI7 | 10007.75924 |
| 4639 | SLC7A6 | Q92536 | 10005.05545 |
| 4640 | MIDEAS | Q6PJG2 | 9996.381735 |
| 4641 | FOXO1 | Q12778 | 9989.966696 |
| 4642 | TRIM13 | O60858 | 9970.248042 |
| 4643 | FRK | P42685 | 9962.096563 |
| 4644 | ANKRD13A | Q8IZ07 | 9946.644 |
| 4645 | METTL1 | Q9UBP6 | 9937.545583 |
| 4646 | SMARCD1 | Q96GM5 | 9932.630862 |
| 4647 | HTT | P42858 | 9931.552527 |
| 4648 | ZFPL1 | O95159 | 9928.80025 |
| 4649 | MOCS3 | O95396 | 9926.53916 |
| 4650 | DCAF8 | Q5TAQ9 | 9926.300383 |
| 4651 | L3MBTL3 | Q96JM7 | 9912.293 |
| 4652 | TCEAL3 | Q969E4 | 9902.019877 |
| 4653 | SMG8 | Q8ND04 | 9893.12163 |
| 4654 | RPF2 | Q9H7B2 | 9892.288 |
| 4655 | LRCH1 | Q9Y2L9 | 9885.183848 |
| 4656 | TFIP11 | Q9UBB9 | 9871.551837 |
| 4657 | NR2C2 | P49116 | 9869.075212 |
| 4658 | SCD | O00767 | 9869.056429 |
| 4659 | ABLIM1 | O14639 | 9858.788894 |
| 4660 | GEMIN7 | Q9H840 | 9854.82 |
| 4661 | PHACTR2 | O75167 | 9854.604444 |
| 4662 | LRRK1 | Q38SD2 | 9839.31433 |
| 4663 | KIAA1217 | Q5T5P2 | 9835.162168 |
| 4664 | RSPRY1 | Q96DX4 | 9833.755741 |
| 4665 | NFYA | P23511 | 9830.175 |
| 4666 | POLR3D | P05423 | 9827.82044 |
| 4667 | C3 | P01024 | 9816.883082 |
| 4668 | EARS2 | Q5JPH6 | 9815.260333 |
| 4669 | NRBF2 | Q96F24 | 9810.4266 |
| 4670 | CDKN1A | P38936 | 9803.62336 |
| 4671 | DOCK10 | Q96BY6 | 9795.36864 |
| 4672 | PHKB | Q93100 | 9792.996633 |
| 4673 | TTL | Q8NG68 | 9786.70535 |
| 4674 | GEMIN4 | P57678 | 9784.857558 |
| 4675 | PTPRE | P23469 | 9777.642824 |
| 4676 | PARD3 | Q8TEW0 | 9773.636864 |
| 4677 | KIF1B | O60333 | 9749.037979 |
| 4678 | ESM1 | Q9NQ30 | 9743.338286 |
| 4679 | TMEM223 | A0PJW6 | 9741.881111 |
| 4680 | NIPA1 | Q7RTP0 | 9727.624 |
| 4681 | MRGBP | Q9NV56 | 9722.861111 |
| 4682 | XPC | Q01831 | 9717.180133 |
| 4683 | ACSF3 | Q4G176 | 9716.552943 |
| 4684 | TRIO | O75962 | 9693.11772 |
| 4685 | PPP2R5C | Q13362 | 9689.87724 |
| 4686 | ARFRP1 | Q13795 | 9675.349667 |
| 4687 | CRTC3 | Q6UUV7 | 9672.22815 |
| 4688 | FAS | P25445 | 9669.978126 |
| 4689 | NCOR2 | Q9Y618 | 9656.418632 |
| 4690 | CKM | P06732 | 9651.0456 |
| 4691 | ARHGAP22 | Q7Z5H3 | 9648.466676 |
| 4692 | ACAD8 | Q9UKU7 | 9644.13112 |
| 4693 | TSC2 | P49815 | 9642.672411 |
| 4694 | SLC26A2 | P50443 | 9642.306115 |
| 4695 | MLH1 | P40692 | 9641.12528 |
| 4696 | INPP4A | Q96PE3 | 9640.764667 |
| 4697 | MORF4L1 | Q9UBU8 | 9639.74 |
| 4698 | TBPL1 | P62380 | 9637.169444 |
| 4699 | MTIF2 | P46199 | 9631.137375 |
| 4700 | SEC14L1 | Q92503 | 9629.307462 |
| 4701 | SCARB1 | Q8WTV0 | 9619.394583 |
| 4702 | HAUS3 | Q68CZ6 | 9603.462226 |
| 4703 | ESF1 | Q9H501 | 9593.859263 |
| 4704 | TRNAU1AP | Q9NX07 | 9585.875455 |
| 4705 | CDR2 | Q01850 | 9564.924565 |
| 4706 | AP4S1 | Q9Y587 | 9564.208571 |
| 4707 | GOLPH3 | Q9H4A6 | 9541.911588 |
| 4708 | MAP3K7 | O43318 | 9540.912597 |
| 4709 | DAPK1 | P53355 | 9538.712113 |
| 4710 | SAMD4A | Q9UPU9 | 9537.443906 |
| 4711 | TRIM27 | P14373 | 9511.194458 |
| 4712 | VAV2 | P52735 | 9510.56586 |
| 4713 | RHOT1 | Q8IXI2 | 9510.40897 |
| 4714 | TBC1D20 | Q96BZ9 | 9498.569364 |
| 4715 | RBCK1 | Q9BYM8 | 9497.3215 |
| 4716 | IGF2BP1 | Q9NZI8 | 9489.368167 |
| 4717 | AIF1L | Q9BQI0 | 9484.678125 |
| 4718 | MAN1B1 | Q9UKM7 | 9469.096212 |
| 4719 | SDF2L1 | Q9HCN8 | 9462 |
| 4720 | ARIH2 | O95376 | 9452.346984 |
| 4721 | MAP4K2 | Q12851 | 9446.420316 |
| 4722 | KIF21A | Q7Z4S6 | 9445.758214 |
| 4723 | TRAF7 | Q6Q0C0 | 9430.938947 |
| 4724 | MED31 | Q9Y3C7 | 9429.595286 |
| 4725 | LIG4 | P49917 | 9428.471432 |
| 4726 | IRAK3 | Q9Y616 | 9422.008719 |
| 4727 | SYNC | Q9H7C4 | 9418.89828 |
| 4728 | FADS2 | O95864 | 9414.950222 |
| 4729 | FHL3 | Q13643 | 9408.683786 |
| 4730 | ABRACL | Q9P1F3 | 9407.376667 |
| 4731 | SLC25A29 | Q8N8R3 | 9401.986765 |
| 4732 | LTN1 | O94822 | 9389.672992 |
| 4733 | EIF2AK4 | Q9P2K8 | 9372.925398 |
| 4734 | CHM | P24386 | 9361.854125 |
| 4735 | GTPBP6 | O43824 | 9342.173148 |
| 4736 | CACNG6 | Q9BXT2 | 9331.072727 |
| 4737 | NAA16 | Q6N069 | 9327.470208 |
| 4738 | QSOX1 | O00391 | 9324.326711 |
| 4739 | PLXNA4 | Q9HCM2 | 9322.30811 |
| 4740 | ARMCX2 | Q7L311 | 9319.546857 |
| 4741 | DLGAP5 | Q15398 | 9315.63325 |
| 4742 | MAN2A2 | P49641 | 9311.478818 |
| 4743 | COA7 | Q96BR5 | 9294.611133 |
| 4744 | FRMD4A | Q9P2Q2 | 9290.272471 |
| 4745 | GPRC5A | Q8NFJ5 | 9283.772308 |
| 4746 | ABLIM3 | O94929 | 9280.955166 |
| 4747 | NR3C1 | P04150 | 9255.379668 |
| 4748 | MRPS18B | Q9Y676 | 9244.551786 |
| 4749 | MAPK7 | Q13164 | 9234.363781 |
| 4750 | PTS | Q03393 | 9211.001 |
| 4751 | PLAUR | Q03405 | 9207.451765 |
| 4752 | ITFG1 | Q8TB96 | 9203.234632 |
| 4753 | BTAF1 | O14981 | 9189.595931 |
| 4754 | FAM50B | Q9Y247 | 9188.753529 |
| 4755 | DAG1 | Q14118 | 9181.479209 |
| 4756 | ZNF148 | Q9UQR1 | 9153.156876 |
| 4757 | MOSPD2 | Q8NHP6 | 9152.702815 |
| 4758 | POLD3 | Q15054 | 9151.96556 |
| 4759 | KIN | O60870 | 9141.757917 |
| 4760 | ANKRD17 | O75179 | 9135.500799 |
| 4761 | VPS41 | P49754 | 9132.825783 |
| 4762 | SYNE2 | Q8WXH0 | 9132.454137 |
| 4763 | GPBP1L1 | Q9HC44 | 9122.923192 |
| 4764 | C19orf12 | Q9NSK7 | 9113.549 |
| 4765 | ZDHHC5 | Q9C0B5 | 9106.582344 |
| 4766 | RBM19 | Q9Y4C8 | 9105.273935 |
| 4767 | ACSF2 | Q96CM8 | 9081.772048 |
| 4768 | POLR2M | P0CAP2 | 9080.819909 |
| 4769 | DCBLD1 | Q8N8Z6 | 9080.818528 |
| 4770 | TK1 | P04183 | 9075.603538 |
| 4771 | GUF1 | Q8N442 | 9074.8358 |
| 4772 | ATF6B | Q99941 | 9064.723 |
| 4773 | EFR3A | Q14156 | 9051.18668 |
| 4774 | UBE2J1 | Q9Y385 | 9048.783067 |
| 4775 | TAMALIN | Q7Z6J2 | 9014.333895 |
| 4776 | PRRC2B | Q5JSZ5 | 9013.458648 |
| 4777 | TCIM | Q9NR00 | 9008.53125 |
| 4778 | ADGRL2 | O95490 | 8988.282453 |
| 4779 | OSBPL6 | Q9BZF3 | 8987.94162 |
| 4780 | CADPS2 | Q86UW7 | 8987.297535 |
| 4781 | SCO1 | O75880 | 8981.683133 |
| 4782 | GBA2 | Q9HCG7 | 8976.716978 |
| 4783 | PGGHG | Q32M88 | 8975.613656 |
| 4784 | PRPF39 | Q86UA1 | 8962.015212 |
| 4785 | BRAP | Q7Z569 | 8953.459576 |
| 4786 | DNAJC21 | Q5F1R6 | 8937.441591 |
| 4787 | TMCC3 | Q9ULS5 | 8934.511896 |
| 4788 | TOMM6 | Q96B49 | 8930.006 |
| 4789 | ATAT1 | Q5SQI0 | 8921.979208 |
| 4790 | DPY19L4 | Q7Z388 | 8910.148036 |
| 4791 | CTNNA2 | P26232 | 8905.710286 |
| 4792 | BRAT1 | Q6PJG6 | 8903.574844 |
| 4793 | DAAM1 | Q9Y4D1 | 8890.769869 |
| 4794 | PCMTD1 | Q96MG8 | 8890.555294 |
| 4795 | TRAPPC9 | Q96Q05 | 8887.768571 |
| 4796 | POLR3A | O14802 | 8882.11742 |
| 4797 | IPO11 | Q9UI26 | 8881.946609 |
| 4798 | GAS2L1 | Q99501 | 8880.8169 |
| 4799 | RB1CC1 | Q8TDY2 | 8870.448291 |
| 4800 | NIN | Q8N4C6 | 8870.011564 |
| 4801 | STAG2 | Q8N3U4 | 8868.611226 |
| 4802 | FBXO3 | Q9UK99 | 8866.381842 |
| 4803 | MARK1 | Q9P0L2 | 8860.854565 |
| 4804 | TMEM126B | Q8IUX1 | 8856.254545 |
| 4805 | STK11 | Q15831 | 8838.984056 |
| 4806 | GTF3C6 | Q969F1 | 8813.81125 |
| 4807 | CASP10 | Q92851 | 8813.502087 |
| 4808 | PARL | Q9H300 | 8798.371467 |
| 4809 | MAP3K20 | Q9NYL2 | 8794.451348 |
| 4810 | GRAMD1C | Q8IYS0 | 8791.279231 |
| 4811 | CNOT10 | Q9H9A5 | 8766.967667 |
| 4812 | RIOK3 | O14730 | 8755.352846 |
| 4813 | KRT16 | P08779 | 8744.859452 |
| 4814 | MKI67 | P46013 | 8737.559119 |
| 4815 | NUDT4B | A0A024RBG1 | 8731.02 |
| 4816 | ANAPC2 | Q9UJX6 | 8724.827622 |
| 4817 | CEP44 | Q9C0F1 | 8722.254979 |
| 4818 | INTS1 | Q8N201 | 8717.868779 |
| 4819 | UTP20 | O75691 | 8717.035104 |
| 4820 | NR2F2 | P24468 | 8711.6946 |
| 4821 | KRT14 | P02533 | 8699.443867 |
| 4822 | VWA8 | A3KMH1 | 8696.359927 |
| 4823 | UBE2J2 | Q8N2K1 | 8682.4743 |
| 4824 | MMAA | Q8IVH4 | 8675.059826 |
| 4825 | CHKB | Q9Y259 | 8670.398652 |
| 4826 | RAB1C | Q92928 | 8662.219286 |
| 4827 | CACTIN | Q8WUQ7 | 8659.990034 |
| 4828 | SQLE | Q14534 | 8659.54336 |
| 4829 | MPHOSPH8 | Q99549 | 8655.888273 |
| 4830 | PTBP3 | O95758 | 8654.252789 |
| 4831 | WDR45 | Q9Y484 | 8647.605389 |
| 4832 | RAB31 | Q13636 | 8637.477769 |
| 4833 | TSC1 | Q92574 | 8636.034949 |
| 4834 | GLRX5 | Q86SX6 | 8630.397714 |
| 4835 | OSBPL11 | Q9BXB4 | 8627.142038 |
| 4836 | ESS2 | Q96DF8 | 8624.81868 |
| 4837 | RND3 | P61587 | 8618.025455 |
| 4838 | CAND2 | O75155 | 8612.261915 |
| 4839 | NME7 | Q9Y5B8 | 8601.996571 |
| 4840 | PRKAB2 | O43741 | 8577.125 |
| 4841 | SLC30A6 | Q6NXT4 | 8572.795067 |
| 4842 | TARBP2 | Q15633 | 8567.981479 |
| 4843 | MICA | Q29983 | 8560.525 |
| 4844 | ARF1 | P84077 | 8559.1341 |
| 4845 | NOL12 | Q9UGY1 | 8555.74 |
| 4846 | WDR47 | O94967 | 8545.72174 |
| 4847 | LCLAT1 | Q6UWP7 | 8531.127696 |
| 4848 | THBD | P07204 | 8530.874583 |
| 4849 | OSBPL8 | Q9BZF1 | 8524.055154 |
| 4850 | SUMO3 | P55854 | 8518.119333 |
| 4851 | MED16 | Q9Y2X0 | 8513.323357 |
| 4852 | TBC1D2B | Q9UPU7 | 8503.095623 |
| 4853 | ANLN | Q9NQW6 | 8499.899724 |
| 4854 | ST3GAL6 | Q9Y274 | 8490.650667 |
| 4855 | QSOX2 | Q6ZRP7 | 8486.451659 |
| 4856 | PSEN1 | P49768 | 8484.530421 |
| 4857 | ARVCF | O00192 | 8482.04996 |
| 4858 | NDEL1 | Q9GZM8 | 8476.63213 |
| 4859 | CDC26 | Q8NHZ8 | 8473.0712 |
| 4860 | UBE2L6 | O14933 | 8469.913667 |
| 4861 | FAM91A1 | Q658Y4 | 8461.959205 |
| 4862 | OXR1 | Q8N573 | 8440.517689 |
| 4863 | CTCF | P49711 | 8432.797778 |
| 4864 | MDFIC | Q9P1T7 | 8430.30624 |
| 4865 | WDR43 | Q15061 | 8415.555719 |
| 4866 | TAF5L | O75529 | 8411.681875 |
| 4867 | CAMKK2 | Q96RR4 | 8395.429563 |
| 4868 | ENTPD5 | O75356 | 8393.8455 |
| 4869 | BTBD2 | Q9BX70 | 8391.311095 |
| 4870 | THSD4 | Q6ZMP0 | 8383.836538 |
| 4871 | CEP43 | O95684 | 8376.657006 |
| 4872 | TUBB1 | Q9H4B7 | 8375.651409 |
| 4873 | CCDC90B | Q9GZT6 | 8374.421222 |
| 4874 | TFE3 | P19532 | 8367.720278 |
| 4875 | ATM | Q13315 | 8361.925006 |
| 4876 | SOGA1 | O94964 | 8351.381313 |
| 4877 | SHCBP1 | Q8NEM2 | 8343.999657 |
| 4878 | LPGAT1 | Q92604 | 8341.72255 |
| 4879 | STAG1 | Q8WVM7 | 8339.201429 |
| 4880 | DHCR24 | Q15392 | 8335.280455 |
| 4881 | MEF2A | Q02078 | 8312.010714 |
| 4882 | CORO1A | P31146 | 8307.326476 |
| 4883 | SYNRG | Q9UMZ2 | 8303.202267 |
| 4884 | MAP3K11 | Q16584 | 8294.308585 |
| 4885 | ZFAND6 | Q6FIF0 | 8283.616875 |
| 4886 | IGSF8 | Q969P0 | 8282.177423 |
| 4887 | ARHGEF18 | Q6ZSZ5 | 8275.706966 |
| 4888 | ORAI1 | Q96D31 | 8268.758333 |
| 4889 | LIPA | P38571 | 8262.306417 |
| 4890 | ARFGEF2 | Q9Y6D5 | 8239.962412 |
| 4891 | POLR3C | Q9BUI4 | 8235.253097 |
| 4892 | MLYCD | O95822 | 8230.236133 |
| 4893 | ERCC6 | P0DP91 | 8225.07095 |
| 4894 | THG1L | Q9NWX6 | 8207.960294 |
| 4895 | MBNL2 | Q5VZF2 | 8206.45125 |
| 4896 | BAZ1A | Q9NRL2 | 8200.468493 |
| 4897 | RPP25L | Q8N5L8 | 8187.8 |
| 4898 | PDCD2 | Q16342 | 8187.445733 |
| 4899 | COL6A1 | P12109 | 8182.158481 |
| 4900 | SBNO1 | A3KN83 | 8178.264141 |
| 4901 | POLE4 | Q9NR33 | 8175.176 |
| 4902 | TXN2 | Q99757 | 8167.130625 |
| 4903 | MTMR1 | Q13613 | 8165.418 |
| 4904 | TMEM255B | Q8WV15 | 8164.494286 |
| 4905 | UTP11 | Q9Y3A2 | 8152.875909 |
| 4906 | TGOLN2 | O43493 | 8144.577933 |
| 4907 | SPOUT1 | Q5T280 | 8142.678737 |
| 4908 | DPH7 | Q9BTV6 | 8139.9705 |
| 4909 | BTN2A1 | Q7KYR7 | 8130.85108 |
| 4910 | B3GAT3 | O94766 | 8125.367667 |
| 4911 | FAM8A1 | Q9UBU6 | 8122.766263 |
| 4912 | KRR1 | Q13601 | 8116.332833 |
| 4913 | BAD | Q92934 | 8108.749 |
| 4914 | ORC5 | O43913 | 8102.34037 |
| 4915 | UCK1 | Q9HA47 | 8086.860833 |
| 4916 | TAB2 | Q9NYJ8 | 8082.420364 |
| 4917 | RGP1 | Q92546 | 8081.968619 |
| 4918 | LDAH | Q9H6V9 | 8075.001882 |
| 4919 | CMTR1 | Q8N1G2 | 8058.80951 |
| 4920 | CBR4 | Q8N4T8 | 8057.580091 |
| 4921 | MIF4GD | A9UHW6 | 8057.5266 |
| 4922 | PDGFA | P04085 | 8051.369 |
| 4923 | SHISA4 | Q96DD7 | 8040.745714 |
| 4924 | FUNDC2 | Q9BWH2 | 8039.121333 |
| 4925 | DNAJB2 | P25686 | 8025.023813 |
| 4926 | RAP2C | Q9Y3L5 | 8018.881083 |
| 4927 | THEM4 | Q5T1C6 | 8013.566154 |
| 4928 | KRT73 | Q86Y46 | 8005.561733 |
| 4929 | CPEB2 | Q7Z5Q1 | 7996.023846 |
| 4930 | A1BG | P04217 | 7992.138125 |
| 4931 | DTNBP1 | Q96EV8 | 7990.178833 |
| 4932 | SMYD5 | Q6GMV2 | 7986.5055 |
| 4933 | CCDC71L | Q8N9Z2 | 7985.284769 |
| 4934 | COIL | P38432 | 7984.699185 |
| 4935 | KIFAP3 | Q92845 | 7983.914182 |
| 4936 | RPTOR | Q8N122 | 7982.424306 |
| 4937 | GFER | P55789 | 7977.928556 |
| 4938 | AP1G2 | O75843 | 7976.575806 |
| 4939 | RNF11 | Q9Y3C5 | 7947.06125 |
| 4940 | SLC38A2 | Q96QD8 | 7935.898615 |
| 4941 | EGFL7 | Q9UHF1 | 7932.879167 |
| 4942 | SCD5 | Q86SK9 | 7915.786917 |
| 4943 | COX7B | P24311 | 7913.6 |
| 4944 | PAXBP1 | Q9Y5B6 | 7912.628217 |
| 4945 | KIF13A | Q9H1H9 | 7911.750181 |
| 4946 | GAB2 | Q9UQC2 | 7906.390848 |
| 4947 | ANAPC5 | Q9UJX4 | 7905.877993 |
| 4948 | PITPNM1 | O00562 | 7905.672639 |
| 4949 | TAOK1 | Q7L7X3 | 7902.565275 |
| 4950 | WIPI2 | Q9Y4P8 | 7889.55855 |
| 4951 | IQGAP3 | Q86VI3 | 7882.180554 |
| 4952 | INTS9 | Q9NV88 | 7855.747586 |
| 4953 | CSNK1E | P49674 | 7839.391318 |
| 4954 | GSTA4 | O15217 | 7839.107692 |
| 4955 | PXK | Q7Z7A4 | 7838.096565 |
| 4956 | ARHGAP35 | Q9NRY4 | 7825.202138 |
| 4957 | IL1RAP | Q9NPH3 | 7822.929581 |
| 4958 | RGS19 | P49795 | 7821.853333 |
| 4959 | ZCCHC3 | Q9NUD5 | 7813.558652 |
| 4960 | AGA | P20933 | 7813.345765 |
| 4961 | CD46 | P15529 | 7803.765083 |
| 4962 | GPRC5B | Q9NZH0 | 7797.267583 |
| 4963 | TMEM9 | Q9P0T7 | 7793.9412 |
| 4964 | CEP41 | Q9BYV8 | 7786.331684 |
| 4965 | FCHSD1 | Q86WN1 | 7773.005594 |
| 4966 | KIF4A | O95239 | 7770.647203 |
| 4967 | PUM2 | Q8TB72 | 7751.876486 |
| 4968 | GFPT2 | O94808 | 7741.327381 |
| 4969 | ERCC6L | Q2NKX8 | 7740.279545 |
| 4970 | ZC3HAV1L | Q96H79 | 7733.23038 |
| 4971 | WASHC2A | Q641Q2 | 7730.367908 |
| 4972 | CA13 | Q8N1Q1 | 7724.681 |
| 4973 | TMEM159 | Q96B96 | 7716.32775 |
| 4974 | DYNLL2 | Q96FJ2 | 7707.1178 |
| 4975 | ATF6 | P18850 | 7697.911207 |
| 4976 | ULBP2 | Q9BZM5 | 7696.756714 |
| 4977 | NKIRAS2 | Q9NYR9 | 7693.721667 |
| 4978 | CASKIN2 | Q8WXE0 | 7687.462289 |
| 4979 | OSBPL2 | Q9H1P3 | 7684.427393 |
| 4980 | PHC2 | Q8IXK0 | 7681.283292 |
| 4981 | WDFY3 | Q8IZQ1 | 7680.814692 |
| 4982 | LDLRAP1 | Q5SW96 | 7671.867 |
| 4983 | IP6K1 | Q92551 | 7667.198696 |
| 4984 | MVK | Q03426 | 7665.523722 |
| 4985 | KIF2C | Q99661 | 7663.75975 |
| 4986 | NUF2 | Q9BZD4 | 7655.818269 |
| 4987 | TRPM4 | Q8TD43 | 7651.947373 |
| 4988 | CHCHD1 | Q96BP2 | 7645.336667 |
| 4989 | CLU | P10909 | 7644.683421 |
| 4990 | IVNS1ABP | Q9Y6Y0 | 7628.345433 |
| 4991 | SLC25A30 | Q5SVS4 | 7627.735786 |
| 4992 | GRPEL2 | Q8TAA5 | 7622.586833 |
| 4993 | CTSL | P07711 | 7610.498462 |
| 4994 | PHIP | Q8WWQ0 | 7587.711035 |
| 4995 | TBC1D10B | Q4KMP7 | 7585.243907 |
| 4996 | STAT5B | P51692 | 7584.036897 |
| 4997 | BLOC1S5 | Q8TDH9 | 7570.054091 |
| 4998 | DUS3L | Q96G46 | 7568.181891 |
| 4999 | FZD6 | O60353 | 7564.606057 |
| 5000 | LRCH2 | Q5VUJ6 | 7558.198053 |
| 5001 | GYS2 | P54840 | 7549.765 |
| 5002 | LRATD2 | Q96KN1 | 7542.316063 |
| 5003 | MAK | P20794 | 7535.301211 |
| 5004 | ANAPC1 | Q9H1A4 | 7534.987044 |
| 5005 | CIAO3 | Q9H6Q4 | 7511.669111 |
| 5006 | TAF7 | Q15545 | 7510.768789 |
| 5007 | IMPACT | Q9P2X3 | 7508.508824 |
| 5008 | ARID1B | Q8NFD5 | 7508.130562 |
| 5009 | SLIT1 | O75093 | 7503.158033 |
| 5010 | MARK3 | P27448 | 7491.304163 |
| 5011 | CLEC11A | Q9Y240 | 7486.12725 |
| 5012 | SUMO2 | P61956 | 7482.8675 |
| 5013 | DYM | Q7RTS9 | 7458.497962 |
| 5014 | C9orf78 | Q9NZ63 | 7457.264063 |
| 5015 | RUFY3 | Q7L099 | 7456.140346 |
| 5016 | SENP8 | Q96LD8 | 7455.8096 |
| 5017 | AKAP17A | Q02040 | 7455.223656 |
| 5018 | SAP130 | Q9H0E3 | 7455.134275 |
| 5019 | TAF8 | Q7Z7C8 | 7454.2085 |
| 5020 | ARHGAP10 | A1A4S6 | 7431.150213 |
| 5021 | PTPRG | P23470 | 7425.881257 |
| 5022 | TMEM132A | Q24JP5 | 7424.461657 |
| 5023 | RHBDD2 | Q6NTF9 | 7421.108273 |
| 5024 | ANAPC4 | Q9UJX5 | 7418.257081 |
| 5025 | SMARCA2 | P51531 | 7414.697831 |
| 5026 | GDF15 | Q99988 | 7414.129222 |
| 5027 | RMND1 | Q9NWS8 | 7392.833111 |
| 5028 | DOK1 | Q99704 | 7392.050652 |
| 5029 | SHKBP1 | Q8TBC3 | 7383.795333 |
| 5030 | AGO1 | Q9UL18 | 7365.013705 |
| 5031 | C1GALT1C1 | Q96EU7 | 7359.942313 |
| 5032 | SHROOM2 | Q13796 | 7359.600696 |
| 5033 | OCC1 | Q8TAD7 | 7359.331333 |
| 5034 | BUD31 | P41223 | 7357.634 |
| 5035 | GTF2A2 | P52657 | 7355.87125 |
| 5036 | REXO4 | Q9GZR2 | 7351.229858 |
| 5037 | SYNJ1 | O43426 | 7348.070591 |
| 5038 | PARP2 | Q9UGN5 | 7341.508296 |
| 5039 | KATNB1 | Q9BVA0 | 7339.665514 |
| 5040 | DENND3 | A2RUS2 | 7339.071766 |
| 5041 | HTRA3 | P83110 | 7321.292588 |
| 5042 | CDK2AP1 | O14519 | 7319.05325 |
| 5043 | GPATCH11 | Q8N954 | 7315.947867 |
| 5044 | C17orf75 | Q9HAS0 | 7306.238348 |
| 5045 | STXBP5 | Q5T5C0 | 7303.331383 |
| 5046 | CSTF2T | Q9H0L4 | 7283.037179 |
| 5047 | CSN1S1 | P02662 | 7278.7769 |
| 5048 | SCRN2 | Q96FV2 | 7264.4698 |
| 5049 | SNIP1 | Q8TAD8 | 7259.125722 |
| 5050 | ABL1 | P00519 | 7256.613229 |
| 5051 | ATRX | P46100 | 7254.702465 |
| 5052 | POP4 | O95707 | 7253.886889 |
| 5053 | PACSIN3 | Q9UKS6 | 7251.881318 |
| 5054 | NSUN6 | Q8TEA1 | 7248.889667 |
| 5055 | YY2 | O15391 | 7244.200833 |
| 5056 | NAA80 | Q93015 | 7240.831182 |
| 5057 | POU2F1 | P14859 | 7235.827 |
| 5058 | TTYH3 | Q9C0H2 | 7228.200512 |
| 5059 | PPP6R2 | O75170 | 7222.876549 |
| 5060 | IRF2BPL | Q9H1B7 | 7222.67573 |
| 5061 | TRAF6 | Q9Y4K3 | 7220.634923 |
| 5062 | RABEPK | Q7Z6M1 | 7217.628095 |
| 5063 | KDM2A | Q9Y2K7 | 7214.215254 |
| 5064 | SNUPN | O95149 | 7209.687412 |
| 5065 | MTMR10 | Q9NXD2 | 7208.250489 |
| 5066 | VANGL1 | Q8TAA9 | 7200.440448 |
| 5067 | SFXN5 | Q8TD22 | 7192.528214 |
| 5068 | CAMSAP2 | Q08AD1 | 7191.243236 |
| 5069 | TRIM67 | Q6ZTA4 | 7184.507179 |
| 5070 | ATG9A | Q7Z3C6 | 7180.9285 |
| 5071 | DUSP6 | Q16828 | 7180.718471 |
| 5072 | ZEB1 | P37275 | 7170.779333 |
| 5073 | NADK | O95544 | 7166.2812 |
| 5074 | CLIP2 | Q9UDT6 | 7147.769162 |
| 5075 | EPB41L1 | Q9H4G0 | 7144.488577 |
| 5076 | TRAPPC13 | A5PLN9 | 7140.731095 |
| 5077 | SLC1A4 | P43007 | 7140.489895 |
| 5078 | MED10 | Q9BTT4 | 7138.12875 |
| 5079 | TP53BP2 | Q13625 | 7138.056479 |
| 5080 | SMG6 | Q86US8 | 7136.905593 |
| 5081 | NUP42 | O15504 | 7135.073588 |
| 5082 | PEX13 | Q92968 | 7131.087313 |
| 5083 | GRK2 | P25098 | 7128.361333 |
| 5084 | ADGRG1 | Q9Y653 | 7126.345414 |
| 5085 | ANKHD1 | Q8IWZ3 | 7124.112933 |
| 5086 | SPIN1 | Q9Y657 | 7120.041714 |
| 5087 | MRPS5 | P82675 | 7107.5008 |
| 5088 | BTBD10 | Q9BSF8 | 7094.543478 |
| 5089 | RPRD2 | Q5VT52 | 7080.923297 |
| 5090 | SSH3 | Q8TE77 | 7071.749567 |
| 5091 | SUGP2 | Q8IX01 | 7071.715714 |
| 5092 | MAP2K7 | O14733 | 7071.603045 |
| 5093 | CLEC2B | Q92478 | 7070.331429 |
| 5094 | CREBZF | Q9NS37 | 7065.427895 |
| 5095 | ATAD3B | Q5T9A4 | 7057.944139 |
| 5096 | UBE2F | Q969M7 | 7049.044444 |
| 5097 | LPCAT3 | Q6P1A2 | 7045.858412 |
| 5098 | LRWD1 | Q9UFC0 | 7040.606625 |
| 5099 | RAMAC | Q9BTL3 | 7037.1275 |
| 5100 | OARD1 | Q9Y530 | 7029.448182 |
| 5101 | KDM3B | Q7LBC6 | 7024.525927 |
| 5102 | LCN1 | P31025 | 7019.895 |
| 5103 | MB21D2 | Q8IYB1 | 7019.645964 |
| 5104 | SHE | Q5VZ18 | 7012.84676 |
| 5105 | TPST1 | O60507 | 7006.541889 |
| 5106 | SLC16A14 | Q7RTX9 | 6993.622385 |
| 5107 | CTU1 | Q7Z7A3 | 6991.10533 |
| 5108 | SDE2 | Q6IQ49 | 6987.328208 |
| 5109 | DHRSX | Q8N5I4 | 6984.372947 |
| 5110 | TGFBRAP1 | Q8WUH2 | 6984.128452 |
| 5111 | MYL12B | O14950 | 6983.477222 |
| 5112 | GAN | Q9H2C0 | 6980.605737 |
| 5113 | TMEM222 | Q9H0R3 | 6977.8536 |
| 5114 | INIP | Q9NRY2 | 6971.0024 |
| 5115 | TRMT5 | Q32P41 | 6967.264306 |
| 5116 | ZGPAT | Q8N5A5 | 6963.425367 |
| 5117 | SLC22A10 | Q63ZE4 | 6950.790952 |
| 5118 | VIRMA | Q69YN4 | 6947.826384 |
| 5119 | INPP5A | Q14642 | 6947.576261 |
| 5120 | FADD | Q13158 | 6936.49875 |
| 5121 | PDK1 | Q15118 | 6931.36912 |
| 5122 | ACVR1 | Q04771 | 6896.273684 |
| 5123 | RALBP1 | Q15311 | 6896.030567 |
| 5124 | ELP6 | Q0PNE2 | 6891.315632 |
| 5125 | CHSY1 | Q86X52 | 6883.205356 |
| 5126 | MORC2 | Q9Y6X9 | 6875.228923 |
| 5127 | ZNF687 | Q8N1G0 | 6865.152182 |
| 5128 | ANTXR2 | P58335 | 6864.516652 |
| 5129 | TRAPPC11 | Q7Z392 | 6861.166535 |
| 5130 | BCL2L12 | Q9HB09 | 6851.764556 |
| 5131 | PHF23 | Q9BUL5 | 6842.325333 |
| 5132 | DLC1 | Q96QB1 | 6836.409282 |
| 5133 | PLEKHA2 | Q9HB19 | 6825.198 |
| 5134 | RBM15B | Q8NDT2 | 6824.552771 |
| 5135 | NFATC2IP | Q8NCF5 | 6820.677222 |
| 5136 | GRK4 | P32298 | 6813.828241 |
| 5137 | FAM168A | Q92567 | 6813.777 |
| 5138 | CEP170B | Q9Y4F5 | 6812.054919 |
| 5139 | VEZT | Q9HBM0 | 6804.021924 |
| 5140 | BCAR3 | O75815 | 6792.828417 |
| 5141 | ZMYM4 | Q5VZL5 | 6788.056473 |
| 5142 | SERINC1 | Q9NRX5 | 6773.247647 |
| 5143 | WFS1 | O76024 | 6770.495125 |
| 5144 | ATP11B | Q9Y2G3 | 6761.198333 |
| 5145 | RPAP1 | Q9BWH6 | 6754.788203 |
| 5146 | LUM | P51884 | 6739.219938 |
| 5147 | DNAJA4 | Q8WW22 | 6737.010909 |
| 5148 | ERF | P50548 | 6734.717136 |
| 5149 | SRPX2 | O60687 | 6732.217591 |
| 5150 | SIRPA | P78324 | 6731.291615 |
| 5151 | NAGPA | Q9UK23 | 6725.3855 |
| 5152 | TXNL4B | Q9NX01 | 6719.6425 |
| 5153 | ANKRD52 | Q8NB46 | 6715.680045 |
| 5154 | CACNB3 | P54284 | 6714.89468 |
| 5155 | FUCA2 | Q9BTY2 | 6714.64948 |
| 5156 | GTF3C4 | Q9UKN8 | 6714.529 |
| 5157 | POLR3F | Q9H1D9 | 6697.5015 |
| 5158 | GPR180 | Q86V85 | 6689.97 |
| 5159 | GATA6 | Q92908 | 6683.607235 |
| 5160 | SLC30A5 | Q8TAD4 | 6680.618241 |
| 5161 | CLMP | Q9H6B4 | 6666.30615 |
| 5162 | DDHD2 | O94830 | 6664.948387 |
| 5163 | FN3KRP | Q9HA64 | 6662.224571 |
| 5164 | REEP4 | Q9H6H4 | 6659.362333 |
| 5165 | ABT1 | Q9ULW3 | 6658.373529 |
| 5166 | KIF11 | P52732 | 6652.941766 |
| 5167 | EFNB2 | P52799 | 6650.549333 |
| 5168 | MEN1 | O00255 | 6638.605321 |
| 5169 | FASTKD2 | Q9NYY8 | 6633.105049 |
| 5170 | XPA | P23025 | 6630.803111 |
| 5171 | MORC3 | Q14149 | 6625.428041 |
| 5172 | CHD2 | O14647 | 6617.498616 |
| 5173 | UTP25 | Q68CQ4 | 6616.94835 |
| 5174 | OGFOD3 | Q6PK18 | 6615.874412 |
| 5175 | GPRASP2 | Q96D09 | 6608.732389 |
| 5176 | IREB2 | P48200 | 6607.570313 |
| 5177 | MED17 | Q9NVC6 | 6594.523848 |
| 5178 | CNOT11 | Q9UKZ1 | 6594.258783 |
| 5179 | GINS4 | Q9BRT9 | 6591.778714 |
| 5180 | CCDC97 | Q96F63 | 6588.605 |
| 5181 | MACO1 | Q8N5G2 | 6584.168118 |
| 5182 | AGO3 | Q9H9G7 | 6583.839279 |
| 5183 | MYL12A | P19105 | 6563.211444 |
| 5184 | TBRG4 | Q969Z0 | 6534.275176 |
| 5185 | GPATCH8 | Q9UKJ3 | 6534.239082 |
| 5186 | MTCL1 | Q9Y4B5 | 6518.917723 |
| 5187 | UBE2W | Q96B02 | 6518.80125 |
| 5188 | POTEKP | Q9BYX7 | 6508.059222 |
| 5189 | CCNB1 | P14635 | 6504.526762 |
| 5190 | TFPT | P0C1Z6 | 6504.280889 |
| 5191 | RMI2 | Q96E14 | 6502.29875 |
| 5192 | MED25 | Q71SY5 | 6500.276944 |
| 5193 | PIK3CA | P42336 | 6491.396047 |
| 5194 | TM6SF1 | Q9BZW5 | 6487.5586 |
| 5195 | FAM171A1 | Q5VUB5 | 6484.364686 |
| 5196 | SMC6 | Q96SB8 | 6478.443492 |
| 5197 | SDSL | Q96GA7 | 6478.290133 |
| 5198 | IPO13 | O94829 | 6474.5957 |
| 5199 | ZFYVE21 | Q9BQ24 | 6460.477091 |
| 5200 | RMC1 | Q96DM3 | 6454.107 |
| 5201 | DCAF5 | Q96JK2 | 6449.3679 |
| 5202 | BSCL2 | Q96G97 | 6446.776923 |
| 5203 | DHPS | P49366 | 6424.509238 |
| 5204 | ATXN1 | P54253 | 6420.999323 |
| 5205 | SLU7 | O95391 | 6416.391294 |
| 5206 | TAF9 | Q16594 | 6409.081706 |
| 5207 | CNNM3 | Q8NE01 | 6405.202564 |
| 5208 | BMP2K | Q9NSY1 | 6404.370674 |
| 5209 | CDH24 | Q86UP0 | 6404.002903 |
| 5210 | PLK1 | P53350 | 6403.391714 |
| 5211 | CSNK1G2 | P78368 | 6398.501 |
| 5212 | PLAU | P00749 | 6397.47248 |
| 5213 | CHML | P26374 | 6368.50525 |
| 5214 | CDYL | Q9Y232 | 6367.674269 |
| 5215 | PLEKHF1 | Q96S99 | 6367.2275 |
| 5216 | PSMG4 | Q5JS54 | 6353.67 |
| 5217 | ATP2B3 | Q16720 | 6348.609115 |
| 5218 | PI4KB | Q9UBF8 | 6343.393 |
| 5219 | NECTIN3 | Q9NQS3 | 6339.305905 |
| 5220 | NFIC | P08651 | 6334.81325 |
| 5221 | CHD8 | Q9HCK8 | 6326.454955 |
| 5222 | APBB2 | Q92870 | 6325.360054 |
| 5223 | C19orf47 | Q8N9M1 | 6323.68 |
| 5224 | NMRK1 | Q9NWW6 | 6319.891 |
| 5225 | ATF7IP | Q6VMQ6 | 6315.441227 |
| 5226 | TERF2 | Q15554 | 6307.496477 |
| 5227 | CRIM1 | Q9NZV1 | 6297.53506 |
| 5228 | NSMAF | Q92636 | 6296.625881 |
| 5229 | NID2 | Q14112 | 6281.134103 |
| 5230 | RBM6 | P78332 | 6275.688672 |
| 5231 | KHNYN | O15037 | 6270.028308 |
| 5232 | NVL | O15381 | 6264.700374 |
| 5233 | TRIM38 | O00635 | 6255.936316 |
| 5234 | RALGAPB | Q86X10 | 6249.038558 |
| 5235 | TNFAIP8L1 | Q8WVP5 | 6246.5309 |
| 5236 | MAST4 | O15021 | 6231.863677 |
| 5237 | MCUB | Q9NWR8 | 6231.750667 |
| 5238 | COX15 | Q7KZN9 | 6221.98515 |
| 5239 | KIAA1191 | Q96A73 | 6220.976053 |
| 5240 | BACE2 | Q9Y5Z0 | 6220.084684 |
| 5241 | SEPSECS | Q9HD40 | 6219.538519 |
| 5242 | CTDP1 | Q9Y5B0 | 6214.646096 |
| 5243 | ZWILCH | Q9H900 | 6204.224594 |
| 5244 | MAPKAP1 | Q9BPZ7 | 6199.521192 |
| 5245 | MIA2 | Q96PC5 | 6199.296469 |
| 5246 | ERCC4 | Q92889 | 6192.445694 |
| 5247 | HIP1 | O00291 | 6188.777298 |
| 5248 | CLMN | Q96JQ2 | 6183.60756 |
| 5249 | HABP4 | Q5JVS0 | 6183.5565 |
| 5250 | MTMR14 | Q8NCE2 | 6176.58431 |
| 5251 | ALKBH4 | Q9NXW9 | 6172.601071 |
| 5252 | EFCAB14 | O75071 | 6170.35737 |
| 5253 | SOAT1 | P35610 | 6163.865957 |
| 5254 | RBMX2 | Q9Y388 | 6161.500333 |
| 5255 | TARS2 | Q9BW92 | 6155.474128 |
| 5256 | DIAPH3 | Q9NSV4 | 6151.321828 |
| 5257 | MED24 | O75448 | 6144.3818 |
| 5258 | UQCC3 | Q6UW78 | 6135.54 |
| 5259 | KLHL22 | Q53GT1 | 6109.536758 |
| 5260 | MYO1B | O43795 | 6102.116548 |
| 5261 | DNM1 | Q05193 | 6094.752174 |
| 5262 | RBSN | Q9H1K0 | 6077.623028 |
| 5263 | MDN1 | Q9NU22 | 6075.452541 |
| 5264 | TXNIP | Q9H3M7 | 6073.552227 |
| 5265 | TJAP1 | Q5JTD0 | 6072.915821 |
| 5266 | TXLNG | Q9NUQ3 | 6062.02788 |
| 5267 | PITPNM3 | Q9BZ71 | 6046.27761 |
| 5268 | SRPK2 | P78362 | 6025.424103 |
| 5269 | NUBPL | Q8TB37 | 6023.8592 |
| 5270 | NOL3 | O60936 | 6014.665788 |
| 5271 | TMEM63A | O94886 | 6013.958844 |
| 5272 | GCSH | P23434 | 6011.681429 |
| 5273 | DDX31 | Q9H8H2 | 6009.191756 |
| 5274 | ISG20L2 | Q9H9L3 | 6009.11619 |
| 5275 | LENG8 | Q96PV6 | 6006.138789 |
| 5276 | GRAMD4 | Q6IC98 | 5996.147758 |
| 5277 | MYO18A | Q92614 | 5995.638596 |
| 5278 | CARMIL1 | Q5VZK9 | 5980.800556 |
| 5279 | ATP9A | O75110 | 5969.546642 |
| 5280 | HEATR5B | Q9P2D3 | 5952.664023 |
| 5281 | TOP3B | O95985 | 5951.128745 |
| 5282 | PARP14 | Q460N5 | 5946.589995 |
| 5283 | ARID4B | Q4LE39 | 5946.301567 |
| 5284 | ZKSCAN1 | P17029 | 5938.314731 |
| 5285 | MIER1 | Q8N108 | 5930.81872 |
| 5286 | DHX32 | Q7L7V1 | 5925.015667 |
| 5287 | TTC28 | Q96AY4 | 5923.151492 |
| 5288 | WAPL | Q7Z5K2 | 5921.218103 |
| 5289 | ABHD6 | Q9BV23 | 5919.688556 |
| 5290 | SLC38A10 | Q9HBR0 | 5917.663054 |
| 5291 | TFB2M | Q9H5Q4 | 5915.996458 |
| 5292 | HGSNAT | Q68CP4 | 5912.438174 |
| 5293 | CFAP410 | O43822 | 5912.275765 |
| 5294 | TRMT61A | Q96FX7 | 5905.767077 |
| 5295 | AGPAT4 | Q9NRZ5 | 5901.895 |
| 5296 | MTA3 | Q9BTC8 | 5901.239156 |
| 5297 | MCM3AP | O60318 | 5899.918135 |
| 5298 | TAF9B | Q9HBM6 | 5899.415533 |
| 5299 | LIMK2 | P53671 | 5880.481765 |
| 5300 | CHPT1 | Q8WUD6 | 5879.326154 |
| 5301 | NRBP2 | Q9NSY0 | 5879.161208 |
| 5302 | COL13A1 | Q5TAT6 | 5875.889895 |
| 5303 | ATP7A | Q04656 | 5874.814517 |
| 5304 | SLC12A7 | Q9Y666 | 5872.825047 |
| 5305 | EEF1AKNMT | Q8N6R0 | 5862.2703 |
| 5306 | MED22 | Q15528 | 5853.24775 |
| 5307 | SPC25 | Q9HBM1 | 5852.502636 |
| 5308 | DPH2 | Q9BQC3 | 5839.419619 |
| 5309 | DTX3L | Q8TDB6 | 5830.455051 |
| 5310 | FASTKD5 | Q7L8L6 | 5830.413268 |
| 5311 | ECSIT | Q9BQ95 | 5828.316217 |
| 5312 | UGCG | Q16739 | 5820.30605 |
| 5313 | MDC1 | Q14676 | 5818.061954 |
| 5314 | UBE2Q1 | Q7Z7E8 | 5813.4506 |
| 5315 | DPP8 | Q6V1X1 | 5809.856591 |
| 5316 | NSMCE3 | Q96MG7 | 5808.132095 |
| 5317 | TEX30 | Q5JUR7 | 5805.228182 |
| 5318 | ANTXR1 | Q9H6X2 | 5804.640182 |
| 5319 | JCAD | Q9P266 | 5804.375463 |
| 5320 | AP3M2 | P53677 | 5800.383545 |
| 5321 | DMAP1 | Q9NPF5 | 5796.87063 |
| 5322 | PLA2G4A | P47712 | 5794.197545 |
| 5323 | TNS2 | Q63HR2 | 5793.505507 |
| 5324 | TGS1 | Q96RS0 | 5792.831892 |
| 5325 | KATNA1 | O75449 | 5791.098333 |
| 5326 | SLC43A3 | Q8NBI5 | 5783.965714 |
| 5327 | LYZ | P61626 | 5780.739 |
| 5328 | RGCC | Q9H4X1 | 5778.5044 |
| 5329 | PRKAR1B | P31321 | 5777.742118 |
| 5330 | ARMH3 | Q5T2E6 | 5770.770379 |
| 5331 | PTPRA | P18433 | 5766.828868 |
| 5332 | GET1 | O00258 | 5753.729182 |
| 5333 | MSH3 | P20585 | 5750.702345 |
| 5334 | UROS | P10746 | 5743.479417 |
| 5335 | PLS1 | Q14651 | 5736.454171 |
| 5336 | SLC37A4 | O43826 | 5735.002538 |
| 5337 | VLDLR | P98155 | 5712.4944 |
| 5338 | ZSWIM8 | A7E2V4 | 5710.774338 |
| 5339 | DHX33 | Q9H6R0 | 5710.526613 |
| 5340 | RNF170 | Q96K19 | 5697.294333 |
| 5341 | CCDC88C | Q9P219 | 5692.934205 |
| 5342 | PPP1R13B | Q96KQ4 | 5688.95668 |
| 5343 | R3HCC1 | Q9Y3T6 | 5686.66219 |
| 5344 | SMARCAL1 | Q9NZC9 | 5685.906196 |
| 5345 | RAB3IP | Q96QF0 | 5683.223474 |
| 5346 | ID1 | P41134 | 5677.404556 |
| 5347 | TRMT13 | Q9NUP7 | 5657.455533 |
| 5348 | PDE4DIP | Q5VU43 | 5647.764787 |
| 5349 | CDYL2 | Q8N8U2 | 5647.0535 |
| 5350 | KDM5C | P41229 | 5645.683148 |
| 5351 | FITM2 | Q8N6M3 | 5640.111333 |
| 5352 | PGAP1 | Q75T13 | 5633.153378 |
| 5353 | AMMECR1L | Q6DCA0 | 5630.647667 |
| 5354 | LATS1 | O95835 | 5623.799 |
| 5355 | AVEN | Q9NQS1 | 5622.008591 |
| 5356 | JUND | P17535 | 5621.966417 |
| 5357 | APOBEC3C | Q9NRW3 | 5611.8776 |
| 5358 | CFAP36 | Q96G28 | 5602.291563 |
| 5359 | VPS37C | A5D8V6 | 5594.774375 |
| 5360 | MED21 | Q13503 | 5585.494833 |
| 5361 | IRAK1 | P51617 | 5580.10425 |
| 5362 | GNPAT | O15228 | 5579.061914 |
| 5363 | UBL7 | Q96S82 | 5568.131 |
| 5364 | CCNDBP1 | O95273 | 5567.817833 |
| 5365 | PARP9 | Q8IXQ6 | 5565.480378 |
| 5366 | MSRA | Q9UJ68 | 5563.207267 |
| 5367 | MINK1 | Q8N4C8 | 5560.858903 |
| 5368 | RIC1 | Q4ADV7 | 5558.0115 |
| 5369 | GPX3 | P22352 | 5553.584727 |
| 5370 | CDH3 | P22223 | 5548.88 |
| 5371 | TBC1D8B | Q0IIM8 | 5545.124294 |
| 5372 | IL17D | Q8TAD2 | 5542.888214 |
| 5373 | SPOCK1 | Q08629 | 5542.051905 |
| 5374 | CD99L2 | Q8TCZ2 | 5536.686545 |
| 5375 | PALD1 | Q9ULE6 | 5523.08879 |
| 5376 | SLC39A10 | Q9ULF5 | 5521.73471 |
| 5377 | FBXO38 | Q6PIJ6 | 5507.348298 |
| 5378 | PLXNB1 | O43157 | 5507.274125 |
| 5379 | KIF3B | O15066 | 5506.053643 |
| 5380 | INTS14 | Q96SY0 | 5505.763804 |
| 5381 | TCF25 | Q9BQ70 | 5502.215375 |
| 5382 | SAMD9 | Q5K651 | 5500.415727 |
| 5383 | EPB41 | P11171 | 5500.172818 |
| 5384 | CDK5RAP1 | Q96SZ6 | 5490.704219 |
| 5385 | CCND1 | P24385 | 5482.669421 |
| 5386 | ELOA | Q14241 | 5480.887142 |
| 5387 | TOM1L1 | O75674 | 5479.315286 |
| 5388 | IGHMBP2 | P38935 | 5478.386778 |
| 5389 | RIF1 | Q5UIP0 | 5478.100812 |
| 5390 | APTX | Q7Z2E3 | 5477.164778 |
| 5391 | LRIF1 | Q5T3J3 | 5475.261028 |
| 5392 | ISCU | Q9H1K1 | 5472.097333 |
| 5393 | SZRD1 | Q7Z422 | 5470.797 |
| 5394 | SH3TC1 | Q8TE82 | 5467.909915 |
| 5395 | FCHSD2 | O94868 | 5462.531925 |
| 5396 | KYAT1 | Q16773 | 5461.190714 |
| 5397 | RHBDF1 | Q96CC6 | 5458.959118 |
| 5398 | NUDT18 | Q6ZVK8 | 5445.204 |
| 5399 | GDF6 | Q6KF10 | 5444.644136 |
| 5400 | SNX19 | Q92543 | 5444.555375 |
| 5401 | KCTD15 | Q96SI1 | 5443.031278 |
| 5402 | LMBRD1 | Q9NUN5 | 5441.437632 |
| 5403 | DGKH | Q86XP1 | 5436.706502 |
| 5404 | PRDM2 | Q13029 | 5433.968312 |
| 5405 | NATD1 | Q8N6N6 | 5429.101429 |
| 5406 | MAFG | O15525 | 5428.645727 |
| 5407 | PHACTR4 | Q8IZ21 | 5421.744657 |
| 5408 | BRI3BP | Q8WY22 | 5420.7742 |
| 5409 | CIP2A | Q8TCG1 | 5419.208579 |
| 5410 | MT-ND5 | P03915 | 5417.470857 |
| 5411 | RICTOR | Q6R327 | 5414.640403 |
| 5412 | HMGN4 | O00479 | 5413.03525 |
| 5413 | ADPGK | Q9BRR6 | 5410.556335 |
| 5414 | CYTH2 | Q99418 | 5395.834524 |
| 5415 | KLF13 | Q9Y2Y9 | 5394.693077 |
| 5416 | PFKFB3 | Q16875 | 5385.197722 |
| 5417 | C6orf120 | Q7Z4R8 | 5382.165 |
| 5418 | RXRB | P28702 | 5374.757311 |
| 5419 | VPS13A | Q96RL7 | 5373.586228 |
| 5420 | SLC25A15 | Q9Y619 | 5362.603118 |
| 5421 | ASTE1 | Q2TB18 | 5361.317194 |
| 5422 | CARD10 | Q9BWT7 | 5357.448456 |
| 5423 | GXYLT1 | Q4G148 | 5349.872778 |
| 5424 | SYTL4 | Q96C24 | 5343.550895 |
| 5425 | ABCA8 | O94911 | 5332.944123 |
| 5426 | MBD3 | O95983 | 5325.497688 |
| 5427 | CBLL1 | Q75N03 | 5322.778421 |
| 5428 | ARRB2 | P32121 | 5321.6445 |
| 5429 | SIGIRR | Q6IA17 | 5320.165722 |
| 5430 | TK2 | O00142 | 5313.930875 |
| 5431 | R3HDM1 | Q15032 | 5306.878828 |
| 5432 | ZC3H7A | Q8IWR0 | 5300.88084 |
| 5433 | HELZ | P42694 | 5299.06127 |
| 5434 | TBC1D22B | Q9NU19 | 5297.448071 |
| 5435 | NACAD | O15069 | 5295.227833 |
| 5436 | ABCB6 | Q9NP58 | 5294.251105 |
| 5437 | MYCBP2 | O75592 | 5293.365383 |
| 5438 | MBLAC1 | A4D2B0 | 5278.7768 |
| 5439 | SERPINE2 | P07093 | 5276.400476 |
| 5440 | HDHD3 | Q9BSH5 | 5269.921077 |
| 5441 | WDHD1 | O75717 | 5262.570672 |
| 5442 | TRAFD1 | O14545 | 5261.429292 |
| 5443 | SRSF8 | Q9BRL6 | 5254.335875 |
| 5444 | USP19 | O94966 | 5251.139155 |
| 5445 | CLSTN1 | O94985 | 5238.278528 |
| 5446 | DCBLD2 | Q96PD2 | 5235.899588 |
| 5447 | SMAD1 | Q15797 | 5223.938182 |
| 5448 | PIK3IP1 | Q96FE7 | 5220.7272 |
| 5449 | HTATSF1 | O43719 | 5220.117648 |
| 5450 | FRMD5 | Q7Z6J6 | 5219.407667 |
| 5451 | FDX2 | Q6P4F2 | 5219.329875 |
| 5452 | FIG4 | Q92562 | 5218.52866 |
| 5453 | KRT6A | P02538 | 5209.1889 |
| 5454 | RNF5 | Q99942 | 5205.2755 |
| 5455 | MEF2C | Q06413 | 5204.945714 |
| 5456 | DGCR8 | Q8WYQ5 | 5190.379206 |
| 5457 | URGCP | Q8TCY9 | 5190.31388 |
| 5458 | NOL8 | Q76FK4 | 5183.928857 |
| 5459 | CHD3 | Q12873 | 5181.849828 |
| 5460 | CSNK1G3 | Q9Y6M4 | 5179.537 |
| 5461 | GON7 | Q9BXV9 | 5176.903 |
| 5462 | MIB1 | Q86YT6 | 5167.325346 |
| 5463 | ARL6IP1 | Q15041 | 5164.8695 |
| 5464 | SYNE1 | Q8NF91 | 5164.01299 |
| 5465 | STK39 | Q9UEW8 | 5156.1585 |
| 5466 | SRBD1 | Q8N5C6 | 5143.4723 |
| 5467 | TAF4 | O00268 | 5140.642306 |
| 5468 | SCAF1 | Q9H7N4 | 5138.343133 |
| 5469 | NME4 | O00746 | 5129.84 |
| 5470 | RFC1 | P35251 | 5126.1778 |
| 5471 | ERRFI1 | Q9UJM3 | 5121.655619 |
| 5472 | SPRY4 | Q9C004 | 5112.147846 |
| 5473 | TXNDC11 | Q6PKC3 | 5111.316511 |
| 5474 | BMP6 | P22004 | 5091.467447 |
| 5475 | SLC7A11 | Q9UPY5 | 5090.07025 |
| 5476 | ROGDI | Q9GZN7 | 5082.35005 |
| 5477 | USP13 | Q92995 | 5081.708929 |
| 5478 | C18orf25 | Q96B23 | 5081.044869 |
| 5479 | SLC49A4 | Q96SL1 | 5076.018889 |
| 5480 | NCAPG2 | Q86XI2 | 5074.915515 |
| 5481 | OSCP1 | Q8WVF1 | 5073.879583 |
| 5482 | CCM2 | Q9BSQ5 | 5068.9546 |
| 5483 | MEPCE | Q7L2J0 | 5064.863638 |
| 5484 | VASH1 | Q7L8A9 | 5060.944909 |
| 5485 | RPP40 | O75818 | 5052.811222 |
| 5486 | HSCB | Q8IWL3 | 5045.6675 |
| 5487 | RGS20 | O76081 | 5043.646323 |
| 5488 | EHHADH | Q08426 | 5037.356119 |
| 5489 | ASAH1 | Q13510 | 5029.244957 |
| 5490 | NDUFA7 | O95182 | 5027.226586 |
| 5491 | TNIK | Q9UKE5 | 5025.752015 |
| 5492 | FLVCR1 | Q9Y5Y0 | 5018.425 |
| 5493 | SENP1 | Q9P0U3 | 5016.617161 |
| 5494 | ALB | P02768 | 5016.060789 |
| 5495 | NHSL2 | Q5HYW2 | 5004.956794 |
| 5496 | RBBP6 | Q7Z6E9 | 4995.940755 |
| 5497 | TRIT1 | Q9H3H1 | 4992.23263 |
| 5498 | ETS1 | P14921 | 4989.769053 |
| 5499 | GTF3C2 | Q8WUA4 | 4984.929912 |
| 5500 | TAF12 | Q16514 | 4983.731667 |
| 5501 | TMEM259 | Q4ZIN3 | 4983.681238 |
| 5502 | STX2 | P32856 | 4978.5202 |
| 5503 | UNC13B | O14795 | 4978.128885 |
| 5504 | MYO10 | Q9HD67 | 4968.89667 |
| 5505 | MED23 | Q9ULK4 | 4968.776931 |
| 5506 | PLXNA3 | P51805 | 4957.960371 |
| 5507 | CERCAM | Q5T4B2 | 4956.460192 |
| 5508 | GALNT10 | Q86SR1 | 4953.485355 |
| 5509 | KRT84 | Q9NSB2 | 4947.515789 |
| 5510 | FTSJ1 | Q9UET6 | 4946.182 |
| 5511 | STXBP4 | Q6ZWJ1 | 4945.496233 |
| 5512 | FLI1 | Q01543 | 4936.94401 |
| 5513 | STARD10 | Q9Y365 | 4931.959417 |
| 5514 | MLF2 | Q15773 | 4920.3719 |
| 5515 | FIBP | O43427 | 4920.2021 |
| 5516 | ICA1 | Q05084 | 4917.748148 |
| 5517 | PRKACB | P22694 | 4915.609895 |
| 5518 | INTS6 | Q9UL03 | 4908.912902 |
| 5519 | TIMM10 | P62072 | 4899.268 |
| 5520 | TAX1BP1 | Q86VP1 | 4888.029457 |
| 5521 | GIPC2 | Q8TF65 | 4880.203308 |
| 5522 | PTPRM | P28827 | 4878.768545 |
| 5523 | MPC2 | O95563 | 4867.93725 |
| 5524 | PCBP4 | P57723 | 4865.620769 |
| 5525 | CDCP1 | Q9H5V8 | 4856.247929 |
| 5526 | SPG7 | Q9UQ90 | 4855.639098 |
| 5527 | KDELR1 | P24390 | 4852.683 |
| 5528 | FOXK2 | Q01167 | 4849.332185 |
| 5529 | SLC25A46 | Q96AG3 | 4843.213474 |
| 5530 | CCNH | P51946 | 4841.743389 |
| 5531 | PLEKHA7 | Q6IQ23 | 4840.781642 |
| 5532 | IFT74 | Q96LB3 | 4840.080975 |
| 5533 | CIZ1 | Q9ULV3 | 4835.369523 |
| 5534 | GARRE1 | O15063 | 4834.610563 |
| 5535 | TMED3 | Q9Y3Q3 | 4822.593917 |
| 5536 | SMDT1 | Q9H4I9 | 4820.913 |
| 5537 | GPAT4 | Q86UL3 | 4819.001364 |
| 5538 | FBXO4 | Q9UKT5 | 4805.709889 |
| 5539 | VMP1 | Q96GC9 | 4802.71895 |
| 5540 | UBE2T | Q9NPD8 | 4798.2928 |
| 5541 | NT5C | Q8TCD5 | 4787.757636 |
| 5542 | GTF2H4 | Q92759 | 4781.879174 |
| 5543 | JAM3 | Q9BX67 | 4781.568143 |
| 5544 | MTRR | Q9UBK8 | 4779.1095 |
| 5545 | ANAPC16 | Q96DE5 | 4768.781143 |
| 5546 | CPNE8 | Q86YQ8 | 4767.306966 |
| 5547 | PMF1 | Q6P1K2 | 4766.388692 |
| 5548 | SPRYD3 | Q8NCJ5 | 4765.092 |
| 5549 | ZC3H13 | Q5T200 | 4764.240686 |
| 5550 | SLC25A19 | Q9HC21 | 4757.535818 |
| 5551 | TM4SF18 | Q96CE8 | 4752.6515 |
| 5552 | TECPR1 | Q7Z6L1 | 4751.556068 |
| 5553 | DHX57 | Q6P158 | 4738.409288 |
| 5554 | RELB | Q01201 | 4734.32069 |
| 5555 | GFOD1 | Q9NXC2 | 4731.284 |
| 5556 | VAMP8 | Q9BV40 | 4727.102 |
| 5557 | SULF1 | Q8IWU6 | 4719.319256 |
| 5558 | HAUS6 | Q7Z4H7 | 4719.149925 |
| 5559 | CALCOCO1 | Q9P1Z2 | 4714.777324 |
| 5560 | SETDB1 | Q15047 | 4713.710016 |
| 5561 | CLK2 | P49760 | 4710.018 |
| 5562 | BOD1L1 | Q8NFC6 | 4698.3382 |
| 5563 | PGAP6 | Q9HCN3 | 4696.923 |
| 5564 | HSD17B8 | Q92506 | 4695.950167 |
| 5565 | TBC1D2 | Q9BYX2 | 4690.391484 |
| 5566 | CRTC1 | Q6UUV9 | 4689.56075 |
| 5567 | FAM210B | Q96KR6 | 4684.913143 |
| 5568 | COX7A1 | P24310 | 4670.7256 |
| 5569 | MFSD1 | Q9H3U5 | 4667.023571 |
| 5570 | RPAP2 | Q8IXW5 | 4663.46769 |
| 5571 | DAB2IP | Q5VWQ8 | 4660.413029 |
| 5572 | CACUL1 | Q86Y37 | 4659.303556 |
| 5573 | CMTM3 | Q96MX0 | 4647.51725 |
| 5574 | EPPK1 | P58107 | 4645.269309 |
| 5575 | N6AMT1 | Q9Y5N5 | 4638.321625 |
| 5576 | DNAJB5 | O75953 | 4634.083118 |
| 5577 | NCDN | Q9UBB6 | 4631.359813 |
| 5578 | NEPRO | Q6NW34 | 4618.444722 |
| 5579 | AP5Z1 | O43299 | 4615.237326 |
| 5580 | MICAL3 | Q7RTP6 | 4612.703077 |
| 5581 | PPP1R3D | O95685 | 4611.139316 |
| 5582 | MITD1 | Q8WV92 | 4610.142583 |
| 5583 | TRAPPC10 | P48553 | 4608.725074 |
| 5584 | SLC39A14 | Q15043 | 4602.405071 |
| 5585 | MTIF3 | Q9H2K0 | 4601.551563 |
| 5586 | ACOT8 | O14734 | 4599.062875 |
| 5587 | LRRC20 | Q8TCA0 | 4580.151242 |
| 5588 | PTDSS1 | P48651 | 4578.237048 |
| 5589 | PNMA1 | Q8ND90 | 4571.716316 |
| 5590 | TMEM120A | Q9BXJ8 | 4566.853733 |
| 5591 | CHAC2 | Q8WUX2 | 4566.606889 |
| 5592 | KIAA0319L | Q8IZA0 | 4560.034837 |
| 5593 | WRAP53 | Q9BUR4 | 4551.622304 |
| 5594 | TTC9C | Q8N5M4 | 4548.1394 |
| 5595 | ARHGEF17 | Q96PE2 | 4546.48402 |
| 5596 | RGS12 | O14924 | 4542.246415 |
| 5597 | FAM135B | Q49AJ0 | 4531.625238 |
| 5598 | ABCB8 | Q9NUT2 | 4530.533795 |
| 5599 | CMIP | Q8IY22 | 4519.670818 |
| 5600 | GGACT | Q9BVM4 | 4516.9705 |
| 5601 | AKAP10 | O43572 | 4509.191188 |
| 5602 | NOTCH2 | Q04721 | 4503.706134 |
| 5603 | TDRD3 | Q9H7E2 | 4499.105614 |
| 5604 | GEMIN2 | O14893 | 4495.907143 |
| 5605 | TIMMDC1 | Q9NPL8 | 4494.659063 |
| 5606 | NUBP1 | P53384 | 4494.6 |
| 5607 | PNPLA8 | Q9NP80 | 4491.992306 |
| 5608 | CETN3 | O15182 | 4491.784 |
| 5609 | PEX6 | Q13608 | 4484.377421 |
| 5610 | SLC22A8 | Q8TCC7 | 4474.48619 |
| 5611 | WWC2 | Q6AWC2 | 4472.168181 |
| 5612 | HVCN1 | Q96D96 | 4468.132636 |
| 5613 | ZFYVE16 | Q7Z3T8 | 4437.174852 |
| 5614 | CYLD | Q9NQC7 | 4436.71294 |
| 5615 | SHARPIN | Q9H0F6 | 4436.058625 |
| 5616 | LYSMD3 | Q7Z3D4 | 4433.664538 |
| 5617 | FUT11 | Q495W5 | 4430.971833 |
| 5618 | PLEKHM2 | Q8IWE5 | 4429.513227 |
| 5619 | DCP1B | Q8IZD4 | 4425.097379 |
| 5620 | F2 | P00734 | 4422.445323 |
| 5621 | MCEE | Q96PE7 | 4417.508545 |
| 5622 | ANKRD44 | Q8N8A2 | 4409.900511 |
| 5623 | FUOM | A2VDF0 | 4404.222286 |
| 5624 | PLEKHG2 | Q9H7P9 | 4396.92015 |
| 5625 | ZC3H18 | Q86VM9 | 4394.38034 |
| 5626 | MAD2L1BP | Q15013 | 4378.464538 |
| 5627 | NSMCE2 | Q96MF7 | 4378.440846 |
| 5628 | USP11 | P51784 | 4375.906313 |
| 5629 | CLEC16A | Q2KHT3 | 4373.185761 |
| 5630 | LIMD1 | Q9UGP4 | 4366.122533 |
| 5631 | ATE1 | O95260 | 4364.639 |
| 5632 | RACGAP1 | Q9H0H5 | 4360.393412 |
| 5633 | C1orf52 | Q8N6N3 | 4360.016667 |
| 5634 | PISD | Q9UG56 | 4359.053077 |
| 5635 | DNAJC16 | Q9Y2G8 | 4352.960242 |
| 5636 | EIF1AX | P47813 | 4346.68 |
| 5637 | ZHX1 | Q9UKY1 | 4339.387133 |
| 5638 | ARAP1 | Q96P48 | 4335.748333 |
| 5639 | TAPT1 | Q6NXT6 | 4333.714276 |
| 5640 | HSP90AB4P | Q58FF6 | 4329.174667 |
| 5641 | TENM3 | Q9P273 | 4327.127891 |
| 5642 | TXNDC15 | Q96J42 | 4324.670765 |
| 5643 | MSMO1 | Q15800 | 4320.0696 |
| 5644 | RTN1 | Q16799 | 4319.339567 |
| 5645 | SLC7A5 | Q01650 | 4319.002611 |
| 5646 | NFXL1 | Q6ZNB6 | 4312.023396 |
| 5647 | ZNF318 | Q5VUA4 | 4310.049981 |
| 5648 | AP4E1 | Q9UPM8 | 4303.992833 |
| 5649 | DVL2 | O14641 | 4301.148226 |
| 5650 | MGME1 | Q9BQP7 | 4280.58 |
| 5651 | KDM4B | O94953 | 4273.262854 |
| 5652 | PNISR | Q8TF01 | 4268.77913 |
| 5653 | RASAL2 | Q9UJF2 | 4268.505627 |
| 5654 | IFT43 | Q96FT9 | 4268.002636 |
| 5655 | PRXL2C | Q7RTV5 | 4266.652 |
| 5656 | RTF2 | Q9BY42 | 4248.602075 |
| 5657 | GPCPD1 | Q9NPB8 | 4242.687472 |
| 5658 | MET | P08581 | 4236.329462 |
| 5659 | MFSD5 | Q6N075 | 4234.703846 |
| 5660 | ARHGAP5 | Q13017 | 4231.078644 |
| 5661 | CCDC66 | A2RUB6 | 4227.510604 |
| 5662 | URB1 | O60287 | 4216.699673 |
| 5663 | CDC20 | Q12834 | 4214.832476 |
| 5664 | SLC38A7 | Q9NVC3 | 4211.699071 |
| 5665 | NHEJ1 | Q9H9Q4 | 4204.217111 |
| 5666 | GTPBP2 | Q9BX10 | 4201.35337 |
| 5667 | ZNFX1 | Q9P2E3 | 4189.729939 |
| 5668 | DICER1 | Q9UPY3 | 4189.695991 |
| 5669 | KCTD9 | Q7L273 | 4188.74005 |
| 5670 | PRKRIP1 | Q9H875 | 4179.374364 |
| 5671 | METTL25 | Q8N6Q8 | 4168.83797 |
| 5672 | OSBPL10 | Q9BXB5 | 4165.056054 |
| 5673 | ZNF800 | Q2TB10 | 4164.289818 |
| 5674 | TNFRSF10C | O14798 | 4164.079429 |
| 5675 | PRIM2 | P49643 | 4156.628379 |
| 5676 | CAPN7 | Q9Y6W3 | 4139.741396 |
| 5677 | HGFAC | Q04756 | 4139.501731 |
| 5678 | PHF14 | O94880 | 4138.470763 |
| 5679 | AKTIP | Q9H8T0 | 4137.016077 |
| 5680 | AFAP1L2 | Q8N4X5 | 4136.897692 |
| 5681 | MLLT1 | Q03111 | 4136.484286 |
| 5682 | CTNNAL1 | Q9UBT7 | 4132.161051 |
| 5683 | WDR91 | A4D1P6 | 4130.662675 |
| 5684 | NHSL1 | Q5SYE7 | 4130.558397 |
| 5685 | RHBDF2 | Q6PJF5 | 4128.52459 |
| 5686 | CDC40 | O60508 | 4128.415222 |
| 5687 | CROT | Q9UKG9 | 4128.169879 |
| 5688 | TDRD7 | Q8NHU6 | 4114.540607 |
| 5689 | MIS12 | Q9H081 | 4108.405 |
| 5690 | ST6GAL1 | P15907 | 4107.0737 |
| 5691 | ST6GALNAC4 | Q9H4F1 | 4101.040083 |
| 5692 | TRRAP | Q9Y4A5 | 4098.343335 |
| 5693 | LGALS9 | O00182 | 4089.02775 |
| 5694 | AKAP1 | Q92667 | 4087.7645 |
| 5695 | FOXC2 | Q99958 | 4084.066588 |
| 5696 | PLPP6 | Q8IY26 | 4081.046667 |
| 5697 | RYBP | Q8N488 | 4075.552 |
| 5698 | FAM124B | Q9H5Z6 | 4074.846259 |
| 5699 | HUS1 | O60921 | 4074.756077 |
| 5700 | WDR41 | Q9HAD4 | 4072.408 |
| 5701 | SGSM2 | O43147 | 4070.714884 |
| 5702 | EP400 | Q96L91 | 4064.003297 |
| 5703 | STARD4 | Q96DR4 | 4058.935909 |
| 5704 | HIRA | P54198 | 4058.091107 |
| 5705 | ERCC6 | Q03468 | 4053.684493 |
| 5706 | ZADH2 | Q8N4Q0 | 4053.1166 |
| 5707 | PPTC7 | Q8NI37 | 4050.463 |
| 5708 | TTC21B | Q7Z4L5 | 4043.944588 |
| 5709 | HCFC2 | Q9Y5Z7 | 4043.138733 |
| 5710 | CAPN5 | O15484 | 4041.722543 |
| 5711 | GTF3C1 | Q12789 | 4040.227634 |
| 5712 | RRP36 | Q96EU6 | 4032.287722 |
| 5713 | POLG | P54098 | 4030.378129 |
| 5714 | TSR3 | Q9UJK0 | 4024.359286 |
| 5715 | SUZ12 | Q15022 | 4022.686611 |
| 5716 | FOSL2 | P15408 | 4021.8235 |
| 5717 | HEATR5A | Q86XA9 | 4016.82452 |
| 5718 | DMXL1 | Q9Y485 | 4015.493453 |
| 5719 | RPP14 | O95059 | 4013.752667 |
| 5720 | ATF7 | P17544 | 4008.117176 |
| 5721 | CDCA8 | Q53HL2 | 4006.196571 |
| 5722 | RBPJ | Q06330 | 3995.228095 |
| 5723 | CDK13 | Q14004 | 3990.837401 |
| 5724 | PKD2 | Q13563 | 3983.601571 |
| 5725 | GOLM2 | Q6P4E1 | 3972.886928 |
| 5726 | TDRKH | Q9Y2W6 | 3965.8316 |
| 5727 | INTS8 | Q75QN2 | 3961.980207 |
| 5728 | TBC1D25 | Q3MII6 | 3955.418792 |
| 5729 | SBF2 | Q86WG5 | 3941.885033 |
| 5730 | CCDC102A | Q96A19 | 3936.804208 |
| 5731 | EBAG9 | O00559 | 3933.992571 |
| 5732 | NFKBIE | O00221 | 3930.174778 |
| 5733 | IQSEC1 | Q6DN90 | 3921.273863 |
| 5734 | PEX16 | Q9Y5Y5 | 3920.444294 |
| 5735 | DDX49 | Q9Y6V7 | 3916.788704 |
| 5736 | PTEN | P60484 | 3914.547471 |
| 5737 | INTS2 | Q9H0H0 | 3910.225918 |
| 5738 | NOA1 | Q8NC60 | 3904.479829 |
| 5739 | TAF6 | P49848 | 3892.72641 |
| 5740 | FLCN | Q8NFG4 | 3890.566742 |
| 5741 | KANK4 | Q5T7N3 | 3884.744444 |
| 5742 | MARCHF3 | Q86UD3 | 3879.356308 |
| 5743 | MRTFA | Q969V6 | 3878.458406 |
| 5744 | MRPL51 | Q4U2R6 | 3871.442 |
| 5745 | TXNRD2 | Q9NNW7 | 3871.06756 |
| 5746 | TNFRSF10A | O00220 | 3866.1042 |
| 5747 | TNRC6B | Q9UPQ9 | 3859.308422 |
| 5748 | RPGR | Q92834 | 3855.063159 |
| 5749 | SDC4 | P31431 | 3851.933333 |
| 5750 | NAA40 | Q86UY6 | 3834.77 |
| 5751 | ITGB4 | P16144 | 3825.239245 |
| 5752 | MOCS2 | O96007 | 3824.675636 |
| 5753 | SAMD4B | Q5PRF9 | 3817.765254 |
| 5754 | GDE1 | Q9NZC3 | 3816.440389 |
| 5755 | C6orf47 | O95873 | 3812.943083 |
| 5756 | C1orf131 | Q8NDD1 | 3802.873385 |
| 5757 | YJU2 | Q9BW85 | 3785.719267 |
| 5758 | GIMAP6 | Q6P9H5 | 3783.746462 |
| 5759 | COBLL1 | Q53SF7 | 3779.130067 |
| 5760 | CRYBG3 | Q68DQ2 | 3771.703046 |
| 5761 | KIF20A | O95235 | 3768.908496 |
| 5762 | COX7A2L | O14548 | 3768.495333 |
| 5763 | NR2F1 | P10589 | 3768.441729 |
| 5764 | TNFAIP8 | O95379 | 3766.3451 |
| 5765 | THAP11 | Q96EK4 | 3764.99425 |
| 5766 | GMIP | Q9P107 | 3764.155343 |
| 5767 | PHF2 | O75151 | 3754.426021 |
| 5768 | DHRS11 | Q6UWP2 | 3745.916647 |
| 5769 | CTDSPL2 | Q05D32 | 3744.8973 |
| 5770 | TTI2 | Q6NXR4 | 3743.877043 |
| 5771 | ART4 | Q93070 | 3741.263895 |
| 5772 | F13A1 | P00488 | 3740.841771 |
| 5773 | UQCRQ | O14949 | 3737.748333 |
| 5774 | CHD1 | O14646 | 3734.460621 |
| 5775 | TEFM | Q96QE5 | 3727.828227 |
| 5776 | ARL5B | Q96KC2 | 3726.837 |
| 5777 | BMPR2 | Q13873 | 3724.536214 |
| 5778 | RING1 | Q06587 | 3723.449429 |
| 5779 | PRAG1 | Q86YV5 | 3711.564308 |
| 5780 | PLCB1 | Q9NQ66 | 3710.52994 |
| 5781 | EMSY | Q7Z589 | 3709.347582 |
| 5782 | LTBP1 | Q14766 | 3700.845273 |
| 5783 | UBR3 | Q6ZT12 | 3698.679528 |
| 5784 | EHBP1 | Q8NDI1 | 3697.044758 |
| 5785 | ABHD13 | Q7L211 | 3692.608444 |
| 5786 | NR4A3 | Q92570 | 3681.2971 |
| 5787 | MRPL30 | Q8TCC3 | 3668.744222 |
| 5788 | PRND | Q9UKY0 | 3668.609778 |
| 5789 | SLC1A1 | P43005 | 3657.031368 |
| 5790 | HEG1 | Q9ULI3 | 3655.194135 |
| 5791 | GLIPR1 | P48060 | 3651.950909 |
| 5792 | IGF1R | P08069 | 3650.947957 |
| 5793 | NFKBIB | Q15653 | 3649.130875 |
| 5794 | VHL | P40337 | 3646.3034 |
| 5795 | SCAF8 | Q9UPN6 | 3645.99422 |
| 5796 | ZEB2 | O60315 | 3645.575808 |
| 5797 | EHMT2 | Q96KQ7 | 3641.920375 |
| 5798 | WDR7 | Q9Y4E6 | 3637.674029 |
| 5799 | ERICH1 | Q86X53 | 3620.2975 |
| 5800 | HMCN1 | Q96RW7 | 3618.779327 |
| 5801 | CHST3 | Q7LGC8 | 3618.760909 |
| 5802 | FRA10AC1 | Q70Z53 | 3617.368294 |
| 5803 | PHKA1 | P46020 | 3615.267403 |
| 5804 | POTEF | A5A3E0 | 3615.217132 |
| 5805 | SNX29 | Q8TEQ0 | 3615.211357 |
| 5806 | MTFR1L | Q9H019 | 3614.753846 |
| 5807 | FKRP | Q9H9S5 | 3614.392958 |
| 5808 | SNX16 | P57768 | 3612.261941 |
| 5809 | PHAX | Q9H814 | 3610.3489 |
| 5810 | KDM5B | Q9UGL1 | 3609.538 |
| 5811 | CCDC59 | Q9P031 | 3603.287111 |
| 5812 | IL17RA | Q96F46 | 3601.941148 |
| 5813 | SORT1 | Q99523 | 3600.471412 |
| 5814 | DIXDC1 | Q155Q3 | 3598.279784 |
| 5815 | MED14 | O60244 | 3597.033491 |
| 5816 | NCALD | P61601 | 3590.736154 |
| 5817 | OGFOD1 | Q8N543 | 3588.88888 |
| 5818 | SMURF1 | Q9HCE7 | 3585.920488 |
| 5819 | TMLHE | Q9NVH6 | 3582.795148 |
| 5820 | ARHGEF11 | O15085 | 3577.276648 |
| 5821 | ARHGEF37 | A1IGU5 | 3576.310865 |
| 5822 | RIOK1 | Q9BRS2 | 3568.016385 |
| 5823 | KPTN | Q9Y664 | 3559.263857 |
| 5824 | NDUFAF1 | Q9Y375 | 3557.961611 |
| 5825 | GCFC2 | P16383 | 3557.290556 |
| 5826 | MIOS | Q9NXC5 | 3552.407415 |
| 5827 | ZC3H4 | Q9UPT8 | 3550.543014 |
| 5828 | MMADHC | Q9H3L0 | 3549.7768 |
| 5829 | PHF10 | Q8WUB8 | 3549.504773 |
| 5830 | KRT77 | Q7Z794 | 3545.239935 |
| 5831 | PREPL | Q4J6C6 | 3542.072263 |
| 5832 | ORC4 | O43929 | 3539.09396 |
| 5833 | PPP1R16B | Q96T49 | 3537.08716 |
| 5834 | METTL14 | Q9HCE5 | 3532.989938 |
| 5835 | SNX30 | Q5VWJ9 | 3527.477583 |
| 5836 | EVI5L | Q96CN4 | 3525.032436 |
| 5837 | PAAT | Q9H8K7 | 3523.9115 |
| 5838 | LIN9 | Q5TKA1 | 3521.35516 |
| 5839 | STARD7 | Q9NQZ5 | 3518.965435 |
| 5840 | PLBD1 | Q6P4A8 | 3516.727391 |
| 5841 | CENPC | Q03188 | 3511.385667 |
| 5842 | AEBP1 | Q8IUX7 | 3509.00914 |
| 5843 | KIFC1 | Q9BW19 | 3506.845268 |
| 5844 | GTSE1 | Q9NYZ3 | 3506.443086 |
| 5845 | HFE | Q30201 | 3504.542667 |
| 5846 | TMEM245 | Q9H330 | 3500.47904 |
| 5847 | PTGS1 | P23219 | 3497.794034 |
| 5848 | IGFBP3 | P17936 | 3490.333944 |
| 5849 | SP1 | P08047 | 3483.0931 |
| 5850 | TMEM41A | Q96HV5 | 3481.690444 |
| 5851 | POLB | P06746 | 3481.648389 |
| 5852 | FADS1 | O60427 | 3480.891591 |
| 5853 | CERS1 | P27544 | 3480.5827 |
| 5854 | PTPRJ | Q12913 | 3478.778772 |
| 5855 | ICMT | O60725 | 3469.5969 |
| 5856 | MAGI1 | Q96QZ7 | 3468.577221 |
| 5857 | RALGPS2 | Q86X27 | 3462.603371 |
| 5858 | CEP131 | Q9UPN4 | 3449.602703 |
| 5859 | EHMT1 | Q9H9B1 | 3439.966424 |
| 5860 | TPST2 | O60704 | 3436.609063 |
| 5861 | EPS8L2 | Q9H6S3 | 3435.7675 |
| 5862 | EVI2B | P34910 | 3433.886875 |
| 5863 | ALG12 | Q9BV10 | 3433.653333 |
| 5864 | RNF185 | Q96GF1 | 3427.604833 |
| 5865 | ANKRD28 | O15084 | 3427.301628 |
| 5866 | TOR1AIP2 | Q9H496 | 3425.35075 |
| 5867 | ANGPTL2 | Q9UKU9 | 3416.945839 |
| 5868 | PIP5K1A | Q99755 | 3409.317308 |
| 5869 | D2HGDH | Q8N465 | 3404.194087 |
| 5870 | BAP1 | Q92560 | 3393.553818 |
| 5871 | FRMD6 | Q96NE9 | 3391.915848 |
| 5872 | KIAA2013 | Q8IYS2 | 3389.705609 |
| 5873 | LCAT | P04180 | 3385.803688 |
| 5874 | XYLT2 | Q9H1B5 | 3369.239813 |
| 5875 | ATG2B | Q96BY7 | 3367.567096 |
| 5876 | STIM2 | Q9P246 | 3363.075774 |
| 5877 | MRPL32 | Q9BYC8 | 3360.066571 |
| 5878 | ZNF22 | P17026 | 3359.480583 |
| 5879 | DNM3 | Q9UQ16 | 3352.395102 |
| 5880 | AP5B1 | Q2VPB7 | 3351.821057 |
| 5881 | NSMCE1 | Q8WV22 | 3336.816333 |
| 5882 | GPS2 | Q13227 | 3321.777 |
| 5883 | FOSL1 | P15407 | 3317.205429 |
| 5884 | CAMSAP1 | Q5T5Y3 | 3313.011909 |
| 5885 | C1orf43 | Q9BWL3 | 3302.404615 |
| 5886 | GPN2 | Q9H9Y4 | 3300.4156 |
| 5887 | TOR3A | Q9H497 | 3298.888 |
| 5888 | CIT | O14578 | 3289.623168 |
| 5889 | NDST1 | P52848 | 3283.99125 |
| 5890 | FAT4 | Q6V0I7 | 3271.428366 |
| 5891 | BAZ2A | Q9UIF9 | 3266.015439 |
| 5892 | COMP | P49747 | 3265.385667 |
| 5893 | WDSUB1 | Q8N9V3 | 3252.871136 |
| 5894 | SRGAP1 | Q7Z6B7 | 3246.770509 |
| 5895 | TXNRD3 | Q86VQ6 | 3246.419758 |
| 5896 | INO80C | Q6PI98 | 3244.736727 |
| 5897 | TMEM47 | Q9BQJ4 | 3244.71 |
| 5898 | TRIAP1 | O43715 | 3230.9835 |
| 5899 | MEIS3P1 | A6NDR6 | 3229.916667 |
| 5900 | ADGRE5 | P48960 | 3229.693939 |
| 5901 | ZMYM2 | Q9UBW7 | 3229.48006 |
| 5902 | MEF2D | Q14814 | 3228.420067 |
| 5903 | FARS2 | O95363 | 3228.055227 |
| 5904 | TMEM168 | Q9H0V1 | 3227.4955 |
| 5905 | SPG11 | Q96JI7 | 3224.744929 |
| 5906 | USP40 | Q9NVE5 | 3224.385944 |
| 5907 | IRF2BP1 | Q8IU81 | 3223.782571 |
| 5908 | ALDH8A1 | Q9H2A2 | 3219.684696 |
| 5909 | CSNK1G1 | Q9HCP0 | 3214.136667 |
| 5910 | SLC38A1 | Q9H2H9 | 3199.688333 |
| 5911 | NIPBL | Q6KC79 | 3197.786282 |
| 5912 | TMEM147 | Q9BVK8 | 3194.4575 |
| 5913 | GNG11 | P61952 | 3193.222333 |
| 5914 | SMC5 | Q8IY18 | 3192.762643 |
| 5915 | ABCA3 | Q99758 | 3191.528696 |
| 5916 | KAT7 | O95251 | 3190.734655 |
| 5917 | BICD1 | Q96G01 | 3183.344148 |
| 5918 | IRF9 | Q00978 | 3179.54525 |
| 5919 | METTL21A | Q8WXB1 | 3176.338556 |
| 5920 | PSTPIP2 | Q9H939 | 3168.2343 |
| 5921 | SAMD9L | Q8IVG5 | 3163.734244 |
| 5922 | CA8 | P35219 | 3161.537818 |
| 5923 | DDX28 | Q9NUL7 | 3160.407441 |
| 5924 | CIC | Q96RK0 | 3157.556051 |
| 5925 | DHX35 | Q9H5Z1 | 3154.305184 |
| 5926 | MLIP | Q5VWP3 | 3149.136326 |
| 5927 | CRYAB | P02511 | 3147.680545 |
| 5928 | DHDDS | Q86SQ9 | 3147.1398 |
| 5929 | PHRF1 | Q9P1Y6 | 3142.728082 |
| 5930 | BAIAP2 | Q9UQB8 | 3139.280343 |
| 5931 | LDLR | P01130 | 3136.383435 |
| 5932 | ACCS | Q96QU6 | 3127.422857 |
| 5933 | STK11IP | Q8N1F8 | 3125.305933 |
| 5934 | FGG | P02679 | 3121.925 |
| 5935 | OBI1 | Q5W0B1 | 3119.733725 |
| 5936 | TASOR | Q9UK61 | 3118.823939 |
| 5937 | RNF123 | Q5XPI4 | 3117.838071 |
| 5938 | KIF22 | Q14807 | 3105.114833 |
| 5939 | TIMM29 | Q9BSF4 | 3098.163692 |
| 5940 | FAM210A | Q96ND0 | 3096.926357 |
| 5941 | BUD13 | Q9BRD0 | 3094.724657 |
| 5942 | CCDC85A | Q96PX6 | 3094.701571 |
| 5943 | DENND11 | A4D1U4 | 3093.88528 |
| 5944 | TNS3 | Q68CZ2 | 3090.38137 |
| 5945 | GASK1B | Q6UWH4 | 3088.512 |
| 5946 | DPH6 | Q7L8W6 | 3081.958462 |
| 5947 | DAXX | Q9UER7 | 3079.718545 |
| 5948 | TMEM131 | Q92545 | 3079.62475 |
| 5949 | CCBE1 | Q6UXH8 | 3076.892632 |
| 5950 | FAM53C | Q9NYF3 | 3075.479938 |
| 5951 | TBC1D8 | O95759 | 3072.901509 |
| 5952 | AMZ2 | Q86W34 | 3066.853789 |
| 5953 | GALC | P54803 | 3061.726656 |
| 5954 | KSR1 | Q8IVT5 | 3061.498528 |
| 5955 | ADARB1 | P78563 | 3053.706933 |
| 5956 | COL12A1 | Q99715 | 3051.469374 |
| 5957 | PBX2 | P40425 | 3048.3949 |
| 5958 | KIAA1549L | Q6ZVL6 | 3043.749656 |
| 5959 | ERBB2 | P04626 | 3035.859184 |
| 5960 | FBXO9 | Q9UK97 | 3028.843731 |
| 5961 | COX19 | Q49B96 | 3020.005333 |
| 5962 | CLN8 | Q9UBY8 | 3014.189889 |
| 5963 | PCNT | O95613 | 3013.369937 |
| 5964 | TACC3 | Q9Y6A5 | 3011.549829 |
| 5965 | ULK3 | Q6PHR2 | 3005.964125 |
| 5966 | ZNHIT2 | Q9UHR6 | 3001.51555 |
| 5967 | CPTP | Q5TA50 | 2991.857071 |
| 5968 | CHFR | Q96EP1 | 2989.702719 |
| 5969 | VAMP4 | O75379 | 2988.410283 |
| 5970 | TEP1 | Q99973 | 2984.113548 |
| 5971 | IDNK | Q5T6J7 | 2980.657545 |
| 5972 | HAUS2 | Q9NVX0 | 2969.783 |
| 5973 | B4GALT5 | O43286 | 2969.779118 |
| 5974 | SCAF11 | Q99590 | 2968.979667 |
| 5975 | SCARF1 | Q14162 | 2967.504914 |
| 5976 | CTSS | P25774 | 2963.8902 |
| 5977 | RBMS1 | P29558 | 2959.067636 |
| 5978 | CSRP2 | Q16527 | 2958.711818 |
| 5979 | RANBP17 | Q9H2T7 | 2952.085636 |
| 5980 | GPSM3 | Q9Y4H4 | 2949.27425 |
| 5981 | PSMB10 | P40306 | 2945.756364 |
| 5982 | PDGFC | Q9NRA1 | 2940.747889 |
| 5983 | SH3BP5L | Q7L8J4 | 2936.539818 |
| 5984 | MANEA | Q5SRI9 | 2924.72645 |
| 5985 | AMBRA1 | Q9C0C7 | 2919.6973 |
| 5986 | SIKE1 | Q9BRV8 | 2919.171286 |
| 5987 | PANK2 | Q9BZ23 | 2911.96213 |
| 5988 | TBC1D9 | Q6ZT07 | 2905.287197 |
| 5989 | FBXL20 | Q96IG2 | 2901.56464 |
| 5990 | F2R | P25116 | 2897.368538 |
| 5991 | NLRP1 | Q9C000 | 2890.623459 |
| 5992 | LIN54 | Q6MZP7 | 2890.27303 |
| 5993 | RECK | O95980 | 2889.332711 |
| 5994 | WWP2 | O00308 | 2886.597 |
| 5995 | GLUL | P15104 | 2884.311 |
| 5996 | NCOA3 | Q9Y6Q9 | 2884.122821 |
| 5997 | DESI2 | Q9BSY9 | 2883.655833 |
| 5998 | PPCDC | Q96CD2 | 2882.992333 |
| 5999 | AMN1 | Q8IY45 | 2882.463857 |
| 6000 | RERE | Q9P2R6 | 2868.828981 |
| 6001 | FEZ1 | Q99689 | 2865.2915 |
| 6002 | CPS1 | P31327 | 2859.007158 |
| 6003 | ACTR8 | Q9H981 | 2858.656273 |
| 6004 | CCDC186 | Q7Z3E2 | 2851.422667 |
| 6005 | CALCOCO2 | Q13137 | 2844.2545 |
| 6006 | AURKAIP1 | Q9NWT8 | 2838.076 |
| 6007 | PJA2 | O43164 | 2837.343212 |
| 6008 | ALG6 | Q9Y672 | 2831.517857 |
| 6009 | DROSHA | Q9NRR4 | 2830.495019 |
| 6010 | PRKD1 | Q15139 | 2829.735548 |
| 6011 | ITPR1 | Q14643 | 2829.029124 |
| 6012 | UBR2 | Q8IWV8 | 2828.931621 |
| 6013 | CLCN3 | P51790 | 2822.900758 |
| 6014 | UBN1 | Q9NPG3 | 2820.805725 |
| 6015 | SECISBP2L | Q93073 | 2819.010902 |
| 6016 | PHTF1 | Q9UMS5 | 2815.515371 |
| 6017 | S100A8 | P05109 | 2815.290875 |
| 6018 | ANKRD54 | Q6NXT1 | 2814.975185 |
| 6019 | KATNBL1 | Q9H079 | 2814.615426 |
| 6020 | PRUNE1 | Q86TP1 | 2806.734455 |
| 6021 | MAU2 | Q9Y6X3 | 2801.296429 |
| 6022 | GPAT3 | Q53EU6 | 2800.721043 |
| 6023 | PRR14 | Q9BWN1 | 2794.713333 |
| 6024 | CSN2 | P02666 | 2792.994286 |
| 6025 | CCDC92 | Q53HC0 | 2790.8369 |
| 6026 | BBS7 | Q8IWZ6 | 2789.694559 |
| 6027 | DDX55 | Q8NHQ9 | 2786.580182 |
| 6028 | PICK1 | Q9NRD5 | 2785.3526 |
| 6029 | SHB | Q15464 | 2782.74916 |
| 6030 | C12orf29 | Q8N999 | 2781.514 |
| 6031 | FBP1 | P09467 | 2781.459 |
| 6032 | CASP8 | Q14790 | 2778.50548 |
| 6033 | NOCT | Q9UK39 | 2777.64916 |
| 6034 | KIF15 | Q9NS87 | 2776.753181 |
| 6035 | SLC2A3 | P11169 | 2772.610583 |
| 6036 | UBAC1 | Q9BSL1 | 2771.463609 |
| 6037 | WDR89 | Q96FK6 | 2771.280444 |
| 6038 | MAN1A1 | P33908 | 2766.347419 |
| 6039 | FARP1 | Q9Y4F1 | 2764.829529 |
| 6040 | OAS1 | P00973 | 2764.5225 |
| 6041 | DNAL1 | Q4LDG9 | 2759.06225 |
| 6042 | SLC25A33 | Q9BSK2 | 2757.672667 |
| 6043 | UIMC1 | Q96RL1 | 2756.605626 |
| 6044 | AFF4 | Q9UHB7 | 2755.866948 |
| 6045 | PMM1 | Q92871 | 2744.7446 |
| 6046 | BCKDHB | P21953 | 2744.556529 |
| 6047 | SPIRE1 | Q08AE8 | 2740.578595 |
| 6048 | CSPP1 | Q1MSJ5 | 2736.973965 |
| 6049 | SIDT2 | Q8NBJ9 | 2733.423964 |
| 6050 | ASB8 | Q9H765 | 2730.700526 |
| 6051 | WWC3 | Q9ULE0 | 2730.314816 |
| 6052 | DCAF16 | Q9NXF7 | 2722.76375 |
| 6053 | WDR20 | Q8TBZ3 | 2721.265667 |
| 6054 | RNGTT | O60942 | 2712.112781 |
| 6055 | P2RX4 | Q99571 | 2711.656895 |
| 6056 | UBE3B | Q7Z3V4 | 2711.053745 |
| 6057 | HLA-G | P17693 | 2709.2705 |
| 6058 | PCMTD2 | Q9NV79 | 2707.470556 |
| 6059 | ZFAND5 | O76080 | 2702.039364 |
| 6060 | FAM171A2 | A8MVW0 | 2700.504 |
| 6061 | FHIP2B | Q86V87 | 2698.962294 |
| 6062 | ATR | Q13535 | 2698.856607 |
| 6063 | MELTF | P08582 | 2697.364171 |
| 6064 | CEBPD | P49716 | 2696.466286 |
| 6065 | RNF146 | Q9NTX7 | 2696.166429 |
| 6066 | ABL2 | P42684 | 2694.800368 |
| 6067 | RASSF3 | Q86WH2 | 2694.774286 |
| 6068 | ZYG11B | Q9C0D3 | 2692.118727 |
| 6069 | PPHLN1 | Q8NEY8 | 2688.809208 |
| 6070 | PTPN2 | P17706 | 2685.260077 |
| 6071 | SHROOM3 | Q8TF72 | 2683.739223 |
| 6072 | GALT | P07902 | 2679.009813 |
| 6073 | PHC3 | Q8NDX5 | 2678.650458 |
| 6074 | SIPA1L1 | O43166 | 2676.260326 |
| 6075 | RAPGEF6 | Q8TEU7 | 2672.292646 |
| 6076 | DAGLB | Q8NCG7 | 2665.819688 |
| 6077 | PLEKHG5 | O94827 | 2654.963522 |
| 6078 | EXT1 | Q16394 | 2644.790528 |
| 6079 | ABITRAM | Q9NX38 | 2643.073455 |
| 6080 | DGUOK | Q16854 | 2643.064615 |
| 6081 | TRPC4AP | Q8TEL6 | 2642.137886 |
| 6082 | KRI1 | Q8N9T8 | 2641.247962 |
| 6083 | MKNK1 | Q9BUB5 | 2639.360789 |
| 6084 | CCNL2 | Q96S94 | 2630.85181 |
| 6085 | AFTPH | Q6ULP2 | 2625.379263 |
| 6086 | SMIM4 | Q8WVI0 | 2623.764 |
| 6087 | TTI1 | O43156 | 2621.277939 |
| 6088 | SIRT3 | Q9NTG7 | 2619.754353 |
| 6089 | TLE1 | Q04724 | 2619.483322 |
| 6090 | PAN3 | Q58A45 | 2611.41036 |
| 6091 | NANP | Q8TBE9 | 2607.230154 |
| 6092 | SFXN2 | Q96NB2 | 2605.426882 |
| 6093 | RGL1 | Q9NZL6 | 2602.714882 |
| 6094 | CRTC2 | Q53ET0 | 2593.7912 |
| 6095 | ATXN1L | P0C7T5 | 2592.463483 |
| 6096 | METTL2B | Q6P1Q9 | 2590.131714 |
| 6097 | RUNX1 | Q01196 | 2585.499 |
| 6098 | CARNMT1 | Q8N4J0 | 2585.290211 |
| 6099 | YY1 | P25490 | 2582.164313 |
| 6100 | ACTR5 | Q9H9F9 | 2579.503 |
| 6101 | VRK3 | Q8IV63 | 2576.54052 |
| 6102 | LRRC17 | Q8N6Y2 | 2572.537636 |
| 6103 | GALNT15 | Q8N3T1 | 2572.160036 |
| 6104 | PHF8 | Q9UPP1 | 2569.139976 |
| 6105 | SNX24 | Q9Y343 | 2568.620909 |
| 6106 | TDRP | Q86YL5 | 2567.726222 |
| 6107 | ANKS1A | Q92625 | 2563.039019 |
| 6108 | JOSD2 | Q8TAC2 | 2562.0825 |
| 6109 | DNAJC25 | Q9H1X3 | 2560.400625 |
| 6110 | WDR55 | Q9H6Y2 | 2558.600476 |
| 6111 | HERC2 | O95714 | 2555.892838 |
| 6112 | RMND5A | Q9H871 | 2553.633261 |
| 6113 | PPP2CA | P67775 | 2551.36375 |
| 6114 | PCIF1 | Q9H4Z3 | 2541.256182 |
| 6115 | DGKZ | Q13574 | 2540.151405 |
| 6116 | IFRD2 | Q12894 | 2537.275944 |
| 6117 | SIPA1L3 | O60292 | 2535.699781 |
| 6118 | ARID5B | Q14865 | 2532.208794 |
| 6119 | NSRP1 | Q9H0G5 | 2531.913348 |
| 6120 | LMO4 | P61968 | 2528.700125 |
| 6121 | XPO6 | Q96QU8 | 2526.94628 |
| 6122 | KRT17 | Q04695 | 2526.215069 |
| 6123 | CCDC9B | Q6ZUT6 | 2525.455077 |
| 6124 | PPP2CB | P62714 | 2524.94475 |
| 6125 | CRACR2A | Q9BSW2 | 2524.873128 |
| 6126 | GNB3 | P16520 | 2521.176875 |
| 6127 | SLC35G2 | Q8TBE7 | 2520.662667 |
| 6128 | PLCL1 | Q15111 | 2518.563444 |
| 6129 | SYNM | O15061 | 2517.024407 |
| 6130 | SLC6A6 | P31641 | 2516.875111 |
| 6131 | ST6GALNAC3 | Q8NDV1 | 2516.541765 |
| 6132 | MSANTD2 | Q6P1R3 | 2514.059261 |
| 6133 | APBA2 | Q99767 | 2512.84932 |
| 6134 | MON1B | Q7L1V2 | 2503.338938 |
| 6135 | POMK | Q9H5K3 | 2503.083625 |
| 6136 | ALG3 | Q92685 | 2502.895118 |
| 6137 | SP2 | Q02086 | 2501.659714 |
| 6138 | ASB3 | Q9Y575 | 2499.028333 |
| 6139 | METTL2A | Q96IZ6 | 2498.48975 |
| 6140 | ELL | P55199 | 2494.990286 |
| 6141 | KIAA0754 | O94854 | 2486.237778 |
| 6142 | GFOD2 | Q3B7J2 | 2484.656438 |
| 6143 | CROCC | Q5TZA2 | 2483.563522 |
| 6144 | CUX1 | P39880 | 2483.374361 |
| 6145 | TYK2 | P29597 | 2482.563547 |
| 6146 | NUAK1 | O60285 | 2482.507303 |
| 6147 | CDK16 | Q00536 | 2482.097893 |
| 6148 | PAFAH2 | Q99487 | 2480.03532 |
| 6149 | DSP | P15924 | 2471.754596 |
| 6150 | ACTR6 | Q9GZN1 | 2469.768944 |
| 6151 | CLCN5 | P51795 | 2465.18 |
| 6152 | ZNF609 | O15014 | 2464.87225 |
| 6153 | TRPC4 | Q9UBN4 | 2463.869821 |
| 6154 | LMF1 | Q96S06 | 2459.554857 |
| 6155 | HERC1 | Q15751 | 2450.108812 |
| 6156 | WFIKKN2 | Q8TEU8 | 2443.340524 |
| 6157 | SPTA1 | P02549 | 2435.647164 |
| 6158 | ITPKB | P27987 | 2434.943259 |
| 6159 | CYB5D2 | Q8WUJ1 | 2432.711615 |
| 6160 | MICALL2 | Q8IY33 | 2431.85722 |
| 6161 | KLHL18 | O94889 | 2426.858818 |
| 6162 | SETX | Q7Z333 | 2420.937849 |
| 6163 | PPP1R3B | Q86XI6 | 2418.677 |
| 6164 | ZDHHC6 | Q9H6R6 | 2405.260133 |
| 6165 | SYN1 | P17600 | 2400.229032 |
| 6166 | TANGO6 | Q9C0B7 | 2399.997069 |
| 6167 | SMARCAD1 | Q9H4L7 | 2397.926939 |
| 6168 | RNF220 | Q5VTB9 | 2391.727238 |
| 6169 | EIF2AK3 | Q9NZJ5 | 2380.417949 |
| 6170 | SMG1 | Q96Q15 | 2376.75062 |
| 6171 | MON1A | Q86VX9 | 2371.717711 |
| 6172 | ELP4 | Q96EB1 | 2370.911458 |
| 6173 | ATP11A | P98196 | 2370.719788 |
| 6174 | LZTR1 | Q8N653 | 2370.362958 |
| 6175 | SGMS1 | Q86VZ5 | 2369.13015 |
| 6176 | SLC2A6 | Q9UGQ3 | 2360.282824 |
| 6177 | KLHDC4 | Q8TBB5 | 2358.933304 |
| 6178 | TTF2 | Q9UNY4 | 2357.284185 |
| 6179 | SPPL2B | Q8TCT7 | 2340.840069 |
| 6180 | UHRF1 | Q96T88 | 2340.79941 |
| 6181 | GIGYF1 | O75420 | 2335.182404 |
| 6182 | ORC2 | Q13416 | 2333.911839 |
| 6183 | KRT8 | P05787 | 2332.855966 |
| 6184 | USP16 | Q9Y5T5 | 2330.453721 |
| 6185 | DZIP3 | Q86Y13 | 2328.585072 |
| 6186 | CYP2S1 | Q96SQ9 | 2324.673864 |
| 6187 | ITIH1 | P19827 | 2323.488917 |
| 6188 | FAM43A | Q8N2R8 | 2322.28281 |
| 6189 | PRIM1 | P49642 | 2322.021885 |
| 6190 | SESN2 | P58004 | 2320.961542 |
| 6191 | ALG13 | Q9NP73 | 2320.094733 |
| 6192 | ARHGEF40 | Q8TER5 | 2319.431812 |
| 6193 | GRK5 | P34947 | 2306.759414 |
| 6194 | PRORP | O15091 | 2302.265809 |
| 6195 | COL4A1 | P02462 | 2302.179949 |
| 6196 | NGLY1 | Q96IV0 | 2300.571694 |
| 6197 | KLHL20 | Q9Y2M5 | 2299.847676 |
| 6198 | KLHL12 | Q53G59 | 2299.073414 |
| 6199 | ADM | P35318 | 2297.53 |
| 6200 | INTS3 | Q68E01 | 2296.848042 |
| 6201 | PRPF18 | Q99633 | 2288.963211 |
| 6202 | B3GALNT1 | O75752 | 2287.836 |
| 6203 | TECPR2 | O15040 | 2285.62993 |
| 6204 | SLC25A16 | P16260 | 2281.728105 |
| 6205 | EXTL3 | O43909 | 2279.07064 |
| 6206 | CARD6 | Q9BX69 | 2276.807855 |
| 6207 | C7orf26 | Q96N11 | 2273.358778 |
| 6208 | TRPT1 | Q86TN4 | 2272.114933 |
| 6209 | ADGB | Q8N7X0 | 2269.85427 |
| 6210 | PUS3 | Q9BZE2 | 2269.388966 |
| 6211 | SLC26A6 | Q9BXS9 | 2266.866107 |
| 6212 | N4BP1 | O75113 | 2261.091392 |
| 6213 | FBXO30 | Q8TB52 | 2260.637063 |
| 6214 | TUBGCP6 | Q96RT7 | 2260.544649 |
| 6215 | ZNF462 | Q96JM2 | 2255.5145 |
| 6216 | TNK2 | Q07912 | 2254.176591 |
| 6217 | CCDC85B | Q15834 | 2248.722455 |
| 6218 | IL18R1 | Q13478 | 2248.178607 |
| 6219 | DERPC | P0CG12 | 2247.996742 |
| 6220 | TMCC1 | O94876 | 2247.073206 |
| 6221 | RWDD2B | P57060 | 2245.107563 |
| 6222 | DMAC1 | Q96GE9 | 2243.6535 |
| 6223 | ZER1 | Q7Z7L7 | 2239.524541 |
| 6224 | PIKFYVE | Q9Y2I7 | 2233.392336 |
| 6225 | ADAMTS4 | O75173 | 2231.4425 |
| 6226 | ZMYND11 | Q15326 | 2230.692172 |
| 6227 | MPV17 | P39210 | 2229.58075 |
| 6228 | FOXJ2 | Q9P0K8 | 2224.446533 |
| 6229 | CREBBP | Q92793 | 2221.264405 |
| 6230 | TMEM242 | Q9NWH2 | 2219.116875 |
| 6231 | NT5DC3 | Q86UY8 | 2216.134143 |
| 6232 | NFATC1 | O95644 | 2214.936211 |
| 6233 | SMAD5 | Q99717 | 2212.687818 |
| 6234 | TNFAIP2 | Q03169 | 2212.368333 |
| 6235 | MGAT4A | Q9UM21 | 2212.093 |
| 6236 | PMS1 | P54277 | 2205.033037 |
| 6237 | SGK3 | Q96BR1 | 2198.72955 |
| 6238 | NBEAL2 | Q6ZNJ1 | 2191.298811 |
| 6239 | TRMU | O75648 | 2190.428519 |
| 6240 | PKN3 | Q6P5Z2 | 2184.680659 |
| 6241 | THNSL1 | Q8IYQ7 | 2184.046528 |
| 6242 | PTGR2 | Q8N8N7 | 2181.418611 |
| 6243 | C19orf54 | Q5BKX5 | 2176.032636 |
| 6244 | MALT1 | Q9UDY8 | 2175.939595 |
| 6245 | CLK3 | P49761 | 2175.767111 |
| 6246 | NR1I3 | Q14994 | 2175.591875 |
| 6247 | PACS2 | Q86VP3 | 2170.142853 |
| 6248 | KDM4A | O75164 | 2168.597304 |
| 6249 | HMMR | O75330 | 2168.143341 |
| 6250 | POMGNT1 | Q8WZA1 | 2166.887273 |
| 6251 | ATG12 | O94817 | 2166.33575 |
| 6252 | GINS2 | Q9Y248 | 2166.092 |
| 6253 | TRIM59 | Q8IWR1 | 2163.838706 |
| 6254 | CASTOR1 | Q8WTX7 | 2161.789 |
| 6255 | GTPBP8 | Q8N3Z3 | 2161.498824 |
| 6256 | SKI | P12755 | 2153.686 |
| 6257 | THADA | Q6YHU6 | 2153.398806 |
| 6258 | PJA1 | Q8NG27 | 2152.236074 |
| 6259 | RAVER2 | Q9HCJ3 | 2151.69208 |
| 6260 | CMSS1 | Q9BQ75 | 2151.494545 |
| 6261 | ANKRD40 | Q6AI12 | 2147.5114 |
| 6262 | TRIM24 | O15164 | 2139.630571 |
| 6263 | MRM1 | Q6IN84 | 2138.899706 |
| 6264 | TSPAN15 | O95858 | 2135.39725 |
| 6265 | MKLN1 | Q9UL63 | 2134.373773 |
| 6266 | THAP1 | Q9NVV9 | 2134.216 |
| 6267 | CHEK2 | O96017 | 2132.11068 |
| 6268 | PAM | P19021 | 2130.367667 |
| 6269 | MEX3C | Q5U5Q3 | 2129.523144 |
| 6270 | RAP2A | P10114 | 2126.450545 |
| 6271 | PANX1 | Q96RD7 | 2125.923516 |
| 6272 | HLTF | Q14527 | 2125.896804 |
| 6273 | SPAG1 | Q07617 | 2124.853472 |
| 6274 | CEP55 | Q53EZ4 | 2123.568385 |
| 6275 | KIF23 | Q02241 | 2121.058237 |
| 6276 | ATRIP | Q8WXE1 | 2116.001719 |
| 6277 | MAP3K4 | Q9Y6R4 | 2113.758011 |
| 6278 | PITPNM2 | Q9BZ72 | 2111.769063 |
| 6279 | SERPINB12 | Q96P63 | 2111.628571 |
| 6280 | RAB2B | Q8WUD1 | 2110.5516 |
| 6281 | MECOM | Q03112 | 2107.40869 |
| 6282 | FAM214A | Q32MH5 | 2098.406078 |
| 6283 | AMD1 | P17707 | 2095.108211 |
| 6284 | PI4K2B | Q8TCG2 | 2087.931935 |
| 6285 | DENND6A | Q8IWF6 | 2073.592724 |
| 6286 | RPS6KA1 | Q15418 | 2071.321628 |
| 6287 | FAM3A | P98173 | 2066.9775 |
| 6288 | ITGB1BP1 | O14713 | 2065.900417 |
| 6289 | XYLB | O75191 | 2065.280462 |
| 6290 | OSBPL7 | Q9BZF2 | 2062.238022 |
| 6291 | SPEN | Q96T58 | 2060.352631 |
| 6292 | LY75 | O60449 | 2059.732976 |
| 6293 | PPIF | P30405 | 2059.459091 |
| 6294 | FGGY | Q96C11 | 2055.233586 |
| 6295 | RGPD3 | A6NKT7 | 2051.014945 |
| 6296 | PHLDA1 | Q8WV24 | 2043.787059 |
| 6297 | FAM166B | A8MTA8 | 2038.290667 |
| 6298 | ANKRD46 | Q86W74 | 2035.5048 |
| 6299 | TANGO2 | Q6ICL3 | 2034.364353 |
| 6300 | EPHA4 | P54764 | 2033.64574 |
| 6301 | FBXW9 | Q5XUX1 | 2028.804077 |
| 6302 | SMAD6 | O43541 | 2028.7565 |
| 6303 | KRT6B | P04259 | 2028.2973 |
| 6304 | PTCD1 | O75127 | 2026.752706 |
| 6305 | NKTR | P30414 | 2022.261219 |
| 6306 | PRMT9 | Q6P2P2 | 2022.181947 |
| 6307 | EXT2 | Q93063 | 2022.111703 |
| 6308 | FBXO21 | O94952 | 2021.71764 |
| 6309 | OAF | Q86UD1 | 2016.889583 |
| 6310 | HACE1 | Q8IYU2 | 2016.757973 |
| 6311 | STRADA | Q7RTN6 | 2016.456043 |
| 6312 | PDZD8 | Q8NEN9 | 2016.312667 |
| 6313 | APBA3 | O96018 | 2013.708048 |
| 6314 | VKORC1 | Q9BQB6 | 2011.915 |
| 6315 | KIDINS220 | Q9ULH0 | 2011.800861 |
| 6316 | MAP4K3 | Q8IVH8 | 2011.638574 |
| 6317 | LAGE3 | Q14657 | 2011.566714 |
| 6318 | RASSF8 | Q8NHQ8 | 2010.076731 |
| 6319 | ZNF608 | Q9ULD9 | 2009.934 |
| 6320 | DHX40 | Q8IX18 | 2009.31325 |
| 6321 | USP35 | Q9P2H5 | 2008.327755 |
| 6322 | COL3A1 | P02461 | 2001.787265 |
| 6323 | MAD2L1 | Q13257 | 1998.747692 |
| 6324 | YEATS2 | Q9ULM3 | 1993.795467 |
| 6325 | CCNC | P24863 | 1992.784615 |
| 6326 | RGS3 | P49796 | 1992.003185 |
| 6327 | BPTF | Q12830 | 1989.352601 |
| 6328 | YOD1 | Q5VVQ6 | 1984.063 |
| 6329 | TCF12 | Q99081 | 1983.601778 |
| 6330 | HDGFL1 | Q5TGJ6 | 1983.553333 |
| 6331 | TBC1D10A | Q9BXI6 | 1982.707207 |
| 6332 | KNSTRN | Q9Y448 | 1982.663333 |
| 6333 | KDELR2 | P33947 | 1981.986 |
| 6334 | ALKBH8 | Q96BT7 | 1979.669139 |
| 6335 | TAP2 | Q03519 | 1977.207103 |
| 6336 | ZNF592 | Q92610 | 1973.659462 |
| 6337 | RNF115 | Q9Y4L5 | 1970.920083 |
| 6338 | TYW1 | Q9NV66 | 1967.770912 |
| 6339 | MYO19 | Q96H55 | 1967.724976 |
| 6340 | TMEM102 | Q8N9M5 | 1965.1505 |
| 6341 | NAGS | Q8N159 | 1961.835769 |
| 6342 | UNC119 | Q13432 | 1955.543455 |
| 6343 | NLRX1 | Q86UT6 | 1953.7172 |
| 6344 | TAF6L | Q9Y6J9 | 1951.371586 |
| 6345 | TNPO2 | O14787 | 1950.423608 |
| 6346 | AGTPBP1 | Q9UPW5 | 1945.372894 |
| 6347 | ATF2 | P15336 | 1940.939563 |
| 6348 | PLAAT3 | P53816 | 1940.674143 |
| 6349 | HKDC1 | Q2TB90 | 1939.867255 |
| 6350 | SYNGR1 | O43759 | 1939.260286 |
| 6351 | SLC18B1 | Q6NT16 | 1932.487692 |
| 6352 | CHIC2 | Q9UKJ5 | 1930.870364 |
| 6353 | SLC35C2 | Q9NQQ7 | 1923.7444 |
| 6354 | MROH1 | Q8NDA8 | 1922.051671 |
| 6355 | CDK12 | Q9NYV4 | 1920.034577 |
| 6356 | ANKRD27 | Q96NW4 | 1919.741491 |
| 6357 | CRCP | O75575 | 1916.841143 |
| 6358 | TRAM2 | Q15035 | 1914.763176 |
| 6359 | NPHP3 | Q7Z494 | 1914.283641 |
| 6360 | CHCHD6 | Q9BRQ6 | 1913.579 |
| 6361 | ATPAF1 | Q5TC12 | 1912.165313 |
| 6362 | TFPI2 | P48307 | 1911.3657 |
| 6363 | ARNT | P27540 | 1911.031618 |
| 6364 | PRMT3 | O60678 | 1910.50804 |
| 6365 | CCDC106 | Q9BWC9 | 1907.488333 |
| 6366 | MGRN1 | O60291 | 1906.469577 |
| 6367 | TNFRSF12A | Q9NP84 | 1905.794286 |
| 6368 | PRKAG2 | Q9UGJ0 | 1901.608636 |
| 6369 | FER | P16591 | 1896.834455 |
| 6370 | SMG5 | Q9UPR3 | 1894.486585 |
| 6371 | NYNRIN | Q9P2P1 | 1893.711571 |
| 6372 | STRBP | Q96SI9 | 1892.076146 |
| 6373 | RPS6KC1 | Q96S38 | 1888.462377 |
| 6374 | TRMT6 | Q9UJA5 | 1886.822804 |
| 6375 | CYTH1 | Q15438 | 1886.713727 |
| 6376 | SIN3B | O75182 | 1884.415707 |
| 6377 | NUDT12 | Q9BQG2 | 1881.64075 |
| 6378 | TTC17 | Q96AE7 | 1881.164161 |
| 6379 | DUS1L | Q6P1R4 | 1880.19575 |
| 6380 | USF2 | Q15853 | 1878.1279 |
| 6381 | STARD3 | Q14849 | 1878.014864 |
| 6382 | FAM76B | Q5HYJ3 | 1876.067 |
| 6383 | HTR1B | P28222 | 1873.975111 |
| 6384 | NME6 | O75414 | 1871.159273 |
| 6385 | MED12 | Q93074 | 1864.106118 |
| 6386 | TUBA8 | Q9NY65 | 1862.113318 |
| 6387 | RAB34 | P0DI83 | 1860.114467 |
| 6388 | POLRMT | O00411 | 1853.497069 |
| 6389 | REPS2 | Q8NFH8 | 1851.626758 |
| 6390 | NOM1 | Q5C9Z4 | 1849.839091 |
| 6391 | LIPG | Q9Y5X9 | 1848.672409 |
| 6392 | HPSE | Q9Y251 | 1844.743583 |
| 6393 | ACD | Q96AP0 | 1842.285529 |
| 6394 | MARS2 | Q96GW9 | 1840.427867 |
| 6395 | DHRS3 | O75911 | 1838.939214 |
| 6396 | AKT2 | P31751 | 1837.999167 |
| 6397 | GK | P32189 | 1829.34731 |
| 6398 | IFT122 | Q9HBG6 | 1827.705852 |
| 6399 | TMEM160 | Q9NX00 | 1825.66 |
| 6400 | MACROD1 | Q9BQ69 | 1820.255714 |
| 6401 | POM121 | Q96HA1 | 1817.518906 |
| 6402 | DENND4A | Q7Z401 | 1817.345023 |
| 6403 | DDX60 | Q8IY21 | 1816.878722 |
| 6404 | MEAF6 | Q9HAF1 | 1815.665333 |
| 6405 | DGAT1 | O75907 | 1814.967588 |
| 6406 | HID1 | Q8IV36 | 1805.3698 |
| 6407 | MED1 | Q15648 | 1804.438787 |
| 6408 | PIGH | Q14442 | 1803.8711 |
| 6409 | RAB11A | P62491 | 1802.269929 |
| 6410 | KRT80 | Q6KB66 | 1800.903875 |
| 6411 | ADSS1 | Q8N142 | 1797.599652 |
| 6412 | MUL1 | Q969V5 | 1796.395889 |
| 6413 | TAF10 | Q12962 | 1791.612286 |
| 6414 | JAG2 | Q9Y219 | 1789.290216 |
| 6415 | FOXJ3 | Q9UPW0 | 1788.696412 |
| 6416 | PIP4P2 | Q8N4L2 | 1786.5754 |
| 6417 | NCOR1 | O75376 | 1786.388051 |
| 6418 | PIBF1 | Q8WXW3 | 1781.332322 |
| 6419 | RFX5 | P48382 | 1775.954053 |
| 6420 | AMOTL2 | Q9Y2J4 | 1769.524281 |
| 6421 | CASTOR2 | A6NHX0 | 1767.967714 |
| 6422 | TRIM41 | Q8WV44 | 1767.015758 |
| 6423 | RAD9A | Q99638 | 1766.512667 |
| 6424 | MTHFS | P49914 | 1755.721667 |
| 6425 | AURKB | Q96GD4 | 1744.235762 |
| 6426 | SUPT3H | O75486 | 1740.1671 |
| 6427 | PIAS1 | O75925 | 1738.677821 |
| 6428 | PCNX4 | Q63HM2 | 1736.712826 |
| 6429 | LTBP3 | Q9NS15 | 1735.780154 |
| 6430 | PHF21A | Q96BD5 | 1725.9104 |
| 6431 | PEX1 | O43933 | 1725.819382 |
| 6432 | PABIR1 | Q96E09 | 1725.568182 |
| 6433 | FUT1 | P19526 | 1721.121571 |
| 6434 | ALAS1 | P13196 | 1719.717556 |
| 6435 | ERC2 | O15083 | 1711.765833 |
| 6436 | ABRAXAS1 | Q6UWZ7 | 1707.4249 |
| 6437 | STON1 | Q9Y6Q2 | 1707.2295 |
| 6438 | ALMS1 | Q8TCU4 | 1706.455924 |
| 6439 | SNX14 | Q9Y5W7 | 1702.99693 |
| 6440 | FAM83G | A6ND36 | 1701.650452 |
| 6441 | COQ3 | Q9NZJ6 | 1699.588813 |
| 6442 | ALKBH3 | Q96Q83 | 1697.939294 |
| 6443 | ODF2 | Q5BJF6 | 1697.722214 |
| 6444 | PLXNA1 | Q9UIW2 | 1696.277911 |
| 6445 | NUS1 | Q96E22 | 1693.553125 |
| 6446 | SPATA33 | Q96N06 | 1693.361556 |
| 6447 | FAIM | Q9NVQ4 | 1688.1896 |
| 6448 | RPUSD2 | Q8IZ73 | 1681.165862 |
| 6449 | TADA1 | Q96BN2 | 1679.450929 |
| 6450 | AP5S1 | Q9NUS5 | 1676.867571 |
| 6451 | NIBAN1 | Q9BZQ8 | 1669.766872 |
| 6452 | CSN1S2 | P02663 | 1667.310769 |
| 6453 | DUSP22 | Q9NRW4 | 1663.698417 |
| 6454 | CKAP2 | Q8WWK9 | 1660.779611 |
| 6455 | AP4B1 | Q9Y6B7 | 1659.248167 |
| 6456 | LPIN2 | Q92539 | 1654.893317 |
| 6457 | HERPUD2 | Q9BSE4 | 1654.693 |
| 6458 | CWC22 | Q9HCG8 | 1654.439786 |
| 6459 | SLC2A10 | O95528 | 1653.450125 |
| 6460 | HLA-B | P01889 | 1652.823056 |
| 6461 | RPLP0P6 | Q8NHW5 | 1649.850667 |
| 6462 | PRSS3 | P35030 | 1647.551071 |
| 6463 | TSPAN7 | P41732 | 1647.358286 |
| 6464 | MTURN | Q8N3F0 | 1644.032333 |
| 6465 | SRCAP | Q6ZRS2 | 1638.154043 |
| 6466 | CRBN | Q96SW2 | 1637.806333 |
| 6467 | C2CD2 | Q9Y426 | 1637.36331 |
| 6468 | GTSF1 | Q8WW33 | 1635.754667 |
| 6469 | TSPAN6 | O43657 | 1635.101692 |
| 6470 | ZFC3H1 | O60293 | 1630.847309 |
| 6471 | DIP2A | Q14689 | 1627.474373 |
| 6472 | S100A4 | P26447 | 1620.641667 |
| 6473 | POMGNT2 | Q8NAT1 | 1619.120538 |
| 6474 | RBL2 | Q08999 | 1617.998161 |
| 6475 | SKA3 | Q8IX90 | 1616.117773 |
| 6476 | PHKA2 | P46019 | 1611.724895 |
| 6477 | ATRN | O75882 | 1609.691719 |
| 6478 | PYGM | P11217 | 1608.21751 |
| 6479 | USHBP1 | Q8N6Y0 | 1602.796038 |
| 6480 | ARHGAP21 | Q5T5U3 | 1601.897124 |
| 6481 | UNK | Q9C0B0 | 1601.833618 |
| 6482 | RANGRF | Q9HD47 | 1601.4055 |
| 6483 | C6orf89 | Q6UWU4 | 1598.948188 |
| 6484 | CEP97 | Q8IW35 | 1597.8223 |
| 6485 | CCDC169 | A6NNP5 | 1593.568 |
| 6486 | SPTY2D1 | Q68D10 | 1591.199429 |
| 6487 | TNFAIP1 | Q13829 | 1587.107095 |
| 6488 | SH3BP1 | Q9Y3L3 | 1586.180706 |
| 6489 | CPB2 | Q96IY4 | 1583.598136 |
| 6490 | PLA2G4C | Q9UP65 | 1581.451083 |
| 6491 | ACSS1 | Q9NUB1 | 1581.068606 |
| 6492 | RBM4B | Q9BQ04 | 1580.450211 |
| 6493 | RTN4IP1 | Q8WWV3 | 1578.302174 |
| 6494 | BRD8 | Q9H0E9 | 1577.713542 |
| 6495 | TMEM59 | Q9BXS4 | 1572.740071 |
| 6496 | CASP1 | P29466 | 1571.820632 |
| 6497 | SOX7 | Q9BT81 | 1569.5925 |
| 6498 | ZMIZ1 | Q9ULJ6 | 1566.242773 |
| 6499 | PRPS1 | P60891 | 1564.840875 |
| 6500 | GATB | O75879 | 1563.006167 |
| 6501 | LRRC31 | Q6UY01 | 1562.3604 |
| 6502 | ANKRD1 | Q15327 | 1555.888706 |
| 6503 | HSPB6 | O14558 | 1551.421429 |
| 6504 | SETD1A | O15047 | 1551.260375 |
| 6505 | SEC11C | Q9BY50 | 1551.1658 |
| 6506 | ALS2 | Q96Q42 | 1548.584311 |
| 6507 | PDE8A | O60658 | 1543.879955 |
| 6508 | CDC37L1 | Q7L3B6 | 1543.142833 |
| 6509 | CEP250 | Q9BV73 | 1542.085757 |
| 6510 | NFRKB | Q6P4R8 | 1541.901552 |
| 6511 | DOLK | Q9UPQ8 | 1540.421643 |
| 6512 | PARG | Q86W56 | 1539.848564 |
| 6513 | TMOD2 | Q9NZR1 | 1536.13935 |
| 6514 | ZFP36L2 | P47974 | 1532.5965 |
| 6515 | PINX1 | Q96BK5 | 1531.717643 |
| 6516 | ARL4A | P40617 | 1530.034222 |
| 6517 | GINS3 | Q9BRX5 | 1526.689786 |
| 6518 | SUFU | Q9UMX1 | 1526.018789 |
| 6519 | TEX2 | Q8IWB9 | 1524.082582 |
| 6520 | TENT4B | Q8NDF8 | 1524.011536 |
| 6521 | EPG5 | Q9HCE0 | 1520.499621 |
| 6522 | MOSPD1 | Q9UJG1 | 1520.159 |
| 6523 | ARL6 | Q9H0F7 | 1520.025333 |
| 6524 | BMP1 | P13497 | 1515.229765 |
| 6525 | SDCCAG8 | Q86SQ7 | 1514.8581 |
| 6526 | PIGO | Q8TEQ8 | 1514.389978 |
| 6527 | PEAR1 | Q5VY43 | 1512.940286 |
| 6528 | LMBRD2 | Q68DH5 | 1511.5768 |
| 6529 | FOXP4 | Q8IVH2 | 1511.11163 |
| 6530 | UBE2G2 | P60604 | 1510.2092 |
| 6531 | PDSS2 | Q86YH6 | 1504.511346 |
| 6532 | SARM1 | Q6SZW1 | 1503.196114 |
| 6533 | TNIP2 | Q8NFZ5 | 1499.378136 |
| 6534 | TMCO3 | Q6UWJ1 | 1496.627656 |
| 6535 | SSR2 | P43308 | 1494.98 |
| 6536 | AKAP11 | Q9UKA4 | 1490.281693 |
| 6537 | SEMA3F | Q13275 | 1485.83881 |
| 6538 | TDRD5 | Q8NAT2 | 1479.444 |
| 6539 | HIC1 | Q14526 | 1477.11129 |
| 6540 | DSG1 | Q02413 | 1472.884585 |
| 6541 | TACO1 | Q9BSH4 | 1467.8062 |
| 6542 | ZFYVE19 | Q96K21 | 1465.183966 |
| 6543 | BHMT | Q93088 | 1464.975333 |
| 6544 | WDR81 | Q562E7 | 1460.536398 |
| 6545 | NEDD9 | Q14511 | 1459.092767 |
| 6546 | MARCHF7 | Q9H992 | 1458.886688 |
| 6547 | BBX | Q8WY36 | 1457.414878 |
| 6548 | SCOC | Q9UIL1 | 1456.569571 |
| 6549 | CABLES1 | Q8TDN4 | 1453.481211 |
| 6550 | CACNB2 | Q08289 | 1450.7675 |
| 6551 | HDAC3 | O15379 | 1444.26775 |
| 6552 | IFNGR1 | P15260 | 1443.938526 |
| 6553 | USP32 | Q8NFA0 | 1442.629961 |
| 6554 | ZBTB10 | Q96DT7 | 1442.418205 |
| 6555 | MSL1 | Q68DK7 | 1441.690167 |
| 6556 | GORAB | Q5T7V8 | 1440.54275 |
| 6557 | PIK3C2B | O00750 | 1439.310697 |
| 6558 | ARID2 | Q68CP9 | 1438.153286 |
| 6559 | MARF1 | Q9Y4F3 | 1437.30983 |
| 6560 | FBXO11 | Q86XK2 | 1436.7944 |
| 6561 | ERCC5 | P28715 | 1436.468415 |
| 6562 | NF1 | P21359 | 1436.354643 |
| 6563 | ZFYVE26 | Q68DK2 | 1435.321672 |
| 6564 | GNAI1 | P63096 | 1434.862647 |
| 6565 | SNAP47 | Q5SQN1 | 1433.679933 |
| 6566 | TARS3 | A2RTX5 | 1433.1753 |
| 6567 | GCAT | O75600 | 1432.448647 |
| 6568 | AGBL3 | Q8NEM8 | 1427.879385 |
| 6569 | PAXIP1 | Q6ZW49 | 1427.576976 |
| 6570 | LRRC41 | Q15345 | 1427.574229 |
| 6571 | COMTD1 | Q86VU5 | 1423.873654 |
| 6572 | PIGN | O95427 | 1422.662967 |
| 6573 | MME | P08473 | 1421.13865 |
| 6574 | OMA1 | Q96E52 | 1420.629667 |
| 6575 | TCF4 | P15884 | 1419.0952 |
| 6576 | SERPINA7 | P05543 | 1417.086913 |
| 6577 | THBS3 | P49746 | 1412.619541 |
| 6578 | ELF2 | Q15723 | 1409.523577 |
| 6579 | LATS2 | Q9NRM7 | 1406.691978 |
| 6580 | TUBGCP5 | Q96RT8 | 1404.602679 |
| 6581 | DEF8 | Q6ZN54 | 1402.175808 |
| 6582 | SSH1 | Q8WYL5 | 1400.331676 |
| 6583 | TMEM69 | Q5SWH9 | 1395.823 |
| 6584 | COQ7 | Q99807 | 1395.494857 |
| 6585 | KCTD16 | Q68DU8 | 1390.765917 |
| 6586 | HACD2 | Q6Y1H2 | 1390.743846 |
| 6587 | SLC25A17 | O43808 | 1389.7916 |
| 6588 | CUL7 | Q14999 | 1386.222759 |
| 6589 | DCP2 | Q8IU60 | 1386.019905 |
| 6590 | CPA4 | Q9UI42 | 1382.612091 |
| 6591 | SCLT1 | Q96NL6 | 1382.535452 |
| 6592 | BRD7 | Q9NPI1 | 1378.395281 |
| 6593 | SLC9A6 | Q92581 | 1376.80276 |
| 6594 | CSTA | P01040 | 1375.925286 |
| 6595 | LPIN1 | Q14693 | 1367.7455 |
| 6596 | FBXW5 | Q969U6 | 1367.388893 |
| 6597 | IFT22 | Q9H7X7 | 1367.285909 |
| 6598 | LNX2 | Q8N448 | 1366.857308 |
| 6599 | GDAP2 | Q9NXN4 | 1366.808111 |
| 6600 | PLCD3 | Q8N3E9 | 1364.723316 |
| 6601 | KRIT1 | O00522 | 1363.847349 |
| 6602 | MBTPS1 | Q14703 | 1360.723 |
| 6603 | CCND3 | P30281 | 1360.704867 |
| 6604 | WDTC1 | Q8N5D0 | 1353.736026 |
| 6605 | RCE1 | Q9Y256 | 1351.8459 |
| 6606 | FHIP1B | Q8N612 | 1351.2125 |
| 6607 | DNAH3 | Q8TD57 | 1348.376126 |
| 6608 | TULP3 | O75386 | 1345.964529 |
| 6609 | SEMA3C | Q99985 | 1345.851025 |
| 6610 | SLC25A32 | Q9H2D1 | 1345.30725 |
| 6611 | RAD18 | Q9NS91 | 1342.037188 |
| 6612 | ING1 | Q9UK53 | 1341.684955 |
| 6613 | OCLN | Q16625 | 1340.843063 |
| 6614 | JAK2 | O60674 | 1340.774937 |
| 6615 | ZNF830 | Q96NB3 | 1339.476333 |
| 6616 | FEM1B | Q9UK73 | 1338.394139 |
| 6617 | C9 | P02748 | 1326.872074 |
| 6618 | GSTCD | Q8NEC7 | 1323.595125 |
| 6619 | RNF149 | Q8NC42 | 1323.328778 |
| 6620 | TLR4 | O00206 | 1318.5819 |
| 6621 | KIAA1522 | Q9P206 | 1317.771667 |
| 6622 | SNX21 | Q969T3 | 1317.443 |
| 6623 | MIER2 | Q8N344 | 1316.99395 |
| 6624 | ZNF579 | Q8NAF0 | 1316.60687 |
| 6625 | CASP14 | P31944 | 1315.032333 |
| 6626 | WWP1 | Q9H0M0 | 1314.00437 |
| 6627 | CEACAM1 | P13688 | 1311.964923 |
| 6628 | PCNX3 | Q9H6A9 | 1311.860494 |
| 6629 | USP12 | O75317 | 1310.212211 |
| 6630 | RAD51C | O43502 | 1309.253278 |
| 6631 | INO80E | Q8NBZ0 | 1307.241333 |
| 6632 | REEP6 | Q96HR9 | 1302.4365 |
| 6633 | PLAT | P00750 | 1302.120968 |
| 6634 | EIF4ENIF1 | Q9NRA8 | 1300.687833 |
| 6635 | USP36 | Q9P275 | 1298.767656 |
| 6636 | ADAM23 | O75077 | 1297.618293 |
| 6637 | CBX2 | Q14781 | 1290.317172 |
| 6638 | PACC1 | Q9H813 | 1288.900615 |
| 6639 | RNF166 | Q96A37 | 1286.3959 |
| 6640 | DPF3 | Q92784 | 1283.60395 |
| 6641 | NACC2 | Q96BF6 | 1283.587284 |
| 6642 | DGKB | Q9Y6T7 | 1282.812286 |
| 6643 | INTS5 | Q6P9B9 | 1281.42103 |
| 6644 | OGN | P20774 | 1280.841 |
| 6645 | MVB12B | Q9H7P6 | 1277.683994 |
| 6646 | VARS2 | Q5ST30 | 1276.584609 |
| 6647 | PHETA2 | Q6ICB4 | 1273.706154 |
| 6648 | TUT1 | Q9H6E5 | 1273.140077 |
| 6649 | SLC19A2 | O60779 | 1272.4946 |
| 6650 | ARHGEF6 | Q15052 | 1269.987463 |
| 6651 | PSEN2 | P49810 | 1264.857063 |
| 6652 | FAM98C | Q17RN3 | 1264.3834 |
| 6653 | CYP2U1 | Q7Z449 | 1263.087897 |
| 6654 | KMT2E | Q8IZD2 | 1261.501065 |
| 6655 | SUSD6 | Q92537 | 1260.294333 |
| 6656 | SMYD4 | Q8IYR2 | 1256.330364 |
| 6657 | C12orf4 | Q9NQ89 | 1254.75989 |
| 6658 | PRG4 | Q92954 | 1250.363863 |
| 6659 | HERPUD1 | Q15011 | 1250.282615 |
| 6660 | HPS5 | Q9UPZ3 | 1249.787967 |
| 6661 | NSG1 | P42857 | 1243.9906 |
| 6662 | FBN1 | P35555 | 1243.716517 |
| 6663 | ATP13A2 | Q9NQ11 | 1241.33224 |
| 6664 | PHF3 | Q92576 | 1240.580531 |
| 6665 | MEX3A | A1L020 | 1237.769727 |
| 6666 | ALKBH7 | Q9BT30 | 1237.381846 |
| 6667 | RAD1 | O60671 | 1234.060176 |
| 6668 | HMGXB4 | Q9UGU5 | 1233.225238 |
| 6669 | KAT14 | Q9H8E8 | 1231.108972 |
| 6670 | BLZF1 | Q9H2G9 | 1230.594417 |
| 6671 | WRN | Q14191 | 1228.56358 |
| 6672 | CDAN1 | Q8IWY9 | 1228.28294 |
| 6673 | CCDC174 | Q6PII3 | 1224.516524 |
| 6674 | USP33 | Q8TEY7 | 1221.987224 |
| 6675 | NCOA6 | Q14686 | 1220.540717 |
| 6676 | RIOX2 | Q8IUF8 | 1216.575015 |
| 6677 | DSG4 | Q86SJ6 | 1215.032571 |
| 6678 | USP3 | Q9Y6I4 | 1213.457308 |
| 6679 | LMTK2 | Q8IWU2 | 1212.456717 |
| 6680 | ETNK1 | Q9HBU6 | 1212.114143 |
| 6681 | CCDC171 | Q6TFL3 | 1211.46875 |
| 6682 | H1-1 | Q02539 | 1210.227286 |
| 6683 | SGCE | O43556 | 1206.658944 |
| 6684 | BRD2 | P25440 | 1204.996232 |
| 6685 | CALHM2 | Q9HA72 | 1203.004111 |
| 6686 | POLL | Q9UGP5 | 1202.8974 |
| 6687 | ARG1 | P05089 | 1202.526941 |
| 6688 | TSC22D2 | O75157 | 1197.875583 |
| 6689 | PHETA1 | Q8N4B1 | 1194.439182 |
| 6690 | NTHL1 | P78549 | 1193.901235 |
| 6691 | TTC26 | A0AVF1 | 1191.932414 |
| 6692 | ZNF281 | Q9Y2X9 | 1191.719167 |
| 6693 | HECTD4 | Q9Y4D8 | 1191.00239 |
| 6694 | MFSD14B | Q5SR56 | 1190.676462 |
| 6695 | ZBED1 | O96006 | 1188.248723 |
| 6696 | SNED1 | Q8TER0 | 1187.842217 |
| 6697 | FLT1 | P17948 | 1186.146658 |
| 6698 | ABHD17A | Q96GS6 | 1185.975625 |
| 6699 | TDP1 | Q9NUW8 | 1185.11969 |
| 6700 | SF3B5 | Q9BWJ5 | 1182.4676 |
| 6701 | RAF1 | P04049 | 1180.656053 |
| 6702 | RAB38 | P57729 | 1180.36225 |
| 6703 | ZWINT | O95229 | 1179.323857 |
| 6704 | UNG | P13051 | 1177.712692 |
| 6705 | CHEK1 | O14757 | 1174.793375 |
| 6706 | REX1BD | Q96EN9 | 1171.659167 |
| 6707 | ZNF581 | Q9P0T4 | 1171.063333 |
| 6708 | CABIN1 | Q9Y6J0 | 1168.761832 |
| 6709 | WDCP | Q9H6R7 | 1166.642971 |
| 6710 | ZBTB33 | Q86T24 | 1163.945412 |
| 6711 | RGS4 | P49798 | 1161.827 |
| 6712 | CBLB | Q13191 | 1160.394019 |
| 6713 | CHN1 | P15882 | 1160.348192 |
| 6714 | MTRES1 | Q9P0P8 | 1156.094923 |
| 6715 | PARS2 | Q7L3T8 | 1155.07076 |
| 6716 | ATP12A | P54707 | 1148.839192 |
| 6717 | MAP2K5 | Q13163 | 1147.559591 |
| 6718 | KBTBD4 | Q9NVX7 | 1143.965231 |
| 6719 | TLK1 | Q9UKI8 | 1143.901088 |
| 6720 | NUSAP1 | Q9BXS6 | 1137.326962 |
| 6721 | SPATA20 | Q8TB22 | 1136.54137 |
| 6722 | BRMS1L | Q5PSV4 | 1135.91725 |
| 6723 | ATAD2 | Q6PL18 | 1134.640414 |
| 6724 | PIK3R2 | O00459 | 1134.002744 |
| 6725 | TCTN3 | Q6NUS6 | 1133.353944 |
| 6726 | POLA1 | P09884 | 1132.816479 |
| 6727 | SGCB | Q16585 | 1131.761765 |
| 6728 | RIN1 | Q13671 | 1131.472389 |
| 6729 | TAGLN3 | Q9UI15 | 1131.302857 |
| 6730 | CDIP1 | Q9H305 | 1131.271 |
| 6731 | ROBO1 | Q9Y6N7 | 1130.197086 |
| 6732 | USP34 | Q70CQ2 | 1125.281141 |
| 6733 | MOCS1 | Q9NZB8 | 1125.226111 |
| 6734 | FAM118A | Q9NWS6 | 1124.430579 |
| 6735 | PIAS3 | Q9Y6X2 | 1123.7604 |
| 6736 | HPS3 | Q969F9 | 1123.544821 |
| 6737 | USP53 | Q70EK8 | 1121.430983 |
| 6738 | ABCG1 | P45844 | 1120.97431 |
| 6739 | ORC3 | Q9UBD5 | 1116.85645 |
| 6740 | SMPDL3A | Q92484 | 1113.692773 |
| 6741 | DCAF11 | Q8TEB1 | 1113.221417 |
| 6742 | DFFB | O76075 | 1112.877533 |
| 6743 | SH3BP5 | O60239 | 1111.991 |
| 6744 | P3H2 | Q8IVL5 | 1110.207342 |
| 6745 | DCUN1D3 | Q8IWE4 | 1106.361188 |
| 6746 | IFT81 | Q8WYA0 | 1106.136375 |
| 6747 | ZNF618 | Q5T7W0 | 1105.560122 |
| 6748 | SOX17 | Q9H6I2 | 1104.332 |
| 6749 | MAML1 | Q92585 | 1100.13569 |
| 6750 | SGK1 | O00141 | 1096.785579 |
| 6751 | GMEB2 | Q9UKD1 | 1096.466733 |
| 6752 | KLHL21 | Q9UJP4 | 1095.011989 |
| 6753 | UMODL1 | Q5DID0 | 1094.719149 |
| 6754 | CDK5RAP2 | Q96SN8 | 1094.015391 |
| 6755 | ILRUN | Q9H6K1 | 1087.64725 |
| 6756 | SH2B1 | Q9NRF2 | 1085.574444 |
| 6757 | UTP23 | Q9BRU9 | 1085.473778 |
| 6758 | PCF11 | O94913 | 1085.440012 |
| 6759 | AURKA | O14965 | 1081.082955 |
| 6760 | MED26 | O95402 | 1079.522607 |
| 6761 | SUPT20H | Q8NEM7 | 1077.098969 |
| 6762 | B9D1 | Q9UPM9 | 1073.634444 |
| 6763 | NFIX | Q14938 | 1066.357345 |
| 6764 | NEIL2 | Q969S2 | 1065.168278 |
| 6765 | PKLR | P30613 | 1062.917515 |
| 6766 | INSR | P06213 | 1059.115808 |
| 6767 | TAF2 | Q6P1X5 | 1058.039797 |
| 6768 | CYFIP2 | Q96F07 | 1056.406838 |
| 6769 | WDR54 | Q9H977 | 1053.9825 |
| 6770 | PLTP | P55058 | 1051.784167 |
| 6771 | NCOA7 | Q8NI08 | 1047.910596 |
| 6772 | USF1 | P22415 | 1047.677 |
| 6773 | ATP8A1 | Q9Y2Q0 | 1047.216691 |
| 6774 | GSAP | A4D1B5 | 1045.929211 |
| 6775 | ALG8 | Q9BVK2 | 1045.775789 |
| 6776 | CEP89 | Q96ST8 | 1044.910452 |
| 6777 | TACC2 | O95359 | 1043.850416 |
| 6778 | SNX13 | Q9Y5W8 | 1043.045898 |
| 6779 | FBH1 | Q8NFZ0 | 1042.15537 |
| 6780 | MED6 | O75586 | 1041.685786 |
| 6781 | IQCB1 | Q15051 | 1041.0971 |
| 6782 | GINS1 | Q14691 | 1038.379615 |
| 6783 | INCENP | Q9NQS7 | 1035.868854 |
| 6784 | PIP4P1 | Q86T03 | 1035.642 |
| 6785 | ZMAT3 | Q9HA38 | 1034.587923 |
| 6786 | STAT5A | P42229 | 1033.574452 |
| 6787 | ASNSD1 | Q9NWL6 | 1032.430029 |
| 6788 | BMP4 | P12644 | 1029.383389 |
| 6789 | RNF2 | Q99496 | 1029.223813 |
| 6790 | SPPL3 | Q8TCT6 | 1028.667091 |
| 6791 | ENGASE | Q8NFI3 | 1025.23539 |
| 6792 | TCP11L2 | Q8N4U5 | 1024.869643 |
| 6793 | DDIT4 | Q9NX09 | 1024.3642 |
| 6794 | NKAPD1 | Q6ZUT1 | 1022.941143 |
| 6795 | PREX2 | Q70Z35 | 1022.313707 |
| 6796 | DOP1B | Q9Y3R5 | 1021.219374 |
| 6797 | DDX59 | Q5T1V6 | 1021.078676 |
| 6798 | DENND5A | Q6IQ26 | 1019.650708 |
| 6799 | APOL3 | O95236 | 1018.956333 |
| 6800 | SKA2 | Q8WVK7 | 1018.9478 |
| 6801 | PRKX | P51817 | 1017.694188 |
| 6802 | DHRS13 | Q6UX07 | 1016.773333 |
| 6803 | PRMT2 | P55345 | 1016.460333 |
| 6804 | NAB2 | Q15742 | 1016.264045 |
| 6805 | MORC4 | Q8TE76 | 1014.50717 |
| 6806 | ALKBH1 | Q13686 | 1013.128368 |
| 6807 | RHOJ | Q9H4E5 | 1012.257375 |
| 6808 | CNTROB | Q8N137 | 1011.079333 |
| 6809 | CIB1 | Q99828 | 1010.093429 |
| 6810 | SPATA2L | Q8IUW3 | 1009.3643 |
| 6811 | CRIPT | Q9P021 | 1005.82 |
| 6812 | RNASEH2B | Q5TBB1 | 1003.717188 |
| 6813 | C5 | P01031 | 999.4477558 |
| 6814 | PCDH12 | Q9NPG4 | 999.4141509 |
| 6815 | SDHAF2 | Q9NX18 | 998.6256 |
| 6816 | PPP2R2D | Q66LE6 | 996.9227778 |
| 6817 | MTHFR | P42898 | 996.4150909 |
| 6818 | FZR1 | Q9UM11 | 993.1101071 |
| 6819 | COQ2 | Q96H96 | 992.578875 |
| 6820 | EPB41L4A | Q9HCS5 | 992.0803846 |
| 6821 | CFH | P08603 | 991.5730323 |
| 6822 | RBP4 | P02753 | 988.8736 |
| 6823 | MRS2 | Q9HD23 | 988.57968 |
| 6824 | ARSK | Q6UWY0 | 984.559 |
| 6825 | TLK2 | Q86UE8 | 981.3873513 |
| 6826 | CASP2 | P42575 | 979.0656667 |
| 6827 | DCAF1 | Q9Y4B6 | 978.0375 |
| 6828 | PMS2 | P54278 | 977.9325532 |
| 6829 | TENT2 | Q6PIY7 | 970.8325833 |
| 6830 | PDK2 | Q15119 | 970.387 |
| 6831 | KCTD3 | Q9Y597 | 969.9212195 |
| 6832 | BMPR1A | P36894 | 968.53444 |
| 6833 | MED29 | Q9NX70 | 964.7193 |
| 6834 | DXO | O77932 | 963.7367895 |
| 6835 | UHRF1BP1L | A0JNW5 | 962.2331098 |
| 6836 | FAM107A | O95990 | 959.5433 |
| 6837 | ARFGEF3 | Q5TH69 | 956.2868529 |
| 6838 | CKB | P12277 | 955.7355294 |
| 6839 | C9orf72 | Q96LT7 | 954.0973478 |
| 6840 | NPR1 | P16066 | 949.9423962 |
| 6841 | RFXAP | O00287 | 947.1305 |
| 6842 | GPR108 | Q9NPR9 | 946.42476 |
| 6843 | MPPE1 | Q53F39 | 946.1094118 |
| 6844 | ZBTB38 | Q8NAP3 | 944.5704262 |
| 6845 | CUX1 | Q13948 | 944.1358333 |
| 6846 | NOX4 | Q9NPH5 | 942.6806667 |
| 6847 | SETD2 | Q9BYW2 | 942.062735 |
| 6848 | KMT2A | Q03164 | 938.7156775 |
| 6849 | TASOR2 | Q5VWN6 | 938.5797679 |
| 6850 | TTC3 | P53804 | 937.5628421 |
| 6851 | TAB3 | Q8N5C8 | 936.8755517 |
| 6852 | PRPH | P41219 | 934.6812903 |
| 6853 | PROS1 | P07225 | 933.3338 |
| 6854 | LYZ | P00698 | 933.028 |
| 6855 | DGKQ | P52824 | 930.8851556 |
| 6856 | SENP7 | Q9BQF6 | 930.3758723 |
| 6857 | ZDHHC18 | Q9NUE0 | 929.5178222 |
| 6858 | ZDHHC17 | Q8IUH5 | 928.0792963 |
| 6859 | TMEM98 | Q9Y2Y6 | 927.8637778 |
| 6860 | AHDC1 | Q5TGY3 | 922.6250464 |
| 6861 | NEK1 | Q96PY6 | 918.3785606 |
| 6862 | LTO1 | Q8WV07 | 915.9960909 |
| 6863 | MCUR1 | Q96AQ8 | 914.25825 |
| 6864 | SCARF2 | Q96GP6 | 907.3740513 |
| 6865 | MANBAL | Q9NQG1 | 906.68625 |
| 6866 | MTERF4 | Q7Z6M4 | 903.1074348 |
| 6867 | ADAMTS13 | Q76LX8 | 902.7439683 |
| 6868 | CCDC14 | Q49A88 | 902.408125 |
| 6869 | SH2B3 | Q9UQQ2 | 901.167 |
| 6870 | ANGEL2 | Q5VTE6 | 900.4722593 |
| 6871 | HMG20A | Q9NP66 | 897.8825385 |
| 6872 | ACAD11 | Q709F0 | 897.7715641 |
| 6873 | XPNPEP2 | O43895 | 896.7809375 |
| 6874 | RASGRF1 | Q13972 | 895.9493651 |
| 6875 | RIPK3 | Q9Y572 | 892.3523077 |
| 6876 | DGKE | P52429 | 889.4858077 |
| 6877 | PZP | P20742 | 888.8317213 |
| 6878 | COX6B1 | P14854 | 888.6651429 |
| 6879 | SLC26A11 | Q86WA9 | 887.70043 |
| 6880 | IFT57 | Q9NWB7 | 887.3742083 |
| 6881 | INSYN2B | A6NMK8 | 884.8371923 |
| 6882 | NSD3 | Q9BZ95 | 883.4967246 |
| 6883 | PCDH7 | O60245 | 883.0549348 |
| 6884 | CDSN | Q15517 | 880.363 |
| 6885 | ADAM7 | Q9H2U9 | 877.8091892 |
| 6886 | ZBTB21 | Q9ULJ3 | 876.6651157 |
| 6887 | RPS6KB1 | P23443 | 875.6757619 |
| 6888 | CEP290 | O15078 | 875.0049589 |
| 6889 | SIK3 | Q9Y2K2 | 873.6622294 |
| 6890 | AMIGO2 | Q86SJ2 | 869.6702308 |
| 6891 | EFCAB3 | Q8N7B9 | 868.8569231 |
| 6892 | LRRC45 | Q96CN5 | 866.5383171 |
| 6893 | ZNF106 | Q9H2Y7 | 863.3641398 |
| 6894 | SLC66A3 | Q8N755 | 863.2892222 |
| 6895 | MTUS1 | Q9ULD2 | 863.2766563 |
| 6896 | RHPN2 | Q8IUC4 | 863.1814146 |
| 6897 | PRMT7 | Q9NVM4 | 859.5822727 |
| 6898 | CENPH | Q9H3R5 | 857.1140667 |
| 6899 | UBXN2A | P68543 | 855.7485 |
| 6900 | EVL | Q9UI08 | 855.1667143 |
| 6901 | LCOR | Q96JN0 | 851.69045 |
| 6902 | OSGEPL1 | Q9H4B0 | 851.5247059 |
| 6903 | MLXIP | Q9HAP2 | 851.1724625 |
| 6904 | ZNF362 | Q5T0B9 | 850.9314286 |
| 6905 | TPRN | Q4KMQ1 | 849.7723429 |
| 6906 | CLASRP | Q8N2M8 | 848.1958421 |
| 6907 | RSBN1 | Q5VWQ0 | 847.492 |
| 6908 | DIP2C | Q9Y2E4 | 846.1615915 |
| 6909 | JUNB | P17275 | 844.62 |
| 6910 | PGS1 | Q32NB8 | 843.3765417 |
| 6911 | SEPTIN3 | Q9UH03 | 842.2687059 |
| 6912 | COL1A2 | P08123 | 840.7143521 |
| 6913 | NCOA4 | Q13772 | 839.8434 |
| 6914 | KRT13 | P13646 | 837.6736786 |
| 6915 | EP300 | Q09472 | 836.3538919 |
| 6916 | WDR19 | Q8NEZ3 | 834.288961 |
| 6917 | FBXW8 | Q8N3Y1 | 828.4936296 |
| 6918 | PLEKHM1 | Q9Y4G2 | 827.1218125 |
| 6919 | SETD9 | Q8NE22 | 826.04375 |
| 6920 | SYT11 | Q9BT88 | 824.7939474 |
| 6921 | JADE3 | Q92613 | 824.430225 |
| 6922 | TATDN3 | Q17R31 | 824.1 |
| 6923 | POMT1 | Q9Y6A1 | 823.0438286 |
| 6924 | IRAK2 | O43187 | 821.4209375 |
| 6925 | MADD | Q8WXG6 | 821.3490128 |
| 6926 | FAM120C | Q9NX05 | 820.3325455 |
| 6927 | FPGT | O14772 | 820.1376471 |
| 6928 | NEK3 | P51956 | 820.046 |
| 6929 | PIEZO2 | Q9H5I5 | 818.5642446 |
| 6930 | NSD2 | O96028 | 814.7688514 |
| 6931 | KDM6A | O15550 | 814.569623 |
| 6932 | CFAP298 | P57076 | 813.0667333 |
| 6933 | HECW2 | Q9P2P5 | 812.8086222 |
| 6934 | PNPLA4 | P41247 | 811.7216667 |
| 6935 | BNIP3 | Q12983 | 811.1663636 |
| 6936 | ADCY9 | O60503 | 807.6777846 |
| 6937 | ATP7B | P35670 | 807.4116552 |
| 6938 | ARL5A | Q9Y689 | 806.4671 |
| 6939 | IFFO2 | Q5TF58 | 806.12805 |
| 6940 | COQ8B | Q96D53 | 805.3525667 |
| 6941 | IFT140 | Q96RY7 | 802.7664756 |
| 6942 | RHOF | Q9HBH0 | 801.2280833 |
| 6943 | TBC1D7 | Q9P0N9 | 798.8295 |
| 6944 | RNF19A | Q9NV58 | 798.3028889 |
| 6945 | COL1A1 | P02452 | 797.9710778 |
| 6946 | DALRD3 | Q5D0E6 | 796.9621154 |
| 6947 | RBM23 | Q86U06 | 795.8763684 |
| 6948 | SLC7A14 | Q8TBB6 | 795.6484615 |
| 6949 | SLC25A40 | Q8TBP6 | 794.1728125 |
| 6950 | WDR35 | Q9P2L0 | 792.4587188 |
| 6951 | LCMT2 | O60294 | 791.5482759 |
| 6952 | MT-CO3 | P00414 | 790.8184 |
| 6953 | SLC9A7 | Q96T83 | 787.6346875 |
| 6954 | APOA2 | P02652 | 787.0676 |
| 6955 | LRRC1 | Q9BTT6 | 786.601 |
| 6956 | KLHL13 | Q9P2N7 | 786.2071429 |
| 6957 | MRPL57 | Q9BQC6 | 784.79 |
| 6958 | MAFK | O60675 | 784.0013 |
| 6959 | SFXN4 | Q6P4A7 | 780.1432353 |
| 6960 | IFRD1 | O00458 | 775.8588636 |
| 6961 | LGALS7 | P47929 | 774.403 |
| 6962 | NTAQ1 | Q96HA8 | 773.14475 |
| 6963 | ZCRB1 | Q8TBF4 | 770.5764286 |
| 6964 | EPN3 | Q9H201 | 770.51205 |
| 6965 | VGLL4 | Q14135 | 767.7008125 |
| 6966 | PKHD1L1 | Q86WI1 | 758.3985853 |
| 6967 | CCNT2 | O60583 | 757.9066379 |
| 6968 | RAB11FIP1 | Q6WKZ4 | 752.854537 |
| 6969 | MARCHF6 | O60337 | 749.0337143 |
| 6970 | VCPKMT | Q9H867 | 748.8062222 |
| 6971 | CNNM4 | Q6P4Q7 | 746.7917317 |
| 6972 | JMY | Q8N9B5 | 746.1553488 |
| 6973 | JMJD1C | Q15652 | 745.475625 |
| 6974 | TSTD2 | Q5T7W7 | 744.7772143 |
| 6975 | IFT172 | Q9UG01 | 743.9047264 |
| 6976 | RASA2 | Q15283 | 740.3449574 |
| 6977 | ZHX2 | Q9Y6X8 | 739.99225 |
| 6978 | WASHC2C | Q9Y4E1 | 738.8577969 |
| 6979 | POLR3E | Q9NVU0 | 734.18675 |
| 6980 | CES2 | O00748 | 732.4346667 |
| 6981 | ARHGAP12 | Q8IWW6 | 730.2991522 |
| 6982 | PMS2P11 | Q13670 | 728.0472308 |
| 6983 | KRT78 | Q8N1N4 | 725.6733333 |
| 6984 | CFLAR | O15519 | 725.6654074 |
| 6985 | TCF3 | P15923 | 722.813 |
| 6986 | CXCR4 | P61073 | 722.6336667 |
| 6987 | ATP6V1B1 | P15313 | 721.7721154 |
| 6988 | IKBKE | Q14164 | 721.7570769 |
| 6989 | CTSF | Q9UBX1 | 721.036 |
| 6990 | MED27 | Q6P2C8 | 718.9125 |
| 6991 | ATG2A | Q2TAZ0 | 717.6606835 |
| 6992 | TAF3 | Q5VWG9 | 714.8897188 |
| 6993 | CTBS | Q01459 | 711.7638947 |
| 6994 | ARHGAP27 | Q6ZUM4 | 708.7924118 |
| 6995 | MTERF3 | Q96E29 | 708.4197727 |
| 6996 | LHFPL2 | Q6ZUX7 | 708.3968333 |
| 6997 | PGPEP1 | Q9NXJ5 | 708.0498889 |
| 6998 | THSD7A | Q9UPZ6 | 707.8312564 |
| 6999 | RXRA | P19793 | 706.9004348 |
| 7000 | FAM76A | Q8TAV0 | 706.8152222 |
| 7001 | GGNBP2 | Q9H3C7 | 706.5301935 |
| 7002 | DLG5 | Q8TDM6 | 706.3602407 |
| 7003 | SHROOM1 | Q2M3G4 | 705.9500294 |
| 7004 | CHAF1B | Q13112 | 704.427963 |
| 7005 | SMUG1 | Q53HV7 | 701.469 |
| 7006 | EFHC1 | Q5JVL4 | 696.5602564 |
| 7007 | GTPBP3 | Q969Y2 | 696.22045 |
| 7008 | RPF1 | Q9H9Y2 | 695.4734375 |
| 7009 | POLG2 | Q9UHN1 | 694.3554167 |
| 7010 | THAP12 | O43422 | 692.601675 |
| 7011 | FBLN2 | P98095 | 690.4242128 |
| 7012 | PDE5A | O76074 | 690.3354694 |
| 7013 | TMEM219 | Q86XT9 | 690.0898889 |
| 7014 | ENKD1 | Q9H0I2 | 689.9917368 |
| 7015 | REXO1 | Q8N1G1 | 689.8733333 |
| 7016 | RSAD1 | Q9HA92 | 689.546087 |
| 7017 | ANK3 | Q12955 | 687.618307 |
| 7018 | KLHDC8B | Q8IXV7 | 683.4793125 |
| 7019 | ESPN | B1AK53 | 683.4296207 |
| 7020 | PDK3 | Q15120 | 682.8670455 |
| 7021 | KIF7 | Q2M1P5 | 682.4441053 |
| 7022 | ATP9B | O43861 | 676.5639855 |
| 7023 | TUBG2 | Q9NRH3 | 675.9358636 |
| 7024 | EDEM2 | Q9BV94 | 675.1411818 |
| 7025 | DYNC2I2 | Q96EX3 | 673.3766667 |
| 7026 | MANBA | O00462 | 672.3760488 |
| 7027 | UHRF1BP1 | Q6BDS2 | 671.3834615 |
| 7028 | KCNAB1 | Q14722 | 670.8765455 |
| 7029 | AMOTL1 | Q8IY63 | 669.0571957 |
| 7030 | TRMT11 | Q7Z4G4 | 668.7606 |
| 7031 | PFN3 | P60673 | 667.8466667 |
| 7032 | MTMR11 | A4FU01 | 667.4588235 |
| 7033 | WNK3 | Q9BYP7 | 667.2358108 |
| 7034 | ABHD2 | P08910 | 663.277 |
| 7035 | GAB1 | Q13480 | 661.4646429 |
| 7036 | TUBAL3 | A6NHL2 | 661.1308333 |
| 7037 | ATG4C | Q96DT6 | 661.0293333 |
| 7038 | CTNNBIP1 | Q9NSA3 | 660.9073333 |
| 7039 | PEG10 | Q86TG7 | 657.5330741 |
| 7040 | NAALADL2 | Q58DX5 | 656.4741 |
| 7041 | YAE1 | Q9NRH1 | 653.4443846 |
| 7042 | FKBP14 | Q9NWM8 | 653.17775 |
| 7043 | RNF34 | Q969K3 | 651.5454706 |
| 7044 | CAGE1 | Q8TC20 | 651.4619444 |
| 7045 | TMEM184B | Q9Y519 | 650.5097692 |
| 7046 | FBXL19 | Q6PCT2 | 644.313 |
| 7047 | MX1 | P20591 | 643.9022941 |
| 7048 | KIFC3 | Q9BVG8 | 642.8142653 |
| 7049 | CCDC168 | Q8NDH2 | 639.9738235 |
| 7050 | MIGA1 | Q8NAN2 | 639.4582059 |
| 7051 | SIPA1L2 | Q9P2F8 | 638.7896548 |
| 7052 | JMJD7 | P0C870 | 638.5788421 |
| 7053 | KLF10 | Q13118 | 638.1257727 |
| 7054 | IBTK | Q9P2D0 | 638.0532192 |
| 7055 | TOX2 | Q96NM4 | 636.6584667 |
| 7056 | KMT2D | O14686 | 636.6192416 |
| 7057 | DGCR6L | Q9BY27 | 636.493 |
| 7058 | LIMK1 | P53667 | 635.1250541 |
| 7059 | PCDHGA11 | Q9Y5H2 | 633.7000345 |
| 7060 | NRDE2 | Q9H7Z3 | 629.7520893 |
| 7061 | CEP192 | Q8TEP8 | 627.2994071 |
| 7062 | STK17B | O94768 | 626.5833333 |
| 7063 | PANK3 | Q9H999 | 624.8421818 |
| 7064 | CSGALNACT2 | Q8N6G5 | 624.8270606 |
| 7065 | AKR1C2 | P52895 | 621.6666111 |
| 7066 | GPR161 | Q8N6U8 | 621.2034091 |
| 7067 | WAC | Q9BTA9 | 621.0629231 |
| 7068 | KIAA0100 | Q14667 | 615.6656729 |
| 7069 | VPS13D | Q5THJ4 | 615.2548317 |
| 7070 | MTSS1 | O43312 | 614.9999063 |
| 7071 | HELLS | Q9NRZ9 | 613.6702326 |
| 7072 | MAST2 | Q6P0Q8 | 613.3886071 |
| 7073 | TBCK | Q8TEA7 | 610.5524878 |
| 7074 | TACSTD2 | P09758 | 610.2646429 |
| 7075 | MTMR3 | Q13615 | 609.7032769 |
| 7076 | MCOLN1 | Q9GZU1 | 605.6899583 |
| 7077 | CEP85 | Q6P2H3 | 605.380037 |
| 7078 | SEL1L3 | Q68CR1 | 604.2480408 |
| 7079 | ASB1 | Q9Y576 | 603.7838947 |
| 7080 | BLM | P54132 | 603.7458861 |
| 7081 | TRIM11 | Q96F44 | 603.4521364 |
| 7082 | CDCA7L | Q96GN5 | 603.2649231 |
| 7083 | TADA3 | O75528 | 601.5973 |
| 7084 | PLEKHA6 | Q9Y2H5 | 600.4232653 |
| 7085 | FOXO3 | O43524 | 594.925 |
| 7086 | FURIN | P09958 | 594.7974848 |
| 7087 | CRY1 | Q16526 | 594.0978387 |
| 7088 | ING3 | Q9NXR8 | 592.732 |
| 7089 | VPS54 | Q9P1Q0 | 592.4926957 |
| 7090 | EXD3 | Q8N9H8 | 589.0511333 |
| 7091 | SYDE1 | Q6ZW31 | 587.6772368 |
| 7092 | FUT4 | P22083 | 584.6981154 |
| 7093 | NSUN4 | Q96CB9 | 582.836875 |
| 7094 | RNFT1 | Q5M7Z0 | 582.28 |
| 7095 | PKP4 | Q99569 | 581.9462063 |
| 7096 | TIMM17A | Q99595 | 581.0041429 |
| 7097 | KIT | P10721 | 580.5854167 |
| 7098 | CDKN1B | P46527 | 580.4820909 |
| 7099 | POLI | Q9UNA4 | 579.3804872 |
| 7100 | RFTN2 | Q52LD8 | 579.28936 |
| 7101 | LACC1 | Q8IV20 | 578.4821667 |
| 7102 | MTX3 | Q5HYI7 | 576.38 |
| 7103 | PRR11 | Q96HE9 | 574.9047222 |
| 7104 | PCGF5 | Q86SE9 | 564.4325385 |
| 7105 | OTUD5 | Q96G74 | 561.9815 |
| 7106 | PCDHB2 | Q9Y5E7 | 558.7507857 |
| 7107 | MPV17L2 | Q567V2 | 558.1817778 |
| 7108 | USP38 | Q8NB14 | 558.1325116 |
| 7109 | PPP1R37 | O75864 | 553.7875517 |
| 7110 | SERPINF2 | P08697 | 553.685 |
| 7111 | METTL15 | A6NJ78 | 553.5185 |
| 7112 | GSTM2 | P28161 | 552.5210588 |
| 7113 | SMG7 | Q92540 | 549.6331395 |
| 7114 | GLDC | P23378 | 549.3102326 |
| 7115 | USP28 | Q96RU2 | 548.3794035 |
| 7116 | COL6A2 | P12110 | 547.3417 |
| 7117 | DUSP7 | Q16829 | 546.4905882 |
| 7118 | PRC1 | O43663 | 545.2577419 |
| 7119 | EID2 | Q8N6I1 | 545.1005 |
| 7120 | MAT1A | Q00266 | 542.3187895 |
| 7121 | ZFP91 | Q96JP5 | 541.6515417 |
| 7122 | ITGB8 | P26012 | 541.4054048 |
| 7123 | GPR19 | Q15760 | 540.3676923 |
| 7124 | STK17A | Q9UEE5 | 539.1708462 |
| 7125 | CMTR2 | Q8IYT2 | 536.3405714 |
| 7126 | TRAPPC14 | Q8WVR3 | 535.6265161 |
| 7127 | SERPINB3 | P29508 | 535.3999545 |
| 7128 | LTBP4 | Q8N2S1 | 532.5725362 |
| 7129 | MAGI3 | Q5TCQ9 | 531.8934865 |
| 7130 | METTL17 | Q9H7H0 | 531.4856667 |
| 7131 | ZNF512B | Q96KM6 | 529.8062632 |
| 7132 | COP1 | Q8NHY2 | 528.4150625 |
| 7133 | MARVELD1 | Q9BSK0 | 528.3734 |
| 7134 | RTKN | Q9BST9 | 527.298931 |
| 7135 | GPATCH4 | Q5T3I0 | 523.4501481 |
| 7136 | VPS13B | Q7Z7G8 | 522.7833316 |
| 7137 | TAL1 | P17542 | 521.7836667 |
| 7138 | NOSTRIN | Q8IVI9 | 521.6048966 |
| 7139 | #N/A | P00760 | 520.73025 |
| 7140 | MYO9A | B2RTY4 | 518.9674685 |
| 7141 | IRS2 | Q9Y4H2 | 518.2977234 |
| 7142 | PCDHGA7 | Q9Y5G6 | 514.557 |
| 7143 | EPC1 | Q9H2F5 | 513.6474211 |
| 7144 | RREB1 | Q92766 | 512.0872605 |
| 7145 | DIPK1A | Q5T7M9 | 510.4185714 |
| 7146 | TMEM177 | Q53S58 | 505.6628462 |
| 7147 | DHX34 | Q14147 | 504.5775968 |
| 7148 | PKMYT1 | Q99640 | 503.0221579 |
| 7149 | DDX60L | Q5H9U9 | 502.4891383 |
| 7150 | TASP1 | Q9H6P5 | 500.8678125 |
| 7151 | C3orf38 | Q5JPI3 | 499.42475 |
| 7152 | COL10A1 | Q03692 | 498.5139394 |
| 7153 | MGAT5 | Q09328 | 496.8745263 |
| 7154 | ZNF746 | Q6NUN9 | 496.8354167 |
| 7155 | RFX2 | P48378 | 496.2667647 |
| 7156 | SMARCD3 | Q6STE5 | 494.2361786 |
| 7157 | BNIP3L | O60238 | 492.9922222 |
| 7158 | UBXN8 | O00124 | 492.8506667 |
| 7159 | RCOR3 | Q9P2K3 | 492.0314 |
| 7160 | SIRT6 | Q8N6T7 | 490.4959444 |
| 7161 | GVINP1 | Q7Z2Y8 | 488.495839 |
| 7162 | STYK1 | Q6J9G0 | 487.69692 |
| 7163 | FLRT2 | O43155 | 487.2565161 |
| 7164 | NOS1 | P29475 | 483.8475676 |
| 7165 | CCNA1 | P78396 | 483.1425 |
| 7166 | HELZ2 | Q9BYK8 | 478.408889 |
| 7167 | FANCI | Q9NVI1 | 476.7807683 |
| 7168 | KHDC4 | Q7Z7F0 | 476.7185714 |
| 7169 | ASAP3 | Q8TDY4 | 475.4920882 |
| 7170 | TMEM161A | Q9NX61 | 474.2372727 |
| 7171 | UBTD2 | Q8WUN7 | 473.7765 |
| 7172 | TRUB2 | O95900 | 472.0469048 |
| 7173 | CCDC149 | Q6ZUS6 | 471.7615385 |
| 7174 | TNFRSF6B | O95407 | 470.7468462 |
| 7175 | ECHDC3 | Q96DC8 | 468.3915789 |
| 7176 | MGARP | Q8TDB4 | 468.0625556 |
| 7177 | LRP5 | O75197 | 467.5281067 |
| 7178 | NFIA | Q12857 | 467.3113333 |
| 7179 | POT1 | Q9NUX5 | 465.3636207 |
| 7180 | TBX20 | Q9UMR3 | 462.00435 |
| 7181 | TMEM41B | Q5BJD5 | 461.197 |
| 7182 | FSIP2 | Q5CZC0 | 459.0155028 |
| 7183 | TIMP2 | P16035 | 458.5441111 |
| 7184 | ZFAND1 | Q8TCF1 | 457.1487647 |
| 7185 | RNF169 | Q8NCN4 | 456.7860526 |
| 7186 | TRIM46 | Q7Z4K8 | 455.22925 |
| 7187 | TOPORS | Q9NS56 | 455.2254583 |
| 7188 | FAM184A | Q8NB25 | 455.0971642 |
| 7189 | SLC10A4 | Q96EP9 | 454.1033333 |
| 7190 | JADE2 | Q9NQC1 | 451.6882162 |
| 7191 | FAM89B | Q8N5H3 | 451.3414286 |
| 7192 | ZNF644 | Q9H582 | 451.2072609 |
| 7193 | CHAF1A | Q13111 | 449.9479 |
| 7194 | IFT80 | Q9P2H3 | 449.1296286 |
| 7195 | TIMM10B | Q9Y5J6 | 448.67375 |
| 7196 | CDCA5 | Q96FF9 | 448.4236154 |
| 7197 | NT5C1A | Q9BXI3 | 447.8542105 |
| 7198 | DENND4B | O75064 | 445.8756607 |
| 7199 | PYURF | Q96I23 | 444.3588 |
| 7200 | UNC50 | Q53HI1 | 442.752 |
| 7201 | COX6A1 | P12074 | 441.2476 |
| 7202 | FAM92A | A1XBS5 | 441.1752143 |
| 7203 | SDK2 | Q58EX2 | 441.0438182 |
| 7204 | PTOV1 | Q86YD1 | 441.0193913 |
| 7205 | ZNF384 | Q8TF68 | 440.5088571 |
| 7206 | TRIM68 | Q6AZZ1 | 439.2290833 |
| 7207 | CXXC1 | Q9P0U4 | 438.6832083 |
| 7208 | ZNF629 | Q9UEG4 | 438.4774884 |
| 7209 | NBEAL1 | Q6ZS30 | 438.3043992 |
| 7210 | CCDC85C | A6NKD9 | 437.6317222 |
| 7211 | TRAK1 | Q9UPV9 | 437.3383333 |
| 7212 | NOTCH4 | Q99466 | 437.0403333 |
| 7213 | STOML1 | Q9UBI4 | 435.8726667 |
| 7214 | CLCN6 | P51797 | 434.6084359 |
| 7215 | RC3H1 | Q5TC82 | 430.58444 |
| 7216 | GJA4 | P35212 | 430.5088333 |
| 7217 | VEZF1 | Q14119 | 425.1878889 |
| 7218 | OSBPL1A | Q9BXW6 | 424.7005741 |
| 7219 | MGA | Q8IWI9 | 424.286932 |
| 7220 | ZMYM3 | Q14202 | 424.0876786 |
| 7221 | ANKS1B | Q7Z6G8 | 423.2355932 |
| 7222 | PATZ1 | Q9HBE1 | 423.0899706 |
| 7223 | LRIG1 | Q96JA1 | 422.3105345 |
| 7224 | CLCN4 | P51793 | 420.9458846 |
| 7225 | CASC3 | O15234 | 418.4508519 |
| 7226 | HDAC10 | Q969S8 | 418.4417083 |
| 7227 | COX11 | Q9Y6N1 | 418.359 |
| 7228 | TRAF3 | Q13114 | 417.7411316 |
| 7229 | NCKIPSD | Q9NZQ3 | 416.9165667 |
| 7230 | PCLAF | Q15004 | 414.588 |
| 7231 | LARP1B | Q659C4 | 414.3835 |
| 7232 | TMEM231 | Q9H6L2 | 414.061 |
| 7233 | SAYSD1 | Q9NPB0 | 411.8141429 |
| 7234 | MYO5C | Q9NQX4 | 409.9601961 |
| 7235 | PAN2 | Q504Q3 | 409.7786863 |
| 7236 | RNPC3 | Q96LT9 | 409.0495652 |
| 7237 | CEP104 | O60308 | 408.1806604 |
| 7238 | TARBP1 | Q13395 | 406.7158736 |
| 7239 | RAB20 | Q9NX57 | 406.5392857 |
| 7240 | POLK | Q9UBT6 | 405.7867755 |
| 7241 | PIK3CB | P42338 | 405.2432982 |
| 7242 | MAPKAPK5 | Q8IW41 | 404.4746538 |
| 7243 | MBD1 | Q9UIS9 | 403.7266154 |
| 7244 | IGHA1 | P01876 | 403.5845333 |
| 7245 | SCYL3 | Q8IZE3 | 402.5811429 |
| 7246 | OSBPL5 | Q9H0X9 | 402.4734314 |
| 7247 | ZBTB16 | Q05516 | 400.818303 |
| 7248 | PLPP1 | O14494 | 400.6566 |
| 7249 | FAM193A | P78312 | 400.6440638 |
| 7250 | BCL9L | Q86UU0 | 398.9047 |
| 7251 | TNRC6C | Q9HCJ0 | 398.4394857 |
| 7252 | LRP1 | Q07954 | 398.20116 |
| 7253 | NKIRAS1 | Q9NYS0 | 398.1861538 |
| 7254 | BTBD19 | C9JJ37 | 397.6843333 |
| 7255 | JAG1 | P78504 | 397.3130727 |
| 7256 | NEK5 | Q6P3R8 | 397.1174857 |
| 7257 | SPAG5 | Q96R06 | 397.0504167 |
| 7258 | SLC35F6 | Q8N357 | 396.1691667 |
| 7259 | ANKRD13C | Q8N6S4 | 393.818 |
| 7260 | FIZ1 | Q96SL8 | 392.4260667 |
| 7261 | WDR59 | Q6PJI9 | 391.5168776 |
| 7262 | KCTD18 | Q6PI47 | 390.1003158 |
| 7263 | CDH4 | P55283 | 389.4228125 |
| 7264 | TWSG1 | Q9GZX9 | 387.2448889 |
| 7265 | ZBTB11 | O95625 | 386.6336047 |
| 7266 | SLC35B3 | Q9H1N7 | 386.4364615 |
| 7267 | CFAP54 | Q96N23 | 385.4340368 |
| 7268 | KRT82 | Q9NSB4 | 384.8090909 |
| 7269 | ZNF330 | Q9Y3S2 | 383.8529412 |
| 7270 | LYST | Q99698 | 382.4181006 |
| 7271 | PLEKHG1 | Q9ULL1 | 378.808806 |
| 7272 | EMCN | Q9ULC0 | 377.8834167 |
| 7273 | APOBEC3B | Q9UH17 | 377.6084091 |
| 7274 | KDM3A | Q9Y4C1 | 376.5244304 |
| 7275 | RLIM | Q9NVW2 | 374.3897667 |
| 7276 | TMEM120B | A0PK00 | 372.8107143 |
| 7277 | FRMD4B | Q9Y2L6 | 372.1736604 |
| 7278 | FRY | Q5TBA9 | 372.1051049 |
| 7279 | POLA2 | Q14181 | 370.8265652 |
| 7280 | BBS2 | Q9BXC9 | 370.4932727 |
| 7281 | DMXL2 | Q8TDJ6 | 369.0696889 |
| 7282 | AJUBA | Q96IF1 | 368.672 |
| 7283 | ZNF845 | Q96IR2 | 367.3592 |
| 7284 | CYB5R4 | Q7L1T6 | 367.1759697 |
| 7285 | ABCC10 | Q5T3U5 | 366.7537258 |
| 7286 | TRAF3IP1 | Q8TDR0 | 366.3713235 |
| 7287 | #N/A | P02534 | 365.5218214 |
| 7288 | RALGAPA1 | Q6GYQ0 | 365.2944176 |
| 7289 | ELK3 | P41970 | 363.285 |
| 7290 | PDSS1 | Q5T2R2 | 361.75825 |
| 7291 | ARRDC1 | Q8N5I2 | 361.5730769 |
| 7292 | ZNF532 | Q9HCE3 | 360.7476575 |
| 7293 | SMCR8 | Q8TEV9 | 359.0312292 |
| 7294 | ABCA2 | Q9BZC7 | 359.0240187 |
| 7295 | ADCY6 | O43306 | 356.1939318 |
| 7296 | NFATC4 | Q14934 | 354.9932143 |
| 7297 | HYAL2 | Q12891 | 354.9845 |
| 7298 | FAM111A | Q96PZ2 | 354.7753125 |
| 7299 | ABHD5 | Q8WTS1 | 353.8001875 |
| 7300 | SMPD2 | O60906 | 353.5282727 |
| 7301 | FOXP1 | Q9H334 | 353.1110769 |
| 7302 | C7 | P10643 | 351.5275 |
| 7303 | NFAT5 | O94916 | 351.3710909 |
| 7304 | PDE4D | Q08499 | 350.6934167 |
| 7305 | LMBR1 | Q8WVP7 | 350.3823615 |
| 7306 | GALNT11 | Q8NCW6 | 350.3246765 |
| 7307 | NRG1 | Q02297 | 349.8244483 |
| 7308 | RGS7 | P49802 | 349.6185652 |
| 7309 | AREL1 | O15033 | 349.5628205 |
| 7310 | MFSD8 | Q8NHS3 | 349.4845625 |
| 7311 | NOPCHAP1 | Q8N5I9 | 347.0108333 |
| 7312 | KLHL3 | Q9UH77 | 346.0889032 |
| 7313 | TCF20 | Q9UGU0 | 345.8369551 |
| 7314 | SGF29 | Q96ES7 | 344.6097273 |
| 7315 | INPP5E | Q9NRR6 | 344.1604722 |
| 7316 | CAPN15 | O75808 | 343.4108302 |
| 7317 | RAB36 | O95755 | 342.3752941 |
| 7318 | SLBP | Q14493 | 342.3660909 |
| 7319 | SIRT1 | Q96EB6 | 342.0885436 |
| 7320 | RPUSD3 | Q6P087 | 339.0037333 |
| 7321 | PRADC1 | Q9BSG0 | 338.8244 |
| 7322 | FARP2 | O94887 | 336.7803929 |
| 7323 | ALG14 | Q96F25 | 336.4451667 |
| 7324 | PLCL2 | Q9UPR0 | 336.0986 |
| 7325 | TMUB2 | Q71RG4 | 335.8661111 |
| 7326 | TNC | P24821 | 335.0370288 |
| 7327 | RRN3 | Q9NYV6 | 333.6080256 |
| 7328 | SSH2 | Q76I76 | 333.3592656 |
| 7329 | PDE2A | O00408 | 332.7403529 |
| 7330 | PAPOLG | Q9BWT3 | 331.5383171 |
| 7331 | BAG4 | O95429 | 331.2742857 |
| 7332 | CBX8 | Q9HC52 | 330.7621 |
| 7333 | PASK | Q96RG2 | 330.7062963 |
| 7334 | BRAF | P15056 | 327.1068065 |
| 7335 | FAM104A | Q969W3 | 326.9005 |
| 7336 | TRMT10A | Q8TBZ6 | 326.371 |
| 7337 | APOH | P02749 | 326.3505882 |
| 7338 | KDM2B | Q8NHM5 | 324.6094444 |
| 7339 | RAI1 | Q7Z5J4 | 323.3442976 |
| 7340 | FGD6 | Q6ZV73 | 319.6756946 |
| 7341 | GPBP1 | Q86WP2 | 318.5749167 |
| 7342 | COQ8A | Q8NI60 | 316.3824 |
| 7343 | SEMA4C | Q9C0C4 | 316.0911111 |
| 7344 | UNC5B | Q8IZJ1 | 315.2125349 |
| 7345 | RMDN2 | Q96LZ7 | 312.9787273 |
| 7346 | C5orf22 | Q49AR2 | 310.1511176 |
| 7347 | INO80 | Q9ULG1 | 308.7014595 |
| 7348 | CEP350 | Q5VT06 | 308.6188182 |
| 7349 | HIRIP3 | Q9BW71 | 307.4346429 |
| 7350 | DEPP1 | Q9NTK1 | 306.9019167 |
| 7351 | KCNAB2 | Q13303 | 305.743 |
| 7352 | STS | P08842 | 304.5876923 |
| 7353 | BANP | Q8N9N5 | 302.2623529 |
| 7354 | PHC1 | P78364 | 302.0431538 |
| 7355 | PIAS4 | Q8N2W9 | 301.8543043 |
| 7356 | HYLS1 | Q96M11 | 297.9177857 |
| 7357 | LENG1 | Q96BZ8 | 296.8271818 |
| 7358 | SPTB | P11277 | 295.4127907 |
| 7359 | PPP1R32 | Q7Z5V6 | 295.234 |
| 7360 | OAS2 | P29728 | 295.2169394 |
| 7361 | CBARP | Q8N350 | 294.9488667 |
| 7362 | KPNA5 | O15131 | 294.7802083 |
| 7363 | FAM157C | P0CG43 | 294.172 |
| 7364 | OFD1 | O75665 | 293.8936949 |
| 7365 | GID4 | Q8IVV7 | 293.4041429 |
| 7366 | TBP | P20226 | 293.2821111 |
| 7367 | ST3GAL2 | Q16842 | 291.5692778 |
| 7368 | SEPHS2 | Q99611 | 290.2083571 |
| 7369 | NAT9 | Q9BTE0 | 290.1725556 |
| 7370 | PIP | P12273 | 289.6216667 |
| 7371 | PLCG2 | P16885 | 287.5883971 |
| 7372 | CBX4 | O00257 | 286.8440769 |
| 7373 | ADPRH | P54922 | 284.0462778 |
| 7374 | SLC15A4 | Q8N697 | 283.8667 |
| 7375 | COL6A3 | P12111 | 283.5628353 |
| 7376 | ARMCX4 | Q5H9R4 | 283.5099065 |
| 7377 | MRPL52 | Q86TS9 | 282.6556 |
| 7378 | ZDHHC12 | Q96GR4 | 280.6803333 |
| 7379 | ARHGEF5 | Q12774 | 280.151 |
| 7380 | CEP63 | Q96MT8 | 278.9776053 |
| 7381 | GZF1 | Q9H116 | 278.7095 |
| 7382 | ENOSF1 | Q7L5Y1 | 278.6518077 |
| 7383 | THSD1 | Q9NS62 | 277.7236 |
| 7384 | NDST2 | P52849 | 276.6944681 |
| 7385 | SENP6 | Q9GZR1 | 275.6373962 |
| 7386 | DPYS | Q14117 | 275.5354167 |
| 7387 | RNF13 | O43567 | 274.6649091 |
| 7388 | SNX25 | Q9H3E2 | 273.2782885 |
| 7389 | GSDMA | Q96QA5 | 271.35192 |
| 7390 | NEBL | O76041 | 270.4843968 |
| 7391 | ABCA5 | Q8WWZ7 | 269.0367742 |
| 7392 | CENPT | Q96BT3 | 265.3184545 |
| 7393 | PLEKHG4 | Q58EX7 | 264.9226939 |
| 7394 | USP22 | Q9UPT9 | 264.1203826 |
| 7395 | SRRD | Q9UH36 | 263.9352667 |
| 7396 | APC | P25054 | 262.4369045 |
| 7397 | RFX7 | Q2KHR2 | 262.4230862 |
| 7398 | PPP2R3A | Q06190 | 262.2445455 |
| 7399 | PUS7L | Q9H0K6 | 258.2797021 |
| 7400 | DNMT3A | Q9Y6K1 | 258.1448148 |
| 7401 | ITPRIPL2 | Q3MIP1 | 257.7225882 |
| 7402 | MID1 | O15344 | 256.2203667 |
| 7403 | ADAT1 | Q9BUB4 | 254.6134643 |
| 7404 | AS3MT | Q9HBK9 | 254.5610769 |
| 7405 | PARP10 | Q53GL7 | 254.4508367 |
| 7406 | ARR3 | P36575 | 254.348125 |
| 7407 | MED13 | Q9UHV7 | 253.3607073 |
| 7408 | KANSL1 | Q7Z3B3 | 253.1597308 |
| 7409 | MTFMT | Q96DP5 | 253.10236 |
| 7410 | CAMKK1 | Q8N5S9 | 252.849069 |
| 7411 | VPS9D1 | Q9Y2B5 | 251.70975 |
| 7412 | C4orf33 | Q8N1A6 | 251.5855556 |
| 7413 | ZNF276 | Q8N554 | 250.445697 |
| 7414 | RTTN | Q86VV8 | 249.9913263 |
| 7415 | SNAPC3 | Q92966 | 248.3244348 |
| 7416 | SLC12A6 | Q9UHW9 | 245.067856 |
| 7417 | SCARA3 | Q6AZY7 | 242.6954286 |
| 7418 | IL13RA1 | P78552 | 242.5503 |
| 7419 | HMG20B | Q9P0W2 | 242.0806316 |
| 7420 | RGPD5 | Q99666 | 239.8183111 |
| 7421 | FAP | Q12884 | 238.5635581 |
| 7422 | MED28 | Q9H204 | 237.3588333 |
| 7423 | PYROXD1 | Q8WU10 | 234.6117368 |
| 7424 | ARHGAP23 | Q9P227 | 233.7025802 |
| 7425 | AGAP1 | Q9UPQ3 | 233.5342727 |
| 7426 | MLLT10 | P55197 | 233.2036923 |
| 7427 | NDOR1 | Q9UHB4 | 231.8284815 |
| 7428 | PAH | P00439 | 231.3710345 |
| 7429 | NINJ1 | Q92982 | 231.34425 |
| 7430 | IGKC | P01834 | 229.5508 |
| 7431 | FZD4 | Q9ULV1 | 228.0246842 |
| 7432 | OBSL1 | O75147 | 227.8078637 |
| 7433 | ST7 | Q9NRC1 | 227.5041724 |
| 7434 | MLX | Q9UH92 | 227.363 |
| 7435 | POLE2 | P56282 | 226.74724 |
| 7436 | RASAL3 | Q86YV0 | 226.2641053 |
| 7437 | DPP4 | P27487 | 224.6383077 |
| 7438 | IRF6 | O14896 | 224.405 |
| 7439 | NBR1 | Q14596 | 222.020697 |
| 7440 | C8orf37 | Q96NL8 | 220.7351538 |
| 7441 | LRRC32 | Q14392 | 218.989037 |
| 7442 | ZNF654 | Q8IZM8 | 218.5152745 |
| 7443 | TTC33 | Q6PID6 | 218.3895 |
| 7444 | DMWD | Q09019 | 216.7110345 |
| 7445 | FGD1 | P98174 | 213.9119111 |
| 7446 | DDHD1 | Q8NEL9 | 213.8779767 |
| 7447 | POLE | Q07864 | 213.3647477 |
| 7448 | DENND5B | Q6ZUT9 | 213.2256129 |
| 7449 | SLC8A1 | P32418 | 212.2771765 |
| 7450 | KRT4 | P19013 | 211.4127143 |
| 7451 | SLC35A1 | P78382 | 210.3286667 |
| 7452 | TCN2 | P20062 | 210.1486364 |
| 7453 | MTG2 | Q9H4K7 | 208.98425 |
| 7454 | ZP1 | P60852 | 208.2798571 |
| 7455 | TSR2 | Q969E8 | 208.23 |
| 7456 | ZNF516 | Q92618 | 207.1024242 |
| 7457 | HMCN2 | Q8NDA2 | 204.9414474 |
| 7458 | KCTD1 | Q719H9 | 203.751 |
| 7459 | GABBR2 | O75899 | 203.6032 |
| 7460 | BUB1 | O43683 | 202.4971923 |
| 7461 | TBPL2 | Q6SJ96 | 200.3018571 |
| 7462 | APLF | Q8IW19 | 200.1386364 |
| 7463 | PPIP5K1 | Q6PFW1 | 199.9853846 |
| 7464 | KAT2A | Q92830 | 199.6312778 |
| 7465 | SLC48A1 | Q6P1K1 | 199.4735 |
| 7466 | LIPF | P07098 | 198.7578571 |
| 7467 | RNASEH1 | O60930 | 198.6328571 |
| 7468 | TRIM62 | Q9BVG3 | 197.6330385 |
| 7469 | AFMID | Q63HM1 | 197.5657647 |
| 7470 | ADAT3 | Q96EY9 | 197.45365 |
| 7471 | LPXN | O60711 | 197.345375 |
| 7472 | NPR2 | P20594 | 196.8555965 |
| 7473 | CCNL1 | Q9UK58 | 196.836619 |
| 7474 | RNF130 | Q86XS8 | 195.3364762 |
| 7475 | TRMT2B | Q96GJ1 | 194.9006071 |
| 7476 | L3MBTL2 | Q969R5 | 194.0983103 |
| 7477 | RPUSD4 | Q96CM3 | 193.9012 |
| 7478 | TMEM44 | Q2T9K0 | 193.6703478 |
| 7479 | FLG2 | Q5D862 | 192.9876197 |
| 7480 | ANKRD11 | Q6UB99 | 192.3530865 |
| 7481 | ECT2 | Q9H8V3 | 191.6895714 |
| 7482 | VCAM1 | P19320 | 191.3782381 |
| 7483 | PCGF2 | P35227 | 191.1400667 |
| 7484 | SLC19A1 | P41440 | 190.965125 |
| 7485 | CWF19L2 | Q2TBE0 | 189.52292 |
| 7486 | ZBTB45 | Q96K62 | 187.8235263 |
| 7487 | FAM185A | Q8N0U4 | 187.2785909 |
| 7488 | PRG2 | P13727 | 187.17 |
| 7489 | NCAPD3 | P42695 | 186.9692286 |
| 7490 | FBXW11 | Q9UKB1 | 186.5173636 |
| 7491 | SYVN1 | Q86TM6 | 184.99 |
| 7492 | BACH1 | O14867 | 184.6423 |
| 7493 | NHLRC3 | Q5JS37 | 182.3310714 |
| 7494 | GTF2IRD1 | Q9UHL9 | 181.48512 |
| 7495 | RCCD1 | A6NED2 | 180.9342 |
| 7496 | KIAA0586 | Q9BVV6 | 180.7660896 |
| 7497 | KDM1B | Q8NB78 | 180.6587778 |
| 7498 | TOPBP1 | Q92547 | 180.4675974 |
| 7499 | MTO1 | Q9Y2Z2 | 180.4413659 |
| 7500 | RAD54L2 | Q9Y4B4 | 180.1443824 |
| 7501 | MEGF8 | Q7Z7M0 | 179.4247054 |
| 7502 | SENP5 | Q96HI0 | 179.3918 |
| 7503 | NLRP13 | Q86W25 | 178.4184615 |
| 7504 | TMEM260 | Q9NX78 | 177.7688485 |
| 7505 | CERK | Q8TCT0 | 177.49275 |
| 7506 | IFI27 | P40305 | 177.28675 |
| 7507 | SLC23A2 | Q9UGH3 | 175.5145 |
| 7508 | MIB2 | Q96AX9 | 175.50886 |
| 7509 | METAP1D | Q6UB28 | 175.4754615 |
| 7510 | ZNRF1 | Q8ND25 | 175.3333571 |
| 7511 | CLUAP1 | Q96AJ1 | 173.812381 |
| 7512 | ZBTB20 | Q9HC78 | 173.7837742 |
| 7513 | TTN | Q8WZ42 | 173.508289 |
| 7514 | BBS1 | Q8NFJ9 | 172.3595758 |
| 7515 | DNAH12 | Q6ZR08 | 172.1795235 |
| 7516 | SERAC1 | Q96JX3 | 171.8281111 |
| 7517 | ISG20 | Q96AZ6 | 171.4743 |
| 7518 | ACVR1B | P36896 | 171.3228095 |
| 7519 | GPT2 | Q8TD30 | 170.6541923 |
| 7520 | ZC2HC1A | Q96GY0 | 169.6309091 |
| 7521 | LDB2 | O43679 | 169.3725333 |
| 7522 | CAMK4 | Q16566 | 168.4937667 |
| 7523 | PDE7B | Q9NP56 | 167.42464 |
| 7524 | ICOSLG | O75144 | 167.4089167 |
| 7525 | KAT8 | Q9H7Z6 | 165.13832 |
| 7526 | NEB | P20929 | 164.8317005 |
| 7527 | SDK1 | Q7Z5N4 | 164.1944186 |
| 7528 | CHKA | P35790 | 163.8469545 |
| 7529 | ZNF280C | Q8ND82 | 163.7754878 |
| 7530 | GNA14 | O95837 | 163.7323333 |
| 7531 | CENPE | Q02224 | 163.3928383 |
| 7532 | DTNA | Q9Y4J8 | 163.1338095 |
| 7533 | PEX12 | O00623 | 163.0173529 |
| 7534 | NEURL4 | Q96JN8 | 162.0541607 |
| 7535 | PBX1 | P40424 | 161.7431875 |
| 7536 | HDAC4 | P56524 | 161.0934314 |
| 7537 | TAF1 | P21675 | 160.042557 |
| 7538 | DNAAF2 | Q9NVR5 | 159.28148 |
| 7539 | COQ5 | Q5HYK3 | 158.9742727 |
| 7540 | FBXL12 | Q9NXK8 | 158.5805714 |
| 7541 | PTPRS | Q13332 | 158.2925341 |
| 7542 | BICRA | Q9NZM4 | 157.4272564 |
| 7543 | ADAMTS7 | Q9UKP4 | 156.3865965 |
| 7544 | MLLT6 | P55198 | 155.6336897 |
| 7545 | USP42 | Q9H9J4 | 155.3211509 |
| 7546 | KLHL7 | Q8IXQ5 | 154.9877353 |
| 7547 | MAPKBP1 | O60336 | 154.8503521 |
| 7548 | TMEM267 | Q0VDI3 | 154.2808182 |
| 7549 | TUBA3E | Q6PEY2 | 153.5809091 |
| 7550 | SLC36A4 | Q6YBV0 | 153.54525 |
| 7551 | ASF1B | Q9NVP2 | 153.3107778 |
| 7552 | ABAT | P80404 | 151.1066667 |
| 7553 | DPY19L3 | Q6ZPD9 | 151.0802692 |
| 7554 | DVL3 | Q92997 | 150.5681786 |
| 7555 | IMPG2 | Q9BZV3 | 149.8720455 |
| 7556 | FAM184B | Q9ULE4 | 147.9142029 |
| 7557 | LRP6 | O75581 | 147.2092632 |
| 7558 | TWNK | Q96RR1 | 145.6535294 |
| 7559 | GPAM | Q9HCL2 | 144.8681538 |
| 7560 | SPPL2A | Q8TCT8 | 144.2411111 |
| 7561 | SDC3 | O75056 | 144.1602769 |
| 7562 | USP26 | Q9BXU7 | 143.5432609 |
| 7563 | RNF41 | Q9H4P4 | 143.389 |
| 7564 | TMEM87B | Q96K49 | 142.58812 |
| 7565 | SAAL1 | Q96ER3 | 141.271344 |
| 7566 | AZGP1 | P25311 | 139.6488889 |
| 7567 | HRH1 | P35367 | 139.5858636 |
| 7568 | GC | P02774 | 139.1847143 |
| 7569 | TGM1 | P22735 | 135.7983778 |
| 7570 | R3HCC1L | Q7Z5L2 | 135.0487771 |
| 7571 | C2orf69 | Q8N8R5 | 134.5757727 |
| 7572 | MED30 | Q96HR3 | 133.689 |
| 7573 | MFSD14A | Q96MC6 | 133.4140833 |
| 7574 | HMCES | Q96FZ2 | 132.7325652 |
| 7575 | CENPF | P49454 | 131.0162073 |
| 7576 | CHTF18 | Q8WVB6 | 130.8696279 |
| 7577 | FBXL6 | Q8N531 | 130.6541154 |
| 7578 | RNF141 | Q8WVD5 | 129.8322222 |
| 7579 | TTC21A | Q8NDW8 | 129.6 |
| 7580 | GPRIN1 | Q7Z2K8 | 129.2706494 |
| 7581 | CUL9 | Q8IWT3 | 129.1982742 |
| 7582 | SESN1 | Q9Y6P5 | 128.8297692 |
| 7583 | POGK | Q9P215 | 127.6611185 |
| 7584 | QSER1 | Q2KHR3 | 126.9067937 |
| 7585 | DYNC2H1 | Q8NCM8 | 125.9592954 |
| 7586 | NPRL3 | Q12980 | 125.24312 |
| 7587 | KLHL26 | Q53HC5 | 124.4762432 |
| 7588 | JADE1 | Q6IE81 | 123.77674 |
| 7589 | PLA2R1 | Q13018 | 123.1452931 |
| 7590 | ECM1 | Q16610 | 122.4823667 |
| 7591 | ZNF827 | Q17R98 | 122.4449831 |
| 7592 | KDM5A | P29375 | 118.4732386 |
| 7593 | GPATCH1 | Q9BRR8 | 118.2872857 |
| 7594 | TTK | P33981 | 117.4211304 |
| 7595 | NRK | Q7Z2Y5 | 116.6743506 |
| 7596 | ATP6V0D2 | Q8N8Y2 | 115.89 |
| 7597 | LIPE | Q05469 | 115.7881228 |
| 7598 | RPS6KA5 | O75582 | 115.3885349 |
| 7599 | TSEN54 | Q7Z6J9 | 114.8787619 |
| 7600 | GON4L | Q3T8J9 | 114.58 |
| 7601 | ZNHIT6 | Q9NWK9 | 114.3514375 |
| 7602 | FBXO44 | Q9H4M3 | 113.0189333 |
| 7603 | PNMA6A | P0CW24 | 112.1369565 |
| 7604 | KIAA0232 | Q92628 | 110.8728125 |
| 7605 | NOVA1 | P51513 | 110.831 |
| 7606 | SOS2 | Q07890 | 110.3768533 |
| 7607 | ST3GAL1 | Q11201 | 110.3209444 |
| 7608 | LZTS2 | Q9BRK4 | 110.0184571 |
| 7609 | NSD1 | Q96L73 | 109.2394179 |
| 7610 | ZNF574 | Q6ZN55 | 109.0066774 |
| 7611 | TCTN2 | Q96GX1 | 108.7626563 |
| 7612 | CEP112 | Q8N8E3 | 108.2347222 |
| 7613 | KNL1 | Q8NG31 | 107.8816557 |
| 7614 | SLC25A21 | Q9BQT8 | 107.4421053 |
| 7615 | ZC3H12A | Q5D1E8 | 106.991 |
| 7616 | GANC | Q8TET4 | 106.932449 |
| 7617 | WDR73 | Q6P4I2 | 106.4494348 |
| 7618 | NCR3LG1 | Q68D85 | 104.4543913 |
| 7619 | LDB1 | Q86U70 | 104.2125 |
| 7620 | TREX1 | Q9NSU2 | 104.1505625 |
| 7621 | CDK19 | Q9BWU1 | 103.9388636 |
| 7622 | CHST12 | Q9NRB3 | 103.5697895 |
| 7623 | RAB11FIP3 | O75154 | 103.4193793 |
| 7624 | TATDN2 | Q93075 | 103.0367436 |
| 7625 | KIF18B | Q86Y91 | 102.6361364 |
| 7626 | DISC1 | Q9NRI5 | 102.298 |
| 7627 | MED8 | Q96G25 | 102.2105714 |
| 7628 | NCOA2 | Q15596 | 101.5946111 |
| 7629 | CNTLN | Q9NXG0 | 100.9092 |
| 7630 | ARID4A | P29374 | 100.7033333 |
| 7631 | SHOC1 | Q5VXU9 | 98.8412987 |
| 7632 | PIK3CD | O00329 | 98.73964912 |
| 7633 | NFATC3 | Q12968 | 98.35469697 |
| 7634 | ALOX12B | O75342 | 98.1215 |
| 7635 | KRT23 | Q9C075 | 97.84185185 |
| 7636 | GATAD1 | Q8WUU5 | 97.45230769 |
| 7637 | ABCA6 | Q8N139 | 96.48692188 |
| 7638 | TPGS1 | Q6ZTW0 | 96.07116667 |
| 7639 | MALSU1 | Q96EH3 | 96.03692308 |
| 7640 | PDE10A | Q9Y233 | 96.00518868 |
| 7641 | FAM83D | Q9H4H8 | 95.4179 |
| 7642 | ROR1 | Q01973 | 94.94658974 |
| 7643 | UBOX5 | O94941 | 94.61632 |
| 7644 | DMD | P11532 | 92.96753234 |
| 7645 | SNAPC4 | Q5SXM2 | 92.63366176 |
| 7646 | SECISBP2 | Q96T21 | 90.34722571 |
| 7647 | TRAF1 | Q13077 | 89.0272381 |
| 7648 | SUCO | Q9UBS9 | 88.12407692 |
| 7649 | CEP76 | Q8TAP6 | 88.12351667 |
| 7650 | SH3TC2 | Q8TF17 | 86.32180556 |
| 7651 | ASXL2 | Q76L83 | 85.99343662 |
| 7652 | FBRS | Q9HAH7 | 84.31375 |
| 7653 | ADCK5 | Q3MIX3 | 84.09567647 |
| 7654 | SUPT7L | O94864 | 82.69003333 |
| 7655 | MAGED4 | Q96JG8 | 82.37928125 |
| 7656 | DSC1 | Q08554 | 82.11095556 |
| 7657 | USP9Y | O00507 | 81.9345 |
| 7658 | CTSO | P43234 | 80.50523077 |
| 7659 | MYO7A | Q13402 | 80.4539633 |
| 7660 | PSRC1 | Q6PGN9 | 80.12478261 |
| 7661 | CUEDC1 | Q9NWM3 | 79.64345455 |
| 7662 | SGPP1 | Q9BX95 | 79.13294118 |
| 7663 | PRR12 | Q9ULL5 | 79.121375 |
| 7664 | KDM5D | Q9BY66 | 78.9047561 |
| 7665 | FNIP2 | Q9P278 | 76.72672131 |
| 7666 | ADAM12 | O43184 | 76.52595238 |
| 7667 | RTN2 | O75298 | 73.43481481 |
| 7668 | B3GNT2 | Q9NY97 | 72.87043478 |
| 7669 | TANC2 | Q9HCD6 | 72.03221176 |
| 7670 | PTGS2 | P35354 | 71.312 |
| 7671 | ZFP90 | Q8TF47 | 70.4421875 |
| 7672 | KRT33B | Q14525 | 69.82646154 |
| 7673 | HPDL | Q96IR7 | 68.68166667 |
| 7674 | STK19 | P49842 | 68.50093333 |
| 7675 | SLC25A35 | Q3KQZ1 | 67.78735 |
| 7676 | UROC1 | Q96N76 | 67.6882 |
| 7677 | STAP2 | Q9UGK3 | 67.50090909 |
| 7678 | VEGFC | P49767 | 65.87388462 |
| 7679 | CHST7 | Q9NS84 | 65.51192857 |
| 7680 | TUBB8 | Q3ZCM7 | 65.13854 |
| 7681 | RPS4Y2 | Q8TD47 | 64.398875 |
| 7682 | REST | Q13127 | 64.13342222 |
| 7683 | TEX14 | Q8IWB6 | 63.95527027 |
| 7684 | KLHL8 | Q9P2G9 | 62.887075 |
| 7685 | FAM200B | P0CF97 | 62.55666667 |
| 7686 | NSL1 | Q96IY1 | 62.3945 |
| 7687 | CLSPN | Q9HAW4 | 61.37162712 |
| 7688 | FAM111B | Q6SJ93 | 61.3232093 |
| 7689 | RLF | Q13129 | 60.58031395 |
| 7690 | SREBF2 | Q12772 | 60.46584783 |
| 7691 | RC3H2 | Q9HBD1 | 60.23766071 |
| 7692 | SGSM3 | Q96HU1 | 60.10535897 |
| 7693 | KATNIP | O60303 | 59.80157895 |
| 7694 | ZFPM2 | Q8WW38 | 58.61414426 |
| 7695 | MAP3K2 | Q9Y2U5 | 58.44943333 |
| 7696 | BMI1 | P35226 | 58.06439286 |
| 7697 | GMNN | O75496 | 57.25885714 |
| 7698 | DTX2 | Q86UW9 | 56.2636087 |
| 7699 | SZT2 | Q5T011 | 56.10765734 |
| 7700 | ECE2 | P0DPD6 | 56.03762791 |
| 7701 | DPAGT1 | Q9H3H5 | 54.78676923 |
| 7702 | ZFHX4 | Q86UP3 | 54.38566883 |
| 7703 | CAPRIN2 | Q6IMN6 | 54.00336364 |
| 7704 | RCBTB2 | O95199 | 53.03138889 |
| 7705 | ARL14EP | Q8N8R7 | 52.54592308 |
| 7706 | PWWP2A | Q96N64 | 52.50144242 |
| 7707 | GPRIN3 | Q6ZVF9 | 51.3173 |
| 7708 | ZBTB4 | Q9P1Z0 | 50.34034375 |
| 7709 | ZFYVE9 | O95405 | 49.71161818 |
| 7710 | HMBOX1 | Q6NT76 | 49.07188235 |
| 7711 | CKAP2L | Q8IYA6 | 47.71459524 |
| 7712 | DYNC1I1 | O14576 | 47.65663636 |
| 7713 | CCNA2 | P20248 | 47.50063636 |
| 7714 | NPDC1 | Q9NQX5 | 47.123875 |
| 7715 | DSEL | Q8IZU8 | 47.04117647 |
| 7716 | MED13L | Q71F56 | 45.85702381 |
| 7717 | FER1L5 | A0AVI2 | 45.52235849 |
| 7718 | SCAPER | Q9BY12 | 45.35434848 |
| 7719 | SP140L | Q9H930 | 45.304825 |
| 7720 | SKP2 | Q13309 | 43.52595652 |
| 7721 | CCDC126 | Q96EE4 | 42.495875 |
| 7722 | FLG | P20930 | 42.17094203 |
| 7723 | ABCG2 | Q9UNQ0 | 41.49564286 |
| 7724 | TOP1MT | Q969P6 | 41.24441176 |
| 7725 | RASSF7 | Q02833 | 40.6486 |
| 7726 | RNF216 | Q9NWF9 | 40.58163415 |
| 7727 | TRIM37 | O94972 | 40.19626829 |
| 7728 | LCORL | Q8N3X6 | 39.26764286 |
| 7729 | HGD | Q93099 | 39.0357619 |
| 7730 | PRKCQ | Q04759 | 38.89692308 |
| 7731 | TRPM3 | Q9HCF6 | 37.62084615 |
| 7732 | NFX1 | Q12986 | 37.45332787 |
| 7733 | GNPTAB | Q3T906 | 37.2628125 |
| 7734 | MAP3K6 | O95382 | 37.00110417 |
| 7735 | FASTKD1 | Q53R41 | 36.88723256 |
| 7736 | FCSK | Q8N0W3 | 36.79186047 |
| 7737 | MASTL | Q96GX5 | 36.2387561 |
| 7738 | CFAP97 | Q9P2B7 | 35.69177778 |
| 7739 | CSF1 | P09603 | 35.67608696 |
| 7740 | POMT2 | Q9UKY4 | 35.51369444 |
| 7741 | R3HDM2 | Q9Y2K5 | 35.35188 |
| 7742 | KIAA1109 | Q2LD37 | 35.15649784 |
| 7743 | UBN2 | Q6ZU65 | 34.8295082 |
| 7744 | TFDP2 | Q14188 | 34.16106667 |
| 7745 | CDC45 | O75419 | 34.10057692 |
| 7746 | AXIN1 | O15169 | 34.06517073 |
| 7747 | FAHD2B | Q6P2I3 | 33.4613125 |
| 7748 | TBX18 | O95935 | 31.16610714 |
| 7749 | TMEM67 | Q5HYA8 | 30.39613333 |
| 7750 | HLA-F | P30511 | 30.29811111 |
| 7751 | BAZ2B | Q9UIF8 | 29.81769231 |
| 7752 | AFF1 | P51825 | 29.77959184 |
| 7753 | FHOD3 | Q2V2M9 | 29.50080303 |
| 7754 | TMEM135 | Q86UB9 | 29.45596154 |
| 7755 | USP6NL | Q92738 | 29.181 |
| 7756 | PGK2 | P07205 | 28.58892308 |
| 7757 | SP110 | Q9HB58 | 28.46669697 |
| 7758 | BUB1B | O60566 | 28.43232787 |
| 7759 | PCDH9 | Q9HC56 | 28.19482143 |
| 7760 | USP1 | O94782 | 26.77192683 |
| 7761 | CDCA2 | Q69YH5 | 26.6055625 |
| 7762 | TENT5A | Q96IP4 | 26.0018125 |
| 7763 | RARA | P10276 | 25.05522222 |
| 7764 | DTNB | O60941 | 23.7873125 |
| 7765 | ZNF217 | O75362 | 23.6812807 |
| 7766 | DPH1 | Q9BZG8 | 23.40188 |
| 7767 | ACAD10 | Q6JQN1 | 22.39657627 |
| 7768 | INPP5B | P32019 | 22.25823077 |
| 7769 | PPP1R3G | B7ZBB8 | 21.22329412 |
| 7770 | CFAP44 | Q96MT7 | 20.92642857 |
| 7771 | TBC1D12 | O60347 | 20.87905128 |
| 7772 | PEX10 | O60683 | 20.06442857 |
| 7773 | AOX1 | Q06278 | 19.14388406 |
| 7774 | PAQR7 | Q86WK9 | 18.769 |
| 7775 | PHF12 | Q96QT6 | 18.07515789 |
| 7776 | BEND3 | Q5T5X7 | 16.66504167 |
| 7777 | TRAK2 | O60296 | 16.6452439 |
| 7778 | FBXL15 | Q9H469 | 16.600625 |
| 7779 | AGGF1 | Q8N302 | 16.51831034 |
| 7780 | RECQL5 | O94762 | 16.49288596 |
| 7781 | TIMELESS | Q9UNS1 | 15.68715789 |
| 7782 | XPR1 | Q9UBH6 | 15.45569697 |
| 7783 | PARD3B | Q8TEW8 | 15.08523529 |
| 7784 | ZC3H6 | P61129 | 14.64034146 |
| 7785 | RAB11FIP2 | Q7L804 | 14.52071429 |
| 7786 | DOT1L | Q8TEK3 | 14.2727027 |
| 7787 | DEPDC5 | O75140 | 14.25758974 |
| 7788 | FANCD2 | Q9BXW9 | 14.22612162 |
| 7789 | BRD9 | Q9H8M2 | 13.68838462 |
| 7790 | TTC7A | Q9ULT0 | 13.63129787 |
| 7791 | CEP120 | Q8N960 | 13.19242857 |
| 7792 | PRR14L | Q5THK1 | 12.42520661 |
| 7793 | ZNF292 | O60281 | 11.67617829 |
| 7794 | N4BP2 | Q86UW6 | 10.23453846 |
| 7795 | ADGRG6 | Q86SQ4 | 9.930529412 |
| 7796 | ATAD2B | Q9ULI0 | 7.646041096 |
| 7797 | AR | P10275 | 7.245108108 |
| 7798 | ARHGAP45 | Q92619 | 7.078232558 |
| 7799 | EDRF1 | Q3B7T1 | 6.998875 |
| 7800 | SGO2 | Q562F6 | 6.915197183 |
| 7801 | CCP110 | O43303 | 6.415653846 |
| 7802 | KIF16B | Q96L93 | 5.96272 |
| 7803 | CRACD | Q6ZU35 | 5.857612903 |
| 7804 | PRIMPOL | Q96LW4 | 5.690392857 |
| 7805 | RAPGEF3 | O95398 | 5.105125 |
| 7806 | WEE1 | P30291 | 4.939090909 |
| 7807 | HRNR | Q86YZ3 | 3.200403509 |
| 7808 | PLEKHA8 | Q96JA3 | 3.047509677 |
| 7809 | TNXB | P22105 | 2.939069767 |
| 7810 | TBC1D1 | Q86TI0 | 2.59331746 |
| 7811 | TNRC18 | O15417 | 2.413295082 |

**Table S2**. Significantly differentially abundant proteins in hypoxic HCAECs compared to 20% O_2_

| **Uniprot** | **Gene name** | **Protein name** | **Log2 fold change** | **Adj. P-value** |
| --- | --- | --- | --- | --- |
| Q16647 | PTGIS | Prostacyclin synthase (EC 5.3.99.4) (Hydroperoxy icosatetraenoate dehydratase) (EC 4.2.1.152) (Prostaglandin I2 synthase) | 2.80 | 1.88E-09 |
| Q12882 | DPYD | Dihydropyrimidine dehydrogenase [NADP(+)] (DHPDHase) (DPD) (EC 1.3.1.2) (Dihydrothymine dehydrogenase) (Dihydrouracil dehydrogenase) | 2.62 | 2.41E-17 |
| P11166 | SLC2A1 | Solute carrier family 2, facilitated glucose transporter member 1 (Glucose transporter type 1, erythrocyte/brain) (GLUT-1) (HepG2 glucose transporter) | 2.46 | 4.26E-12 |
| P78540 | ARG2 | Arginase-2, mitochondrial (EC 3.5.3.1) (Arginase II) (Kidney-type arginase) (Non-hepatic arginase) (Type II arginase) | 2.33 | 3.09E-10 |
| P28300 | LOX | Protein-lysine 6-oxidase (EC 1.4.3.13) (Lysyl oxidase) [Cleaved into: Protein-lysine 6-oxidase, long form; Protein-lysine 6-oxidase, short form] | 2.15 | 8.36E-16 |
| P98095 | FBLN2 | Fibulin-2 (FIBL-2) | 1.97 | 3.34E-04 |
| Q13541 | EIF4EBP1 | Eukaryotic translation initiation factor 4E-binding protein 1 (4E-BP1) (eIF4E-binding protein 1) (Phosphorylated heat- and acid-stable protein regulated by insulin 1) (PHAS-I) | 1.93 | 2.00E-08 |
| Q9BY76 | ANGPTL4 | Angiopoietin-related protein 4 (Angiopoietin-like protein 4) (Hepatic fibrinogen/angiopoietin-related protein) (HFARP) [Cleaved into: ANGPTL4 N-terminal chain; ANGPTL4 C-terminal chain] | 1.93 | 4.26E-12 |
| P02794 | FTH1 | Ferritin heavy chain (Ferritin H subunit) (EC 1.16.3.1) (Cell proliferation-inducing gene 15 protein) [Cleaved into: Ferritin heavy chain, N-terminally processed] | 1.89 | 1.82E-11 |
| P27144 | AK4 | Adenylate kinase 4, mitochondrial (AK 4) (EC 2.7.4.10) (EC 2.7.4.6) (Adenylate kinase 3-like) (GTP:AMP phosphotransferase AK4) | 1.88 | 1.34E-12 |
| Q9H4B7 | TUBB1 | Tubulin beta-1 chain | 1.86 | 2.65E-04 |
| P02792 | FTL | Ferritin light chain (Ferritin L subunit) | 1.83 | 1.50E-08 |
| P0CG12 | DERPC | Decreased expression in renal and prostate cancer protein | 1.81 | 5.42E-03 |
| P04114 | APOB | Apolipoprotein B-100 (Apo B-100) [Cleaved into: Apolipoprotein B-48 (Apo B-48)] | 1.78 | 3.34E-11 |
| P19971 | TYMP | Thymidine phosphorylase (TP) (EC 2.4.2.4) (Gliostatin) (Platelet-derived endothelial cell growth factor) (PD-ECGF) (TdRPase) | 1.77 | 2.16E-15 |
| P18859 | ATP5PF | ATP synthase-coupling factor 6, mitochondrial (ATPase subunit F6) (ATP synthase peripheral stalk subunit F6) | 1.72 | 8.52E-11 |
| P26232 | CTNNA2 | Catenin alpha-2 (Alpha N-catenin) (Alpha-catenin-related protein) | 1.69 | 3.39E-02 |
| Q15493 | RGN | Regucalcin (RC) (Gluconolactonase) (GNL) (EC 3.1.1.17) (Senescence marker protein 30) (SMP-30) | 1.65 | 1.36E-08 |
| Q12983 | BNIP3 | BCL2/adenovirus E1B 19 kDa protein-interacting protein 3 | 1.62 | 5.71E-03 |
| P01023 | A2M | Alpha-2-macroglobulin (Alpha-2-M) (C3 and PZP-like alpha-2-macroglobulin domain-containing protein 5) | 1.62 | 2.54E-11 |
| Q9H5V8 | CDCP1 | CUB domain-containing protein 1 (Membrane glycoprotein gp140) (Subtractive immunization M plus HEp3-associated 135 kDa protein) (SIMA135) (Transmembrane and associated with src kinases) (CD antigen CD318) | 1.50 | 1.10E-02 |
| Q15847 | ADIRF | Adipogenesis regulatory factor (Adipogenesis factor rich in obesity) (Adipose most abundant gene transcript 2 protein) (Adipose-specific protein 2) (apM-2) | 1.49 | 3.59E-05 |
| P09104 | ENO2 | Gamma-enolase (EC 4.2.1.11) (2-phospho-D-glycerate hydro-lyase) (Enolase 2) (Neural enolase) (Neuron-specific enolase) (NSE) | 1.47 | 9.31E-17 |
| P62328 | TMSB4X | Thymosin beta-4 (T beta-4) (Fx) [Cleaved into: Hemoregulatory peptide AcSDKP (Ac-Ser-Asp-Lys-Pro) (N-acetyl-SDKP) (AcSDKP) (Seraspenide)] | 1.44 | 3.92E-06 |
| P61812 | TGFB2 | Transforming growth factor beta-2 proprotein (Cetermin) (Glioblastoma-derived T-cell suppressor factor) (G-TSF) [Cleaved into: Latency-associated peptide (LAP); Transforming growth factor beta-2 (TGF-beta-2)] | 1.41 | 3.68E-10 |
| Q9H2T7 | RANBP17 | Ran-binding protein 17 | 1.41 | 2.02E-03 |
| P00749 | PLAU | Urokinase-type plasminogen activator (U-plasminogen activator) (uPA) (EC 3.4.21.73) [Cleaved into: Urokinase-type plasminogen activator long chain A; Urokinase-type plasminogen activator short chain A; Urokinase-type plasminogen activator chain B] | 1.40 | 2.50E-09 |
| P01008 | SERPINC1 | Antithrombin-III (ATIII) (Serpin C1) | 1.40 | 8.82E-10 |
| P09467 | FBP1 | Fructose-1,6-bisphosphatase 1 (FBPase 1) (EC 3.1.3.11) (D-fructose-1,6-bisphosphate 1-phosphohydrolase 1) (Liver FBPase) | 1.39 | 4.51E-06 |
| Q6KF10 | GDF6 | Growth/differentiation factor 6 (GDF-6) (Bone morphogenetic protein 13) (BMP-13) (Growth/differentiation factor 16) | 1.36 | 4.21E-06 |
| P01031 | C5 | Complement C5 (C3 and PZP-like alpha-2-macroglobulin domain-containing protein 4) [Cleaved into: Complement C5 beta chain; Complement C5 alpha chain; C5a anaphylatoxin; Complement C5 alpha' chain] | 1.35 | 6.82E-03 |
| Q6NZY7 | CDC42EP5 | Cdc42 effector protein 5 (Binder of Rho GTPases 3) | 1.35 | 3.51E-04 |
| P36871 | PGM1 | Phosphoglucomutase-1 (PGM 1) (EC 5.4.2.2) (Glucose phosphomutase 1) | 1.33 | 8.09E-14 |
| O95295 | SNAPIN | SNARE-associated protein Snapin (Biogenesis of lysosome-related organelles complex 1 subunit 7) (BLOC-1 subunit 7) (Synaptosomal-associated protein 25-binding protein) (SNAP-associated protein) | 1.31 | 2.00E-08 |
| P05362 | ICAM1 | Intercellular adhesion molecule 1 (ICAM-1) (Major group rhinovirus receptor) (CD antigen CD54) | 1.31 | 1.34E-12 |
| O43175 | PHGDH | D-3-phosphoglycerate dehydrogenase (3-PGDH) (EC 1.1.1.95) (2-oxoglutarate reductase) (EC 1.1.1.399) (Malate dehydrogenase) (EC 1.1.1.37) | 1.30 | 1.47E-12 |
| O43854 | EDIL3 | EGF-like repeat and discoidin I-like domain-containing protein 3 (Developmentally-regulated endothelial cell locus 1 protein) (Integrin-binding protein DEL1) | 1.29 | 5.64E-13 |
| Q9UFG5 | C19orf25 | UPF0449 protein C19orf25 | 1.29 | 4.72E-05 |
| O00115 | DNASE2 | Deoxyribonuclease-2-alpha (EC 3.1.22.1) (Acid DNase) (Deoxyribonuclease II alpha) (DNase II alpha) (Lysosomal DNase II) (R31240_2) | 1.29 | 7.56E-09 |
| Q96JY6 | PDLIM2 | PDZ and LIM domain protein 2 (PDZ-LIM protein mystique) | 1.28 | 2.58E-09 |
| P00742 | F10 | Coagulation factor X (EC 3.4.21.6) (Stuart factor) (Stuart-Prower factor) [Cleaved into: Factor X light chain; Factor X heavy chain; Activated factor Xa heavy chain] | 1.28 | 6.07E-08 |
| Q8N142 | ADSS1 | Adenylosuccinate synthetase isozyme 1 (AMPSase 1) (AdSS 1) (EC 6.3.4.4) (Adenylosuccinate synthetase, basic isozyme) (Adenylosuccinate synthetase, muscle isozyme) (M-type adenylosuccinate synthetase) (Adenylosuccinate synthetase-like 1) (AdSSL1) (IMP--aspartate ligase 1) | 1.28 | 3.32E-03 |
| Q5T013 | HYI | Putative hydroxypyruvate isomerase (EC 5.3.1.22) (Endothelial cell apoptosis protein E-CE1) | 1.28 | 6.67E-12 |
| Q06033 | ITIH3 | Inter-alpha-trypsin inhibitor heavy chain H3 (ITI heavy chain H3) (ITI-HC3) (Inter-alpha-inhibitor heavy chain 3) (Serum-derived hyaluronan-associated protein) (SHAP) | 1.27 | 2.59E-07 |
| Q9NPH3 | IL1RAP | Interleukin-1 receptor accessory protein (IL-1 receptor accessory protein) (IL-1RAcP) (EC 3.2.2.6) (Interleukin-1 receptor 3) (IL-1R-3) (IL-1R3) | 1.24 | 1.82E-09 |
| P63313 | TMSB10 | Thymosin beta-10 | 1.24 | 7.51E-08 |
| P69905 | HBA1 | Hemoglobin subunit alpha (Alpha-globin) (Hemoglobin alpha chain) [Cleaved into: Hemopressin] | 1.23 | 2.20E-03 |
| Q5EB52 | MEST | Mesoderm-specific transcript homolog protein (EC 3.-.-.-) (Paternally-expressed gene 1 protein) | 1.21 | 9.92E-04 |
| Q9H2A2 | ALDH8A1 | 2-aminomuconic semialdehyde dehydrogenase (EC 1.2.1.32) (Aldehyde dehydrogenase 12) (Aldehyde dehydrogenase family 8 member A1) | 1.20 | 1.22E-03 |
| P00966 | ASS1 | Argininosuccinate synthase (EC 6.3.4.5) (Citrulline--aspartate ligase) | 1.20 | 1.30E-06 |
| P35754 | GLRX | Glutaredoxin-1 (Thioltransferase-1) (TTase-1) | 1.20 | 2.39E-07 |
| P46937 | YAP1 | Transcriptional coactivator YAP1 (Yes-associated protein 1) (Protein yorkie homolog) (Yes-associated protein YAP65 homolog) | 1.19 | 1.45E-08 |
| Q01995 | TAGLN | Transgelin (22 kDa actin-binding protein) (Protein WS3-10) (Smooth muscle protein 22-alpha) (SM22-alpha) | 1.17 | 1.45E-10 |
| P02649 | APOE | Apolipoprotein E (Apo-E) | 1.16 | 7.34E-08 |
| P13611 | VCAN | Versican core protein (Chondroitin sulfate proteoglycan core protein 2) (Chondroitin sulfate proteoglycan 2) (Glial hyaluronate-binding protein) (GHAP) (Large fibroblast proteoglycan) (PG-M) | 1.16 | 3.99E-12 |
| Q96S16 | JMJD8 | JmjC domain-containing protein 8 (Jumonji domain-containing protein 8) | 1.15 | 2.17E-06 |
| Q9UL42 | PNMA2 | Paraneoplastic antigen Ma2 (40 kDa neuronal protein) (Onconeuronal antigen Ma2) (Paraneoplastic neuronal antigen MM2) | 1.15 | 6.56E-12 |
| Q9Y572 | RIPK3 | Receptor-interacting serine/threonine-protein kinase 3 (EC 2.7.11.1) (RIP-like protein kinase 3) (Receptor-interacting protein 3) (RIP-3) | 1.14 | 3.85E-02 |
| P42357 | HAL | Histidine ammonia-lyase (Histidase) (EC 4.3.1.3) | 1.14 | 2.28E-10 |
| P19823 | ITIH2 | Inter-alpha-trypsin inhibitor heavy chain H2 (ITI heavy chain H2) (ITI-HC2) (Inter-alpha-inhibitor heavy chain 2) (Inter-alpha-trypsin inhibitor complex component II) (Serum-derived hyaluronan-associated protein) (SHAP) | 1.13 | 1.05E-09 |
| O00469 | PLOD2 | Procollagen-lysine,2-oxoglutarate 5-dioxygenase 2 (EC 1.14.11.4) (Lysyl hydroxylase 2) (LH2) | 1.13 | 8.09E-14 |
| O14613 | CDC42EP2 | Cdc42 effector protein 2 (Binder of Rho GTPases 1) | 1.12 | 1.25E-04 |
| P30626 | SRI | Sorcin (22 kDa protein) (CP-22) (CP22) (V19) | 1.11 | 2.37E-08 |
| P37235 | HPCAL1 | Hippocalcin-like protein 1 (Calcium-binding protein BDR-1) (HLP2) (Visinin-like protein 3) (VILIP-3) | 1.10 | 4.27E-08 |
| Q5W0Z9 | ZDHHC20 | Palmitoyltransferase ZDHHC20 (EC 2.3.1.225) (Acyltransferase ZDHHC20) (EC 2.3.1.-) (DHHC domain-containing cysteine-rich protein 20) (DHHC20) (Zinc finger DHHC domain-containing protein 20) | 1.10 | 6.27E-11 |
| P14927 | UQCRB | Cytochrome b-c1 complex subunit 7 (Complex III subunit 7) (Complex III subunit VII) (QP-C) (Ubiquinol-cytochrome c reductase complex 14 kDa protein) | 1.10 | 1.82E-06 |
| P54259 | ATN1 | Atrophin-1 (Dentatorubral-pallidoluysian atrophy protein) | 1.09 | 1.64E-05 |
| Q9UBL6 | CPNE7 | Copine-7 (Copine VII) | 1.08 | 1.33E-09 |
| Q9NVM1 | EVA1B | Protein eva-1 homolog B (Protein FAM176B) | 1.07 | 7.79E-07 |
| Q9H6Q4 | CIAO3 | Cytosolic iron-sulfur assembly component 3 (Cytosolic Fe-S cluster assembly factor NARFL) (Iron-only hydrogenase-like protein 1) (IOP1) (Nuclear prelamin A recognition factor-like protein) (Protein related to Narf) | 1.07 | 7.50E-09 |
| Q5TAT6 | COL13A1 | Collagen alpha-1(XIII) chain (COLXIIIA1) | 1.07 | 5.52E-07 |
| Q7Z5Q1 | CPEB2 | Cytoplasmic polyadenylation element-binding protein 2 (CPE-BP2) (CPE-binding protein 2) (hCPEB-2) | 1.07 | 1.04E-07 |
| P13929 | ENO3 | Beta-enolase (EC 4.2.1.11) (2-phospho-D-glycerate hydro-lyase) (Enolase 3) (Muscle-specific enolase) (MSE) (Skeletal muscle enolase) | 1.07 | 2.56E-02 |
| O75558 | STX11 | Syntaxin-11 | 1.06 | 1.05E-09 |
| O75508 | CLDN11 | Claudin-11 (Oligodendrocyte-specific protein) | 1.06 | 2.90E-04 |
| Q9BYX2 | TBC1D2 | TBC1 domain family member 2A (Armus) (Prostate antigen recognized and identified by SEREX 1) (PARIS-1) | 1.05 | 1.96E-08 |
| Q93062 | RBPMS | RNA-binding protein with multiple splicing (RBP-MS) (Heart and RRM expressed sequence) (Hermes) | 1.04 | 6.13E-06 |
| P12259 | F5 | Coagulation factor V (Activated protein C cofactor) (Proaccelerin, labile factor) [Cleaved into: Coagulation factor V heavy chain; Coagulation factor V light chain] | 1.04 | 1.65E-09 |
| Q04756 | HGFAC | Hepatocyte growth factor activator (HGF activator) (HGFA) (EC 3.4.21.-) [Cleaved into: Hepatocyte growth factor activator short chain; Hepatocyte growth factor activator long chain] | 1.04 | 1.26E-05 |
| P07093 | SERPINE2 | Glia-derived nexin (GDN) (Peptidase inhibitor 7) (PI-7) (Protease nexin 1) (PN-1) (Protease nexin I) (Serpin E2) | 1.04 | 1.67E-05 |
| Q92597 | NDRG1 | Protein NDRG1 (Differentiation-related gene 1 protein) (DRG-1) (N-myc downstream-regulated gene 1 protein) (Nickel-specific induction protein Cap43) (Reducing agents and tunicamycin-responsive protein) (RTP) (Rit42) | 1.02 | 1.39E-08 |
| Q9H910 | JPT2 | Jupiter microtubule associated homolog 2 (Hematological and neurological expressed 1-like protein) (HN1-like protein) | 1.01 | 4.51E-06 |
| C9JLW8 | MCRIP1 | Mapk-regulated corepressor-interacting protein 1 (Granulin-2) (Protein FAM195B) | 1.01 | 4.21E-05 |
| O00244 | ATOX1 | Copper transport protein ATOX1 (Metal transport protein ATX1) | 1.00 | 1.55E-04 |
| P51911 | CNN1 | Calponin-1 (Basic calponin) (Calponin H1, smooth muscle) | 1.00 | 1.24E-05 |
| P17612 | PRKACA | cAMP-dependent protein kinase catalytic subunit alpha (PKA C-alpha) (EC 2.7.11.11) | 1.00 | 2.35E-04 |
| P01024 | C3 | Complement C3 (C3 and PZP-like alpha-2-macroglobulin domain-containing protein 1) [Cleaved into: Complement C3 beta chain; C3-beta-c (C3bc); Complement C3 alpha chain; C3a anaphylatoxin; Acylation stimulating protein (ASP) (C3adesArg); Complement C3b alpha' chain; Complement C3c alpha' chain fragment 1; Complement C3dg fragment; Complement C3g fragment; Complement C3d fragment; Complement C3f fragment; Complement C3c alpha' chain fragment 2] | 1.00 | 1.61E-07 |
| P55290 | CDH13 | Cadherin-13 (Heart cadherin) (H-cadherin) (P105) (Truncated cadherin) (T-cad) (T-cadherin) | 0.99 | 4.52E-11 |
| O94953 | KDM4B | Lysine-specific demethylase 4B (EC 1.14.11.66) (JmjC domain-containing histone demethylation protein 3B) (Jumonji domain-containing protein 2B) ([histone H3]-trimethyl-L-lysine(9) demethylase 4B) | 0.97 | 1.13E-09 |
| A6NFQ2 | TCAF2 | TRPM8 channel-associated factor 2 (TRP channel-associated factor 2) | 0.97 | 6.90E-09 |
| P00734 | F2 | Prothrombin (EC 3.4.21.5) (Coagulation factor II) [Cleaved into: Activation peptide fragment 1; Activation peptide fragment 2; Thrombin light chain; Thrombin heavy chain] | 0.95 | 2.11E-04 |
| Q7Z3B1 | NEGR1 | Neuronal growth regulator 1 (IgLON family member 4) | 0.95 | 6.91E-08 |
| Q9UK76 | JPT1 | Jupiter microtubule associated homolog 1 (Androgen-regulated protein 2) (Hematological and neurological expressed 1 protein) [Cleaved into: Jupiter microtubule associated homolog 1, N-terminally processed] | 0.94 | 8.78E-05 |
| Q9H6X2 | ANTXR1 | Anthrax toxin receptor 1 (Tumor endothelial marker 8) | 0.94 | 7.80E-08 |
| P51884 | LUM | Lumican (Keratan sulfate proteoglycan lumican) (KSPG lumican) | 0.93 | 4.14E-06 |
| P22352 | GPX3 | Glutathione peroxidase 3 (GPx-3) (GSHPx-3) (EC 1.11.1.9) (Extracellular glutathione peroxidase) (Plasma glutathione peroxidase) (GPx-P) (GSHPx-P) | 0.93 | 1.54E-02 |
| Q02952 | AKAP12 | A-kinase anchor protein 12 (AKAP-12) (A-kinase anchor protein 250 kDa) (AKAP 250) (Gravin) (Myasthenia gravis autoantigen) | 0.93 | 7.24E-14 |
| P05026 | ATP1B1 | Sodium/potassium-transporting ATPase subunit beta-1 (Sodium/potassium-dependent ATPase subunit beta-1) | 0.92 | 4.85E-10 |
| Q9NQS3 | NECTIN3 | Nectin-3 (CDw113) (Nectin cell adhesion molecule 3) (Poliovirus receptor-related protein 3) (CD antigen CD113) | 0.91 | 1.79E-07 |
| Q6ZTI6 | RFLNA | Refilin-A (Regulator of filamin protein A) (RefilinA) | 0.91 | 9.19E-08 |
| O43516 | WIPF1 | WAS/WASL-interacting protein family member 1 (Protein PRPL-2) (Wiskott-Aldrich syndrome protein-interacting protein) (WASP-interacting protein) | 0.88 | 3.62E-06 |
| Q9H3M7 | TXNIP | Thioredoxin-interacting protein (Thioredoxin-binding protein 2) (Vitamin D3 up-regulated protein 1) | 0.88 | 7.15E-03 |
| Q16772 | GSTA3 | Glutathione S-transferase A3 (EC 2.5.1.18) (GST class-alpha member 3) (Glutathione S-transferase A3-3) | 0.88 | 6.99E-04 |
| P60903 | S100A10 | Protein S100-A10 (Calpactin I light chain) (Calpactin-1 light chain) (Cellular ligand of annexin II) (S100 calcium-binding protein A10) (p10 protein) (p11) | 0.88 | 2.85E-04 |
| P16144 | ITGB4 | Integrin beta-4 (GP150) (CD antigen CD104) | 0.88 | 1.62E-04 |
| Q8ND76 | CCNY | Cyclin-Y (Cyc-Y) (Cyclin box protein 1) (Cyclin fold protein 1) (cyclin-X) | 0.87 | 3.16E-05 |
| A5A3E0 | POTEF | POTE ankyrin domain family member F (ANKRD26-like family C member 1B) (Chimeric POTE-actin protein) | 0.87 | 5.79E-04 |
| P35625 | TIMP3 | Metalloproteinase inhibitor 3 (Protein MIG-5) (Tissue inhibitor of metalloproteinases 3) (TIMP-3) | 0.87 | 4.87E-05 |
| O60888 | CUTA | Protein CutA (Acetylcholinesterase-associated protein) (Brain acetylcholinesterase putative membrane anchor) | 0.87 | 2.54E-04 |
| Q9Y4K0 | LOXL2 | Lysyl oxidase homolog 2 (EC 1.4.3.13) (Lysyl oxidase-like protein 2) (Lysyl oxidase-related protein 2) (Lysyl oxidase-related protein WS9-14) | 0.87 | 1.11E-05 |
| O14763 | TNFRSF10B | Tumor necrosis factor receptor superfamily member 10B (Death receptor 5) (TNF-related apoptosis-inducing ligand receptor 2) (TRAIL receptor 2) (TRAIL-R2) (CD antigen CD262) | 0.86 | 7.84E-08 |
| Q9GZU7 | CTDSP1 | Carboxy-terminal domain RNA polymerase II polypeptide A small phosphatase 1 (EC 3.1.3.16) (Nuclear LIM interactor-interacting factor 3) (NLI-IF) (NLI-interacting factor 3) (Small C-terminal domain phosphatase 1) (SCP1) (Small CTD phosphatase 1) | 0.86 | 3.04E-05 |
| Q9HD23 | MRS2 | Magnesium transporter MRS2 homolog, mitochondrial (MRS2-like protein) | 0.86 | 8.40E-04 |
| P49591 | SARS1 | Serine--tRNA ligase, cytoplasmic (EC 6.1.1.11) (Seryl-tRNA synthetase) (SerRS) (Seryl-tRNA(Ser/Sec) synthetase) | 0.86 | 1.43E-07 |
| Q9P0N9 | TBC1D7 | TBC1 domain family member 7 (Cell migration-inducing protein 23) | 0.86 | 1.34E-02 |
| Q96HE7 | ERO1A | ERO1-like protein alpha (ERO1-L) (ERO1-L-alpha) (EC 1.8.4.-) (Endoplasmic oxidoreductin-1-like protein) (Endoplasmic reticulum oxidoreductase alpha) (Oxidoreductin-1-L-alpha) | 0.85 | 2.65E-06 |
| Q15005 | SPC25 | Signal peptidase complex subunit 2 (Microsomal signal peptidase 25 kDa subunit) (SPase 25 kDa subunit) | 0.85 | 2.10E-02 |
| P05997 | COL5A2 | Collagen alpha-2(V) chain | 0.85 | 1.84E-09 |
| O60245 | PCDH7 | Protocadherin-7 (Brain-heart protocadherin) (BH-Pcdh) | 0.85 | 6.30E-03 |
| Q9UHQ1 | NARF | Nuclear prelamin A recognition factor (Iron-only hydrogenase-like protein 2) (IOP2) | 0.84 | 3.24E-06 |
| Q9NR00 | TCIM | Transcriptional and immune response regulator (Thyroid cancer protein 1) (TC-1) | 0.84 | 1.00E-03 |
| P12429 | ANXA3 | Annexin A3 (35-alpha calcimedin) (Annexin III) (Annexin-3) (Inositol 1,2-cyclic phosphate 2-phosphohydrolase) (Lipocortin III) (Placental anticoagulant protein III) (PAP-III) | 0.83 | 3.81E-09 |
| Q9NZN3 | EHD3 | EH domain-containing protein 3 (PAST homolog 3) | 0.82 | 1.71E-05 |
| Q92954 | PRG4 | Proteoglycan 4 (Lubricin) (Megakaryocyte-stimulating factor) (Superficial zone proteoglycan) [Cleaved into: Proteoglycan 4 C-terminal part] | 0.82 | 4.72E-04 |
| P06732 | CKM | Creatine kinase M-type (EC 2.7.3.2) (Creatine kinase M chain) (Creatine phosphokinase M-type) (CPK-M) (M-CK) | 0.82 | 1.05E-05 |
| P56211 | ARPP19 | cAMP-regulated phosphoprotein 19 (ARPP-19) | 0.82 | 6.10E-04 |
| P26022 | PTX3 | Pentraxin-related protein PTX3 (Pentaxin-related protein PTX3) (Tumor necrosis factor alpha-induced protein 5) (TNF alpha-induced protein 5) (Tumor necrosis factor-inducible gene 14 protein) (TSG-14) | 0.81 | 3.84E-06 |
| P12111 | COL6A3 | Collagen alpha-3(VI) chain | 0.81 | 1.45E-02 |
| Q07954 | LRP1 | Prolow-density lipoprotein receptor-related protein 1 (LRP-1) (Alpha-2-macroglobulin receptor) (A2MR) (Apolipoprotein E receptor) (APOER) (CD antigen CD91) [Cleaved into: Low-density lipoprotein receptor-related protein 1 85 kDa subunit (LRP-85); Low-density lipoprotein receptor-related protein 1 515 kDa subunit (LRP-515); Low-density lipoprotein receptor-related protein 1 intracellular domain (LRPICD)] | 0.80 | 1.19E-03 |
| Q9HAU0 | PLEKHA5 | Pleckstrin homology domain-containing family A member 5 (PH domain-containing family A member 5) (Phosphoinositol 3-phosphate-binding protein 2) (PEPP-2) | 0.80 | 1.21E-10 |
| Q8WUF5 | PPP1R13L | RelA-associated inhibitor (Inhibitor of ASPP protein) (Protein iASPP) (NFkB-interacting protein 1) (PPP1R13B-like protein) | 0.79 | 4.97E-11 |
| P30039 | PBLD | Phenazine biosynthesis-like domain-containing protein (EC 5.1.-.-) (MAWD-binding protein) (MAWDBP) (Unknown protein 32 from 2D-page of liver tissue) | 0.79 | 3.54E-05 |
| Q5TH69 | ARFGEF3 | Brefeldin A-inhibited guanine nucleotide-exchange protein 3 (ARFGEF family member 3) | 0.79 | 2.16E-06 |
| Q15464 | SHB | SH2 domain-containing adapter protein B | 0.78 | 2.34E-05 |
| P0C0L4 | C4A | Complement C4-A (Acidic complement C4) (C3 and PZP-like alpha-2-macroglobulin domain-containing protein 2) [Cleaved into: Complement C4 beta chain; Complement C4-A alpha chain; C4a anaphylatoxin; C4b-A; C4d-A; Complement C4 gamma chain] | 0.78 | 7.73E-08 |
| Q9HB63 | NTN4 | Netrin-4 (Beta-netrin) (Hepar-derived netrin-like protein) | 0.78 | 9.71E-07 |
| P09972 | ALDOC | Fructose-bisphosphate aldolase C (EC 4.1.2.13) (Brain-type aldolase) | 0.78 | 4.85E-10 |
| P84157 | MXRA7 | Matrix-remodeling-associated protein 7 | 0.78 | 9.92E-06 |
| P04920 | SLC4A2 | Anion exchange protein 2 (AE 2) (Anion exchanger 2) (Non-erythroid band 3-like protein) (BND3L) (Solute carrier family 4 member 2) | 0.78 | 6.16E-08 |
| Q99541 | PLIN2 | Perilipin-2 (Adipophilin) (Adipose differentiation-related protein) (ADRP) | 0.78 | 4.72E-04 |
| P17252 | PRKCA | Protein kinase C alpha type (PKC-A) (PKC-alpha) (EC 2.7.11.13) | 0.77 | 5.70E-12 |
| Q9C005 | DPY30 | Protein dpy-30 homolog (Dpy-30-like protein) (Dpy-30L) | 0.77 | 1.57E-04 |
| P10124 | SRGN | Serglycin (Hematopoietic proteoglycan core protein) (Platelet proteoglycan core protein) (P.PG) (Secretory granule proteoglycan core protein) | 0.76 | 2.74E-04 |
| Q9Y5L4 | TIMM13 | Mitochondrial import inner membrane translocase subunit Tim13 | 0.76 | 4.86E-05 |
| Q9NZ45 | CISD1 | CDGSH iron-sulfur domain-containing protein 1 (Cysteine transaminase CISD1) (EC 2.6.1.3) (MitoNEET) | 0.75 | 1.86E-04 |
| O95379 | TNFAIP8 | Tumor necrosis factor alpha-induced protein 8 (TNF alpha-induced protein 8) (Head and neck tumor and metastasis-related protein) (MDC-3.13) (NF-kappa-B-inducible DED-containing protein) (NDED) (SCC-S2) (TNF-induced protein GG2-1) | 0.74 | 1.12E-03 |
| Q53GG5 | PDLIM3 | PDZ and LIM domain protein 3 (Actinin-associated LIM protein) (Alpha-actinin-2-associated LIM protein) | 0.74 | 3.47E-06 |
| Q8N2G8 | GHDC | GH3 domain-containing protein | 0.74 | 4.08E-06 |
| P05452 | CLEC3B | Tetranectin (TN) (C-type lectin domain family 3 member B) (Plasminogen kringle 4-binding protein) | 0.74 | 5.27E-07 |
| P60059 | SEC61G | Protein transport protein Sec61 subunit gamma | 0.73 | 1.97E-03 |
| Q99471 | PFDN5 | Prefoldin subunit 5 (Myc modulator 1) (c-Myc-binding protein Mm-1) | 0.73 | 5.31E-06 |
| Q16799 | RTN1 | Reticulon-1 (Neuroendocrine-specific protein) | 0.73 | 5.81E-04 |
| Q155Q3 | DIXDC1 | Dixin (Coiled-coil protein DIX1) (Coiled-coil-DIX1) (DIX domain-containing protein 1) | 0.73 | 8.66E-06 |
| P62942 | FKBP1A | Peptidyl-prolyl cis-trans isomerase FKBP1A (PPIase FKBP1A) (EC 5.2.1.8) (12 kDa FK506-binding protein) (12 kDa FKBP) (FKBP-12) (Calstabin-1) (FK506-binding protein 1A) (FKBP-1A) (Immunophilin FKBP12) (Rotamase) | 0.73 | 8.97E-06 |
| P36955 | SERPINF1 | Pigment epithelium-derived factor (PEDF) (Cell proliferation-inducing gene 35 protein) (EPC-1) (Serpin F1) | 0.73 | 4.20E-07 |
| Q9H0W9 | C11orf54 | Ester hydrolase C11orf54 (EC 3.1.-.-) | 0.72 | 6.71E-03 |
| Q9NXG6 | P4HTM | Transmembrane prolyl 4-hydroxylase (P4H-TM) (EC 1.14.11.29) (Hypoxia-inducible factor prolyl hydroxylase 4) (HIF-PH4) (HIF-prolyl hydroxylase 4) (HPH-4) | 0.72 | 3.05E-08 |
| P41229 | KDM5C | Lysine-specific demethylase 5C (EC 1.14.11.67) (Histone demethylase JARID1C) (Jumonji/ARID domain-containing protein 1C) (Protein SmcX) (Protein Xe169) ([histone H3]-trimethyl-L-lysine(4) demethylase 5C) | 0.72 | 1.40E-05 |
| O14907 | TAX1BP3 | Tax1-binding protein 3 (Glutaminase-interacting protein 3) (Tax interaction protein 1) (TIP-1) (Tax-interacting protein 1) | 0.72 | 2.16E-04 |
| P04181 | OAT | Ornithine aminotransferase, mitochondrial (EC 2.6.1.13) (Ornithine delta-aminotransferase) (Ornithine--oxo-acid aminotransferase) [Cleaved into: Ornithine aminotransferase, hepatic form; Ornithine aminotransferase, renal form] | 0.72 | 3.10E-06 |
| Q6NY19 | KANK3 | KN motif and ankyrin repeat domain-containing protein 3 (Ankyrin repeat domain-containing protein 47) | 0.71 | 4.10E-09 |
| Q14653 | IRF3 | Interferon regulatory factor 3 (IRF-3) | 0.71 | 2.12E-03 |
| P08727 | KRT19 | Keratin, type I cytoskeletal 19 (Cytokeratin-19) (CK-19) (Keratin-19) (K19) | 0.71 | 5.44E-04 |
| Q86U90 | YRDC | Threonylcarbamoyl-AMP synthase (EC 2.7.7.87) (Dopamine receptor-interacting protein 3) (Ischemia/reperfusion-inducible protein homolog) (hIRIP) | 0.70 | 1.22E-04 |
| Q99758 | ABCA3 | Phospholipid-transporting ATPase ABCA3 (EC 7.6.2.1) (ABC-C transporter) (ATP-binding cassette sub-family A member 3) (ATP-binding cassette transporter 3) (ATP-binding cassette 3) (Xenobiotic-transporting ATPase ABCA3) (EC 7.6.2.2) [Cleaved into: 150 Kda mature form] | 0.70 | 5.51E-06 |
| O43294 | TGFB1I1 | Transforming growth factor beta-1-induced transcript 1 protein (Androgen receptor coactivator 55 kDa protein) (Androgen receptor-associated protein of 55 kDa) (Hydrogen peroxide-inducible clone 5 protein) (Hic-5) | 0.70 | 2.03E-05 |
| P52943 | CRIP2 | Cysteine-rich protein 2 (CRP-2) (Protein ESP1) | 0.70 | 1.75E-06 |
| Q14393 | GAS6 | Growth arrest-specific protein 6 (GAS-6) (AXL receptor tyrosine kinase ligand) | 0.70 | 6.27E-07 |
| O43768 | ENSA | Alpha-endosulfine (ARPP-19e) | 0.69 | 3.21E-03 |
| Q8IWU6 | SULF1 | Extracellular sulfatase Sulf-1 (hSulf-1) (Arylsulfatase) (EC 3.1.6.1) (N-acetylglucosamine-6-sulfatase) (EC 3.1.6.14) [Cleaved into: Extracellular sulfatase Sulf-2 secreted form] | 0.69 | 4.03E-03 |
| P02461 | COL3A1 | Collagen alpha-1(III) chain | 0.69 | 2.66E-06 |
| Q9P2A4 | ABI3 | ABI gene family member 3 (New molecule including SH3) (Nesh) | 0.69 | 6.68E-10 |
| O60315 | ZEB2 | Zinc finger E-box-binding homeobox 2 (Smad-interacting protein 1) (SMADIP1) (Zinc finger homeobox protein 1b) | 0.69 | 9.68E-08 |
| Q92546 | RGP1 | RAB6A-GEF complex partner protein 2 (Retrograde Golgi transport protein RGP1 homolog) | 0.69 | 4.33E-04 |
| P23142 | FBLN1 | Fibulin-1 (FIBL-1) | 0.69 | 1.47E-06 |
| P13674 | P4HA1 | Prolyl 4-hydroxylase subunit alpha-1 (4-PH alpha-1) (EC 1.14.11.2) (Procollagen-proline,2-oxoglutarate-4-dioxygenase subunit alpha-1) | 0.69 | 6.34E-08 |
| O15427 | SLC16A3 | Monocarboxylate transporter 4 (MCT 4) (Solute carrier family 16 member 3) | 0.69 | 3.32E-03 |
| Q16739 | UGCG | Ceramide glucosyltransferase (EC 2.4.1.80) (GLCT-1) (Glucosylceramide synthase) (GCS) (Glycosylceramide synthase) (UDP-glucose ceramide glucosyltransferase) (UDP-glucose:N-acylsphingosine D-glucosyltransferase) | 0.69 | 5.40E-06 |
| Q5T6V5 | C9orf64 | Queuosine 5'-phosphate N-glycosylase/hydrolase (EC 3.2.2.-) (Q-nucleotide N-glycosylase 1) (Queuine salvage protein QNG1) (Queuosine-nucleotide N-glycosylase/hydrolase) | 0.69 | 2.55E-04 |
| Q96H79 | ZC3HAV1L | Zinc finger CCCH-type antiviral protein 1-like | 0.68 | 2.46E-03 |
| Q58DX5 | NAALADL2 | Inactive N-acetylated-alpha-linked acidic dipeptidase-like protein 2 (NAALADase L2) | 0.68 | 2.45E-02 |
| Q9P1T7 | MDFIC | MyoD family inhibitor domain-containing protein (I-mfa domain-containing protein) (hIC) | 0.68 | 2.59E-03 |
| Q6YHK3 | CD109 | CD109 antigen (150 kDa TGF-beta-1-binding protein) (C3 and PZP-like alpha-2-macroglobulin domain-containing protein 7) (Platelet-specific Gov antigen) (p180) (r150) (CD antigen CD109) | 0.68 | 1.72E-07 |
| Q8N126 | CADM3 | Cell adhesion molecule 3 (Brain immunoglobulin receptor) (Immunoglobulin superfamily member 4B) (IgSF4B) (Nectin-like protein 1) (NECL-1) (Synaptic cell adhesion molecule 3) (SynCAM3) (TSLC1-like protein 1) (TSLL1) | 0.68 | 2.38E-06 |
| Q16527 | CSRP2 | Cysteine and glycine-rich protein 2 (Cysteine-rich protein 2) (CRP2) (LIM domain only protein 5) (LMO-5) (Smooth muscle cell LIM protein) (SmLIM) | 0.68 | 1.45E-02 |
| P23219 | PTGS1 | Prostaglandin G/H synthase 1 (EC 1.14.99.1) (Cyclooxygenase-1) (COX-1) (Prostaglandin H2 synthase 1) (PGH synthase 1) (PGHS-1) (PHS 1) (Prostaglandin-endoperoxide synthase 1) | 0.68 | 2.23E-03 |
| P30530 | AXL | Tyrosine-protein kinase receptor UFO (EC 2.7.10.1) (AXL oncogene) | 0.68 | 4.78E-07 |
| P00338 | LDHA | L-lactate dehydrogenase A chain (LDH-A) (EC 1.1.1.27) (Cell proliferation-inducing gene 19 protein) (LDH muscle subunit) (LDH-M) (Renal carcinoma antigen NY-REN-59) | 0.68 | 6.14E-04 |
| Q8TAA5 | GRPEL2 | GrpE protein homolog 2, mitochondrial (Mt-GrpE#2) | 0.67 | 1.13E-02 |
| P52789 | HK2 | Hexokinase-2 (EC 2.7.1.1) (Hexokinase type II) (HK II) (Hexokinase-B) (Muscle form hexokinase) | 0.67 | 2.96E-09 |
| Q8WWI5 | SLC44A1 | Choline transporter-like protein 1 (CDw92) (Solute carrier family 44 member 1) (CD antigen CD92) | 0.67 | 5.24E-04 |
| P80723 | BASP1 | Brain acid soluble protein 1 (22 kDa neuronal tissue-enriched acidic protein) (Neuronal axonal membrane protein NAP-22) | 0.67 | 3.65E-05 |
| Q6P1K2 | PMF1 | Polyamine-modulated factor 1 (PMF-1) | 0.67 | 5.89E-05 |
| P20674 | COX5A | Cytochrome c oxidase subunit 5A, mitochondrial (Cytochrome c oxidase polypeptide Va) | 0.67 | 5.88E-03 |
| Q6IA17 | SIGIRR | Single Ig IL-1-related receptor (Single Ig IL-1R-related molecule) (Single immunoglobulin domain-containing IL1R-related protein) (Toll/interleukin-1 receptor 8) (TIR8) | 0.67 | 2.23E-03 |
| Q96HQ2 | CDKN2AIPNL | CDKN2AIP N-terminal-like protein (CDKN2A-interacting protein N-terminal-like protein) | 0.67 | 3.04E-03 |
| Q13642 | FHL1 | Four and a half LIM domains protein 1 (FHL-1) (Skeletal muscle LIM-protein 1) (SLIM) (SLIM-1) | 0.67 | 1.03E-05 |
| Q96SL4 | GPX7 | Glutathione peroxidase 7 (GPx-7) (GSHPx-7) (EC 1.11.1.9) (CL683) | 0.67 | 6.70E-07 |
| O43805 | SSNA1 | Microtubule nucleation factor SSNA1 (Nuclear autoantigen of 14 kDa) (Sjoegren syndrome nuclear autoantigen 1) | 0.66 | 3.95E-03 |
| Q8N5K1 | CISD2 | CDGSH iron-sulfur domain-containing protein 2 (Endoplasmic reticulum intermembrane small protein) (MitoNEET-related 1 protein) (Miner1) (Nutrient-deprivation autophagy factor-1) (NAF-1) | 0.66 | 7.55E-04 |
| O75368 | SH3BGRL | Adapter SH3BGRL (SH3 domain-binding glutamic acid-rich-like protein 1) | 0.66 | 5.44E-06 |
| Q9GZT9 | EGLN1 | Egl nine homolog 1 (EC 1.14.11.29) (Hypoxia-inducible factor prolyl hydroxylase 2) (HIF-PH2) (HIF-prolyl hydroxylase 2) (HPH-2) (Prolyl hydroxylase domain-containing protein 2) (PHD2) (SM-20) | 0.66 | 1.92E-06 |
| Q8WXI4 | ACOT11 | Acyl-coenzyme A thioesterase 11 (Acyl-CoA thioesterase 11) (EC 3.1.2.-) (Acyl-CoA thioester hydrolase 11) (Adipose-associated thioesterase) (Brown fat-inducible thioesterase) (BFIT) (Palmitoyl-coenzyme A thioesterase) (EC 3.1.2.2) | 0.66 | 2.65E-06 |
| P16591 | FER | Tyrosine-protein kinase Fer (EC 2.7.10.2) (Feline encephalitis virus-related kinase FER) (Fujinami poultry sarcoma/Feline sarcoma-related protein Fer) (Proto-oncogene c-Fer) (Tyrosine kinase 3) (p94-Fer) | 0.66 | 1.09E-04 |
| Q8TF74 | WIPF2 | WAS/WASL-interacting protein family member 2 (WASP-interacting protein-related protein) (WIP- and CR16-homologous protein) (WIP-related protein) | 0.66 | 1.27E-04 |
| P04075 | ALDOA | Fructose-bisphosphate aldolase A (EC 4.1.2.13) (Lung cancer antigen NY-LU-1) (Muscle-type aldolase) | 0.65 | 9.23E-05 |
| Q96JQ0 | DCHS1 | Protocadherin-16 (Cadherin-19) (Cadherin-25) (Fibroblast cadherin-1) (Protein dachsous homolog 1) | 0.65 | 6.42E-10 |
| P56945 | BCAR1 | Breast cancer anti-estrogen resistance protein 1 (CRK-associated substrate) (Cas scaffolding protein family member 1) (p130cas) | 0.65 | 1.88E-09 |
| P40121 | CAPG | Macrophage-capping protein (Actin regulatory protein CAP-G) | 0.65 | 1.00E-08 |
| P37802 | TAGLN2 | Transgelin-2 (Epididymis tissue protein Li 7e) (SM22-alpha homolog) | 0.65 | 1.83E-05 |
| Q9BQ51 | PDCD1LG2 | Programmed cell death 1 ligand 2 (PD-1 ligand 2) (PD-L2) (PDCD1 ligand 2) (Programmed death ligand 2) (Butyrophilin B7-DC) (B7-DC) (CD antigen CD273) | 0.65 | 4.51E-06 |
| P24844 | MYL9 | Myosin regulatory light polypeptide 9 (20 kDa myosin light chain) (LC20) (MLC-2C) (Myosin RLC) (Myosin regulatory light chain 2, smooth muscle isoform) (Myosin regulatory light chain 9) (Myosin regulatory light chain MRLC1) | 0.64 | 3.88E-04 |
| P27105 | STOM | Stomatin (Erythrocyte band 7 integral membrane protein) (Erythrocyte membrane protein band 7.2) (Protein 7.2b) | 0.64 | 9.45E-08 |
| Q9Y2J2 | EPB41L3 | Band 4.1-like protein 3 (4.1B) (Differentially expressed in adenocarcinoma of the lung protein 1) (DAL-1) (Erythrocyte membrane protein band 4.1-like 3) [Cleaved into: Band 4.1-like protein 3, N-terminally processed] | 0.64 | 1.88E-08 |
| P21926 | CD9 | CD9 antigen (5H9 antigen) (Cell growth-inhibiting gene 2 protein) (Leukocyte antigen MIC3) (Motility-related protein) (MRP-1) (Tetraspanin-29) (Tspan-29) (p24) (CD antigen CD9) | 0.64 | 4.40E-03 |
| Q9BQC3 | DPH2 | 2-(3-amino-3-carboxypropyl)histidine synthase subunit 2 (Diphthamide biosynthesis protein 2) (Diphtheria toxin resistance protein 2) (S-adenosyl-L-methionine:L-histidine 3-amino-3-carboxypropyltransferase 2) | 0.64 | 4.36E-06 |
| Q86TX2 | ACOT1 | Acyl-coenzyme A thioesterase 1 (Acyl-CoA thioesterase 1) (EC 3.1.2.-) (CTE-I) (CTE-Ib) (Inducible cytosolic acyl-coenzyme A thioester hydrolase) (Long chain acyl-CoA thioester hydrolase) (Long chain acyl-CoA hydrolase) (Palmitoyl-coenzyme A thioesterase) (EC 3.1.2.2) | 0.63 | 4.58E-08 |
| Q9H0F7 | ARL6 | ADP-ribosylation factor-like protein 6 (Bardet-Biedl syndrome 3 protein) | 0.63 | 2.25E-03 |
| Q9Y597 | KCTD3 | BTB/POZ domain-containing protein KCTD3 (Renal carcinoma antigen NY-REN-45) | 0.63 | 1.32E-02 |
| Q8N5C1 | CALHM5 | Calcium homeostasis modulator protein 5 (Protein FAM26E) | 0.63 | 1.18E-04 |
| Q9UBT7 | CTNNAL1 | Alpha-catulin (Alpha-catenin-related protein) (ACRP) (Catenin alpha-like protein 1) | 0.63 | 1.62E-03 |
| Q6ZSJ8 | C1orf122 | Uncharacterized protein C1orf122 (Protein ALAESM) | 0.63 | 3.96E-04 |
| Q9NRG1 | PRTFDC1 | Phosphoribosyltransferase domain-containing protein 1 | 0.63 | 2.37E-02 |
| P10589 | NR2F1 | COUP transcription factor 1 (COUP-TF1) (COUP transcription factor I) (COUP-TF I) (Nuclear receptor subfamily 2 group F member 1) (V-erbA-related protein 3) (EAR-3) | 0.62 | 2.31E-03 |
| P30046 | DDT | D-dopachrome decarboxylase (EC 4.1.1.84) (D-dopachrome tautomerase) (Phenylpyruvate tautomerase II) | 0.62 | 7.45E-05 |
| Q9NY12 | GAR1 | H/ACA ribonucleoprotein complex subunit 1 (Nucleolar protein family A member 1) (snoRNP protein GAR1) | 0.62 | 8.20E-04 |
| Q4L180 | FILIP1L | Filamin A-interacting protein 1-like (130 kDa GPBP-interacting protein) (90 kDa GPBP-interacting protein) (Protein down-regulated in ovarian cancer 1) (DOC-1) | 0.62 | 2.40E-09 |
| Q96RW7 | HMCN1 | Hemicentin-1 (Fibulin-6) (FIBL-6) | 0.62 | 5.14E-04 |
| Q99666 | RGPD5 | RANBP2-like and GRIP domain-containing protein 5/6 (Ran-binding protein 2-like 1/2) (RanBP2-like 1/2) (RanBP2L1) (RanBP2L2) (Sperm membrane protein BS-63) | 0.62 | 2.57E-02 |
| Q99417 | MYCBP | c-Myc-binding protein (Associate of Myc 1) (AMY-1) | 0.61 | 1.12E-03 |
| Q6UB35 | MTHFD1L | Monofunctional C1-tetrahydrofolate synthase, mitochondrial (EC 6.3.4.3) (Formyltetrahydrofolate synthetase) | 0.61 | 1.72E-07 |
| Q13057 | COASY | Bifunctional coenzyme A synthase (CoA synthase) (NBP) (POV-2) [Includes: Phosphopantetheine adenylyltransferase (EC 2.7.7.3) (Dephospho-CoA pyrophosphorylase) (Pantetheine-phosphate adenylyltransferase) (PPAT); Dephospho-CoA kinase (DPCK) (EC 2.7.1.24) (Dephosphocoenzyme A kinase) (DPCOAK)] | 0.61 | 5.66E-05 |
| Q9Y3C7 | MED31 | Mediator of RNA polymerase II transcription subunit 31 (Mediator complex subunit 31) (Mediator complex subunit SOH1) (hSOH1) | 0.61 | 1.45E-02 |
| Q9HBL0 | TNS1 | Tensin-1 (EC 3.1.3.-) | 0.61 | 1.05E-09 |
| P17931 | LGALS3 | Galectin-3 (Gal-3) (35 kDa lectin) (Carbohydrate-binding protein 35) (CBP 35) (Galactose-specific lectin 3) (Galactoside-binding protein) (GALBP) (IgE-binding protein) (L-31) (Laminin-binding protein) (Lectin L-29) (Mac-2 antigen) | 0.60 | 1.05E-02 |
| O15212 | PFDN6 | Prefoldin subunit 6 (Protein Ke2) | 0.60 | 1.33E-04 |
| Q00587 | CDC42EP1 | Cdc42 effector protein 1 (Binder of Rho GTPases 5) (Serum protein MSE55) | 0.60 | 5.71E-05 |
| O95183 | VAMP5 | Vesicle-associated membrane protein 5 (VAMP-5) (Myobrevin) | 0.60 | 1.32E-03 |
| Q6ZMP0 | THSD4 | Thrombospondin type-1 domain-containing protein 4 (A disintegrin and metalloproteinase with thrombospondin motifs-like protein 6) (ADAMTS-like protein 6) (ADAMTSL-6) | 0.60 | 6.56E-04 |
| P17813 | ENG | Endoglin (CD antigen CD105) | 0.60 | 2.72E-07 |
| Q3SY69 | ALDH1L2 | Mitochondrial 10-formyltetrahydrofolate dehydrogenase (Mitochondrial 10-FTHFDH) (mtFDH) (EC 1.5.1.6) (Aldehyde dehydrogenase family 1 member L2) | 0.59 | 8.77E-06 |
| Q9BXJ4 | C1QTNF3 | Complement C1q tumor necrosis factor-related protein 3 (Collagenous repeat-containing sequence 26 kDa protein) (CORS26) (Secretory protein CORS26) | 0.59 | 1.12E-02 |
| P21399 | ACO1 | Cytoplasmic aconitate hydratase (Aconitase) (EC 4.2.1.3) (Citrate hydro-lyase) (Ferritin repressor protein) (Iron regulatory protein 1) (IRP1) (Iron-responsive element-binding protein 1) (IRE-BP 1) | 0.59 | 7.33E-07 |
| P51452 | DUSP3 | Dual specificity protein phosphatase 3 (EC 3.1.3.16) (EC 3.1.3.48) (Dual specificity protein phosphatase VHR) (Vaccinia H1-related phosphatase) (VHR) | 0.59 | 1.90E-05 |
| O43760 | SYNGR2 | Synaptogyrin-2 (Cellugyrin) | 0.59 | 1.13E-02 |
| Q9BV57 | ADI1 | Acireductone dioxygenase (Acireductone dioxygenase (Fe(2+)-requiring)) (ARD') (Fe-ARD) (EC 1.13.11.54) (Acireductone dioxygenase (Ni(2+)-requiring)) (ARD) (Ni-ARD) (EC 1.13.11.53) (Membrane-type 1 matrix metalloproteinase cytoplasmic tail-binding protein 1) (MTCBP-1) (Submergence-induced protein-like factor) (Sip-L) | 0.59 | 9.92E-04 |
| Q9UBI6 | GNG12 | Guanine nucleotide-binding protein G(I)/G(S)/G(O) subunit gamma-12 | 0.59 | 1.38E-03 |
| Q8IWZ6 | BBS7 | Bardet-Biedl syndrome 7 protein (BBS2-like protein 1) | 0.59 | 5.77E-05 |
| Q16822 | PCK2 | Phosphoenolpyruvate carboxykinase [GTP], mitochondrial (PEPCK-M) (EC 4.1.1.32) | 0.58 | 4.98E-07 |
| Q5TKA1 | LIN9 | Protein lin-9 homolog (HuLin-9) (hLin-9) (Beta subunit-associated regulator of apoptosis) (TUDOR gene similar protein) (Type I interferon receptor beta chain-associated protein) (pRB-associated protein) | 0.58 | 1.78E-03 |
| Q9UQ16 | DNM3 | Dynamin-3 (EC 3.6.5.5) (Dynamin, testicular) (T-dynamin) | 0.58 | 1.41E-03 |
| P43007 | SLC1A4 | Neutral amino acid transporter A (Alanine/serine/cysteine/threonine transporter 1) (ASCT-1) (Solute carrier family 1 member 4) | 0.58 | 9.78E-03 |
| P58107 | EPPK1 | Epiplakin (450 kDa epidermal antigen) | 0.58 | 1.33E-04 |
| P68106 | FKBP1B | Peptidyl-prolyl cis-trans isomerase FKBP1B (PPIase FKBP1B) (EC 5.2.1.8) (12.6 kDa FK506-binding protein) (12.6 kDa FKBP) (FKBP-12.6) (FK506-binding protein 1B) (FKBP-1B) (Immunophilin FKBP12.6) (Rotamase) (h-FKBP-12) | 0.58 | 1.27E-02 |
| A4D1U4 | DENND11 | DENN domain-containing protein 11 (DENND11) (Protein LCHN) | 0.58 | 2.73E-04 |
| Q9NWU2 | GID8 | Glucose-induced degradation protein 8 homolog (Two hybrid-associated protein 1 with RanBPM) (Twa1) | 0.58 | 9.72E-03 |
| Q9UGN5 | PARP2 | Poly [ADP-ribose] polymerase 2 (PARP-2) (hPARP-2) (EC 2.4.2.30) (ADP-ribosyltransferase diphtheria toxin-like 2) (ARTD2) (DNA ADP-ribosyltransferase PARP2) (EC 2.4.2.-) (NAD(+) ADP-ribosyltransferase 2) (ADPRT-2) (Poly[ADP-ribose] synthase 2) (pADPRT-2) (Protein poly-ADP-ribosyltransferase PARP2) (EC 2.4.2.-) | 0.58 | 1.73E-03 |
| O95210 | STBD1 | Starch-binding domain-containing protein 1 (Genethonin-1) (Glycophagy cargo receptor STBD1) | 0.57 | 2.13E-02 |
| P43005 | SLC1A1 | Excitatory amino acid transporter 3 (Excitatory amino-acid carrier 1) (Neuronal and epithelial glutamate transporter) (Sodium-dependent glutamate/aspartate transporter 3) (Solute carrier family 1 member 1) | 0.57 | 1.96E-02 |
| Q15434 | RBMS2 | RNA-binding motif, single-stranded-interacting protein 2 (Suppressor of CDC2 with RNA-binding motif 3) | 0.56 | 1.18E-06 |
| Q7Z4H3 | HDDC2 | 5'-deoxynucleotidase HDDC2 (EC 3.1.3.89) (HD domain-containing protein 2) (Hepatitis C virus NS5A-transactivated protein 2) (HCV NS5A-transactivated protein 2) | 0.56 | 5.79E-03 |
| Q8IYR2 | SMYD4 | SET and MYND domain-containing protein 4 (EC 2.1.1.-) | 0.56 | 1.31E-02 |
| Q96S97 | MYADM | Myeloid-associated differentiation marker (Protein SB135) | 0.56 | 5.48E-06 |
| Q02078 | MEF2A | Myocyte-specific enhancer factor 2A (Serum response factor-like protein 1) | 0.56 | 2.67E-04 |
| Q8N2R8 | FAM43A | Protein FAM43A | 0.56 | 4.36E-03 |
| Q86X27 | RALGPS2 | Ras-specific guanine nucleotide-releasing factor RalGPS2 (Ral GEF with PH domain and SH3-binding motif 2) (RalA exchange factor RalGPS2) | 0.56 | 1.79E-04 |
| Q9UMX5 | NENF | Neudesin (Cell immortalization-related protein 2) (Neuron-derived neurotrophic factor) (Protein GIG47) (Secreted protein of unknown function) (SPUF protein) | 0.56 | 7.69E-06 |
| Q9Y6B7 | AP4B1 | AP-4 complex subunit beta-1 (AP-4 adaptor complex subunit beta) (Adaptor-related protein complex 4 subunit beta-1) (Beta subunit of AP-4) (Beta4-adaptin) | 0.56 | 1.70E-04 |
| Q8WWI1 | LMO7 | LIM domain only protein 7 (LMO-7) (F-box only protein 20) (LOMP) | 0.56 | 3.50E-07 |
| Q08722 | CD47 | Leukocyte surface antigen CD47 (Antigenic surface determinant protein OA3) (Integrin-associated protein) (IAP) (Protein MER6) (CD antigen CD47) | 0.56 | 7.73E-05 |
| O75356 | ENTPD5 | Nucleoside diphosphate phosphatase ENTPD5 (EC 3.6.1.6) (CD39 antigen-like 4) (ER-UDPase) (Ectonucleoside triphosphate diphosphohydrolase 5) (NTPDase 5) (Guanosine-diphosphatase ENTPD5) (GDPase ENTPD5) (Inosine diphosphate phosphatase ENTPD5) (Nucleoside diphosphatase) (Uridine-diphosphatase ENTPD5) (UDPase ENTPD5) | 0.56 | 4.88E-03 |
| O95081 | AGFG2 | Arf-GAP domain and FG repeat-containing protein 2 (HIV-1 Rev-binding protein-like protein) (Rev/Rex activation domain-binding protein related) (RAB-R) | 0.56 | 1.30E-08 |
| P48454 | PPP3CC | Serine/threonine-protein phosphatase 2B catalytic subunit gamma isoform (EC 3.1.3.16) (CAM-PRP catalytic subunit) (Calcineurin, testis-specific catalytic subunit) (Calmodulin-dependent calcineurin A subunit gamma isoform) | 0.56 | 1.39E-06 |
| Q9BVT8 | TMUB1 | Transmembrane and ubiquitin-like domain-containing protein 1 (Dendritic cell-derived ubiquitin-like protein) (DULP) (Hepatocyte odd protein shuttling protein) (Ubiquitin-like protein SB144) [Cleaved into: iHOPS] | 0.56 | 1.91E-04 |
| Q969X0 | RILPL2 | RILP-like protein 2 (Rab-interacting lysosomal protein-like 2) (p40phox-binding protein) | 0.56 | 2.16E-05 |
| Q15345 | LRRC41 | Leucine-rich repeat-containing protein 41 (Protein Muf1) | 0.56 | 1.05E-02 |
| Q86XK2 | FBXO11 | F-box only protein 11 (Protein arginine N-methyltransferase 9) (Vitiligo-associated protein 1) (VIT-1) | 0.55 | 4.88E-03 |
| Q9NU53 | GINM1 | Glycoprotein integral membrane protein 1 | 0.55 | 7.93E-03 |
| Q9ULZ3 | PYCARD | Apoptosis-associated speck-like protein containing a CARD (hASC) (Caspase recruitment domain-containing protein 5) (PYD and CARD domain-containing protein) (Target of methylation-induced silencing 1) | 0.55 | 6.33E-06 |
| P54707 | ATP12A | Potassium-transporting ATPase alpha chain 2 (HK alpha 2) (Non-gastric H(+)/K(+) ATPase subunit alpha) (EC 7.2.2.19) (Non-gastric Na(+)/K(+) ATPase subunit alpha) (EC 7.2.2.13) (Proton pump) (Sodium pump) | 0.55 | 1.49E-03 |
| Q16626 | MEA1 | Male-enhanced antigen 1 (MEA-1) | 0.55 | 1.00E-02 |
| P63098 | PPP3R1 | Calcineurin subunit B type 1 (Protein phosphatase 2B regulatory subunit 1) (Protein phosphatase 3 regulatory subunit B alpha isoform 1) | 0.55 | 2.82E-03 |
| Q6PJG2 | MIDEAS | Mitotic deacetylase-associated SANT domain protein (ELM2 and SANT domain-containing protein 1) | 0.55 | 3.69E-05 |
| P10586 | PTPRF | Receptor-type tyrosine-protein phosphatase F (EC 3.1.3.48) (Leukocyte common antigen related) (LAR) | 0.55 | 1.40E-06 |
| Q9NRX4 | PHPT1 | 14 kDa phosphohistidine phosphatase (EC 3.9.1.3) (Phosphohistidine phosphatase 1) (PHPT1) (Protein histidine phosphatase) (PHP) (Protein janus-A homolog) | 0.55 | 2.75E-02 |
| Q9BRX5 | GINS3 | DNA replication complex GINS protein PSF3 (GINS complex subunit 3) | 0.55 | 4.11E-02 |
| Q16594 | TAF9 | Transcription initiation factor TFIID subunit 9 (RNA polymerase II TBP-associated factor subunit G) (STAF31/32) (Transcription initiation factor TFIID 31 kDa subunit) (TAFII-31) (TAFII31) (Transcription initiation factor TFIID 32 kDa subunit) (TAFII-32) (TAFII32) | 0.55 | 3.81E-03 |
| O95786 | DDX58 | Antiviral innate immune response receptor RIG-I (ATP-dependent RNA helicase DDX58) (EC 3.6.4.13) (DEAD box protein 58) (RIG-I-like receptor 1) (RLR-1) (RNA sensor RIG-I) (Retinoic acid-inducible gene 1 protein) (RIG-1) (Retinoic acid-inducible gene I protein) (RIG-I) | 0.55 | 1.44E-06 |
| Q9NXH8 | TOR4A | Torsin-4A (Torsin family 4 member A) | 0.54 | 1.91E-07 |
| Q0ZGT2 | NEXN | Nexilin (F-actin-binding protein) (Nelin) | 0.54 | 1.96E-08 |
| Q8NFH8 | REPS2 | RalBP1-associated Eps domain-containing protein 2 (Partner of RalBP1) (RalBP1-interacting protein 2) | 0.54 | 2.25E-02 |
| Q12797 | ASPH | Aspartyl/asparaginyl beta-hydroxylase (EC 1.14.11.16) (Aspartate beta-hydroxylase) (ASP beta-hydroxylase) (Peptide-aspartate beta-dioxygenase) | 0.54 | 4.89E-07 |
| Q9NRX5 | SERINC1 | Serine incorporator 1 (Tumor differentially expressed protein 1-like) (Tumor differentially expressed protein 2) | 0.54 | 1.90E-03 |
| Q9Y5S1 | TRPV2 | Transient receptor potential cation channel subfamily V member 2 (TrpV2) (Osm-9-like TRP channel 2) (OTRPC2) (Vanilloid receptor-like protein 1) (VRL-1) | 0.54 | 7.30E-05 |
| Q8N5N7 | MRPL50 | Large ribosomal subunit protein mL50 (39S ribosomal protein L50, mitochondrial) (L50mt) (MRP-L50) | 0.54 | 1.29E-03 |
| Q9GZM7 | TINAGL1 | Tubulointerstitial nephritis antigen-like (Glucocorticoid-inducible protein 5) (Oxidized LDL-responsive gene 2 protein) (OLRG-2) (Tubulointerstitial nephritis antigen-related protein) (TIN Ag-related protein) (TIN-Ag-RP) | 0.54 | 1.49E-02 |
| Q9BQD3 | KXD1 | KxDL motif-containing protein 1 | 0.54 | 2.59E-03 |
| P29323 | EPHB2 | Ephrin type-B receptor 2 (EC 2.7.10.1) (Developmentally-regulated Eph-related tyrosine kinase) (ELK-related tyrosine kinase) (EPH tyrosine kinase 3) (EPH-like kinase 5) (EK5) (hEK5) (Renal carcinoma antigen NY-REN-47) (Tyrosine-protein kinase TYRO5) (Tyrosine-protein kinase receptor EPH-3) [Cleaved into: EphB2/CTF1; EphB2/CTF2] | 0.54 | 2.73E-06 |
| Q7LGC8 | CHST3 | Carbohydrate sulfotransferase 3 (EC 2.8.2.17) (EC 2.8.2.21) (Chondroitin 6-O-sulfotransferase 1) (C6ST-1) (Chondroitin 6-sulfotransferase) (C6ST) (Galactose/N-acetylglucosamine/N-acetylglucosamine 6-O-sulfotransferase 0) (GST-0) | 0.53 | 5.48E-03 |
| Q9H081 | MIS12 | Protein MIS12 homolog | 0.53 | 4.93E-03 |
| Q9NWY4 | HPF1 | Histone PARylation factor 1 | 0.53 | 1.45E-02 |
| Q8TAD4 | SLC30A5 | Proton-coupled zinc antiporter SLC30A5 (Solute carrier family 30 member 5) (Zinc transporter 5) (ZnT-5) (ZnT-like transporter 1) (hZTL1) | 0.53 | 1.29E-02 |
| P62341 | SELENOT | Thioredoxin reductase-like selenoprotein T (SelT) (EC 1.8.1.9) | 0.53 | 5.61E-03 |
| Q10588 | BST1 | ADP-ribosyl cyclase/cyclic ADP-ribose hydrolase 2 (EC 3.2.2.6) (ADP-ribosyl cyclase 2) (Bone marrow stromal cell antigen 1) (BST-1) (Cyclic ADP-ribose hydrolase 2) (cADPR hydrolase 2) (CD antigen CD157) | 0.53 | 1.48E-03 |
| O60936 | NOL3 | Nucleolar protein 3 (Apoptosis repressor with CARD) (Muscle-enriched cytoplasmic protein) (Myp) (Nucleolar protein of 30 kDa) (Nop30) | 0.53 | 4.89E-02 |
| P23467 | PTPRB | Receptor-type tyrosine-protein phosphatase beta (Protein-tyrosine phosphatase beta) (R-PTP-beta) (EC 3.1.3.48) (Vascular endothelial protein tyrosine phosphatase) (VE-PTP) | 0.53 | 1.50E-08 |
| Q9H098 | FAM107B | Protein FAM107B | 0.53 | 1.10E-05 |
| P14618 | PKM | Pyruvate kinase PKM (EC 2.7.1.40) (Cytosolic thyroid hormone-binding protein) (CTHBP) (Opa-interacting protein 3) (OIP-3) (Pyruvate kinase 2/3) (Pyruvate kinase muscle isozyme) (Threonine-protein kinase PKM2) (EC 2.7.11.1) (Thyroid hormone-binding protein 1) (THBP1) (Tumor M2-PK) (Tyrosine-protein kinase PKM2) (EC 2.7.10.2) (p58) | 0.52 | 4.79E-06 |
| Q5VUJ6 | LRCH2 | Leucine-rich repeat and calponin homology domain-containing protein 2 | 0.52 | 1.92E-04 |
| Q86UX7 | FERMT3 | Fermitin family homolog 3 (Kindlin-3) (MIG2-like protein) (Unc-112-related protein 2) | 0.52 | 8.66E-06 |
| P15144 | ANPEP | Aminopeptidase N (AP-N) (hAPN) (EC 3.4.11.2) (Alanyl aminopeptidase) (Aminopeptidase M) (AP-M) (Microsomal aminopeptidase) (Myeloid plasma membrane glycoprotein CD13) (gp150) (CD antigen CD13) | 0.52 | 2.04E-05 |
| Q9H1B5 | XYLT2 | Xylosyltransferase 2 (EC 2.4.2.26) (Peptide O-xylosyltransferase 1) (Xylosyltransferase II) (XT-II) (XylT-II) | 0.52 | 7.90E-04 |
| Q9Y617 | PSAT1 | Phosphoserine aminotransferase (EC 2.6.1.52) (Phosphohydroxythreonine aminotransferase) (PSAT) | 0.52 | 5.24E-04 |
| P40261 | NNMT | Nicotinamide N-methyltransferase (EC 2.1.1.1) | 0.52 | 1.74E-04 |
| Q9BUH6 | PAXX | Protein PAXX (Paralog of XRCC4 and XLF) (XRCC4-like small protein) | 0.52 | 1.55E-04 |
| Q17RN3 | FAM98C | Protein FAM98C | 0.52 | 9.22E-03 |
| P14174 | MIF | Macrophage migration inhibitory factor (MIF) (EC 5.3.2.1) (Glycosylation-inhibiting factor) (GIF) (L-dopachrome isomerase) (L-dopachrome tautomerase) (EC 5.3.3.12) (Phenylpyruvate tautomerase) | 0.52 | 4.64E-02 |
| Q8IXU6 | SLC35F2 | Solute carrier family 35 member F2 | 0.51 | 5.02E-03 |
| Q2VPK5 | CTU2 | Cytoplasmic tRNA 2-thiolation protein 2 (Cytosolic thiouridylase subunit 2) | 0.51 | 5.66E-05 |
| Q9H4Z3 | PCIF1 | mRNA (2'-O-methyladenosine-N(6)-)-methyltransferase (EC 2.1.1.62) (Cap-specific adenosine methyltransferase) (CAPAM) (hCAPAM) (Phosphorylated CTD-interacting factor 1) (hPCIF1) (Protein phosphatase 1 regulatory subunit 121) | 0.51 | 2.04E-03 |
| O00391 | QSOX1 | Sulfhydryl oxidase 1 (hQSOX) (EC 1.8.3.2) (Quiescin Q6) | 0.51 | 8.50E-07 |
| Q96J84 | KIRREL1 | Kin of IRRE-like protein 1 (Kin of irregular chiasm-like protein 1) (Nephrin-like protein 1) | 0.51 | 1.69E-05 |
| P00558 | PGK1 | Phosphoglycerate kinase 1 (EC 2.7.2.3) (Cell migration-inducing gene 10 protein) (Primer recognition protein 2) (PRP 2) | 0.51 | 4.21E-07 |
| O00148 | DDX39A | ATP-dependent RNA helicase DDX39A (EC 3.6.4.13) (DEAD box protein 39) (Nuclear RNA helicase URH49) | 0.51 | 4.06E-02 |
| P04406 | GAPDH | Glyceraldehyde-3-phosphate dehydrogenase (GAPDH) (EC 1.2.1.12) (Peptidyl-cysteine S-nitrosylase GAPDH) (EC 2.6.99.-) | 0.51 | 4.69E-05 |
| Q9BXW6 | OSBPL1A | Oxysterol-binding protein-related protein 1 (ORP-1) (OSBP-related protein 1) | 0.51 | 2.36E-02 |
| O75506 | HSBP1 | Heat shock factor-binding protein 1 (Nasopharyngeal carcinoma-associated antigen 13) (NPC-A-13) | 0.51 | 2.26E-02 |
| Q9NQC3 | RTN4 | Reticulon-4 (Foocen) (Neurite outgrowth inhibitor) (Nogo protein) (Neuroendocrine-specific protein) (NSP) (Neuroendocrine-specific protein C homolog) (RTN-x) (Reticulon-5) | 0.51 | 7.15E-03 |
| Q8NF91 | SYNE1 | Nesprin-1 (Enaptin) (KASH domain-containing protein 1) (KASH1) (Myocyte nuclear envelope protein 1) (Myne-1) (Nuclear envelope spectrin repeat protein 1) (Synaptic nuclear envelope protein 1) (Syne-1) | 0.51 | 1.14E-08 |
| O94875 | SORBS2 | Sorbin and SH3 domain-containing protein 2 (Arg-binding protein 2) (ArgBP2) (Arg/Abl-interacting protein 2) (Sorbin) | 0.51 | 1.65E-05 |
| P62330 | ARF6 | ADP-ribosylation factor 6 (EC 3.6.5.2) | 0.50 | 5.64E-03 |
| Q7Z449 | CYP2U1 | Cytochrome P450 2U1 (Long-chain fatty acid omega-monooxygenase) (EC 1.14.14.80) | 0.50 | 2.58E-03 |
| Q9P273 | TENM3 | Teneurin-3 (Ten-3) (Protein Odd Oz/ten-m homolog 3) (Tenascin-M3) (Ten-m3) (Teneurin transmembrane protein 3) | 0.50 | 2.91E-06 |
| Q8NI08 | NCOA7 | Nuclear receptor coactivator 7 (140 kDa estrogen receptor-associated protein) (Estrogen nuclear receptor coactivator 1) | 0.50 | 3.77E-02 |
| O43166 | SIPA1L1 | Signal-induced proliferation-associated 1-like protein 1 (SIPA1-like protein 1) (High-risk human papilloma viruses E6 oncoproteins targeted protein 1) (E6-targeted protein 1) | 0.50 | 1.38E-04 |
| P0CAP2 | POLR2M | DNA-directed RNA polymerase II subunit GRINL1A (DNA-directed RNA polymerase II subunit M) (Glutamate receptor-like protein 1A) | 0.50 | 5.98E-03 |
| O75164 | KDM4A | Lysine-specific demethylase 4A (EC 1.14.11.66) (EC 1.14.11.69) (JmjC domain-containing histone demethylation protein 3A) (Jumonji domain-containing protein 2A) ([histone H3]-trimethyl-L-lysine(36) demethylase 4A) ([histone H3]-trimethyl-L-lysine(9) demethylase 4A) | 0.50 | 7.37E-03 |
| P49642 | PRIM1 | DNA primase small subunit (EC 2.7.7.102) (DNA primase 49 kDa subunit) (p49) | 0.50 | 1.84E-02 |
| Q8WV41 | SNX33 | Sorting nexin-33 (SH3 and PX domain-containing protein 3) | 0.50 | 9.37E-06 |
| Q9UKI2 | CDC42EP3 | Cdc42 effector protein 3 (Binder of Rho GTPases 2) (MSE55-related Cdc42-binding protein) | 0.50 | 8.50E-03 |
| Q8IVB5 | LIX1L | LIX1-like protein | 0.50 | 1.05E-03 |
| Q15427 | SF3B4 | Splicing factor 3B subunit 4 (Pre-mRNA-splicing factor SF3b 49 kDa subunit) (Spliceosome-associated protein 49) (SAP 49) | 0.49 | 1.25E-03 |
| O60449 | LY75 | Lymphocyte antigen 75 (Ly-75) (C-type lectin domain family 13 member B) (DEC-205) (gp200-MR6) (CD antigen CD205) | 0.49 | 6.22E-04 |
| Q53TN4 | CYBRD1 | Plasma membrane ascorbate-dependent reductase CYBRD1 (EC 7.2.1.3) (Cytochrome b reductase 1) (Duodenal cytochrome b) (Ferric-chelate reductase 3) | 0.49 | 9.59E-05 |
| Q9UP95 | SLC12A4 | Solute carrier family 12 member 4 (Electroneutral potassium-chloride cotransporter 1) (Erythroid K-Cl cotransporter 1) (hKCC1) | 0.49 | 7.67E-05 |
| Q9H6B4 | CLMP | CXADR-like membrane protein (Adipocyte adhesion molecule) (Coxsackie- and adenovirus receptor-like membrane protein) (CAR-like membrane protein) | 0.49 | 1.52E-02 |
| P06744 | GPI | Glucose-6-phosphate isomerase (GPI) (EC 5.3.1.9) (Autocrine motility factor) (AMF) (Neuroleukin) (NLK) (Phosphoglucose isomerase) (PGI) (Phosphohexose isomerase) (PHI) (Sperm antigen 36) (SA-36) | 0.49 | 1.24E-04 |
| P60033 | CD81 | CD81 antigen (26 kDa cell surface protein TAPA-1) (Target of the antiproliferative antibody 1) (Tetraspanin-28) (Tspan-28) (CD antigen CD81) | 0.49 | 2.75E-05 |
| Q5K651 | SAMD9 | Sterile alpha motif domain-containing protein 9 (SAM domain-containing protein 9) | 0.49 | 1.32E-05 |
| Q9BW60 | ELOVL1 | Elongation of very long chain fatty acids protein 1 (EC 2.3.1.199) (3-keto acyl-CoA synthase ELOVL1) (ELOVL fatty acid elongase 1) (ELOVL FA elongase 1) (Very long chain 3-ketoacyl-CoA synthase 1) (Very long chain 3-oxoacyl-CoA synthase 1) | 0.48 | 3.27E-02 |
| O60551 | NMT2 | Glycylpeptide N-tetradecanoyltransferase 2 (EC 2.3.1.97) (Myristoyl-CoA:protein N-myristoyltransferase 2) (NMT 2) (Peptide N-myristoyltransferase 2) (Protein-lysine myristoyltransferase NMT2) (EC 2.3.1.-) (Type II N-myristoyltransferase) | 0.48 | 1.41E-04 |
| P60174 | TPI1 | Triosephosphate isomerase (TIM) (EC 5.3.1.1) (Methylglyoxal synthase) (EC 4.2.3.3) (Triose-phosphate isomerase) | 0.48 | 8.39E-03 |
| P78330 | PSPH | Phosphoserine phosphatase (PSP) (PSPase) (EC 3.1.3.3) (L-3-phosphoserine phosphatase) (O-phosphoserine phosphohydrolase) | 0.48 | 1.11E-05 |
| Q14678 | KANK1 | KN motif and ankyrin repeat domain-containing protein 1 (Ankyrin repeat domain-containing protein 15) (Kidney ankyrin repeat-containing protein) | 0.48 | 1.67E-08 |
| Q16555 | DPYSL2 | Dihydropyrimidinase-related protein 2 (DRP-2) (Collapsin response mediator protein 2) (CRMP-2) (N2A3) (Unc-33-like phosphoprotein 2) (ULIP-2) | 0.48 | 1.04E-07 |
| Q99685 | MGLL | Monoglyceride lipase (MGL) (EC 3.1.1.23) (HU-K5) (Lysophospholipase homolog) (Lysophospholipase-like) (Monoacylglycerol lipase) (MAGL) | 0.48 | 1.13E-02 |
| Q92743 | HTRA1 | Serine protease HTRA1 (EC 3.4.21.-) (High-temperature requirement A serine peptidase 1) (L56) (Serine protease 11) | 0.48 | 4.41E-04 |
| Q15582 | TGFBI | Transforming growth factor-beta-induced protein ig-h3 (Beta ig-h3) (Kerato-epithelin) (RGD-containing collagen-associated protein) (RGD-CAP) | 0.48 | 1.27E-04 |
| P35613 | BSG | Basigin (5F7) (Collagenase stimulatory factor) (Extracellular matrix metalloproteinase inducer) (EMMPRIN) (Hepatoma-associated antigen) (HAb18G) (Leukocyte activation antigen M6) (OK blood group antigen) (Tumor cell-derived collagenase stimulatory factor) (TCSF) (CD antigen CD147) | 0.48 | 1.19E-03 |
| P10301 | RRAS | Ras-related protein R-Ras (EC 3.6.5.-) (p23) | 0.48 | 2.88E-03 |
| P15151 | PVR | Poliovirus receptor (Nectin-like protein 5) (NECL-5) (CD antigen CD155) | 0.48 | 1.71E-05 |
| Q9NY65 | TUBA8 | Tubulin alpha-8 chain (EC 3.6.5.-) (Alpha-tubulin 8) (Tubulin alpha chain-like 2) [Cleaved into: Dephenylalaninated tubulin alpha-8 chain] | 0.48 | 5.00E-02 |
| Q9UIW2 | PLXNA1 | Plexin-A1 (Semaphorin receptor NOV) | 0.48 | 3.89E-05 |
| Q99622 | C12orf57 | Protein C10 | 0.48 | 1.29E-03 |
| P05412 | JUN | Transcription factor Jun (Activator protein 1) (AP1) (Proto-oncogene c-Jun) (Transcription factor AP-1 subunit Jun) (V-jun avian sarcoma virus 17 oncogene homolog) (p39) | 0.48 | 2.82E-04 |
| P13196 | ALAS1 | 5-aminolevulinate synthase, non-specific, mitochondrial (ALAS-H) (EC 2.3.1.37) (5-aminolevulinic acid synthase 1) (Delta-ALA synthase 1) (Delta-aminolevulinate synthase 1) | 0.47 | 6.15E-03 |
| P08582 | MELTF | Melanotransferrin (Melanoma-associated antigen p97) (CD antigen CD228) | 0.47 | 2.45E-03 |
| Q9HB19 | PLEKHA2 | Pleckstrin homology domain-containing family A member 2 (PH domain-containing family A member 2) (Tandem PH domain-containing protein 2) (TAPP-2) | 0.47 | 7.13E-03 |
| Q8NE01 | CNNM3 | Metal transporter CNNM3 (Ancient conserved domain-containing protein 3) (Cyclin-M3) | 0.47 | 1.90E-05 |
| Q8IXQ6 | PARP9 | Protein mono-ADP-ribosyltransferase PARP9 (EC 2.4.2.-) (ADP-ribosyltransferase diphtheria toxin-like 9) (ARTD9) (B aggressive lymphoma protein) (Poly [ADP-ribose] polymerase 9) (PARP-9) | 0.47 | 1.41E-04 |
| Q9ULC3 | RAB23 | Ras-related protein Rab-23 | 0.47 | 1.06E-05 |
| P02768 | ALB | Albumin | 0.47 | 1.89E-04 |
| O60674 | JAK2 | Tyrosine-protein kinase JAK2 (EC 2.7.10.2) (Janus kinase 2) (JAK-2) | 0.47 | 1.11E-03 |
| Q99584 | S100A13 | Protein S100-A13 (S100 calcium-binding protein A13) | 0.47 | 1.16E-03 |
| Q969Z3 | MTARC2 | Mitochondrial amidoxime reducing component 2 (mARC2) (EC 1.7.-.-) (Molybdenum cofactor sulfurase C-terminal domain-containing protein 2) (MOSC domain-containing protein 2) (Moco sulfurase C-terminal domain-containing protein 2) | 0.47 | 8.11E-04 |
| P05121 | SERPINE1 | Plasminogen activator inhibitor 1 (PAI) (PAI-1) (Endothelial plasminogen activator inhibitor) (Serpin E1) | 0.46 | 1.75E-05 |
| Q8NDH3 | NPEPL1 | Probable aminopeptidase NPEPL1 (EC 3.4.11.-) (Aminopeptidase-like 1) | 0.46 | 4.83E-04 |
| Q14703 | MBTPS1 | Membrane-bound transcription factor site-1 protease (EC 3.4.21.112) (Endopeptidase S1P) (Subtilisin/kexin-isozyme 1) (SKI-1) | 0.46 | 1.86E-02 |
| Q86V88 | MDP1 | Magnesium-dependent phosphatase 1 (MDP-1) (EC 3.1.3.-) (EC 3.1.3.48) | 0.46 | 1.93E-04 |
| Q6NXE6 | ARMC6 | Armadillo repeat-containing protein 6 | 0.46 | 2.34E-02 |
| P05023 | ATP1A1 | Sodium/potassium-transporting ATPase subunit alpha-1 (Na(+)/K(+) ATPase alpha-1 subunit) (EC 7.2.2.13) (Sodium pump subunit alpha-1) | 0.46 | 3.86E-07 |
| Q8N8L6 | ARL10 | ADP-ribosylation factor-like protein 10 | 0.46 | 4.51E-06 |
| P43378 | PTPN9 | Tyrosine-protein phosphatase non-receptor type 9 (EC 3.1.3.48) (Protein-tyrosine phosphatase MEG2) (PTPase MEG2) | 0.46 | 3.17E-03 |
| Q8NAP3 | ZBTB38 | Zinc finger and BTB domain-containing protein 38 | 0.46 | 2.37E-02 |
| Q9NPD8 | UBE2T | Ubiquitin-conjugating enzyme E2 T (EC 2.3.2.23) (Cell proliferation-inducing gene 50 protein) (E2 ubiquitin-conjugating enzyme T) (Ubiquitin carrier protein T) (Ubiquitin-protein ligase T) | 0.46 | 4.99E-02 |
| Q5T5U3 | ARHGAP21 | Rho GTPase-activating protein 21 (Rho GTPase-activating protein 10) (Rho-type GTPase-activating protein 21) | 0.46 | 2.54E-04 |
| P49768 | PSEN1 | Presenilin-1 (PS-1) (EC 3.4.23.-) (Protein S182) [Cleaved into: Presenilin-1 NTF subunit; Presenilin-1 CTF subunit; Presenilin-1 CTF12 (PS1-CTF12)] | 0.46 | 1.50E-04 |
| P05387 | RPLP2 | Large ribosomal subunit protein P2 (60S acidic ribosomal protein P2) (Renal carcinoma antigen NY-REN-44) | 0.46 | 1.62E-03 |
| O43741 | PRKAB2 | 5'-AMP-activated protein kinase subunit beta-2 (AMPK subunit beta-2) | 0.46 | 1.54E-03 |
| Q9Y5Q0 | FADS3 | Fatty acid desaturase 3 (EC 1.14.19.-) (Delta(13) fatty acid desaturase) (Delta(13) desaturase) | 0.46 | 2.80E-02 |
| Q9BX59 | TAPBPL | Tapasin-related protein (TAPASIN-R) (TAP-binding protein-like) (TAP-binding protein-related protein) (TAPBP-R) (Tapasin-like) | 0.45 | 1.38E-02 |
| P16070 | CD44 | CD44 antigen (CDw44) (Epican) (Extracellular matrix receptor III) (ECMR-III) (GP90 lymphocyte homing/adhesion receptor) (HUTCH-I) (Heparan sulfate proteoglycan) (Hermes antigen) (Hyaluronate receptor) (Phagocytic glycoprotein 1) (PGP-1) (Phagocytic glycoprotein I) (PGP-I) (CD antigen CD44) | 0.45 | 3.32E-04 |
| Q7Z7A3 | CTU1 | Cytoplasmic tRNA 2-thiolation protein 1 (EC 2.7.7.-) (ATP-binding domain-containing protein 3) (Cancer-associated gene protein) (Cytoplasmic tRNA adenylyltransferase 1) | 0.45 | 2.53E-02 |
| Q9UMS0 | NFU1 | NFU1 iron-sulfur cluster scaffold homolog, mitochondrial (HIRA-interacting protein 5) | 0.45 | 7.13E-03 |
| O76081 | RGS20 | Regulator of G-protein signaling 20 (RGS20) (Gz-selective GTPase-activating protein) (G(z)GAP) (Gz-GAP) (Regulator of G-protein signaling Z1) (Regulator of Gz-selective protein signaling 1) | 0.45 | 1.18E-02 |
| Q14643 | ITPR1 | Inositol 1,4,5-trisphosphate receptor type 1 (IP3 receptor isoform 1) (IP3R 1) (InsP3R1) (Type 1 inositol 1,4,5-trisphosphate receptor) (Type 1 InsP3 receptor) | 0.45 | 3.21E-05 |
| Q969G5 | CAVIN3 | Caveolae-associated protein 3 (Cavin-3) (Protein kinase C delta-binding protein) (Serum deprivation response factor-related gene product that binds to C-kinase) (hSRBC) | 0.45 | 2.65E-06 |
| Q96EI5 | TCEAL4 | Transcription elongation factor A protein-like 4 (TCEA-like protein 4) (Transcription elongation factor S-II protein-like 4) | 0.45 | 1.09E-02 |
| O15031 | PLXNB2 | Plexin-B2 (MM1) | 0.45 | 4.46E-08 |
| Q9NRF2 | SH2B1 | SH2B adapter protein 1 (Pro-rich, PH and SH2 domain-containing signaling mediator) (PSM) (SH2 domain-containing protein 1B) | 0.45 | 3.10E-02 |
| Q9P2E7 | PCDH10 | Protocadherin-10 | 0.45 | 2.65E-04 |
| Q9NXW9 | ALKBH4 | Alpha-ketoglutarate-dependent dioxygenase alkB homolog 4 (Alkylated DNA repair protein alkB homolog 4) (DNA N6-methyl adenine demethylase ALKBH4) (EC 1.14.11.51) (Lysine-specific demethylase ALKBH4) (EC 1.14.11.-) | 0.44 | 1.23E-04 |
| P29317 | EPHA2 | Ephrin type-A receptor 2 (EC 2.7.10.1) (Epithelial cell kinase) (Tyrosine-protein kinase receptor ECK) | 0.44 | 6.41E-06 |
| O75828 | CBR3 | Carbonyl reductase [NADPH] 3 (EC 1.1.1.184) (NADPH-dependent carbonyl reductase 3) (Quinone reductase CBR3) (EC 1.6.5.10) (Short chain dehydrogenase/reductase family 21C member 2) | 0.44 | 9.33E-04 |
| Q6UWY0 | ARSK | Arylsulfatase K (ASK) (EC 3.1.6.1) (Glucuronate-2-sulfatase) (EC 3.1.6.18) (Telethon sulfatase) | 0.44 | 1.52E-02 |
| P53365 | ARFIP2 | Arfaptin-2 (ADP-ribosylation factor-interacting protein 2) (Partner of RAC1) (POR1) | 0.44 | 7.07E-04 |
| Q9P2N6 | KANSL3 | KAT8 regulatory NSL complex subunit 3 (NSL complex protein NSL3) (Non-specific lethal 3 homolog) (Serum inhibited-related protein) (Testis development protein PRTD) | 0.44 | 1.30E-02 |
| Q9NRQ2 | PLSCR4 | Phospholipid scramblase 4 (PL scramblase 4) (Ca(2+)-dependent phospholipid scramblase 4) (Cell growth-inhibiting gene 43 protein) (TRA1) | 0.44 | 3.60E-03 |
| Q96GD0 | PDXP | Chronophin (EC 3.1.3.16) (EC 3.1.3.74) (Pyridoxal phosphate phosphatase) (PLP phosphatase) | 0.44 | 7.91E-04 |
| Q92759 | GTF2H4 | General transcription factor IIH subunit 4 (Basic transcription factor 2 52 kDa subunit) (BTF2 p52) (General transcription factor IIH polypeptide 4) (TFIIH basal transcription factor complex p52 subunit) | 0.44 | 3.61E-02 |
| Q92508 | PIEZO1 | Piezo-type mechanosensitive ion channel component 1 (Membrane protein induced by beta-amyloid treatment) (Mib) (Protein FAM38A) | 0.44 | 7.33E-07 |
| P21810 | BGN | Biglycan (Bone/cartilage proteoglycan I) (PG-S1) | 0.44 | 1.32E-02 |
| Q6P6C2 | ALKBH5 | RNA demethylase ALKBH5 (EC 1.14.11.53) (Alkylated DNA repair protein alkB homolog 5) (Alpha-ketoglutarate-dependent dioxygenase alkB homolog 5) | 0.43 | 9.14E-05 |
| Q9NP58 | ABCB6 | ATP-binding cassette sub-family B member 6 (ABC-type heme transporter ABCB6) (EC 7.6.2.5) (Mitochondrial ABC transporter 3) (Mt-ABC transporter 3) (P-glycoprotein-related protein) (Ubiquitously-expressed mammalian ABC half transporter) | 0.43 | 1.46E-03 |
| Q9BTT4 | MED10 | Mediator of RNA polymerase II transcription subunit 10 (Mediator complex subunit 10) (Transformation-related gene 17 protein) (TRG-17) (Transformation-related gene 20 protein) (TRG-20) | 0.43 | 6.93E-03 |
| Q8N9N7 | LRRC57 | Leucine-rich repeat-containing protein 57 | 0.43 | 4.59E-03 |
| Q8IYU2 | HACE1 | E3 ubiquitin-protein ligase HACE1 (EC 2.3.2.26) (HECT domain and ankyrin repeat-containing E3 ubiquitin-protein ligase 1) (HECT-type E3 ubiquitin transferase HACE1) | 0.43 | 1.23E-03 |
| Q9HB21 | PLEKHA1 | Pleckstrin homology domain-containing family A member 1 (PH domain-containing family A member 1) (Tandem PH domain-containing protein 1) (TAPP-1) | 0.43 | 2.09E-03 |
| A0PJW6 | TMEM223 | Transmembrane protein 223 | 0.43 | 1.66E-02 |
| Q07820 | MCL1 | Induced myeloid leukemia cell differentiation protein Mcl-1 (Bcl-2-like protein 3) (Bcl2-L-3) (Bcl-2-related protein EAT/mcl1) (mcl1/EAT) | 0.43 | 2.85E-02 |
| Q9BX10 | GTPBP2 | GTP-binding protein 2 | 0.43 | 8.55E-03 |
| Q9NYT0 | PLEK2 | Pleckstrin-2 | 0.43 | 6.54E-05 |
| Q9NRY6 | PLSCR3 | Phospholipid scramblase 3 (PL scramblase 3) (Ca(2+)-dependent phospholipid scramblase 3) | 0.43 | 5.30E-03 |
| Q14191 | WRN | Bifunctional 3'-5' exonuclease/ATP-dependent helicase WRN (DNA helicase, RecQ-like type 3) (RecQ protein-like 2) (Werner syndrome protein) [Includes: 3'-5' exonuclease (EC 3.1.-.-); ATP-dependent helicase (EC 3.6.4.12)] | 0.43 | 1.35E-02 |
| O43809 | NUDT21 | Cleavage and polyadenylation specificity factor subunit 5 (Cleavage and polyadenylation specificity factor 25 kDa subunit) (CPSF 25 kDa subunit) (Cleavage factor Im complex 25 kDa subunit) (CFIm25) (Nucleoside diphosphate-linked moiety X motif 21) (Nudix motif 21) (Nudix hydrolase 21) (Pre-mRNA cleavage factor Im 68 kDa subunit) | 0.43 | 2.21E-03 |
| Q8TEA8 | DTD1 | D-aminoacyl-tRNA deacylase 1 (DTD) (EC 3.1.1.96) (DNA-unwinding element-binding protein B) (DUE-B) (Gly-tRNA(Ala) deacylase) (Histidyl-tRNA synthase-related) | 0.43 | 3.32E-02 |
| P18669 | PGAM1 | Phosphoglycerate mutase 1 (EC 5.4.2.11) (EC 5.4.2.4) (BPG-dependent PGAM 1) (Phosphoglycerate mutase isozyme B) (PGAM-B) | 0.43 | 7.27E-03 |
| Q99959 | PKP2 | Plakophilin-2 | 0.43 | 1.89E-04 |
| P78324 | SIRPA | Tyrosine-protein phosphatase non-receptor type substrate 1 (SHP substrate 1) (SHPS-1) (Brain Ig-like molecule with tyrosine-based activation motifs) (Bit) (CD172 antigen-like family member A) (Inhibitory receptor SHPS-1) (Macrophage fusion receptor) (MyD-1 antigen) (Signal-regulatory protein alpha-1) (Sirp-alpha-1) (Signal-regulatory protein alpha-2) (Sirp-alpha-2) (Signal-regulatory protein alpha-3) (Sirp-alpha-3) (p84) (CD antigen CD172a) | 0.43 | 8.94E-04 |
| Q9Y2G3 | ATP11B | Phospholipid-transporting ATPase IF (EC 7.6.2.1) (ATPase IR) (ATPase class VI type 11B) (P4-ATPase flippase complex alpha subunit ATP11B) | 0.43 | 5.23E-03 |
| Q8TCG2 | PI4K2B | Phosphatidylinositol 4-kinase type 2-beta (EC 2.7.1.67) (Phosphatidylinositol 4-kinase type II-beta) (PI4KII-BETA) | 0.43 | 4.96E-02 |
| Q6GMV3 | PTRHD1 | Putative peptidyl-tRNA hydrolase PTRHD1 (EC 3.1.1.29) (Peptidyl-tRNA hydrolase domain-containing protein 1) | 0.43 | 1.27E-02 |
| Q9BWL3 | C1orf43 | Protein C1orf43 (Hepatitis C virus NS5A-transactivated protein 4) (HCV NS5A-transactivated protein 4) (Protein NICE-3) (S863-3) | 0.42 | 1.40E-02 |
| P78537 | BLOC1S1 | Biogenesis of lysosome-related organelles complex 1 subunit 1 (BLOC-1 subunit 1) (GCN5-like protein 1) (Protein RT14) | 0.42 | 4.27E-04 |
| O60504 | SORBS3 | Vinexin (SH3-containing adapter molecule 1) (SCAM-1) (Sorbin and SH3 domain-containing protein 3) | 0.42 | 1.23E-05 |
| Q6P179 | ERAP2 | Endoplasmic reticulum aminopeptidase 2 (EC 3.4.11.-) (Leukocyte-derived arginine aminopeptidase) (L-RAP) | 0.42 | 1.66E-04 |
| Q9BXS9 | SLC26A6 | Solute carrier family 26 member 6 (Anion exchange transporter) (Pendrin-like protein 1) (Pendrin-L1) | 0.42 | 4.51E-02 |
| P06733 | ENO1 | Alpha-enolase (EC 4.2.1.11) (2-phospho-D-glycerate hydro-lyase) (C-myc promoter-binding protein) (Enolase 1) (MBP-1) (MPB-1) (Non-neural enolase) (NNE) (Phosphopyruvate hydratase) (Plasminogen-binding protein) | 0.42 | 7.22E-04 |
| Q9Y484 | WDR45 | WD repeat domain phosphoinositide-interacting protein 4 (WIPI-4) (WD repeat-containing protein 45) | 0.42 | 7.42E-04 |
| Q6NXT6 | TAPT1 | Transmembrane anterior posterior transformation protein 1 homolog (Cytomegalovirus partial fusion receptor) | 0.42 | 3.19E-04 |
| Q86YS7 | C2CD5 | C2 domain-containing protein 5 (C2 domain-containing phosphoprotein of 138 kDa) | 0.42 | 2.57E-05 |
| Q9NUL5 | SHFL | Shiftless antiviral inhibitor of ribosomal frameshifting protein (SFL) (SHFL) (Interferon-regulated antiviral protein) (IRAV) (Repressor of yield of DENV protein) (RyDEN) | 0.42 | 2.59E-03 |
| P35749 | MYH11 | Myosin-11 (Myosin heavy chain 11) (Myosin heavy chain, smooth muscle isoform) (SMMHC) | 0.42 | 3.83E-02 |
| P30740 | SERPINB1 | Leukocyte elastase inhibitor (LEI) (Monocyte/neutrophil elastase inhibitor) (EI) (M/NEI) (Peptidase inhibitor 2) (PI-2) (Serpin B1) | 0.42 | 6.46E-05 |
| Q92908 | GATA6 | Transcription factor GATA-6 (GATA-binding factor 6) | 0.42 | 1.05E-02 |
| Q9NP97 | DYNLRB1 | Dynein light chain roadblock-type 1 (Bithoraxoid-like protein) (BLP) (Dynein light chain 2A, cytoplasmic) (Dynein-associated protein Km23) (Roadblock domain-containing protein 1) | 0.42 | 3.05E-03 |
| P82909 | MRPS36 | Alpha-ketoglutarate dehydrogenase component 4 | 0.42 | 1.47E-02 |
| Q96RN5 | MED15 | Mediator of RNA polymerase II transcription subunit 15 (Activator-recruited cofactor 105 kDa component) (ARC105) (CTG repeat protein 7a) (Mediator complex subunit 15) (Positive cofactor 2 glutamine/Q-rich-associated protein) (PC2 glutamine/Q-rich-associated protein) (TPA-inducible gene 1 protein) (TIG-1) (Trinucleotide repeat-containing gene 7 protein) | 0.42 | 9.81E-04 |
| Q8N8Z6 | DCBLD1 | Discoidin, CUB and LCCL domain-containing protein 1 | 0.42 | 6.64E-04 |
| Q9BX68 | HINT2 | Adenosine 5'-monophosphoramidase HINT2 (EC 3.9.1.-) (HINT-3) (HIT-17kDa) (Histidine triad nucleotide-binding protein 2, mitochondrial) (HINT-2) (PKCI-1-related HIT protein) | 0.42 | 6.18E-03 |
| P23610 | F8A1 | 40-kDa huntingtin-associated protein (HAP40) (CpG island protein) (Factor VIII intron 22 protein) | 0.42 | 1.47E-02 |
| Q7KYR7 | BTN2A1 | Butyrophilin subfamily 2 member A1 | 0.42 | 1.84E-03 |
| Q9UGL1 | KDM5B | Lysine-specific demethylase 5B (EC 1.14.11.67) (Cancer/testis antigen 31) (CT31) (Histone demethylase JARID1B) (Jumonji/ARID domain-containing protein 1B) (PLU-1) (Retinoblastoma-binding protein 2 homolog 1) (RBP2-H1) ([histone H3]-trimethyl-L-lysine(4) demethylase 5B) | 0.42 | 1.09E-03 |
| P20340 | RAB6A | Ras-related protein Rab-6A (Rab-6) | 0.42 | 7.49E-03 |
| Q9H3H3 | C11orf68 | UPF0696 protein C11orf68 (Basophilic leukemia-expressed protein Bles03) (Protein p5326) | 0.42 | 6.93E-05 |
| Q8WZ82 | OVCA2 | Esterase OVCA2 (EC 3.1.2.-) (Ovarian cancer-associated gene 2 protein) | 0.41 | 3.10E-02 |
| Q969J3 | BORCS5 | BLOC-1-related complex subunit 5 (Loss of heterozygosity 12 chromosomal region 1) (Myristoylated lysosomal protein) (Myrlysin) | 0.41 | 3.76E-05 |
| Q53EZ4 | CEP55 | Centrosomal protein of 55 kDa (Cep55) (Up-regulated in colon cancer 6) | 0.41 | 4.82E-03 |
| Q7Z6I8 | C5orf24 | UPF0461 protein C5orf24 | 0.41 | 2.53E-02 |
| Q9UBQ6 | EXTL2 | Exostosin-like 2 (EC 2.4.1.223) (Alpha-1,4-N-acetylhexosaminyltransferase EXTL2) (Alpha-GalNAcT EXTL2) (EXT-related protein 2) (Glucuronyl-galactosyl-proteoglycan 4-alpha-N-acetylglucosaminyltransferase) [Cleaved into: Processed exostosin-like 2] | 0.41 | 1.09E-02 |
| Q7LBR1 | CHMP1B | Charged multivesicular body protein 1b (CHMP1.5) (Chromatin-modifying protein 1b) (CHMP1b) (Vacuolar protein sorting-associated protein 46-2) (Vps46-2) (hVps46-2) | 0.41 | 1.80E-03 |
| Q9UFW8 | CGGBP1 | CGG triplet repeat-binding protein 1 (CGG-binding protein 1) (20 kDa CGG-binding protein) (p20-CGGBP DNA-binding protein) | 0.41 | 8.24E-04 |
| O15460 | P4HA2 | Prolyl 4-hydroxylase subunit alpha-2 (4-PH alpha-2) (EC 1.14.11.2) (Procollagen-proline,2-oxoglutarate-4-dioxygenase subunit alpha-2) | 0.41 | 3.33E-05 |
| Q4KMQ2 | ANO6 | Anoctamin-6 (Small-conductance calcium-activated nonselective cation channel) (SCAN channel) (Transmembrane protein 16F) | 0.41 | 1.04E-04 |
| Q9Y5W9 | SNX11 | Sorting nexin-11 | 0.41 | 2.31E-03 |
| Q9Y5L0 | TNPO3 | Transportin-3 (Importin-12) (Imp12) (Transportin-SR) (TRN-SR) | 0.41 | 2.76E-02 |
| Q8IVF2 | AHNAK2 | Protein AHNAK2 | 0.41 | 7.97E-07 |
| Q13563 | PKD2 | Polycystin-2 (PC2) (Autosomal dominant polycystic kidney disease type II protein) (Polycystic kidney disease 2 protein) (Polycystwin) (R48321) (Transient receptor potential cation channel subfamily P member 2) | 0.41 | 1.35E-03 |
| P47224 | RABIF | Guanine nucleotide exchange factor MSS4 (Rab-interacting factor) | 0.41 | 5.86E-04 |
| Q9BZF9 | UACA | Uveal autoantigen with coiled-coil domains and ankyrin repeats | 0.41 | 6.70E-07 |
| O95760 | IL33 | Interleukin-33 (IL-33) (Interleukin-1 family member 11) (IL-1F11) (Nuclear factor from high endothelial venules) (NF-HEV) [Cleaved into: Interleukin-33 (95-270); Interleukin-33 (99-270); Interleukin-33 (109-270)] | 0.41 | 1.22E-03 |
| Q8NHP8 | PLBD2 | Putative phospholipase B-like 2 (EC 3.1.1.-) (76 kDa protein) (p76) (LAMA-like protein 2) (Lamina ancestor homolog 2) (Phospholipase B domain-containing protein 2) [Cleaved into: Putative phospholipase B-like 2 32 kDa form; Putative phospholipase B-like 2 45 kDa form] | 0.41 | 2.25E-03 |
| Q9H7F0 | ATP13A3 | Polyamine-transporting ATPase 13A3 (ATPase family homolog up-regulated in senescence cells 1) (Putrescine transporting ATPase) (EC 7.6.2.16) | 0.41 | 6.53E-05 |
| O00762 | UBE2C | Ubiquitin-conjugating enzyme E2 C (EC 2.3.2.23) ((E3-independent) E2 ubiquitin-conjugating enzyme C) (EC 2.3.2.24) (E2 ubiquitin-conjugating enzyme C) (UbcH10) (Ubiquitin carrier protein C) (Ubiquitin-protein ligase C) | 0.41 | 3.28E-02 |
| Q8TD55 | PLEKHO2 | Pleckstrin homology domain-containing family O member 2 (PH domain-containing family O member 2) (Pleckstrin homology domain-containing family Q member 1) (PH domain-containing family Q member 1) | 0.41 | 3.54E-04 |
| P56385 | ATP5ME | ATP synthase subunit e, mitochondrial (ATPase subunit e) (ATP synthase membrane subunit e) [Cleaved into: ATP synthase subunit e, mitochondrial, N-terminally processed] | 0.40 | 1.25E-02 |
| P04632 | CAPNS1 | Calpain small subunit 1 (CSS1) (Calcium-activated neutral proteinase small subunit) (CANP small subunit) (Calcium-dependent protease small subunit) (CDPS) (Calcium-dependent protease small subunit 1) (Calpain regulatory subunit) | 0.40 | 1.23E-05 |
| Q643R3 | LPCAT4 | Lysophospholipid acyltransferase LPCAT4 (1-acylglycerol-3-phosphate O-acyltransferase 7) (1-AGP acyltransferase 7) (1-AGPAT 7) (1-acylglycerophosphocholine O-acyltransferase) (EC 2.3.1.23) (1-acylglycerophosphoserine O-acyltransferase) (EC 2.3.1.n6) (1-alkenylglycerophosphoethanolamine O-acyltransferase) (EC 2.3.1.121) (1-alkylglycerophosphocholine O-acetyltransferase) (EC 2.3.1.67) (Acyltransferase-like 3) (Lysophosphatidylcholine acyltransferase 4) (Lysophosphatidylethanolamine acyltransferase 2) (EC 2.3.1.n7) (Plasmalogen synthase) | 0.40 | 1.43E-04 |
| O43813 | LANCL1 | Glutathione S-transferase LANCL1 (EC 2.5.1.18) (40 kDa erythrocyte membrane protein) (p40) (LanC-like protein 1) | 0.40 | 2.31E-02 |
| Q15173 | PPP2R5B | Serine/threonine-protein phosphatase 2A 56 kDa regulatory subunit beta isoform (PP2A B subunit isoform B'-beta) (PP2A B subunit isoform B56-beta) (PP2A B subunit isoform PR61-beta) (PP2A B subunit isoform R5-beta) | 0.40 | 2.94E-03 |
| P26006 | ITGA3 | Integrin alpha-3 (CD49 antigen-like family member C) (FRP-2) (Galactoprotein B3) (GAPB3) (VLA-3 subunit alpha) (CD antigen CD49c) [Cleaved into: Integrin alpha-3 heavy chain; Integrin alpha-3 light chain] | 0.40 | 2.56E-04 |
| Q8N3V7 | SYNPO | Synaptopodin | 0.40 | 2.10E-05 |
| Q9P2K6 | KLHL42 | Kelch-like protein 42 (Cullin-3-binding protein 9) (Ctb9) (Kelch domain-containing protein 5) | 0.40 | 3.97E-04 |
| O00622 | CCN1 | CCN family member 1 (Cellular communication network factor 1) (Cysteine-rich angiogenic inducer 61) (Insulin-like growth factor-binding protein 10) (IBP-10) (IGF-binding protein 10) (IGFBP-10) (Protein CYR61) (Protein GIG1) | 0.40 | 9.71E-04 |
| Q9BZG1 | RAB34 | Ras-related protein Rab-34 (Ras-related protein Rab-39) (Ras-related protein Rah) | 0.40 | 3.65E-03 |
| Q969X5 | ERGIC1 | Endoplasmic reticulum-Golgi intermediate compartment protein 1 (ER-Golgi intermediate compartment 32 kDa protein) (ERGIC-32) | 0.40 | 2.99E-04 |
| Q5W0V3 | FHIP2A | FHF complex subunit HOOK interacting protein 2A (FHIP2A) | 0.40 | 5.48E-03 |
| O96028 | NSD2 | Histone-lysine N-methyltransferase NSD2 (EC 2.1.1.357) (Multiple myeloma SET domain-containing protein) (MMSET) (Nuclear SET domain-containing protein 2) (Protein trithorax-5) (Wolf-Hirschhorn syndrome candidate 1 protein) | 0.40 | 1.16E-02 |
| A0AVF1 | TTC26 | Intraflagellar transport protein 56 (Tetratricopeptide repeat protein 26) (TPR repeat protein 26) | 0.40 | 4.63E-02 |
| Q8IUZ5 | PHYKPL | 5-phosphohydroxy-L-lysine phospho-lyase (EC 4.2.3.134) (Alanine--glyoxylate aminotransferase 2-like 2) | 0.40 | 1.02E-03 |
| P63000 | RAC1 | Ras-related C3 botulinum toxin substrate 1 (EC 3.6.5.2) (Cell migration-inducing gene 5 protein) (Ras-like protein TC25) (p21-Rac1) | 0.40 | 1.25E-02 |
| O00214 | LGALS8 | Galectin-8 (Gal-8) (Po66 carbohydrate-binding protein) (Po66-CBP) (Prostate carcinoma tumor antigen 1) (PCTA-1) | 0.40 | 1.86E-04 |
| Q7L8J4 | SH3BP5L | SH3 domain-binding protein 5-like (SH3BP-5-like) | 0.40 | 1.38E-02 |
| Q9NV66 | TYW1 | S-adenosyl-L-methionine-dependent tRNA 4-demethylwyosine synthase TYW1 (EC 4.1.3.44) (Radical S-adenosyl methionine and flavodoxin domain-containing protein 1) (tRNA wybutosine-synthesizing protein 1 homolog) (tRNA-yW-synthesizing protein) | 0.40 | 4.69E-02 |
| Q9UBV8 | PEF1 | Peflin (PEF protein with a long N-terminal hydrophobic domain) (Penta-EF hand domain-containing protein 1) | 0.40 | 1.46E-02 |
| Q8IV48 | ERI1 | 3'-5' exoribonuclease 1 (EC 3.1.-.-) (3'-5' exonuclease ERI1) (Eri-1 homolog) (Histone mRNA 3'-end-specific exoribonuclease) (Histone mRNA 3'-exonuclease 1) (Protein 3'hExo) (HEXO) | 0.39 | 4.00E-02 |
| Q8IX04 | UEVLD | Ubiquitin-conjugating enzyme E2 variant 3 (UEV-3) (EV and lactate/malate dehydrogenase domain-containing protein) | 0.39 | 2.25E-02 |
| Q5VV42 | CDKAL1 | Threonylcarbamoyladenosine tRNA methylthiotransferase (EC 2.8.4.5) (CDK5 regulatory subunit-associated protein 1-like 1) (tRNA-t(6)A37 methylthiotransferase) | 0.39 | 1.38E-05 |
| Q9H7C9 | AAMDC | Mth938 domain-containing protein (Adipogenesis associated Mth938 domain-containing protein) | 0.39 | 9.05E-04 |
| Q03112 | MECOM | Histone-lysine N-methyltransferase MECOM (EC 2.1.1.367) (Ecotropic virus integration site 1 protein homolog) (EVI-1) (MDS1 and EVI1 complex locus protein) (Myelodysplasia syndrome 1 protein) (Myelodysplasia syndrome-associated protein 1) | 0.39 | 3.14E-03 |
| Q93096 | PTP4A1 | Protein tyrosine phosphatase type IVA 1 (EC 3.1.3.48) (PTP(CAAXI)) (Protein-tyrosine phosphatase 4a1) (Protein-tyrosine phosphatase of regenerating liver 1) (PRL-1) | 0.39 | 2.79E-03 |
| Q9NUP1 | BLOC1S4 | Biogenesis of lysosome-related organelles complex 1 subunit 4 (BLOC-1 subunit 4) (Protein cappuccino homolog) | 0.39 | 3.33E-02 |
| Q9BSD7 | NTPCR | Cancer-related nucleoside-triphosphatase (NTPase) (EC 3.6.1.15) (Nucleoside triphosphate phosphohydrolase) | 0.39 | 2.13E-03 |
| Q9BQE3 | TUBA1C | Tubulin alpha-1C chain (EC 3.6.5.-) (Alpha-tubulin 6) (Tubulin alpha-6 chain) [Cleaved into: Detyrosinated tubulin alpha-1C chain] | 0.39 | 2.65E-03 |
| Q9BX69 | CARD6 | Caspase recruitment domain-containing protein 6 | 0.39 | 4.68E-04 |
| Q9UKY1 | ZHX1 | Zinc fingers and homeoboxes protein 1 | 0.39 | 4.60E-02 |
| Q8WX93 | PALLD | Palladin (SIH002) (Sarcoma antigen NY-SAR-77) | 0.39 | 2.28E-03 |
| Q9H1K0 | RBSN | Rabenosyn-5 (110 kDa protein) (FYVE finger-containing Rab5 effector protein rabenosyn-5) (RAB effector RBSN) (Zinc finger FYVE domain-containing protein 20) | 0.39 | 1.95E-02 |
| Q96J01 | THOC3 | THO complex subunit 3 (Tho3) (TEX1 homolog) (hTREX45) | 0.39 | 4.95E-02 |
| O43414 | ERI3 | ERI1 exoribonuclease 3 (EC 3.1.-.-) (Prion interactor 1) (Prion protein-interacting protein) | 0.39 | 4.11E-02 |
| P48147 | PREP | Prolyl endopeptidase (PE) (EC 3.4.21.26) (Post-proline cleaving enzyme) | 0.38 | 4.45E-03 |
| A0JNW5 | UHRF1BP1L | Bridge-like lipid transfer protein family member 3B (Syntaxin-6 Habc-interacting protein of 164 kDa) (UHRF1-binding protein 1-like) | 0.38 | 1.84E-03 |
| Q9UMX3 | BOK | Bcl-2-related ovarian killer protein (hBOK) (Bcl-2-like protein 9) (Bcl2-L-9) | 0.38 | 5.85E-03 |
| Q8TB03 | CXorf38 | Uncharacterized protein CXorf38 | 0.38 | 1.18E-02 |
| Q6P4R8 | NFRKB | Nuclear factor related to kappa-B-binding protein (DNA-binding protein R kappa-B) (INO80 complex subunit G) | 0.38 | 9.87E-03 |
| P55786 | NPEPPS | Puromycin-sensitive aminopeptidase (PSA) (EC 3.4.11.14) (Cytosol alanyl aminopeptidase) (AAP-S) | 0.38 | 6.37E-05 |
| P54284 | CACNB3 | Voltage-dependent L-type calcium channel subunit beta-3 (CAB3) (Calcium channel voltage-dependent subunit beta 3) | 0.38 | 4.46E-03 |
| Q96PU8 | QKI | KH domain-containing RNA-binding protein QKI (Protein quaking) (Hqk) (HqkI) | 0.38 | 7.77E-05 |
| Q9GZM8 | NDEL1 | Nuclear distribution protein nudE-like 1 (Protein Nudel) (Mitosin-associated protein 1) | 0.38 | 1.05E-03 |
| Q06481 | APPL2 | Amyloid beta precursor like protein 2 (APPH) (Amyloid beta (A4) precursor-like protein 2) (Amyloid protein homolog) (Amyloid-like protein 2) (APLP-2) (CDEI box-binding protein) (CDEBP) (Sperm membrane protein YWK-II) | 0.38 | 1.59E-05 |
| Q96Q05 | TRAPPC9 | Trafficking protein particle complex subunit 9 (NIK- and IKBKB-binding protein) (Tularik gene 1 protein) | 0.38 | 3.51E-04 |
| Q7Z589 | EMSY | BRCA2-interacting transcriptional repressor EMSY | 0.38 | 1.34E-03 |
| O75051 | PLXNA2 | Plexin-A2 (Semaphorin receptor OCT) | 0.38 | 1.46E-04 |
| Q6ZVL6 | KIAA1549L | UPF0606 protein KIAA1549L | 0.38 | 8.41E-03 |
| Q9H2H8 | PPIL3 | Peptidyl-prolyl cis-trans isomerase-like 3 (PPIase) (EC 5.2.1.8) (Cyclophilin J) (CyPJ) (Cyclophilin-like protein PPIL3) (Rotamase PPIL3) | 0.38 | 4.55E-04 |
| Q68DK2 | ZFYVE26 | Zinc finger FYVE domain-containing protein 26 (FYVE domain-containing centrosomal protein) (FYVE-CENT) (Spastizin) | 0.38 | 3.51E-02 |
| Q6GYQ0 | RALGAPA1 | Ral GTPase-activating protein subunit alpha-1 (GAP-related-interacting partner to E12) (GRIPE) (GTPase-activating Rap/Ran-GAP domain-like 1) (Tuberin-like protein 1) (p240) | 0.38 | 4.62E-02 |
| P78314 | SH3BP2 | SH3 domain-binding protein 2 (3BP-2) | 0.38 | 2.64E-02 |
| Q86WV6 | STING1 | Stimulator of interferon genes protein (hSTING) (Endoplasmic reticulum interferon stimulator) (ERIS) (Mediator of IRF3 activation) (hMITA) (Transmembrane protein 173) | 0.38 | 7.03E-04 |
| Q96RL1 | UIMC1 | BRCA1-A complex subunit RAP80 (Receptor-associated protein 80) (Retinoid X receptor-interacting protein 110) (Ubiquitin interaction motif-containing protein 1) | 0.38 | 1.93E-02 |
| P50453 | SERPINB9 | Serpin B9 (Cytoplasmic antiproteinase 3) (CAP-3) (CAP3) (Peptidase inhibitor 9) (PI-9) | 0.38 | 4.42E-05 |
| O00592 | PODXL | Podocalyxin (GCTM-2 antigen) (Gp200) (Podocalyxin-like protein 1) (PC) (PCLP-1) | 0.38 | 2.93E-02 |
| Q14689 | DIP2A | Disco-interacting protein 2 homolog A (DIP2 homolog A) (EC 6.2.1.1) | 0.37 | 1.89E-02 |
| O43639 | NCK2 | Cytoplasmic protein NCK2 (Growth factor receptor-bound protein 4) (NCK adaptor protein 2) (Nck-2) (SH2/SH3 adaptor protein NCK-beta) | 0.37 | 2.58E-04 |
| P52815 | MRPL12 | Large ribosomal subunit protein bL12m (39S ribosomal protein L12, mitochondrial) (L12mt) (MRP-L12) (5c5-2) | 0.37 | 4.95E-02 |
| Q86U28 | ISCA2 | Iron-sulfur cluster assembly 2 homolog, mitochondrial (HESB-like domain-containing protein 1) | 0.37 | 4.65E-03 |
| Q9ULI3 | HEG1 | Protein HEG homolog 1 | 0.37 | 2.38E-03 |
| P32856 | STX2 | Syntaxin-2 (Epimorphin) | 0.37 | 4.37E-03 |
| Q01813 | PFKP | ATP-dependent 6-phosphofructokinase, platelet type (ATP-PFK) (PFK-P) (EC 2.7.1.11) (6-phosphofructokinase type C) (Phosphofructo-1-kinase isozyme C) (PFK-C) (Phosphohexokinase) | 0.37 | 7.43E-04 |
| O95084 | PRSS23 | Serine protease 23 (EC 3.4.21.-) (Putative secreted protein Zsig13) | 0.37 | 2.59E-03 |
| O60487 | MPZL2 | Myelin protein zero-like protein 2 (Epithelial V-like antigen 1) | 0.37 | 3.97E-02 |
| Q9Y2Q5 | LAMTOR2 | Ragulator complex protein LAMTOR2 (Endosomal adaptor protein p14) (Late endosomal/lysosomal Mp1-interacting protein) (Late endosomal/lysosomal adaptor and MAPK and MTOR activator 2) (Mitogen-activated protein-binding protein-interacting protein) (MAPBP-interacting protein) (Roadblock domain-containing protein 3) | 0.37 | 2.09E-02 |
| Q9NZN4 | EHD2 | EH domain-containing protein 2 (PAST homolog 2) | 0.37 | 2.91E-03 |
| Q9H147 | DNTTIP1 | Deoxynucleotidyltransferase terminal-interacting protein 1 (Terminal deoxynucleotidyltransferase-interacting factor 1) (TdIF1) (TdT-interacting factor 1) | 0.37 | 3.51E-04 |
| P18084 | ITGB5 | Integrin beta-5 | 0.37 | 7.10E-03 |
| Q9NYL9 | TMOD3 | Tropomodulin-3 (Ubiquitous tropomodulin) (U-Tmod) | 0.37 | 3.97E-04 |
| P49761 | CLK3 | Dual specificity protein kinase CLK3 (EC 2.7.12.1) (CDC-like kinase 3) | 0.37 | 3.95E-03 |
| Q13641 | TPBG | Trophoblast glycoprotein (5T4 oncofetal antigen) (5T4 oncofetal trophoblast glycoprotein) (5T4 oncotrophoblast glycoprotein) (M6P1) (Wnt-activated inhibitory factor 1) (WAIF1) | 0.37 | 2.38E-03 |
[truncated: 345,326 more chars]
